# Cnidarian Venoms in the Omics Era: Toxin Discovery and Bioactive Potential

**DOI:** 10.3390/md24090306

**Published:** 2026-09-01

**Authors:** R. Alexandre Barroso, Agostinho Antunes

**Affiliations:** 1 CIIMAR/CIMAR, Interdisciplinary Centre of Marine and Environmental Research, University of Porto, Terminal de Cruzeiros do Porto de Leixões, Av. General Norton de Matos, s/n, 4450-208 Porto, Portugal; barrosoalex98@gmail.com; 2 Department of Biology, Faculty of Sciences, University of Porto, Rua do Campo Alegre, 4169-007 Porto, Portugal

**Keywords:** cnidaria, toxin, venom, omics, bioactive

## Abstract

Cnidaria is a phylum of aquatic invertebrates that includes jellyfish, sea anemones and corals. Their venoms are among the oldest in the animal kingdom and an important source of bioactive molecules. Advances in omics technologies have revolutionized toxin discovery, yet knowledge of cnidarian toxin diversity remains fragmented across databases and individual studies. To address this gap, this review integrates information from 342 toxins curated in the ToxProt database, 76 additional toxins reported in the literature, and evidence from 81 proteomic and 31 transcriptomic studies. Toxins have been characterized from 145 species, with sea anemones accounting for the largest number of studied species (65). Cnidarian toxins are used for prey capture, predator deterrence, territorial competition and digestion. These include toxic enzymes, neurotoxins, protease inhibitors and pore-forming toxins, among others. To date, the three-dimensional structures of 37 toxins have been resolved, and 78 have reported biomedical or biotechnological potential, including antiarrhythmic, analgesic, anticancer, and insecticidal activities. According to omics studies, metalloproteinases, phospholipases, lectins, Kunitz-like peptides, venom coagulation factor and CRiSP/CAP toxins are the most widespread, whereas neurotoxins show a more restricted taxonomic distribution. The first venom-related transcriptomic analysis of three Corallimorpharia and two Antipatharia (black corals) species demonstrates putative pore-forming toxins, while black corals also harbor putative neurotoxins. Overall, this review synthesizes the current knowledge of cnidarian venoms and highlights their remarkable diversity and promise as a source of bioactive molecules.

## 1. Introduction

All living organisms on Earth, from microorganisms to plants and animals, evolved distinct strategies to survive in competitive environments. Among animals, one such strategy is venom production. More than 220,000 venomous species are known, representing approximately 15% of all described animal biodiversity [1]. Venoms are complex mixtures of peptides, proteins, and other bioactive molecules, collectively referred to as toxins, that disrupt the physiology of target organisms following venom delivery and mediate functions such as prey capture, predator defense, competition, digestion, offspring protection and antimicrobial defense [2,3,4]. At the molecular level, many toxin genes originate through gene duplication events followed by recruitment into the venom and the acquisition of novel or partial toxic functions [5]. Repeated gene duplications generate diverse toxin gene families, while others become pseudogenized under the birth-and-death model of evolution [3,6]. Although many toxin families have been convergently recruited into venoms across distantly related animal lineages, venom systems appear to have evolved more than 100 times independently during metazoan evolution, creating unique venom compositions and molecular targets [2,4].

Although invertebrates constitute more than 90% of all extant animal species, little is known about their venom [1]. These include the phylum Cnidaria, widely regarded as the earliest-diverging extant venomous animal lineage, with molecular clock analysis dating its origin to the Tonian–Cryogenian (~798 Ma) [7]. Unlike evolutionary younger venomous lineages (e.g., snakes and cone snails), whose toxins continue to diversify under positive selection associated with recent ecological specialization, toxins from ancient lineages (e.g., cnidarians, coleoids, spiders and centipedes) are predominantly shaped by purifying selection to preserve toxin potency [8,9]. Around 13,300 cnidarian species have been described, showing diverse morphologies, life cycles and habitats, ranging from pelagic marine and freshwater forms to sessile benthic species [10]. Cnidarians have a simple body plan with two epithelial layers, a diffuse nerve net, and a single oral opening surrounded by tentacles. However, their defining feature is the cnidocyte, a specialized stinging cell that contains a secretory organelle called cnidocyst. The most common cnidocyst type, the nematocyst, is distributed across the body, varies in morphology and size, and rapidly injects venom upon contact with prey or predators [11,12]. Although most cnidarian stings cause only mild symptoms in humans, some species rank among the most venomous animals in the world. The Australian box jellyfish (*Chironex fleckeri*) [13] and the Portuguese man o’ war (*Physalia physalis*) [14] are capable of causing severe envenomation in humans.

**Figure 1:**
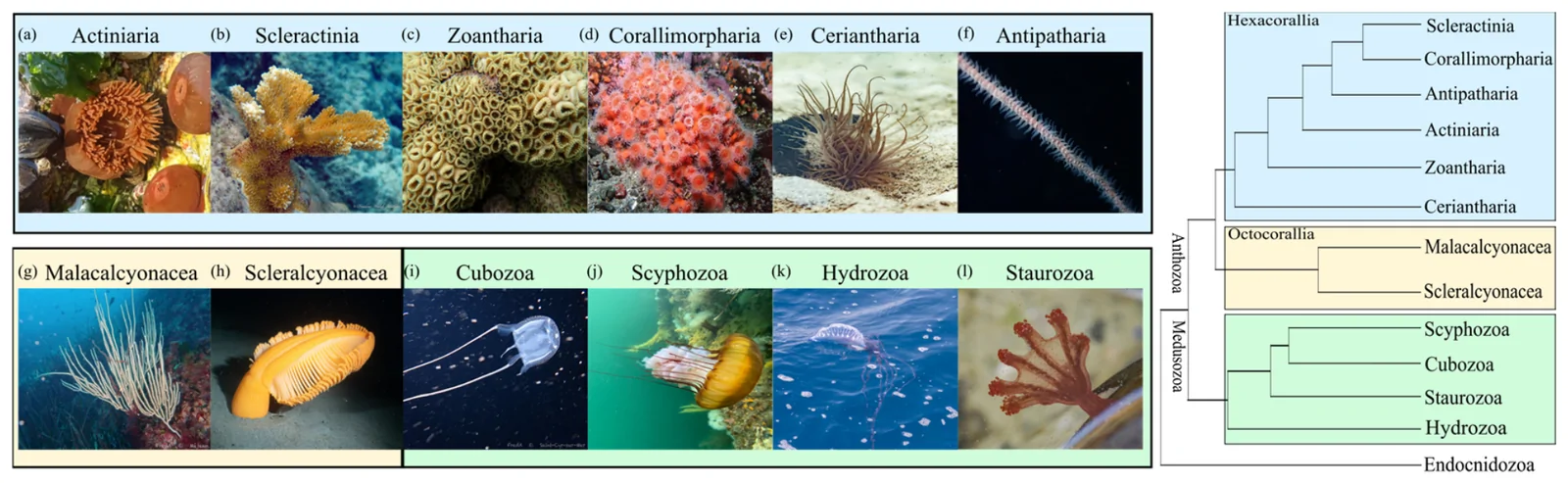
Representative diversity of the phylum Cnidaria, showing the three subphyla: Anthozoa, Medusozoa, and Endocnidozoa. Phylogeny follows previous studies [7,10]. (**a**) *Actinia equina*; (**b**) *Acropora palmata*; (**c**) *Palythoa caribaeorum*; (**d**) *Corynactis californica*; (**e**) *Pachycerianthus delwynae*; (**f**) *Stichopathes luetkeni*; (**g**) *Eunicella singularis*; (**h**) *Ptilosarcus gurneyi*; (**i**) *Carybdea marsupialis*; (**j**) *Chrysaora fuscescens*; (**k**) *Physalia physalis*; (**l**) *Calvadosia campanulata*. Photograph sources and Creative Commons license attributions are provided in Table S1.

Cnidaria is divided into three major subphyla. Anthozoa includes mostly sessile species without a medusa stage and comprises Octocorallia (soft corals, blue corals, sea pens and gorgonians) and Hexacorallia (sea anemones, stony corals, zoanthids, corallimorphs, cerianthids and black corals). Medusozoa comprises mainly free-living species with a medusa stage, including jellyfish, hydroids, and siphonophores. Endocnidozoa consists of obligate endoparasites, including myxozoans and the monotypic Polypodiozoa [7,10,12,15] (Figure 1).

**Figure 2:**
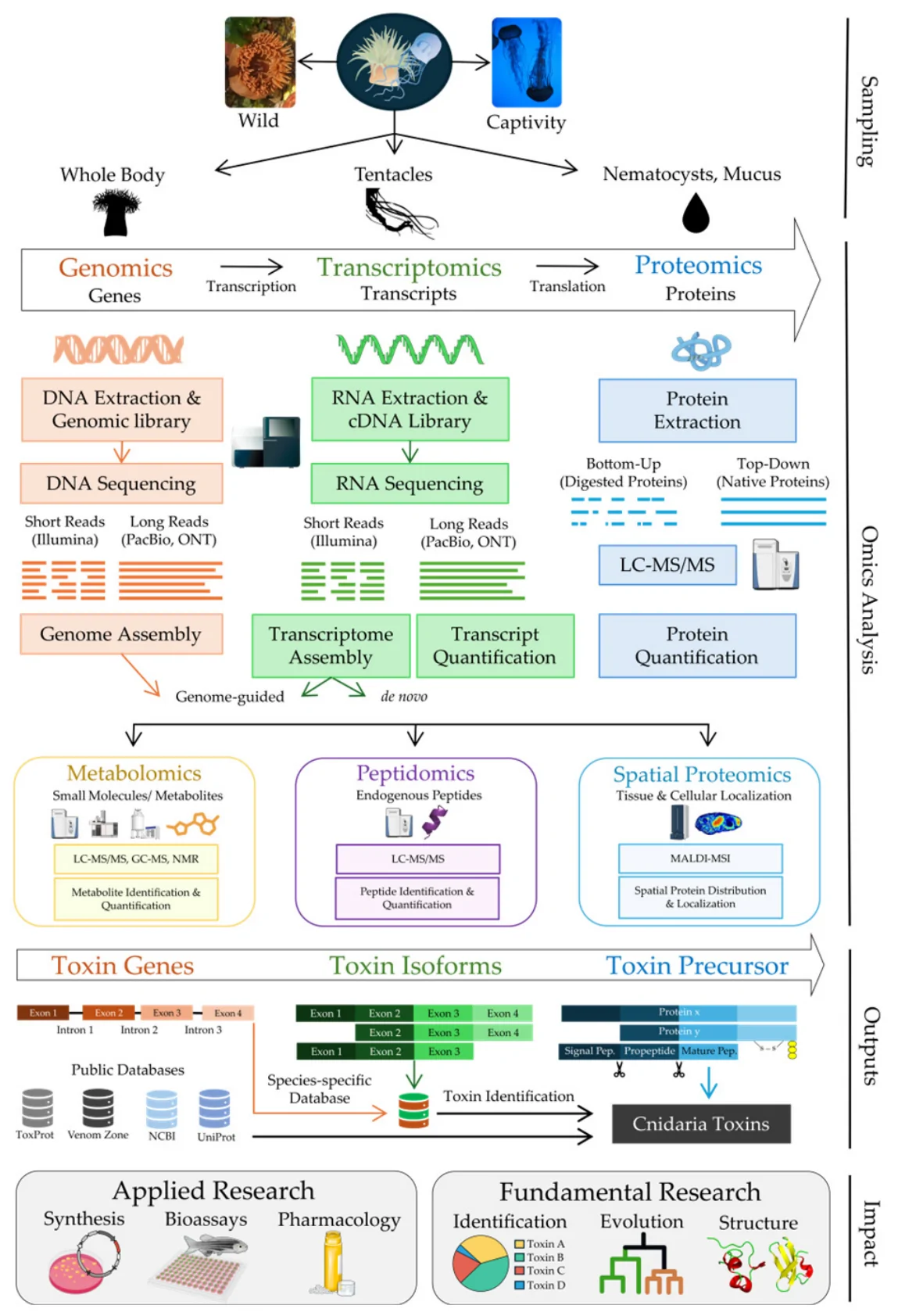
Multi-omics workflow for toxin discovery in Cnidaria. Biological samples are analyzed using genomic, transcriptomic, and proteomic approaches, which may be complemented by metabolomics, peptidomics and spatial proteomics. The integration of public toxin databases with species-specific genomics and transcriptomic resources enables the identification of cnidarian toxins from proteomic datasets, supporting both fundamental and applied toxin research. Colors indicate the genomic (orange), transcriptomic (green), and proteomic (blue) approaches.

The evolution of high-throughput omics technologies (genomics, transcriptomics and proteomics), together with novel bioinformatic tools for processing next-generation sequencing data, has accelerated toxin discovery in venomous organisms that are small, rare or difficult to maintain under laboratory conditions, including cnidarians [1,16], enabling the detection of low-abundance compounds while reducing the need for extensive animal sampling (Figure 2). As all cnidarians are thought to produce venom and omics datasets continue to expand rapidly, studies are needed to integrate and interpret this growing body of information. This review differs from previous works by providing an extensive synthesis of cnidarian protein toxins while emphasizing discoveries enabled by omics approaches. Specifically, this review (1) summarizes the major toxin families described in the ToxProt database, the literature, and omics studies; (2) discusses the structural characteristics of each toxin family; (3) reviews toxins with reported biomedical and biotechnological potential; and (4) synthesizes proteomic and transcriptomic evidence of toxin diversity across the major cnidarian groups, including the first characterization of putative toxin repertoires of the unexplored orders Corallimorpharia and Antipatharia.

## 2. Cnidaria Toxin Families

**Figure 3:**
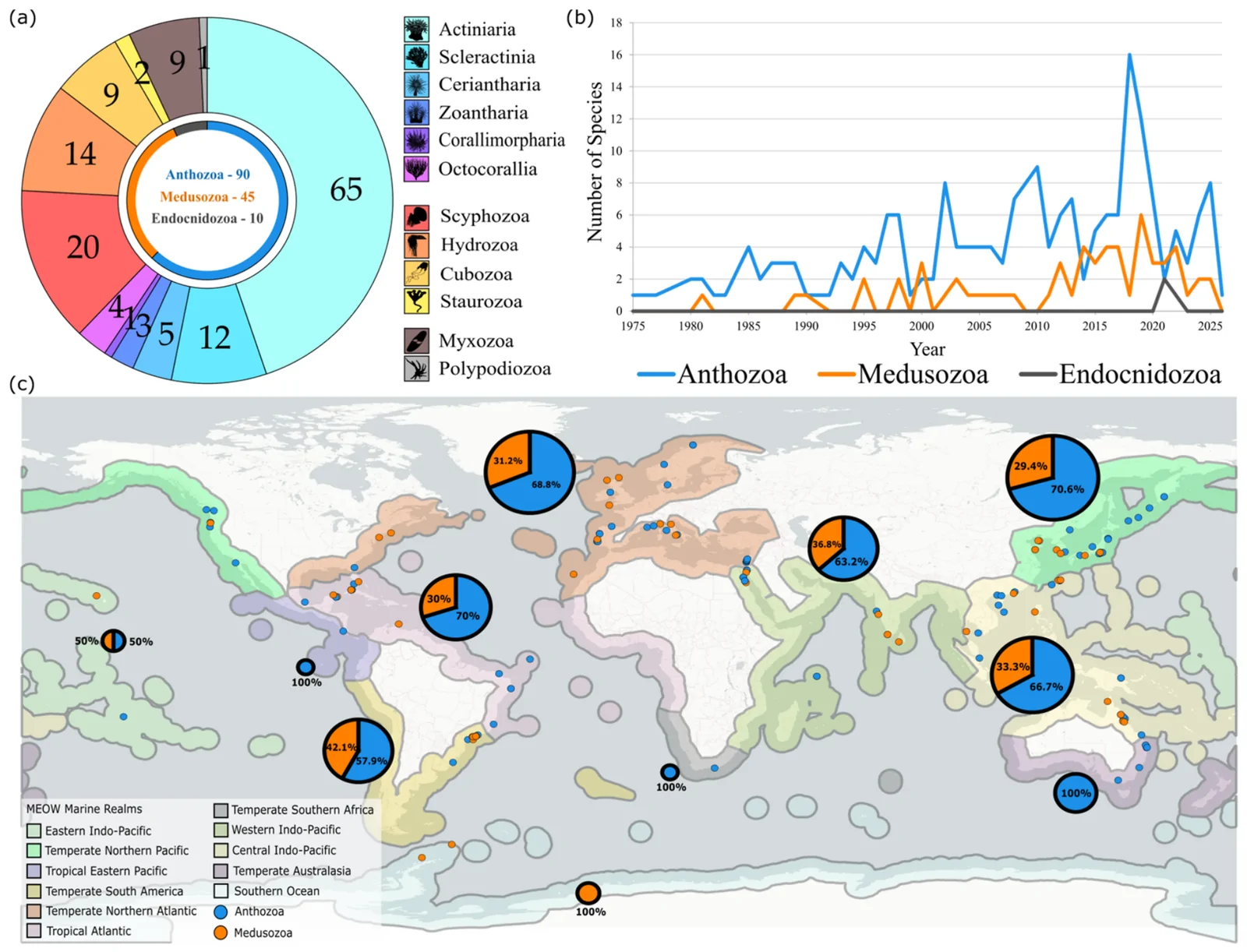
Overview of cnidarian venom studies. (**a**) Number of unique cnidarian species included. (**b**) Timeline of the first toxin reports or venom-related proteomic and transcriptomic studies in each subphylum (1975–2026; 254 studies and six ToxProt entries not associated with any study; Table S7). (**c**) Geographical distribution of sampling localities across the Marine Ecoregions of the World (MEOW) realms [18] (Arctic excluded; 164 sampling locations; File S1). The map was generated in QGIS v3.40.5 (https://qgis.org/ (accessed on 20 January 2026)).

This review includes information from 342 toxins curated from the ToxProt database (Table S2), 76 additional toxins reported in the literature (Table S3), 81 proteomic studies (Table S4), and 31 transcriptomic studies (Table S5). The most common toxin families include toxic enzymes, neurotoxins, and pore-forming toxins, among others. However, it is estimated that ~29,000 cnidarian toxins remain undiscovered [17]. Actiniaria is by far the most studied order, accounting for 82.3% (344/418) of all cnidarian toxins reported in this review, followed by Hydrozoa (5.3%), Scleractinia (5.0%), Cubozoa (3.1%), Scyphozoa (2.4%) and Zoantharia (1.9%), highlighting the strong taxonomic bias in cnidarian venom research. Among the 145 unique cnidarian species included in the studies compiled in this review, Actiniaria accounted for the largest number of species (65), followed by Scyphozoa (20), Hydrozoa (14) and Scleractinia (12) (Figure 3a, Table S7). The greatest increase in first reports of toxin identification or venom characterization through omics analysis occurred in 2018–2019, especially for Anthozoa (Figure 3b, Table S7). Overall, Anthozoa and Medusozoa were mostly sampled in the Temperate Northern Pacific (70.6% and 29.4%), Temperate Northern Atlantic (68.8% and 31.2%) and Central Indo-Pacific (66.7% and 33.3%) marine realms, respectively (Figure 3c, Table S7).

### 2.1. Toxic Enzymes

#### 2.1.1. Phospholipase A2

Phospholipase A2 (PLA_2_) hydrolyzes the sn-2 acyl bond of glycerophospholipids, releasing free fatty acids, such as arachidonic acid, and lysophospholipids, which serve as precursors of bioactive lipid mediators involved in inflammation and other physiological processes [19]. The PLA_2_ superfamily is divided into 15 groups and many subgroups, including five distinct types of enzymes, such as the secreted PLA_2_ (sPLA_2_). sPLA_2_s are low molecular weight, Ca^2+^-dependent enzymes containing a catalytic histidine residue. While pancreatic sPLA_2_s function primarily as digestive enzymes, other sPLA_2_s are active components of snake and other animal venoms. Venom-associated sPLA_2_s belong to Group-IA (cobras and kraits), IIA (rattlesnakes), IIB (Gaboon viper), III (lizards, bees) and IX (cone snails) [19,20]. Several PLA_2_s have also been identified in Cnidaria (Table 1).

Sea anemone PLA_2_s retain the conserved catalytic machinery of sPLA_2_s but form a distinct cnidarian lineage that differs structurally from vertebrate venom PLA2s [21,22]. Within this lineage, CgPLA2, AcPLA2, and a PLA_2_ from *Nematostella vectensis* form a well-supported monophyletic clade, whereas UcPLA2, which carries a C27N substitution disrupting the conserved cysteine (Cys)27–Cys126 disulfide bond, clusters separately [22,23]. PLA_2_s from *Bunodosoma caissarum* and the jellyfish *Rhopilema nomadica* also show moderate and high homology to group III PLA_2_s, respectively [24,25]. Their localization in tentacles [20,21,24] and defensive acontia [20,21,26,27] suggests a role in prey capture and defense. Curiously, the PLA_2_ from the zoanthid *Palythoa caribaeorum* is the first neurotoxic cnidarian PLA_2_ reported [28]. PLA_2_ activity has been reported in 22 cnidarian species, including octocorallians, sea anemones, stony corals, hydrozoans, scyphozoans and cubozoans [20]. The highest activities were detected in the hydrozoan fire coral *Millepora* sp. (735 U/g) and the stony coral *Pocillopora damicornis* (693 U/g), which cause skin irritation upon contact. In sea anemones, PLA_2_ activity was higher in acontia than in whole animal extracts, whereas among cubozoans, the highest activity was detected in the tentacles of the highly venomous *Chironex fleckeri* [20]. More recently, a non-invasive venom extraction method identified PLA2 activity in venom extracts from 12 additional species of sea anemones, stony coral, and scyphozoan species, with the highest activities recorded in *Thalassianthus aster* (61.8 U/g) and *Seriatopora caliendrum* (22.9 U/g) [29].

**Table 1:** Representative toxins from the PLA_2_ family from Cnidaria.

Name	Species	UniProt	LD_50_ (μg/kg)	Ref.
BcPLA2	*Bunodosoma caissarum*	P86780	-	[25]
AcPLA2	*Calliactis palliata*	Q8WS88	-	[21]
PLA2-Cpp1a	*Calliactis polypus*	P0DY42	-	[26,27]
CgPLA2	*Condylactis gigantea*	D2X8K2	-	[22]
Milleporin 1	*Millepora platyphylla*	-	-	[30]
A2-PLTX-Pcb1a	*Palythoa caribaeorum*	-	-	[28]
Toxic PLA2	*Rhopilema nomadica*	P43318	6000 (Fish)	[24]
UcPLA2	*Urticina crassicornis*	A7LCJ2	-	[23]

LD_50_—median lethal dose.

#### 2.1.2. Zinc Metalloproteinases and Astacin-like Metalloproteinases

Zinc metalloproteinases (ZnMPs) are Zn^2+^-dependent proteolytic enzymes commonly found in animal venoms, including those of snakes, ticks and centipedes. In snake venoms, they contribute to hemorrhage, edema, blister formation, and inhibition of platelet aggregation [31,32]. ZnMPs may also contribute to the cytotoxic and inflammatory effects of cnidaria venoms, especially after jellyfish stings [33]. Consistently, venoms from four scyphozoan species exhibited gelatinolytic, caseinolytic, and fibrinolytic activities that were inhibited by a metalloproteinase inhibitor, with the highest cytotoxicity observed in *Cyanea nozakii* [34]. Proteomic analysis has identified putative ShK-containing metalloproteinases in the nematocysts of *C. fleckeri* [35] and *Actinia fragacea* [36], as well as in whole-body extracts of *Pelagia noctiluca* [37]. In *Rhizostoma pulmo*, a ZnMP, Rhizoprotease, was isolated from a fibrinogenolytic venom fraction [38], and in *Phacellophora camtschatica*, it was the major erythrocyte-binding toxin [39]. In the hydromedusae *Podocoryna carnea*, a ZnMP transcript is highly expressed during development and feeding, suggesting roles in tissue remodeling, cell migration, and digestion [40]. Two astacin-family metalloproteinases, NEP-6 (K7Z9Q9) and NEP-14, from *N. vectensis*, are expressed in both nematocysts and pharyngeal gland cells. These proteins originated through recruitment of the Tolloid (also known as BMP1, bone morphogenetic protein 1), which has a conserved role in animal development, but lack the C-terminal CUB domains that normally control target specificity, as observed in spider venom astacin-like toxins, potentially enhancing their toxicity [41].

### 2.2. Neurotoxins

#### 2.2.1. Sea Anemone Sodium Channel Toxin Family

Sea anemone sodium channel toxins (NaTxs) target voltage-gated sodium (Na_v_) channels, delaying channel inactivation and prolonging action potentials [42,43]. NaTxs are divided into four types based on Cys arrangement, amino acid (aa) composition, structure, and immunological cross-reactivity.

Long NaTx, mostly from *Anthopleura*, *Anemonia* and *Bunodosoma* (Actiniidae), are classified as Type I, while others from *Heteractis* and *Stichodactyla* belong to Type II (Table 2). Although both types share a common ancestral origin, they exhibit low sequence homology (~30% between types versus >60% within types), lack immunological cross-reactivity [44], and differ in their interaction with Na_v_ channels [45,46]. Among Type II NaTxs, *N. vectensis* toxin 1 (Nv1) [47] and Halcurin [48] are considered the most basal sea anemone neurotoxins [35]. Nv1 is encoded by a highly repetitive genomic region evolving primarily under concerted evolution, resulting in highly similar toxin genes within species. Its multicopy organization and low sequence polymorphism have been associated with the specialized diet and restricted habitat of *N. vectensis* [47]. However, some Nv1 paralogs have escaped concerted evolution, suggesting additional evolutionary forces shaping toxin diversity [49]. In contrast, type I NaTx exhibit lineage-specific diversification. Av2 from *Anemonia viridis* is encoded by six nearly identical genes, where three paralogs have greater sequence diversity with evidence of positive selection on Av9; *Actinia equina ae1* and *ae2* toxin genes suffer recombinant events [47]; and *Anthopleura* species contain highly homologous neurotoxins within the same species, likely contributing to distinct roles in prey capture and defense [46,50,51].

Type III NaTx are short peptides (~30 aa) from the family Actiniidae [43]. The representative toxin Av3 from *Anemonia sulcata* binds with high affinity to the arthropod Na_v_ channel [43]. Other members include PaTX [101] and the homologous PaTX-like peptides Da I/Da II and Er I [101,102], all sharing similar toxicity towards crabs (Table 3). Type III NaTx have also been detected in the transcriptomes of *A. elegantissima* [103], *B. goanense* [76], *Heterodactyla hemprichii* and *S. haddoni* [104].

Moreover, a distinct class of NaTx shares the mechanism of type I and II NaTx, despite having low sequence homology [105]. Representatives include calitoxins from *Calliactis* [105] and AdE-1 from *Exaiptasia diaphana* [106] (Table 3).

**Table 3:** Representative toxins from the Type III NaTx and the distinct class of NaTx from Cnidaria.

Name	Type	Species	UniProt	LD_50_ (μg/kg)	Targets	Ref.
Av3	III	*Anemonia sulcata*	P01535	<50 (Crabs, LD_100_)	DmNa_v_1	[43,56,107]
Av7	III	*Anemonia viridis*	C3TS04	-	DmNa_v_1	[108]
Av10	III	*Anemonia viridis*	C3TS07	-	-	
Da I/Da II	III	*Dofleinia armata*	P0DMZ2	110 (Crabs)	-	[102]
Er I	III	*Entacmaea quadricolor*	P09949	100 (Crabs)	-	[102]
PaTX	III	*Exocoelactis actinostoloides*	-	10 (Crabs)	-	[101,109]
Calitoxin I	-	*Calliactis parasitica*	P14531	-	-	[105,110]
Calitoxin II	-	*Calliactis parasitica*	P49127	-	-	[105]
Calitoxin-Cpp1a	-	*Calliactis polypus*	P0DY39	-	-	[27]
AdE-1	-	*Exaiptasia diaphana*	E3P6S4	-	hNav1.5, K_v_ channels	[106,111]

LD_50_—median lethal dose; LD_100_—lethal dose (100%).

#### 2.2.2. Sea Anemone Potassium Channel Toxin Family

Sea anemone potassium channel toxins (KTxs) target voltage-gated potassium (K_v_) channels, inhibiting K^+^ conductance and delaying membrane repolarization following an action potential [112]. KTxs are classified into five groups based on disulfide bond pattern, sequence identity, peptide length, and channel-binding affinities.

Type I KTxs are short peptides that primarily block K_v_1 (Shaker-type) channels (Table 4) [113]. They are divided into subtypes 1a and 1b according to the number of residues between the second and third Cys residues from the N-terminus. Subtype 1a includes AETX-K [114], HmK [115] and ShK [116], with four residues in this region; subtype 1b includes Aek [117], Kaliseptine [118], BgK [119], and Avtx [120], with eight residues. The discovery of additional subtype 1a KTxs through molecular cloning supports a possible association between KTx subtype distribution and sea anemone taxonomy, with subtype 1a predominating in Stichodactylidae and Thalassianthidae, and subtype 1b in Actiniidae [114,121]. K_v_1-targeting KTxs have also been detected in crude extracts from several actiniarian families [121], suggesting that many sea anemones remain potential sources of neurotoxins.

Type II KTxs are long polypeptides homologous to Kunitz-type serine protease inhibitors. Several Type II KTxs exhibit dual activity by blocking K_v_ channels and inhibiting serine proteases, including kalicludins from *A. sulcata* (K_v_1.2 and trypsin inhibitor) [118] and APEKTx1 from *A. elegantissima* (K_v_1.1 and trypsin inhibitor) [137]. Additional Kunitz peptides have been identified in several sea anemone species, although many have only been characterized as protease inhibitors [138,139,140,141] or remain functionally uncharacterized [142,143,144] (Table 5). These peptides may act synergistically with other venom components by protecting injected toxins from degradation by endogenous prey proteases.

Type II KTxs evolution has been best characterized in *Radianthus crispa*, where Kunitz-like peptides are encoded by multigene families with distinct N-terminal motifs expressed in the tentacle venom: Gly-Ser (HCGS), Arg-Gly (HCRG), Gly-Asn (HCGN), and Gly-Gly (HCGG) [145]. Evolutionary analyses of the HCGS gene family suggest that these peptides originated from a common ancestral gene and diversified through gene duplication, primarily by substitutions at the reactive P1 residue (essential for serine protease inhibition). The ancestral peptides likely contained Arg at the P1 position (group III) and exhibited broad inhibitory activity against distinct proteases. Subsequently, substitution of Arg by Lys gave rise to peptides more specialized for serine protease inhibition (group I, including SHPI-I and II), whereas substitution by Thr (group II) generated peptides with novel functions, including selective protease inhibition (e.g., Inh VJ and Jn-IV) and ion channel modulation (e.g., APHC peptides targeting TRPV1 channels involved in pain signaling). This lineage-specific diversification likely contributed to the ecological adaptation of *R. crispa* by enabling interactions with a wider range of prey and predators [145,146].

The multigene family HCRG also includes multifunctional peptides such as HCRG1 and HCRG2, which combine K_v_ channel blockade with potent inhibition of trypsin and chymotrypsin [147,148]. Another member, HCRG21, acts as a TRPV1 antagonist [149]. Comparable evolutionary patterns have also been reported in *Radianthus magnifica*, where the HCIQ multigene family encodes peptides highly homologous to HCGS-peptides from *R. crispa*. These peptides retain Arg at the P1 position and show neuroprotective activities [150,151,152,153]. In addition, mucus-derived peptides from *R. magnifica* form a combinatorial library comprising GS and RG variants, resembling that of *R. crispa* [96]. Similar Type II KTx libraries have also been identified in *A. viridis*, including the AsKC/Kalicludins and SA5 peptide families [120].

**Table 5:** Representative toxins from the Type II KTx from Cnidaria.

Name	Species	UniProt (PDB)	Targets	Ref.
AEPI-I	*Actinia equina*	P0DMW6	-	[142]
AEPI-II	*Actinia equina*	P0DMW7	-	[142]
AEPI-III	*Actinia equina*	P0DMW8	-	[142]
AEPI-IV	*Actinia equina*	P0DMW9	-	[142]
AEAPI	*Actinia equina*	P0DMJ2	Trypsin, Plasmin	[138]
ATPI-I/ATPI-II	*Actinia tenebrosa*	A0A6P8HC43	Trypsin, Chymotrypsin, Kallikrein	[140]
Kalicludine-1	*Anemonia sulcata*	Q9TWG0	Trypsin, K_v_1.2	[118]
Kalicludine-2	*Anemonia sulcata*	Q9TWF9	Trypsin, K_v_1.2	[118]
Kalicludine-3	*Anemonia sulcata*	Q9TWF8	Trypsin, K_v_1.2	[118]
AsKC11	*Anemonia viridis*	P0DN15	GIRK1/2 channels, K_v_1.6	[120,154,155]
SA5 II	*Anemonia viridis*	P10280	-	[144]
AXPI-I	*Anthopleura* aff. *xanthogrammica*	P81547	Trypsin, Plasmin, Chymotrypsin, Elastase	[138,139]
AXPI-II	*Anthopleura* aff. *xanthogrammica*	P81548	Trypsin	[139]
AXPI-III	*Anthopleura* aff. *xanthogrammica*	P0DMX0	Trypsin, Chymotrypsin	[143]
APEKTx1	*Anthopleura elegantissima*	P86862	Trypsin, K_v_1.1	[137]
AFAPI I	*Anthopleura fuscoviridis*	P0DMJ3	Trypsin, Plasmin	[138]
AFAPI III	*Anthopleura fuscoviridis*	P0DMJ4	Trypsin, Plasmin	[138]
HMRG1	*Radianthus magnifica*	C0HK72	-	[96]
HMGS1	*Radianthus magnifica*	C0HK73	-	[96]
HMGS2	*Radianthus magnifica*	C0HK74	-	[96]
HMIQ3c1	*Radianthus magnifica*	A0A3G2FQK2	Trypsin, Chymotrypsin, Kallikrein, Elastase, P2X7 ion channels	[150,152,153]
HCRG1	*Radianthus crispa*	C0HJU6	Trypsin, Chymotrypsin, K_v_1.1, K_v_1.2, K_v_1.3, K_v_1.6, insect Shaker IR,	[147,148]
HCRG2	*Radianthus crispa*	C0HJU7	Trypsin, Chymotrypsin, K_v_1.1, K_v_1.2, K_v_1.3, K_v_1.6, insect Shaker IR	[147,148]
HCRG21	*Radianthus crispa*	P0DL86	Trypsin, Chymotrypsin, TRPV1	[149]
HCGS1.19	*Radianthus crispa*	P0DV04	Trypsin	[145,156]
HCGS1.20	*Radianthus crispa*	P0DV05	Trypsin	[145,156]
HCGS1.36	*Radianthus crispa*	P0DV06	Trypsin	[145,156]
HCGS1.10	*Radianthus crispa*	P0DV03	Trypsin	[145,156]
InhVJ	*Radianthus crispa*	P0DMJ5	Trypsin, Chymotrypsin	[157]
Jn-IV	*Radianthus crispa*	P16344	Trypsin	[158]
APHC1	*Radianthus crispa*	B2G331	Trypsin, Chymotrypsin, TRPV1	[159]
APHC2	*Radianthus crispa*	C0HJF4	Trypsin, Chymotrypsin	[160]
APHC3	*Radianthus crispa*	C0HJF3	Trypsin, Chymotrypsin, TRPV1	[160,161]
RmIn I	*Radianthus crispa*	P0DV07	Trypsin, Chymotrypsin	[162]
RmIn II	*Radianthus crispa*	P0DV02	Trypsin, Chymotrypsin	[162]
HCIQ4c7	*Radianthus crispa*	A0A6B7FA07	Trypsin, Elastase, P2X7 ion channels	[150,151]
HCIQ1c9	*Radianthus crispa*	A0A6B7FEJ3	Trypsin	[150,151]
HCIQ2c1	*Radianthus crispa*	A0A6B7FBD3	Trypsin, Chymotrypsin, Kallikrein, Elastase, Cathepsin G, TRPA1	[150,151,163]
SHTX III	*Stichodactyla haddoni*	B1B5I8	K_v_ channels	[100]
ShPI I	*Stichodactyla helianthus*	P31713	Trypsin, Chymotrypsin, Papain, Kallikrein, Elastase, Pepsin, Chymosin, K_v_1.1, K_v_1.2, K_v_1.6	[164,165,166]
ShPI II	*Stichodactyla helianthus*	P81129	-	[141]
PcKuz1	*Palythoa caribaeorum*	P0DQQ9	Trypsin, elastase (LD_50_: 25 μM (Zebrafish))	[167]
PcKuz2	*Palythoa caribaeorum*	P0DQR0	Trypsin, elastase (LD_50_ > 100 μM (Zebrafish))	[167]
PcKuz3	*Palythoa caribaeorum*	P0DQR1	Trypsin, elastase (LD_50_: 10–20 μM (Zebrafish))	[167]

LD_50_—median lethal dose.

Type III KTxs probably evolved from NaTxs under positive selection (ω:1.33), representing a unique evolutionary innovation within sea anemones [8], showing diverse pharmacological activities (Table 6) [168,169]. Blood Depressing Substance (BDS)-1 and BDS-2 from *A. sulcata* were the first selective K_v_3.4 channel blockers in cnidarians [170,171] and were characterized by their antihypertensive properties. In *A. elegantissima*, APETx1 shares 54% sequence identity with BDS-1, but instead targets cardiac K_v_11.1/hERG channels [172]. Its homolog, APETx2, targets ASIC3 and Na_v_1.8, both involved in pain signaling [173,174]; APETx4 modulates Kv10.1/hEAG1, which is overexpressed in many human tumors [175]; and a single mutation (Thr3Pro) in APETx3 abolishes the K_v_11.1/hERG activity, converting it into a potent Na_v_ modulator [176]. A multigene family of APETx-like peptides (Hmg) was identified in *R. magnifica*, with high sequence identity to Hcr 1b peptides of *R. crispa* [177]. Similar Type III KTx libraries have also been described in *A. viridis* [120,155], and APETX-like peptides were identified from the mucus transcriptome of *B. granulifera*, with some displaying toxicity towards crabs [98]. Furthermore, an APETX-like toxin from *A. elegantissima* was found to be downregulated under UV stress [178].

Type IV KTx, also known as Boundless β-Hairpin (BBH; structural class 9a), are short (~30 aa) peptides encoded by multicopy precursor proteins that primarily target ion channels [82,195,196] (Table 7). Following removal of the signal peptide and propeptide, these precursors release multiple identical or distinct mature peptides [60]. Precursors may contain up to six copies of mature toxin (e.g., Am I from *Antheopsis maculata*) or combinations of different mature peptides (e.g., Ms 9a-1 and Ms 9a-2 from *Metridium senile*), enabling sea anemones to rapidly produce higher amounts of toxins [60,82,196].

Type V KTx includes BcsTx3 from *B. caissarum* venom, which induces strong contractile paralysis in crabs by targeting Shaker IR K^+^ channels [197] (Table 7).

Type VI KTx are represented solely by the short 17 aa peptide AbeTx1 from *Actinia bermudensis*, which selectively targets Shaker-related K^+^ channels (Table 7) [198]. However, two transcripts were also detected in the transcriptome of *B. goanense* [76].

**Table 7:** Representative toxins from the Type IV/BBH, V and VI KTx from Cnidaria.

Name	Type	Species	UniProt	LD_50_ (μg/kg)	Targets	Ref.
Av-1	IV	*Anemonia viridis*	P0DMZ8	-	-	[120,155]
Av-2	IV	*Anemonia viridis*	P0DMZ9	-	-	[120,155]
Am I	IV	*Antheopsis maculata*	P69929	830 (Crabs)	-	[60]
Bcg III 21.75	IV	*Bunodosoma cangicum*	P86465	-	-	[74]
Bcg III 21.00	IV	*Bunodosoma cangicum*	P86466	-	-	[74]
Bcg III 23.41	IV	*Bunodosoma cangicum*	P86467	-	-	[74]
Hau II	IV	*Heteractis aurora*	A0A0P0UTI6	-	-	[82]
Hau III	IV	*Heteractis aurora*	A0A0P0UTQ7	-	-	[82]
U-HMTX-Hdu1a	IV	*Homostichanthus duerdeni*	C0HJB4	-	-	[199]
Ms 9a-1	IV	*Metridium senile*	A0A1R3S3A8	-	TRPA1	[195]
Ms 9a-2	IV	*Metridium senile*	A0A1R3S3A8	-	-	[195]
SHTX I	IV	*Stichodactyla haddoni*	P0C7W7	-	-	[100]
SHTX II	IV	*Stichodactyla haddoni*	P0C7W7	-	K_v_ channels	[100]
Ugr 9a-1	IV	*Urticina grebelnyi*	R4ZCU1	-	ASIC3	[196]
Ugr 9a-2	IV	*Urticina grebelnyi*	R4ZCU1	-	-	[196]
Ugr 9a-3	IV	*Urticina grebelnyi*	R4ZCU1	-	-	[196]
BcsTx3	V	*Bunodosoma caissarum*	C0HJC4	-	K_v_1.1, K_v_1.2, K_v_1.3, K_v_1.6, insect Shaker IR	[197]
MsePTx1	V	*Metridium senile*	P0DMD7	-	-	-
NvePTx1	V	*Nematostella vectensis*	A7RMN1	-	-	[12,197,200]
AbeTx1	VI	*Actinia bermudensis*	C0HJD9	-	K_v_1.1, K_v_1.2, K_v_1.3, K_v_1.6, Shaker IR	[198]

LD_50_—median lethal dose.

#### 2.2.3. Sea Anemone PHAB Family

Ate1a (P0DM22), a 17 aa peptide from *A. tenebrosa*, adopts a unique Proline-Hinged Asymmetric β-Hairpin (PHAB) fold. Ate1a-like prepropeptides display a conserved modular architecture consisting of a signal peptide, one or two Cys-containing propeptides, three Cys-free propeptides, and one or more PHAB domains. Phylogenetic analysis suggests that this family evolved through domain duplication and concerted evolution, explaining the high sequence conservation within and between species. Ate1a is mostly expressed in tentacles, supporting a role in prey capture rather than digestion. Consistently, it induced contractile paralysis in amphipods following injections, whereas exposure in the medium had no effect, supporting its role as a nematocyst-delivered neurotoxin. Ate1a inhibits K_v_1.1 (IC_50_: 353 nM), K_v_1.2 (IC_50_: 146 nM), K_v_1.3 (IC_50_: 3051 nM), K_v_1.6 (IC_50_: 191 nM), and partially blocks Shaker IR [201].

#### 2.2.4. Sea Anemone 8 (SA8) Toxin Family

The SA8 family shares superficial sequence similarity with ShK toxins [202] and was first identified in the *A. viridis* expressed sequence tag (EST) database (Avtx 1-5, P0DMZ3-P0DMZ7) [120]. In *Telmatactis stephensoni*, the SA8 family underwent extensive expansion through tandem and proximal gene duplications, together with a key inversion event that recruited one SA8 peptide into the venom [202,203]. This inverted gene exhibits a distinct expression pattern, being upregulated not only in the epidermis but also in gastrodermal structures, suggesting neofunctionalization following escape from the ancestral regulatory network [202]. Proteomic analysis confirmed that peptides derived from this gene are present in the milked venom [127] and localize to the defensive acontia, mesenterial filaments, and pedal disc [202]. Although the recombinant SA8 peptide weakly inhibits hK_v_1.2 channels (IC_50_: 40.8 µM) and shows no toxicity toward *D. melanogaster*, its abundance in defensive tissues s3uggests an auxiliary role in predator deterrence [202]. SA8 transcripts are also highly upregulated in the acrorhagi and tentacles of *A. tenebrosa* [203]. Also, proteomic analysis identified SA8 proteins in the venom of *Calliactis polypus* [27] and in the mucus and tentacles of *B. caissarum* [204].

#### 2.2.5. Cnidaria Small Cysteine Rich Proteins (SCRiPs)

SCRiPs are neurotoxins characterized by an eight-Cys framework with three Cys residues clustered in the C-terminal region, and were first identified through stony corals EST libraries [8,205]. Recombinant Amil-SCRiP2 (C0H691) and Amil-SCRiP3 (C0H692) from *Acropora millepora* induced paralysis and death in zebrafish larvae after approximately 200 min and 16 h of exposure (230 μg/mL), respectively. In contrast, no toxicity was observed in blowfly larvae, suggesting a possible vertebrate selectivity, despite their unknown molecular targets [8]. SCRiPs are among the most highly expressed toxin families in *Stylophora pistillata*, particularly at the branch bases [206], and may exhibit stage-specific expression during coral development [205,207]. Although highly duplicated in stony coral genomes [207], SCRiP transcripts have also been identified in sea anemones, being upregulated in the acrorhagi, suggesting an origin in the common ancestor of both orders [8,104,203]. SCRiPs are classified into four monophyletic clades: *SCRiP-α* (Acroporidae, under diversifying selection), *SCRiP-δ* (seven coral families), *SCRiP-γ* (four coral families, including Acroporidae), and *SCRiP-δ* (Acroporidae, Poritidae and sea anemones) [208,209]. As *SCRiP-δ* may be the most basal and has the largest purifying selection, together with a *SCRiP-δ* from *A. millepora* having the highest potency (Amil-SCRiP2) versus a SCRiP-α (Amil-SCRiP3), *SCRiP-δ* may be the main SCRiP acting in predator deterrence [8,208]. Recently, Hact-SCRiP1 (C0HM70) was isolated from the nematocysts of the stony coral *Heliofungia actiniformis* [210], whereas the SCRiP-like peptide Ueq 12-1 (C0HK26) from *Urticina eques* was isolated from ectodermal secretions. Ueq 12-1 exhibits TRPA1-potentiating and antimicrobial activities, suggesting roles in both prey capture and protection against bacterial infections following tentacle injury [211].

### 2.3. Pore-Forming Toxins (PFTs)

#### 2.3.1. Jellyfish Toxins

Jellyfish toxins (JFTs) are potent pore-forming cytolysins that disrupt cell membranes through the oligomerization of amphipathic α-helices in the N-terminal region, resulting in pore formation. JFTs induce cardiotoxicity, hemolysis, cutaneous inflammation, and necrosis [13,35,212,213]. Although JFTs have evolved under negative selection to preserve pore-forming activity, some residues show evidence of episodic positive selection [8], reflecting adaptation to different prey types [212,214]. JFTs are classified into two major types: Type I includes CfTx-1/2, CqTX-A, CytA1/A2 (*Malo kingi*), and CqTX-A-like (*Hydra magnipapillata*), whereas Type II comprises CfTX-A/B, CaTX-A, CrTX-A, CytB (*M. kingi*) and TX-1/2 (*Aurelia aurita*) (Table 8). CfTX-1/2 (Type I) are more cardiotoxic to rats, whereas CfTXA/B (Type II) have higher hemolytic activity in vitro and may target other cell types. Together with the lack of cross-reactivity between Type I- and II-specific antibodies, these findings suggest structural and functional divergence between the two groups [212]. Also, a mixture of CfTX-1/2 and CfTX-A/B is at least six-fold more hemolytic than either purified toxins or crude venom, indicating synergistic interactions among JFTs [212]. In *C. rastoni*, CrTX-A and CrTX-B may be encoded by the same gene, with CrTX-B synthesized in the tentacle and subsequently modified into CrTX-A before allocation to the nematocyst [215]. Similarly, in *A. alata*, CaTX-A is primarily localized in nematocysts, whereas CaTX-B is only detected in the tentacles [213].

Phylogenetically, JFTs cluster into two major clades: JFT-1, with all known potent cubozoan toxins together with putative toxins from medically important scyphozoans and hydrozoans, and JFT-2, with mainly uncharacterized JFTs. Although JFTs are under purifying selection, several cubozoan JFT-1 toxins (containing CqTX-1, CfTX-A/B, and CfTX-1/2) display episodic positive selection. Extensive gene duplication within this clade, particularly in *Morbakka virulenta*, *Alatina alata* and *Chironex yamaguchii*, may contribute to the high toxicity of these species [216]. Expansion of the CfTX toxin family in the transcriptome and proteome of *Chironex fleckeri* could also be related to the high potency of cubozoan venoms [217].

**Table 8:** Representative toxins from the JFT family from Cnidaria.

Name	Type	Species	UniProt (PDB)	LD_50_ (μg/kg)	HC_50_ (ng/mL) (RBCs)	Ref.
CfTX-1	I	*Chironex fleckeri*	A7L035	-	161 (Sheep)	[13,35,212]
CfTX-2	I	*Chironex fleckeri*	A7L036	-	161 (Sheep)	[13,35,212]
CqTX-A	I	*Chiropsoides quadrigatus*	P58762	80 (Crayfish)	-	[218,219]
CaTX-A	II	*Alatina alata*	Q9GNN8	5–25 (Crayfish)	70 (Sheep)	[213]
CaTX-B	II	*Alatina alata*	Q9GNN8	-	80 (Sheep)	[213]
CfTX-A	II	*Chironex fleckeri*	T1PRE3	-	5 (Sheep)	[35,212]
CfTX-B	II	*Chironex fleckeri*	T1PQV6	-	5 (Sheep)	[35,212]
CrTX-A	II	*Carybdea rastonii*	Q9GV72	20 (Mice); 5 (Crayfish)	1.9 (Sheep)	[215]
CrTX-B	II	*Carybdea rastonii*	Q9GV72	-	2.2 (Sheep)	[215]
CcTX-1	-	*Cyanea capillata*	P0DUW6	-	-	[220]

HC_50_—concentration causing 50% hemolysis; LD_50_—median lethal dose; RBC—red blood cells.

#### 2.3.2. Actinoporins

Actinoporins are α-PFTs that specifically bind sphingomyelin (SM) in lipid membranes (Table 9), where SM functions both as a receptor and as a structural cofactor essential for pore assembly [221,222,223,224,225]. Most actinoporin genes lack introns within their coding regions, as reported for equinatoxins [226,227], RTX-S I [228] and magnificalysins [229,230]. However, Avt-I/Avt-II from *A. villosa* and PsTX-20A from *Phyllodiscus semoni* contain two introns and form a distinct clade separate from Actiniidae, Sagartiidae, and Stichodactylidae. Actinoporins form multigene families with highly homologous isoforms, as seen in equinatoxins [227,231] and fragaceatoxins [221] (5 isoforms); tenebrosins [232,233] (3 isoforms); Or-A and Or-G [234], Cjtox I and II [235] and sticholysins [236] (2 isoforms). Sticholysins differ in thermal stability and biological activity, likely contributing to ecological adaptations, with non-conserved substitutions in the N-terminal region influencing membrane insertion and hemolytic activity [236]. Notably, *R. magnifica* possesses more than 50 highly homologous actinoporin genes whose native and recombinant proteins differ in aa sequence and biological activity [230]. Actinoporins are likely multifunctional toxins involved in prey capture, defense, and digestion, due to their location in nematocysts, tentacles, actinopharynx and mesenteric filaments [235,237]. Phylogenetically, they are divided into two major clades: Clade 1, with few Actinioidea and Metridioidea sequences; and Clade 2, comprising predominantly Metridioidea (Clade 2M) and Actinioidea (Clade 2A) subclades. The absence of actinoporin sequences in Edwardsioidea but the presence of actinoporin-like sequences may reflect an ancestral loss of functional venom components, while retaining ancestral homologous [238].

Actinoporin-like toxins have also been identified in the hydrozoan *Hydra magnipapillata*: *Hydra* actinoporin-like toxins (HALTs). Compared with EqT II from *A. equina*, HALT-1 (A0A8B7DWS6) shows lower hemolytic activity, forms larger pores and has ~100-fold less affinity for SM [239]. Recombinant HALT homologs exhibit cytolytic activity against HeLa cells, with CC_50_ values ranging from 8.66 (rHALT-4) to 22.99 (rHALT-2) µg/mL. All six toxins bind sulfatide, whereas rHALT-1 and rHALT-3 additionally recognize lysophosphatidic acid and sphingosine-1-phosphate. Expression analyses revealed tissue-specific specialization, with HALT-1, 4, and 7 in differentiating stenoteles; HALT-1 and 6 in the female germline during oogenesis; and HALT-2 being highly expressed in gland and mucous cells of the endoderm [240].

**Table 9:** Representative toxins from the actinoporin family from Cnidaria.

Name	Species	UniProt	LD_50_ (μg/kg)	HC_50_ (ng/mL) (RBCs)	Ref.
Avt-I	*Actineria villosa*	Q5R231	-	58 (Sheep), 124 (Red sea bream), 241 (Human), 2669 (Mice)	[241,242]
Avt-II	*Actineria villosa*	D2YZQ3	-	-	[241]
Equinatoxin I’	*Actinia equina*	P0C1H0	23 (Mice)	-	[243]
Equinatoxin I’’	*Actinia equina*	P0C1H1	-	-	[231]
Equinatoxin II	*Actinia equina*	P61914	35 (Mice)	-	[222,243,244]
Equinatoxin III	*Actinia equina*	P0C1H2	83 (Mice)	-	[243]
Equinatoxin IV	*Actinia equina*	Q9Y1U9	-	-	[231]
Equinatoxin V	*Actinia equina*	Q93109	-	-	[227]
Fragaceatoxin A	*Actinia fragacea*	P0DUW8	-	0.4 nM (Sheep)	[221]
Fragaceatoxin B	*Actinia fragacea*	A0A515MEN7	-	0.3 nM (Sheep)	[221]
Fragaceatoxin C	*Actinia fragacea*	B9W5G6	-	1.6 nM (Sheep)	[221,223,245]
Fragaceatoxin D	*Actinia fragacea*	P0DUW9	-	0.4 nM (Sheep)	[221]
Fragaceatoxin E	*Actinia fragacea*	A0A515MEM9	-	1.6 nM (Sheep)	[221]
Tenebrosin A	*Actinia tenebrosa*	P30833	-	0.7 nM (Guinea pig)	[232]
Tenebrosin B	*Actinia tenebrosa*	P30834	-	2.2 nM (Guinea pig)	[232]
Tenebrosin C	*Actinia tenebrosa*	P61915	-	3.0 nM (Guinea pig)	[232,233]
Bandaporin	*Anthopleura asiatica*	C5NSL2	580 (Crayfish, LD_100_)	8.8 (Sheep)	[246]
Nigrelysin	*Anthopleura nigrescens*	A0A345GPN1	-	0.09 nM (Human)	[247]
Caissarolysin I	*Bunodosoma caissarum*	-	-	0.27 μg/mL (Human)	[248]
Cjtox I	*Cribrinopsis japonica*	A0A2Z5Z9X0	-	-	[235]
Cjtox II	*Cribrinopsis japonica*	A0A2Z5Z9H5		-	[235]
Src-I	*Cylista elegans*	Q86FQ0	-	430 (Human)	[249,250]
EnT	*Entacmaea quadricolor*	P0DMX3	-	-	[251]
Or-A	*Oulactis orientalis*	Q5I4B8	16,360 (Mice)	295.9 HU/mg (Mice)	[234]
Or-G	*Oulactis orientalis*	Q5I2B1	16,360 (Mice)	322.6 HU/mg (Mice)	[234]
PsTX-20A	*Phyllodiscus semoni*	P0DL55	50 (Shrimp)	80 (Sheep)	[252]
Pst-I	*Phyllodiscus semoni*	P0DL56	-	-	[241]
RTX-S II	*Radianthus crispa*	P0C1F8	70 (Mice)	3.6 × 10^4^ HU/mg (Rabbit)	[228,253]
RTX-A	*Radianthus crispa*	P58691	50 (Mice); 4.5 (Crabs)	3.5 × 10^4^ HU/mg (Rabbit)	[254,255]
RTX-S	*Radianthus crispa*	-	50 (Mice); 2.4 (Crabs)	5.0 × 10^4^ HU/mg (Rabbit)	[255]
RTX-G	*Radianthus crispa*	-	100 (Mice); 14.3 (Crabs)	1.0 × 10^4^ HU/mg (Rabbit)	[255]
HmT	*Radianthus magnifica*	P0DMX2	-	-	[251]
Magnificalysin I	*Radianthus magnifica*	P58689	140 (Mice)	3.6 × l0^4^ HU/mg (Rabbit)	[256]
Magnificalysin II	*Radianthus magnifica*	P58690	320 (Mice)	3.3 × 10^4^ HU/mg (Rabbit)	[256]
Magnificalysin III	*Radianthus magnifica*	Q9U6X1	-	0.33 (Human)	[229]
Gigantoxin IV	*Stichodactyla gigantea*	-	-	40 (Human)	[257]
Sticholysin I	*Stichodactyla helianthus*	P81662	-	21.7 (Human)	[236,258,259]
Sticholysin II	*Stichodactyla helianthus*	P07845	-	30 (Human)	[224,236,259,260]
SmT-1	*Stichodactyla mertensii*	P0DMX4	-	-	[251]

HC_50_—concentration causing 50% hemolysis; LD_50_—median lethal dose; RBC—red blood cells.

#### 2.3.3. Membrane-Attack Complex/Perforin (MACPF) Family

MACPF domains are common in mammalian immune systems, where MAC and PF proteins released by cytotoxic T-cells and natural killer cells form pores in target cell membranes. However, MACPF domains are widespread across eukaryotes and also occur in pathogenic bacteria *Chlamydiae*, likely via horizontal gene transfer [261]. AvTX-60A (Q76DT2) from *A. villosa* shares high similarity with PsTX-60A (P58911) from *P. semoni*, representing the first MACPF-domain toxins isolated from cnidarian nematocysts, potentially used for prey capture or predator deterrence [261,262,263]. AvTX-60A is highly toxic to mice (minimum lethal dose < 250 μg/kg, i.p.) [261], whereas PsTX-60A and PsTX-60B (P58912) are lethal to shrimp (LD_50_: 800–900 and 800 μg/kg, respectively) and hemolytic toward sheep erythrocytes (HC_50_: 600 and 300 ng/mL, respectively) [262,263].

#### 2.3.4. Hydralysins

Hydralysins are β-PFTs from *Hydra*, forming a distinct cnidarian toxin family more closely related to bacterial and fungal PFTs than to other cnidarian cytolysins [264,265]. The first characterized member, Hydralysin-1 (Hln-1, Q86LR2) from *H. viridissima*, is a non-nematocyst toxin that induces paralysis in blowfly larvae (PD_50_: 22–36 ng per 100 mg body weight), while exhibiting low hemolytic potency (50% hemolysis at 93 µg/mL) [265]. *H. viridissima* expresses three hydralysin isoforms that differ in their N-terminal regions and bioactivities. rHln-2 is ~5-fold more potent than rHln-1 (PD_50_: 10.3 vs. 55.7 ng per 100 mg body weight), whereas rHln-3 lacks detectable hemolytic or paralytic activity. Nevertheless, all three isoforms bind erythrocyte membranes and form pores (~1.2 nm diameter), indicating a conserved pore-forming mechanism. Hemolytic activity of the active isoforms is unaffected by preincubation with cholesterol, SM, or phosphatidylcholine, indicating these lipids are not their primary receptors. Homologues have also been detected in *H. vulgaris* and *H. magnipapillata*, suggesting that hydralysins represent an evolutionarily conserved pore-forming system within *Hydra* [264].

### 2.4. Other Toxins

Other cnidarian toxins with diverse biological activities are listed in Table 10. Acrorhagin I and II, isolated from the acrorhagi of *A. equina*, are lethal to crabs. Homologous variants were identified by RT-PCR, where acrorhagin Ia shares 65% sequence identity with acrorhagin I, and acrorhagin IIa differ from acrorhagin II by a single substitution (Ala31Val) [266,267]. Gigantoxin-I from *S. gigantea* is the first epidermal growth factor (EGF)-like toxin described in Cnidaria [84]. Its localization in nematocysts and toxicity toward crabs suggest that it may represent an ancestral EGF with a venom-related function, later lost in other animal lineages [85]. Homologous EGF-like toxins have also been identified in *A. viridis* [120] and *S. haddoni*, where SHTX-V was detected in nematocysts by RT-PCR [85]. Hau I from *H. aurora* represents a novel toxin class with the same Cys pattern (CXXC-C-C) as the BBH (Type IV KTx) family [82], and transcriptomic analysis identified homologous peptides (HC-18 and HC-19) in *R. crispa* [268]. Hau I is expressed in the column and mesenteric filaments, but not in tentacles, suggesting roles in defense and internal prey immobilization rather than prey capture [82,268]. PhcrTx1 from *Phymanthus crucifer* is the first sea anemone neurotoxin shown to adopt an inhibitor cystine knot (ICK) scaffold, a structural motif common in spider, scorpion, and cone snail venoms [269].

## 3. Three-Dimensional Toxin Structures

Structural biology techniques, including X-ray crystallography, cryo-electron microscopy (cryo-EM) and nuclear magnetic resonance (NMR) spectroscopy, provide atomic-level insights into the three-dimensional structure of peptides [310]. Experimentally resolved structures are available for 37/418 (8.9%) cnidarian toxins (Tables S2 and S3). By revealing conserved folds, catalytic residues, disulfide connectivity, and putative structure–function relationships, structural analysis complements omics-based toxin discovery. Recent advances in protein structure prediction, particularly AlphaFold [311], PepFold4 [312] and MODELLER [313], have enabled structural elucidation of toxins lacking experimentally resolved structures. Examples include the structural characterization of neurotoxins from *Palythoa variabilis* following transcriptomic analysis [296]; structural modeling, molecular dynamics and docking analysis of ZoaKuz1 from *Zoanthus natalensis* with a modeled K_V_1.1 following proteotranscriptomic analysis [309]; molecular docking of the of the AlphaFold predicted structure of the toxin α-KTx 1.13 from *Catostylus* sp. with the K_v_3.1 channel crystal structure following nanoLC-MS/MS identification [277]; novel toxin structure determined by NMR spectroscopy from the nematocyst proteome of *H. actiniformis* [210,289]; and the identification of a potential Ca_v_3.1 channel regulator in *A. equina* through proteotranscriptomic analysis, machine learning and molecular docking [270]. The following sections summarize the current structural knowledge of the major cnidarian toxin families discussed in this review.

**Figure 4:**
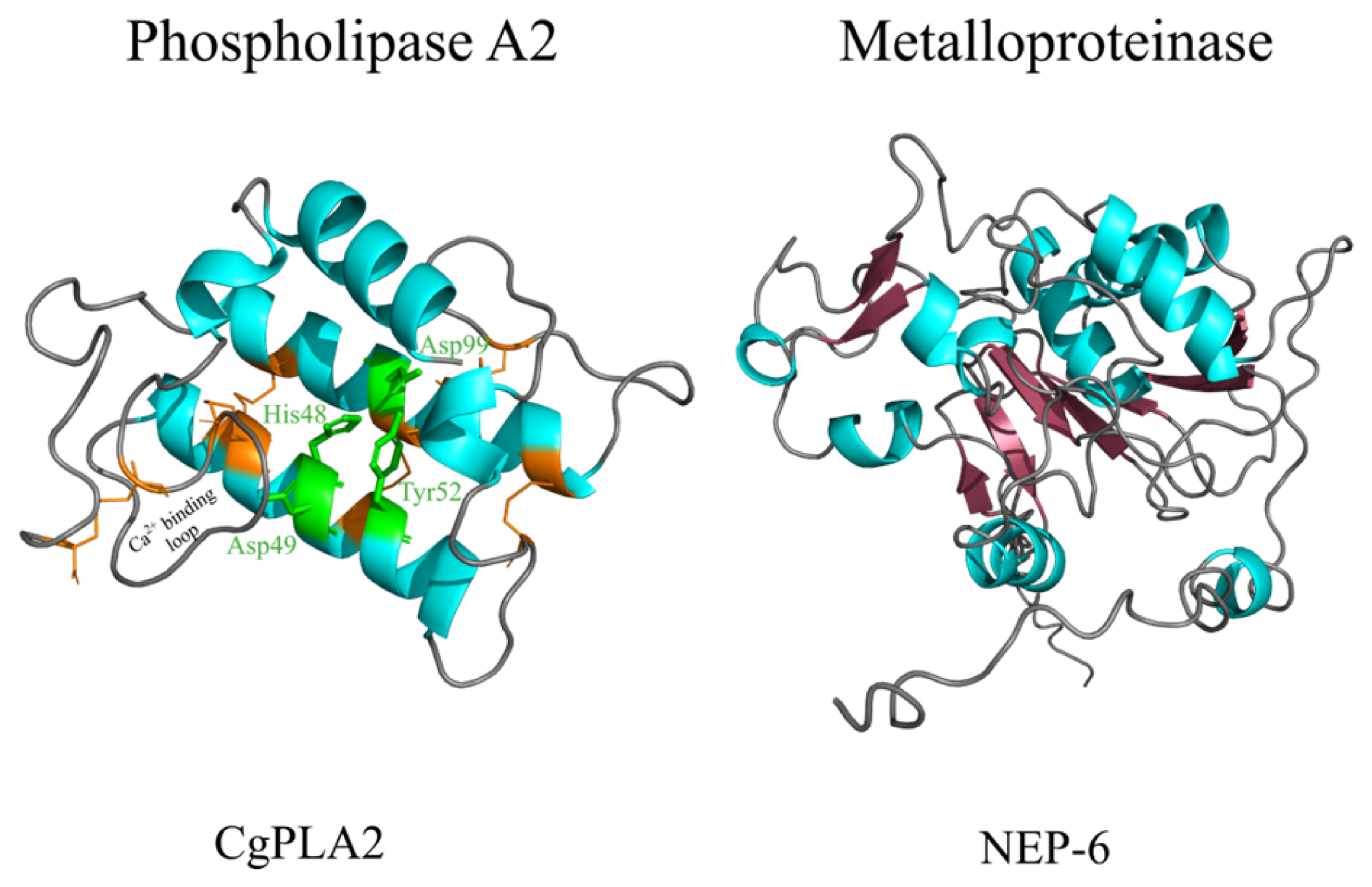
Structural overview of toxic enzymes. Phospholipase A2 is represented by CgPLA2 (AlphaFold: AF-D2X8K2-F1-v6) from *Condylactis gigantea*; and metalloproteinase by NEP6 (AlphaFold: AF-K7Z9Q9-F1-v6) from *Nematostella vectensis*. Colors indicate α-helices (blue), β-sheets (red), the catalytic center (green), and cysteine residues (orange).

Cnidarian PLA_2_s are represented by the conserved catalytic center (His48-Asp49 dyad, Tyr 52 and Asp 99), the Ca^2+^ binding loop, and twelve conserved Cys residues likely forming six disulfide bonds, all characteristic features of sPLA_2_s. Unlike group I and group II/X sPLA_2_s, cnidarian PLA_2_s lack the two additional Cys and the C-terminal extension, respectively [21,22]. Moreover, the astacin-like metalloproteinase NEP-6 illustrates the characteristic mixed α/β architecture of astacin metalloproteinases [41] (Figure 4).

**Figure 5:**
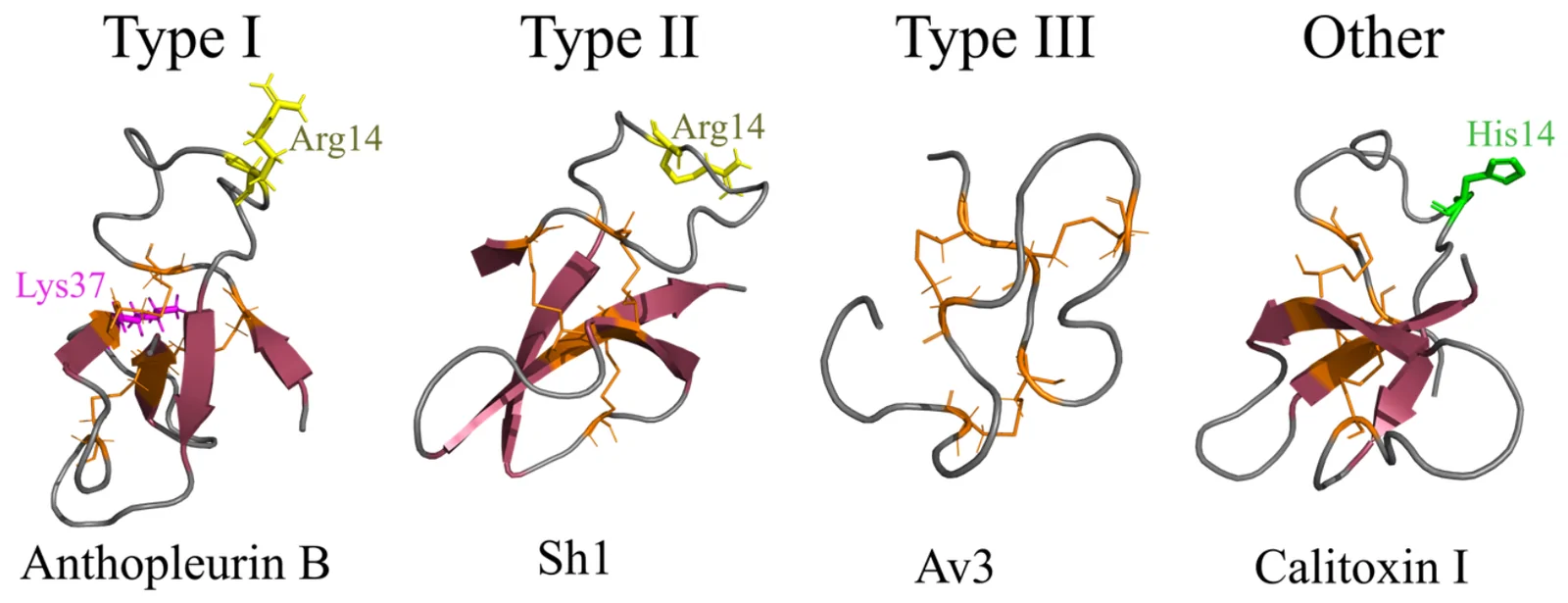
Structural overview of NaTx. Type I is represented by Anthopleurin B (PDB:1APF) from *Anemonia sulcata*; Type II by Sh1 (PDB:1SH1) from *Stichodactyla helianthus*; Type III by Av3 (PDB:1ANS) from *A. sulcata*; and Calitoxin I from *Calliactis parasitica* (predicted by ColabFold/AlphaFold; pLDDT: 82.8, pTM: 0.593, File S2). Colors indicate α-helices (blue), β-sheets (red), the Arg14 loop (yellow), Lys37 (pink), His14 (green), and cysteine residues (orange).

Type I and II NaTx share six conserved Cys residues, a flexible Arg14 loop involved in propeptide processing, four stranded anti-parallel β-sheets connected by three loops, and a basic C-terminal region [69,99]. In Anthopleurin B, Lys37 contributes to Na_v_1.5 binding [314]. In contrast, the Type III NaTx Av3 adopts a compact fold stabilized by three disulfide bonds and features four reverse turns (a distorted type I β-turn, a type I β -turn, and inverse γ-turns) and two other chain reversals, but no regular α-helix or β-sheet [107]. Its bioactive surface is mainly composed of aromatic residues and interacts differently with the site-3 of Na_v_ channels, as a D1701R mutation in DmNa_v_1 abolishes the activity of other site-3 ligands but not Av3 [43]. Calitoxin I also differs structurally due to the Arg14His mutation and a distinct C-terminal motif from Type II NaTx [105] (Figure 5).

**Figure 6:**
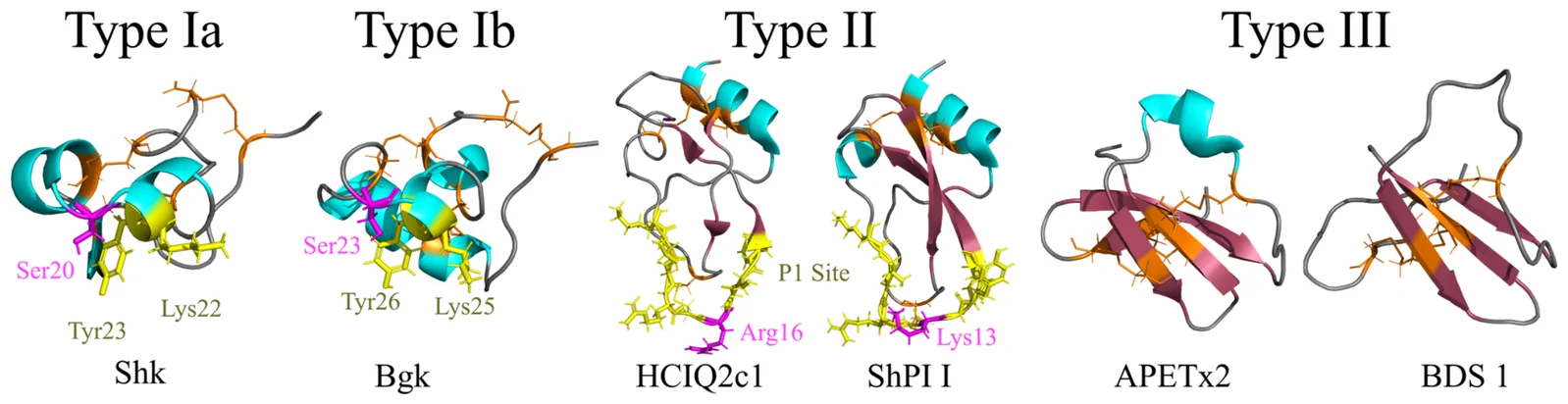
Structural overview of KTx. Type Ia is represented by ShK (PDB: 1ROO) from *Stichodactyla helianthus* and Type Ib by BgK (PDB:1BGK) from *Bunodosoma granuliferum*; Type II by HCIQ2c1 (PDB:9IJW) from *Radianthus crispa* and ShPI I (PDB:1SHP) from *S. helianthus*; and Type III by APETx2 (PDB:1WXN) from *Anthopleura elegantissima* and BDS 1 (PDB:1BDS) from *Anemonia sulcata*. Colors indicate α-helices (blue), β-sheets (red), the Lys-Tyr dyad and Ser residues (yellow and pink), the P1 site (yellow) with Arg16 and Lys13 residues (pink), and cysteine residues (orange).

Type I KTxs are short peptides composed of three α-helices stabilized by three disulfide bonds. Conserved residues, particularly the Lys-Tyr dyad and Ser, are essential for K_v_ channel binding [125,132]. Type II KTx are structurally related to dendrotoxins [315] and consist of long polypeptides containing one to two α-helices and two to four stranded β-sheet stabilized by three disulfide bonds [163,166]. Their canonical binding loop contains the reactive P1 site, typically Lys or Arg, which mediates ~50% of the interaction with the S1 binding pocket of serine proteases, being critical for protease inhibition [118,145,164]. Type III KTxs are stabilized by three disulfide bonds and adopt a triple-stranded antiparallel β-sheet fold. In contrast, APETx2 additionally contains a short α-helice [169,171] (Figure 6).

**Figure 7:**
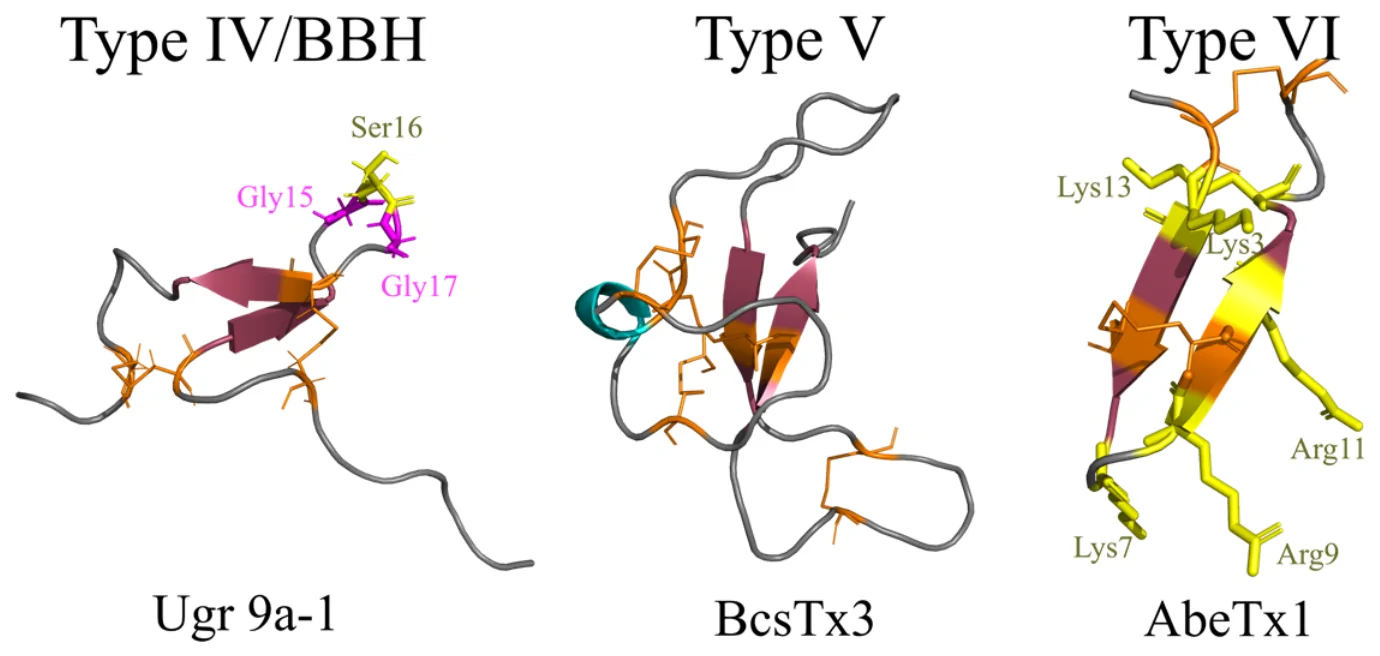
Structural overview of KTx. Type IV is represented by Ugr 9a-1 (PDB: 2LZO) from *Urticina grebelnyi*; Type V by BcsTx3 (AlphaFold: AF-C0HJC4-F1) from *Bunodosoma caissarum*; and Type VI by AbeTx1 (AlphaFold: AF-C0HJD9-F1) from *Actinia bermudensis*. Colors indicate α-helices (blue), β-sheets (red), the Ser/Gly-rich β-turn (yellow and pink), the Lys and Arg residues (yellow), and cysteine residues (orange).

Type IV KTx are stabilized by two disulfide bonds and are characterized by the conserved residues Pro6, Tyr11, Asp14, Asn18, Val20, and Gly27. They adopt the BBH fold, in which the β-hairpin is primarily stabilized by backbone interactions rather than disulfide bonds, with a central Ser/Gly-rich β-turn (residues 15–17) that is essential for proper folding [82,195,196]. The AlphaFold-predicted structure of Type V KTx indicates that it is stabilized by four disulfide bonds [197], whereas the Type VI KTx structure has two β-sheets and two disulfide bonds, and is enriched in Lys and Arg residues that may interact with K_v_1.1 and K_v_1.6, lacking aromatic and aliphatic residues [198] (Figure 7).

**Figure 8:**
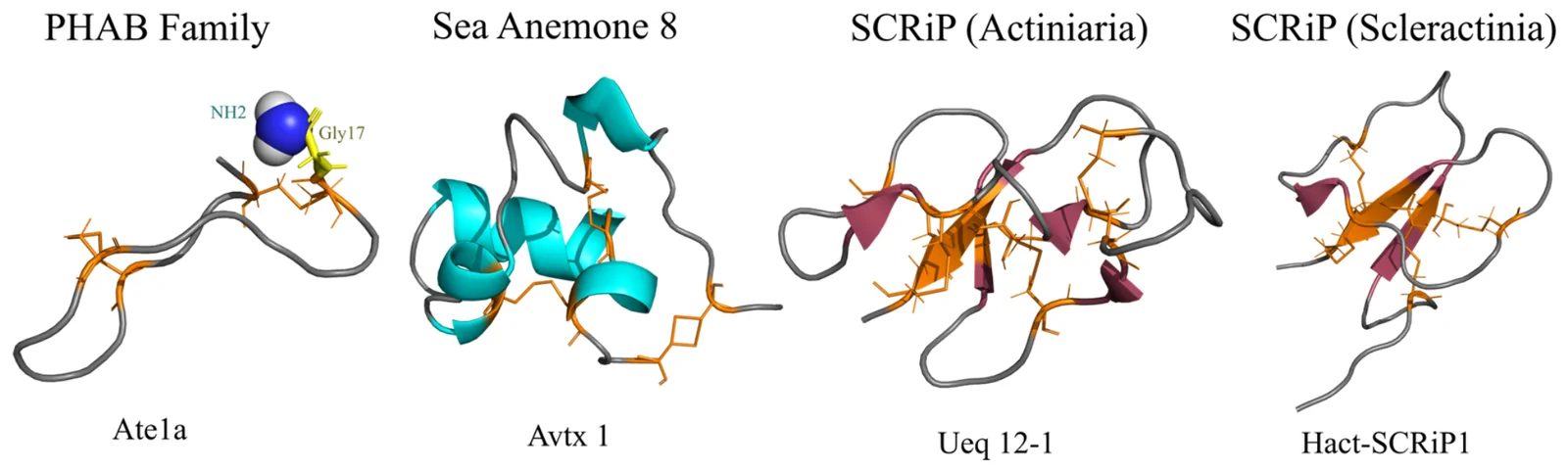
Structural overview of other neurotoxins. The PHAB family is represented by Ate1a (PDB: 6AZA) from *Actinia tenebrosa*; the SA8 family by Avtx1 (predicted by ColabFold/AlphaFold2 with pLDDT: 86.8 pTM: 0.585, File S2); and SCRiPs are represented by Ueq 12-1 (PDB: 5LAH) from *Urticina eques* and by Hact_SCRiP1 (PDB: 7LX4) from *Heliofungia actiniformis*. Colors indicate α-helices (blue), β-sheets (red), the C-terminal Gly17 (yellow), and cysteine residues (orange).

Ate1a from *A. tenebrosa* adopts the PHAB fold, stabilized by two disulfide bonds and a C-terminal glycine amidation [201]. Although the SA8 family shares superficial sequence similarity with ShK toxins, its structure reveals marked differences in overall fold and disulfide connectivity [202]. Among SCRiPs, Ueq 12-1 from *Urticina eques* adopts a W-shaped fold stabilized by five disulfide bridges comprising a three-strand antiparallel β-sheet, a small two-strand parallel β-sheet, and one turn of a 3_10_ helix [211], whereas Hact-SCRiP1 from *Heliofungia actiniformis* adopts a β-defensin-like fold with four disulfide bridges [210] (Figure 8).

**Figure 9:**
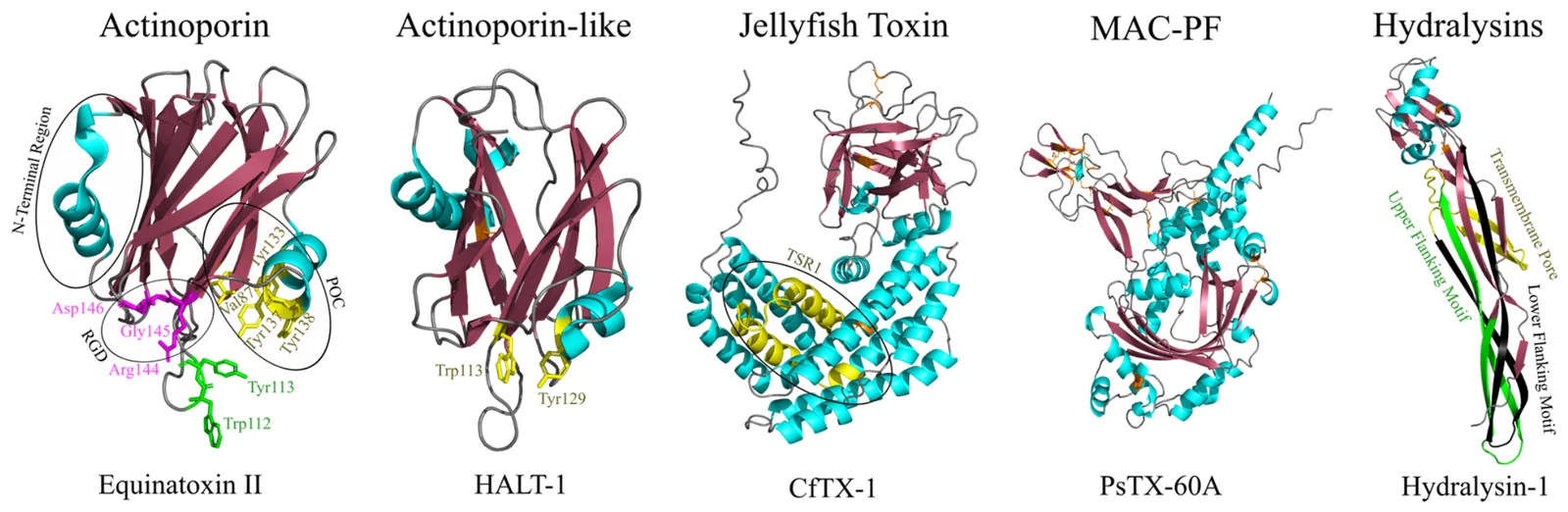
Structural overview of PFTs from Cnidaria. Actinoporins are represented by Equinatoxin II (PDB: 1IAZ) from *Actinia equina*; JFTs by CfTX-1 (AlphaFold: AF-A7L035-F1) from *Chironex fleckeri*; actinoporin-like by HALT-1 (PDB: 7EKZ) from *Hydra magnipapillata*; MACPF by PsTX-60A (AlphaFold: AF-P58911-F1) from *Phyllodiscus semoni*; and hydralysins by Hydralysin-1 (AlphaFold: AF-Q86LR2-F1) from *Hydra viridissima*. Colors indicate α-helices (blue), β-sheets (red), the POC-binding site (yellow), the aromatic-rich region (green), the RDG motif (pink), Trp113-Tyr129 (yellow), the TSR1 region (yellow), hydralysin conserved motifs (yellow, green and black), and cysteine residues (orange).

Actinoporins adopt a β-sandwich core flanked by two α-helices. Pore formation is initiated by specific recognition of SM through an aromatic-rich region, particularly Trp112 and Tyr113, together with the phosphocholine (POC)-binding site. Membrane binding triggers conformational changes that reposition the N-terminal amphipathic α-helix to the lipid–water interface, promoting oligomerization and insertion into the membrane to form a toroidal pore [316,317,318]. Some actinoporins contain an RGD motif that mediates binding to integrin receptors on the cell surface [319]. This motif is absent in Avt-I and PsTX-20 [241], and is replaced by GCD in Or-A and Or-G and by EGD in SrcI [253]. Actinoporins generally lack Cys residues [232,247,251], except for bandaporin, which contains Cys194 [246]. In the actinoporin-like HALT-1 from *Hydra*, Trp113 and Tyr129 are essential for sulfatide, lysophosphatidic acid and sphingosine-1-phosphate binding [240]. JFTs consist of a mixture of α-helices, β-sheets and loop structures. Their N-terminal α-helical region contains a putative transmembrane spanning region, TSR1, which is highly conserved in CfTXs, CqTX-A, CrTXs and CaTX-A, and may be important for pore formation [13,213,219]. Moreover, MACPF toxins display the characteristic mixed α/β architecture with 15 Cys residues [262], and Hydralysins are rich in β-structures and share a set of highly conserved sequence motifs with other β-PFTs (aerolysin, epsilon toxin and LSL), located in and around the pore-forming domains of the toxins [264] (Figure 9).

**Figure 10:**
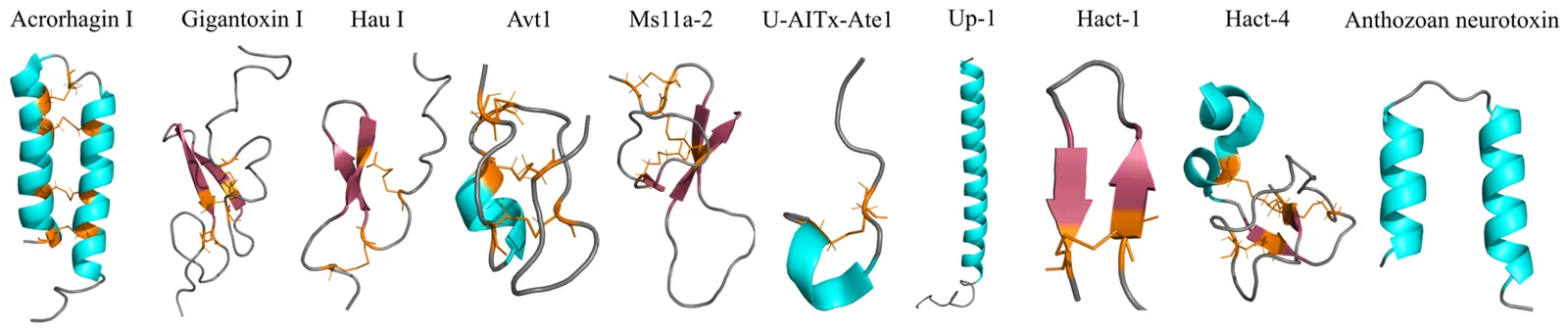
Structural overview of other cnidarian toxins: Acrorhagin I (PDB: 6UX5) from *Actinia equina*; EGF-like Gigantoxin-I (AlphaFold: AF-Q76CA1-F1) from *Stichodactyla gigantea*; Hau I from *Heteractis aurora* (AlphaFold: AF-A0A0P0UTJ1-F1); Avt1 (PDB: 9CRB) from *Aulactinia veratra*; Ms11a-2 (PDB: 6XYH) from *Metridium senile*; U-AITx-Ate1 (PDB: 6OQP) from *Actinia tenebrosa*; Up-1 (AlphaFold: AF-P0C1G1-F1-v6) from *Urticina piscivora*; Hact-1 (AlphaFold: AF-P0DUS1-F1) and Hact-4 (PDB: 7MJ3) from *Heliofungia actiniformis*; and Anthozoan neurotoxin from *Palythoa caribaeorum* (predicted by ColabFold/AlphaFold; pLDDT: 63.2, pTM: 0.286, File S2). Colors indicate α-helices (blue), β-sheets (red), and cysteine residues (orange).

Regarding other toxins, Acrorhagin I adopts a helical hairpin fold stabilized by four disulfide bonds and resembles the B-IV neurotoxin from the worm *C. lacteus* [267]; Gigantoxin-I contains six conserved Cys residues and share 31–33% sequence homology with mammalian EGFs [84]; and Hau I displays the characteristic BBH fold [82]. Avt1 adopts an ICK scaffold, in which a ring is formed by two disulfide bonds with a third disulfide penetrating the ring to create the pseudo-knot, providing high structural, thermal and proteolytic stability [269]. However, new ICK variants, detected in *M. senile*, feature a prolonged loop between the fifth and sixth Cys residues, with three-strand antiparallel β-sheet, as seen in Ms11a-2 [290]. U-AITX-Ate1 adopts an extended loop conformation lacking α-helices and β-sheets with a single disulfide bond [271]. In the coral *H. actiniformis*, Hact-1 forms a β-hairpin structure stabilized by a single disulfide bond, whereas Hact-4 adopts a β-defensin-like fold [210,289]. Finally, the Anthozoan neurotoxin from *P. caribaeorum*, also identified in the *P. variabilis* transcriptome, has an unusual V-shaped α-helical fold (Figure 10) [295,296].

## 4. Biomedical and Biotechnological Potential

Peptides offer some advantages compared to traditional small molecule drugs, including high target specifificy, binding affinity, and potency, resulting in fewer off-target side effects [310,320,321]. More than 200 peptide-based drugs have been approved by the U.S. Food and Drug Administration (FDA) [320]. Hence, venom-derived peptides serve as potential scaffolds for drug discovery, although their intrinsic toxicity prevents direct therapeutic use. To enhace their safety while preserving biological activity, peptide engineering and dose optimizations are needed [321,322]. Also, venom-derived peptides face rapid proteolytic degradation, poor oral bioavailability, short serum half-life, low plasma stability and immunogenicity. Chemical and structural modifications, including aa substitutions, cyclization and PEGylation, together with drug delivery systems such as micelles and lipid nanoparticles, can improve peptide stability and pharmacokinetic properties [310,320,321]. Moreover, the low abundance of toxins in natural venoms and their disulfide-rich structure require the heterologous recombinant expression of toxin genes in bacteria and yeast, allowing their production in the large scale with structural fidelity to test their terapeutical potential, avoiding also contamination [310,323]. Finally, the integration of transcriptomics and proteomics facilitate the identification of novel peptides with therapeutical potential in complexe venom mixtures [322], as seen in sea anemones [96], stony corals [284,302] and zoanthids [309].

**Figure 11:**
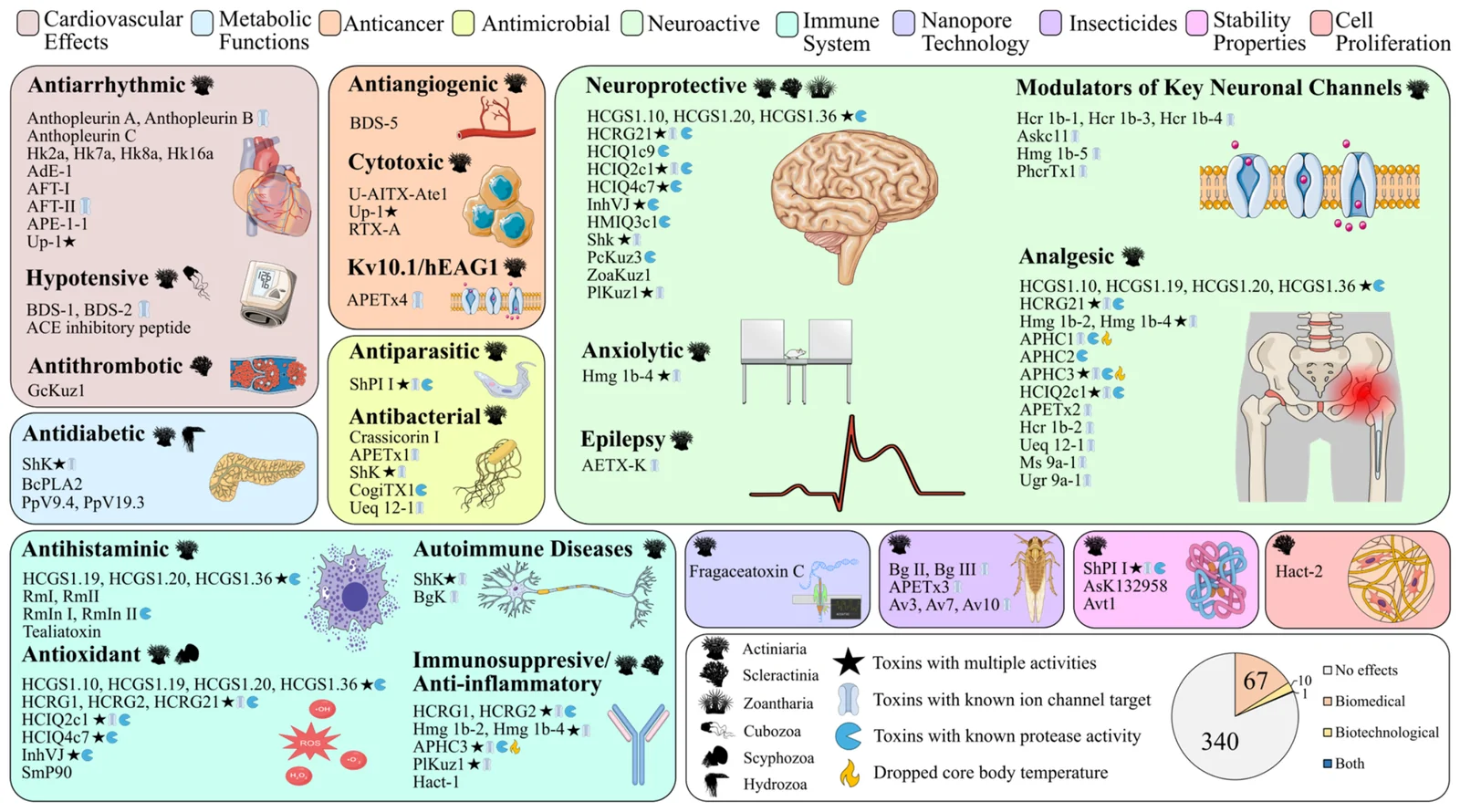
Biomedical and biotechnological potential of Cnidaria toxins, grouped by category. The pie chart represents the number of toxins that have biomedical and biotechnological potential, and those that were not tested for their bioactive potential.

Among the studies included in this review, 78 cnidarian toxins have potential for biomedical and biotechnological applications (Figure 11). This is promising, as several venom-derived peptides from snakes, cone snails, and Gila monster have been approved by the U.S FDA for clinical use, while others remain in preclinical or clinical development. The synthetic Shk analogue dalazatide (ShK-186), derived from the sea anemone *S. helianthus*, has advanced to clinical trials for the treatment of autoimmune diseases [310].

### 4.1. Cardiovascular Effects

Antiarrhythmic: Cnidarian cardiotoxins exhibit positive inotropic properties, highlighting their potential as antiarrhythmic agents [51,61,62,63]. For example, Anthopleurins A and B from *A. elegantissima* show higher affinity for the cardiac Na_v_1.5 isoform [66], and Hk2a, Hk7a, Hk8a and Hk16a from *Anthopleura* sp. stimulate rat atrial tissues, with Hk2a eliciting the strongest response (1038% increase) and Hk7a the longest-lasting effect (240 min). The residue Arg14 in Hk7a may influence the duration of its effects [50]. Moreover, AdE-1 from *E. diaphana* decreased action potential amplitude and time-to-peak without inducing early or delayed after-depolarizations, spontaneous twitches, or irregular rhythm in cardiomyocytes [106,111], and Up-1 from *U. piscivora* increased the contractile force of rat and guinea pig left atria with little or no increase in heart rate [308], although its high toxicity may require structural optimization.

Hypotensive: BDS-1 and BDS-2 from *A. sulcata* were reported as having hypotensive properties, although the mechanism of action is unknown [171]. Both peptides selectively modulate neuronal ion channels, with no activity on cardiac or skeletal muscle Na_v_s [170,179,180]. In contrast, a peptide from *C. quadrigatus* inhibits the angiotensin-converting enzyme with no rat toxicity, promoting vasodilation and preventing bradykinin degradation, thereby lowering blood pressure [278].

Antithrombotic: GcKuz1 from *G. columna* shows potent anticoagulant and antithrombotic activity by inhibiting plasma kallikrein and FXIIa, key enzymes of the contact–kinin pathway. It prolongs clotting times in vitro, reduces arterial and tail thrombosis in mice, and protects brain tissue in ischemic stroke models [284].

### 4.2. Anticancer

Antiangiogenic: A fraction containing BDS-5 from *A. sulcata* inhibits proliferation and angiogenesis of human microvascular endothelial cells (IC_50_: 14 nM), showing potential anti-tumor angiogenic activity. The active principle could be a Kunitz-type inhibitor, which may interact with an integrin due to a solvent exposed RGD motif [181].

Cytotoxic: U-AITX-Ate1 from *A. tenebrosa* showed cytotoxicity against the breast cancer cell line MCF7 (IC_50_: 548 μM) and MDA-MB-231 (IC_50_: 124 μM) [271], and Up-1 from *U. piscivora* to the tumor cell lines KB, L1210, and HEL299 (TC_50_: 40.32, 29.99, and 29.74 μg/mL, respectively) [307]. The actinoporin RTX-A from *R. crispa* is also cytotoxic against several cancer cell lines (IC_50_: 0.57–30 nM). Notably, it inhibits the malignant transformation of mouse JB6 P^+^ Cl41 and human HeLa cells at much lower concentrations (INCC_50_: 0.034 nM), possibly through inhibition of the AP-1 and NF-κB signaling pathways and induction of p53-independent apoptosis [324].

K_v_10.1/hEAG1: APETx4 from *A. elegantissima* potently inhibits the oncogenic K_v_10.1/hEAG1, which is overexpressed in many human tumors. Induces concentration-dependent cytotoxicity in cancer cells while sparing healthy fibroblasts, and does not inhibit the cardiac K_v_11.1/hERG channel, avoiding arrhythmogenic side effects [175].

### 4.3. Antimicrobial

Antibacterial: Crassicorin I from *U. crassicornis* has antibacterial activity against Gram^+^ (*B. subtilis*, MIC: 11.49 μg/mL) and Gram^−^ bacteria (*E. coli*, MIC: 12.21 μg/mL; *S. enterica*, MIC: 11.95 μg/mL). In radial diffusion assays, APETx1 from *A. elegantissima* was also active against *S. enterica*, while ShK from *S. helianthus* inhibited *B. subtilis*, *S. aureus*, *S. enterica* and *P. aeruginosa* [194]. Ueq 12-1 from *U. eques* showed antibacterial activity against *C. glutamicum* (MIC: 50 uM) and, to a lesser extent, *S. aureus* [211]. In contrast, CogiTx1 from *C. gigantea* inhibits the *B. licheniformis* subtilisin A (IC_50_: 5.3 × 10^7^ mol/L) and recombinant *S. maltophilia* protease 1 (1478 U/mg) [192].

Antiparasitic: ShPI I from *S. helianthus* inhibits serine proteases and reduces the viability of *Leishmania amazonensis*, including ultrastructural alterations consistent with parasite damage [325]. Similarly, in *Trypanosoma cruzi*, ShPI I reduces parasite viability in a dose- and time-dependent manner. Complete parasite death occurred at 1 × 10^−5^ M after 24–48 h, whereas 1 × 10^−6^ M reduced viability by 91–95% [326].

### 4.4. Immune System

Autoimmune Diseases: The synthetic peptide ShK-186 (selective K_v_1.3 inhibitor), derived from *S. helianthus*, is well tolerated in patients with moderate psoriasis, reducing disease severity by inhibiting chronically activated memory T-cells and inflammatory mediators [327]. It also inhibits IL-2 production and is effective in experimental autoimmune encephalomyelitis (EAE) and delayed-type hypersensitivity models [328]. Similarly, the synthetic peptide BgK-F6A (selective Kv1.1 blocker) from *B. granuliferum* also alleviates neurological deficits and brain damage in EAE by preserving neuronal function, preventing astrocytic edema, and maintaining brain energy metabolism, showing potential to treat neuroinflammatory disorders (multiple sclerosis, stroke, and trauma) [329].

Immunosuppressive/Anti-inflammatory: HCRG1 and HCRG2 from *R. crispa* reduce pro-inflammatory TNF-α and IL-6 secretions, as well as proIL-1β expression in LPS-activated macrophages [148]. In vivo, HCRG1 reduced carrageenan-induced paw edema in mice (0.1–1 mg/kg), comparable to the drug indomethacin (5 mg/kg) [147]. Similarly, Hmg 1b-4 from *R. magnifica* exhibited stronger anti-inflammatory activity than Hmg 1b-2 and outperformed diclofenac in acute inflammation models [191]. In rat models of osteoarthritis and rheumatoid arthritis, APHC3 from *R. crispa* reduced joint swelling, improved mobility and grip strength, decreased pain sensitivity, lowered IL-1β levels in synovial fluid and prevented cartilage damage, performing as well as diclofenac, ibuprofen, and meloxicam [330]. PlKuz1 from *Porites lutea* also reduced LPS-induced pro-inflammatory cytokine production and neutrophil migration, likely through plasma kallikrein and Kv1.3 inhibition [302]. Finally, HACT-1 from *H. actiniformis* shows weak but detectable immunomodulatory activity by reducing LPS-induced TNFα release without cytotoxicity [289].

Antihistaminic: The RmI/RmII mixture from *R. crispa* delayed the onset and shortened the duration of histamine-induced responses in a dose-dependent manner [255]. Similarly, RmIn I and RmIn II reduced histamine-induced inflammation in rats [162]. HCGS1.20, HCGS1.19 and HCGS1.36 also showed strong antihistaminic and anti-inflammatory activity, comparable to fexofenadine, by blocking histamine-induced Ca^2+^ increase in murine macrophages. Their effects likely involve H_1_-histamine receptor inhibition or interference with inflammatory proteases [331]. Moreover, tealiatoxin from *Urticina felina* blocks histamine-induced contractions of the guinea-pig ileum [306].

Antioxidant: Toxins from *R. crispa* (HCGS1.10, HCGS1.19, HCGS1.20, HCGS1.36, HCRG1, HCRG2, HCRG21 and InhVJ) reduced ROS levels, with HCRG21 showing the strongest effect. HCRG2 and HCRG21 exhibited the highest antiradical effect, scavenging 14.5% of DPPH radicals; and InhVJ showed a concentration-dependent antioxidant effect, peaking at 1–10 µM [156]. At 10 μM, HCIQ2C1 and HCIQ4C7 increased neuroblastoma cell viability by 47% and 17%, respectively, in a 6-OHDA neurotoxic model, likely through ROS reduction [151]. Finally, the antioxidant protein SmP90 from the scyphozoan *S. meleagris* strongly scavenges superoxide anion radicals (EC_50_: 16 ug/mL) [304].

### 4.5. Neuroactive

Neuroprotective: In Neuro-2a cells, toxins from *R. crispa* increased cell viability following 6-OHDA-induced damage, including HCGS1.10 (31.7%), HCGS1.20 (20.9%), HCGS1.36 (22.8%), HCRG21 (23.2%) and InhVJ (38%) [156]. Similarly, HMIQ3c1 from *R. magnifica* increased Neuro-2a cell viability by 39.4% in the presence of β-amyloid (Alzheimer’s model) [153]. HCIQ1c9, HCIQ2c1, HCIQ4c7, and HMIQ3c1 also reduced ROS production and inhibited ATP-activated P2X7 receptors, protecting neuronal cells from Parkinson’s disease inducers. Likewise, the anthozoans Kunitz-like peptides PcKuz3, ZoaKuz1 and PlKuz1 protected dopaminergic neurons and improved locomotor deficits in zebrafish models of Parkinson’s disease (at 2.5–5, 0.2, 0.3–3 μM, respectively) [167,302,309]. Finally, the synthetic peptide ShK-170 from *S. helianthus* promoted neurorestoration by blocking Kv1.3 channels in activated microglia, reducing pro-inflammatory mediators and stimulating neural progenitor cell proliferation [332].

Analgesic: Several cnidarian toxins exert analgesic effects through modulation of TRPV1, TRPA1 and ASIC channels. Hmg 1b-2 and Hmg 1b-4 from *R. magnifica* showed analgesic effects comparable to diclofenac in an acid-induced muscle pain model [191]. Similarly, APHC1, APHC2 and APHC3 from *R. crispa* also produced analgesia in vivo [159,160]. Unlike conventional TRPV1 antagonists, APHC1 and APHC3 relieved pain without inducing hyperthermia and even caused mild hypothermia. APHC3 was particularly effective under acidic conditions, mimicking inflamed tissues, and more effective than APHC1 in the acetic acid writhing teste [161]. Both peptides also reduced TRPV1-dependent contractions in normal and diabetic bladder smooth muscle by inhibition of TRPV1-mediated tachykinin release [333]. Although HCRG21 from *R. crispa* is a weaker TRPV1 inhibitor than APHC1, it showed comparable analgesic activity with a longer duration of action (13h vs. 3h; 0.1 mg/Kg) [149,334]. TRPA1 modulators include Ms 9a-1 from *M. senile* and Ueq 12-1 from *U. eques*, which desensitize TRPA1-expressing neurons through repeated agonist-like activity, reducing pain signaling. Both peptides alleviated AITC-induced pain, inflammation, and CFA-induced hyperalgesia in vivo [195,211]. HCIQ2c1 also reduced AITC- and capsaicin-induced pain and inflammation [163]. Moreover, APETx2 from *A. elegantissima* potently inhibits ASIC3, abolishes acid-induced pain in rats, and is in preclinical development for chronic inflammatory pain [335,336]. Likewise, Hcr 1b-2 from *R. crispa* and Ugr 9-a from *U. grebelnyi* inhibit ASIC channels and reduced writhing responses in vivo. Ugr 9a-1 also alleviates CFA-induced thermal hyperalgesia without causing toxicity or motor impairment [188,196]. Finally, HCGS1.10, HCGS1.19, HCGS1.20 and HCGS1.36 from *R. crispa* also caused analgesic activity in the mouse tail-flick test, with HCGS1.20 and HCGS1.36 being the most effective [337].

Anxiolytic: Hmg 1b-4 from *R. magnifica* causes an anxiolytic effect on mouse behavior in the open field and elevated plus maze tests, whereas Hmg 1b-2 causes an excitatory effect [191].

Epilepsy: AETX-K from *A. erythraea* (high affinity K_v_1.1 modulator) restored currents of the epilepsy-associated K_v_1.1-L296F mutant channel to wild-type levels at 4 pM [122].

Modulators of Key Neuronal Channels: Hcr 1b-1, Hcr 1b-3 and Hcr 1b-4 from *R. crispa* and Hmg 1b-5 from *R. magnifica* primarily target ASIC channels, with Hmg 1b-5 also binding nAChR [177,188]. PhcrTx1 from *P. crucifer* also partially inhibits ASIC currents, with much lower activity against K_v_ channels [298], whereas AsKC11 from *A. sulcata* selectively activates neuronal GIRK1/2 channels [154]. Together, these toxins represent promising candidates for future neuropharmacological studies.

### 4.6. Antidiabetic

ShK-186 from *S. helianthus* prevents diet-induced obesity and insulin resistance in mice through K_v_1.3 blockade, increasing energy expenditure, activating brown adipose tissue, improving hepatic metabolism, and enhancing insulin sensitivity [338]. Similarly, BcPLA2 from *B. caissarum* induces insulin secretion in the presence of high glucose concentration [25]; PpV9.4 and PpV19.3 from *P. physalis* stimulate insulin secretion by increasing cytosolic Ca^2+^ levels in pancreatic β-cells [299].

### 4.7. Biotechnology

Nanopore technology: Engineered nanopores formed by the actinoporin Fragaceatoxin C from *A. fragacea*, reconstituted in SM-free lipid bilayers, enable single-molecule DNA analysis by distinguishing ssDNA homopolymers and allowing dsDNA to pass through a deformable α-helical constriction. Their V-shaped transmembrane structure provides tunable pore size and chemistry, making them suitable for nucleobase discrimination and nanopore sequencing [339].

Stability properties: The synthetic peptide rShPI-1A from *S. helianthus* (PDB: 3OFW), which closely resembles the wild-type ShPI structure [340], retains its conformation and broad protease specificity, while protecting recombinant human miniproinsulin expressed in *Pichia pastoris* from proteolytic degradation [341]. Similarly, AsK132958 from *A. sulcata*, a ShK-like peptide lacking ion channel activity, is more resistant to proteases, making it a promising scaffold for engineering new functions [272]. The ICK peptide Avt1 from *A. veratra* also exhibits high stability against trypsin, chymotrypsin and pepsin for at least 16 h at 37 °C [269].

Insecticides: Bg II and Bg III from *B. granulifera* show high affinity for insect para/TipE channels [78]. APETx3 from *A. elegantissima* also completely blocks inactivation of insect Na_v_s (DmNa_v_1 and BgNa_v_1.1) and is ~10-fold more selective for insect than mammalian Na_v_1.6 channels [176]. Similarly, Av3, Av7 and Av10 from *A. viridis* inhibit DmNa_v_1, with Av3 showing no activity on mammalian Na_v_ channels and competing with the scorpion insect-specific site-3 toxin LqhαIT (K_i_: 21.4 nM) on binding to cockroach neural membranes, demonstrating high insect selectivity [43,108].

Cell Proliferation: Hact-2 from the nematocysts of the stony coral *H. actiniformis* significantly stimulated human fibroblast proliferation, with maximal activity at 50 nM [210], suggesting potential applications in tissue engineering and scaffold design.

## 5. Insights from Each Cnidaria Group

**Figure 12:**
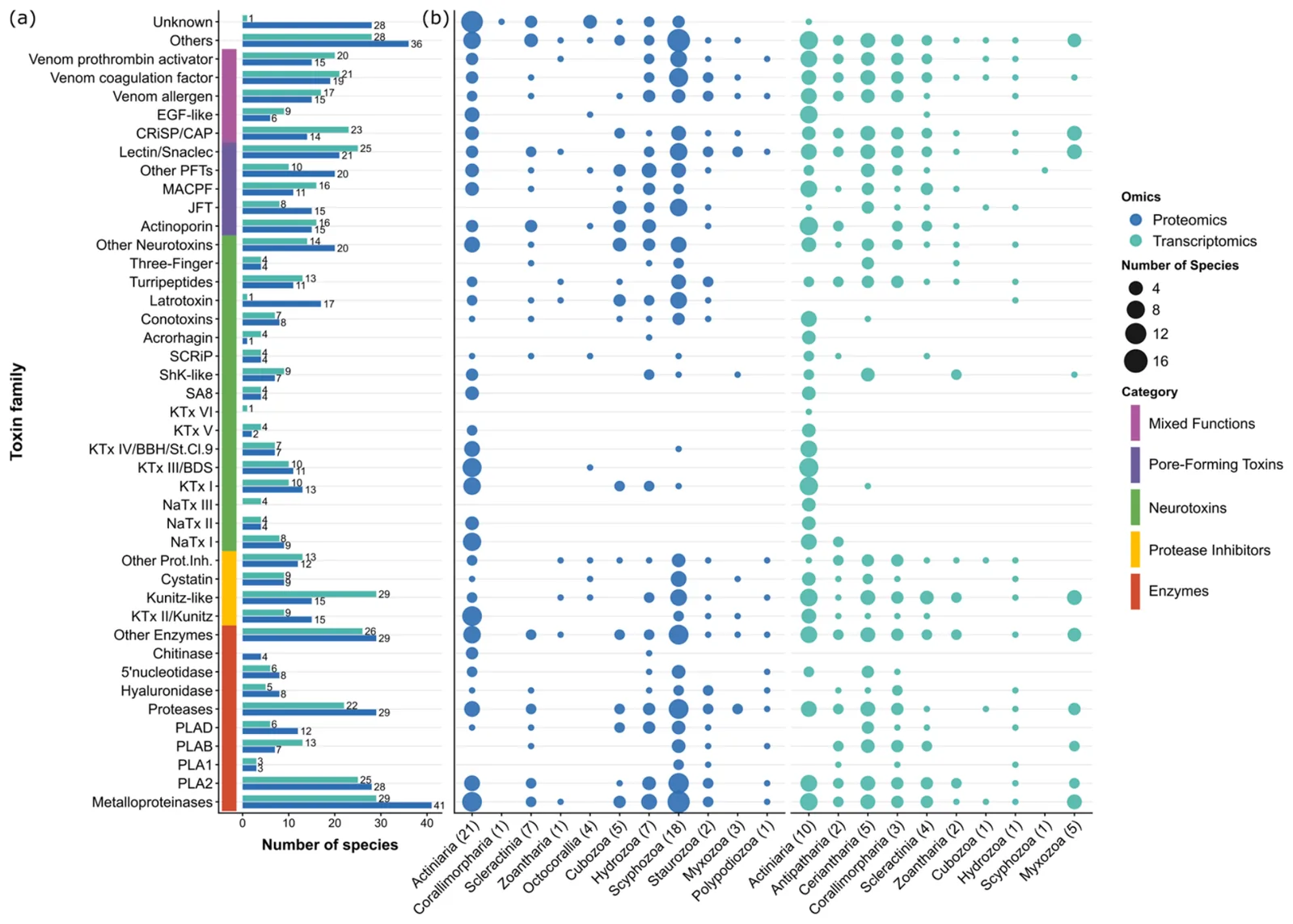
Distribution of toxin families across Cnidaria. (**a**) Number of species in which each toxin family was identified by proteomic (blue) and transcriptomic (green) studies. (**b**) Distribution of toxin families among cnidarian groups based on proteomic and transcriptomic datasets. Putative toxin repertoires from Corallimorpharia and Antipatharia are included. Each species was counted only once per toxin family, regardless of the number of homologs or isoforms identified (Table S7).

Based on the omics studies included in this review, metalloproteinases, phospholipases, lectins, Kunitz-like peptides, venom coagulation factors and CRiSP/CAP toxins are the most widespread toxin families, whereas neurotoxins show a more restricted taxonomic distribution, particularly in sea anemones (Figure 12). The following sections provide an overview of the venom composition, notable omics findings, and current knowledge gaps for each major cnidarian group.

### 5.1. Anthozoa

#### 5.1.1. Actiniaria

Sea anemones are solitary polyps that inhabit substrates ranging from rocky shores and soft sediments to the deep sea [7,342]. They possess the best-characterized venom repertoire among cnidarians, with numerous toxins following a standardized nomenclature [343]. Peptide neurotoxins appear to dominate their venoms [26,344], although many venom components remain uncharacterized [204,345]. Recent transcriptomic and proteomic studies continue to reveal extensive hidden venom diversity. For example, the nematocyst proteome of *S. haddoni* contained 12 novel Cys-rich scaffolds [346]; *P. crucifer* mucus contained 504 peptides within the 1–10 kDa fraction [347]; mucus from *S. helianthus* and *B. granulifera* contained novel crab-paralyzing toxins [98]; tentacle extracts of *Liponema brevicorne* revealed 50–70 novel peptides [348]; and *Urticina* aff. *coriacea* yielded 16 peptides from three ASIC inhibitory fractions [349]. Likewise, proteomic studies of *A. fragacea* [36], *Anthopleura dowii* [350] and *Bunodactis verrucosa* [351] identified several neurotoxins, enzymatic toxins, and actinoporins (Figure 12).

In sea anemones, toxin profiles vary among anatomical regions and developmental stages, reflecting distinct venom functions [203,352]. Accordingly, tissues with similar roles often contain similar nematocyst types specialized for particular envenomation roles [127]. Toxins are also distributed among distinct nematocyst subpopulations within the same tissue and can be produced by ectodermal gland cells [49,87,353], which release toxins into the surrounding water. *N. vectensis* has emerged as a valuable model for investigating tissue-specific toxin expression and function throughout the life cycle. For example, NvePTx1 is maternally deposited into eggs, providing protection against fish predators (Table S6) [200]. Some lineages even evolved unique structures. In Actiniidae, acrorhagi are inflatable, nematocyst-bearing structures used during aggressive intraspecific interactions, triggered by the recognition of non-self-chemical cues rather than prey stimuli [354]. The acrorhagi transcriptome of aggressive *A. elegantissima* polyps showed increased expression of type II KTx and acrorhagin I compared with non-aggressive polyps [103]. In contrast, acontia are thread-like defensive structures unique to Metridioidea, exhibiting mostly cytolytic and neurotoxic activities [27,355]. In *T. stephensoni*, the acontia appear transcriptionally inactive, likely because they contain mature nematocysts preloaded with venom [127], whereas proteomic analysis of *C. polypus* acontia revealed a venom dominated by a single NaTx type I [27], suggesting the rapid delivery of a high dose of a single major toxin rather than a diverse venom cocktail. Sea anemones also employ distinct strategies to diversify their venoms. *A. fuscoviridis* generates toxin diversity through single nucleotide variation, whereas *A. equina* relies more on insertion–deletion events [270]. Venom potency also varies between color morphs of *A. equina*, with green individuals showing greater toxicity toward zebrafish embryos than red morphs [356]. Finally, in *N. vectensis*, duplication of an ancestral ShK-like2 neuropeptide gave rise to the paralogs ShK-like1 and Shk-like2. Although both peptides are toxic to zebrafish, Shk-like1 was recruited to the nematocysts, whereas ShK-like2 retained its neuronal function, illustrating how neuropeptides can be recruited into venom during evolution [5].

#### 5.1.2. Ceriantharia

Tube-dwelling anemones build intricate living tubes using everted tubules produced by ptychocysts [357]. They are the most basal lineage of Hexacorallia [7] and share traits with Medusozoa, including linear mitochondrial genomes [358] and a long-lived pelagic larval stage resembling that of staurozoans [357]. Transcriptomic analysis of *Pachycerianthus* cf. *maua*, *Isarachnanthus nocturnus*, *Ceriantheomorphe brasiliensis* and *Pachycerianthus borealis* showed that venom repertoires are dominated by hemostatic/hemorrhagic toxins and enzymatic components, whereas neurotoxins, protease inhibitors and membrane-active toxins represent a comparatively smaller fraction. Notably, their transcriptomes lack several neurotoxin families commonly found in sea anemones, including NaTxs, KTxs, SCRiPs, ASIC and TRPV1 inhibitors (Figure 12), suggesting that these toxin families expanded after Ceriantharia diverged. *I. nocturnus* possesses fewer venom-related genes, possibly reflecting selective pressures associated with its nocturnal lifestyle and the use of green fluorescent proteins during prey capture [359]. In *Arachnanthus errans*, larvae express more toxin-related genes than polyps, likely reflecting greater exposure to environmental stressors and increased dispersal capacity during the pelagic stage. Phylogenetic analysis supports duplication of ShK-like and Kunitz-type toxin genes within Ceriantharia, with two Kunitz-like copies possibly present in their common ancestor [360].

#### 5.1.3. Zoantharia

Zoanthids are mostly colonial polyps lacking a pedal disc and connected by coenenchyme, often containing a thick mesoglea. In *Palythoa* and *Parapalythoa*, the coenenchyme incorporates sand and other particles, concealing the polyp columns [7,342]. *Palythoa* and *Zoanthus* are best known for harboring dinoflagellates that produce palytoxin, one of the most potent non-peptidic, non-nematocystic marine toxins [361]. A novel V-shaped α-helical toxin was isolated from *P. caribaeorum* venom and acts on TTX-sensitive Na_v_ channels [295], and was subsequently identified in the transcriptome of *P. variabilis*, together with a ShK/Aurelin-like toxin. Both peptides were neurotoxic and lethal to zebrafish [296]. Moreover, three Kunitz-like peptides identified in the *P. caribaeorum* transcriptome were lethal to zebrafish larvae despite exhibiting weak serine protease inhibitory activity, with PcKuz3 also displaying neuroprotective effects [167]. Combined transcriptomic and proteomic analysis of *Z. natalensis* identified diverse toxin-like peptides, including the Kunitz-like peptide ZoaKuz1, which was predicted to block Kv channels and demonstrated neuroprotective activity (Figure 12) [309].

#### 5.1.4. Corallimorpharia

Corallimorphs are solitary anthozoans closely related to stony corals but lacking a calcium carbonate skeleton [7,10]. Comprising approximately 40 species, they possess a broad oral disc and a short column, competing with other reef anthozoans for space while also preying on small fish [342]. Despite their ecological importance, venom composition remains poorly characterized. The only proteomic study to date, conducted on tentacle extracts of *Corallimorphus* cf. *pilatus*, identified 50–83 novel peptides (~1–10 kDa) (Figure 12) that may contribute to its hemolytic, cytotoxic and antimicrobial activities [348].

**Figure 13:**
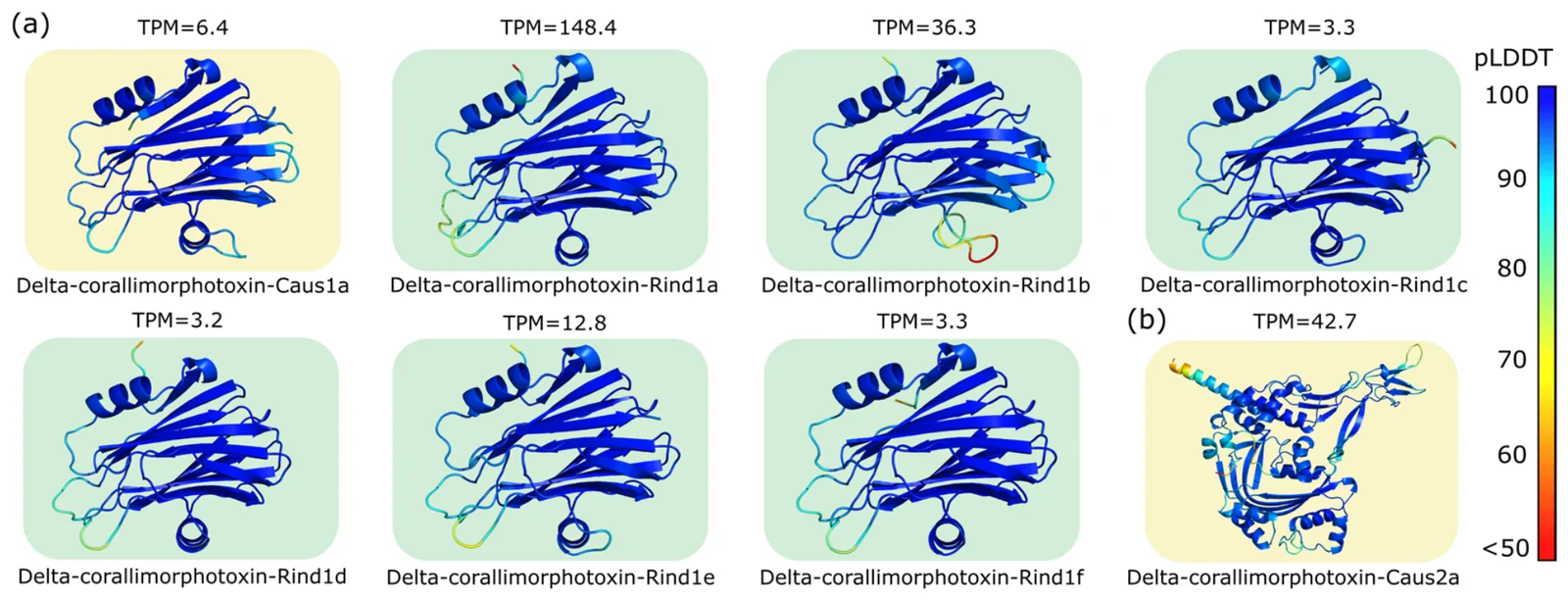
Putative pore-forming toxins detected in the polyp transcriptomes of *Corynactis australis* (Caus) and *Rhodactis indosinensis* (Rind). The nomenclature was given according to Oliveira et al. 2012 [343]. (**a**) actinoporin-like; (**b**) MACPF-like. Models are colored according to pLDDT, with higher values indicating reliable predictions.

To expand this knowledge, we analyzed the putative toxin repertoire of the polyp transcriptomes of *Rhodactis indosinensis*, *Corynactis australis* and *Ricordea yuma* (BUSCO: 97.4%, 97.5% and 91.3% completeness; Figure S1), identifying 153, 210 and 104 putative toxins, respectively. In *R. indosinensis*, the most highly expressed toxins included three lactotransferrin/draculin proteins (153.1–349.6 TPM), one actinoporin (148.4 TMP) and one venom allergen toxin (106.9 TPM). In *C. australis*, the most abundant toxins included two serine proteases (507.0–607.0 TMP), one astacin-domain metalloproteinase (513.6 TPM) and one coagulation factor X (289.6 TPM). In *R. yuma*, the most highly expressed toxins included one venom serine carboxypeptidase (123.5 TMP) and a cystatin-like toxin (108.1 TMP) (Figure 12, Table S8). Notably, the predicted structures of pore-forming toxins identified in *R. indosinensis* and *C. australis*, including actinoporins and a MACPF toxin, closely resemble those of sea anemones toxins (RMSD < 1.9 relative to the actinoporin Sticholysin I (PDB:2KS4) and RMSD: 1.4 relative to the MACPF PsTX-60A (AlphaFold: AF-P58911-F1)), including the absence of Cys residues (Figure 13, Table S8, File S2). The hemolytic activity previously reported in *C.* cf. *pilatus* [348] may be explained by the presence of these pore-forming toxins, although functional validation is required.

#### 5.1.5. Antipatharia

Black corals are colonial anthozoans whose polyps are attached to a black, proteinaceous skeletal axis covered with small thorn-like projections. They occur in two main forms: slender, whip-like colonies and more complex, bushy forms [7,342]. Black corals feed on zooplankton captured with nematocyst-bearing tentacles and mucus nets [362].

**Figure 14:**
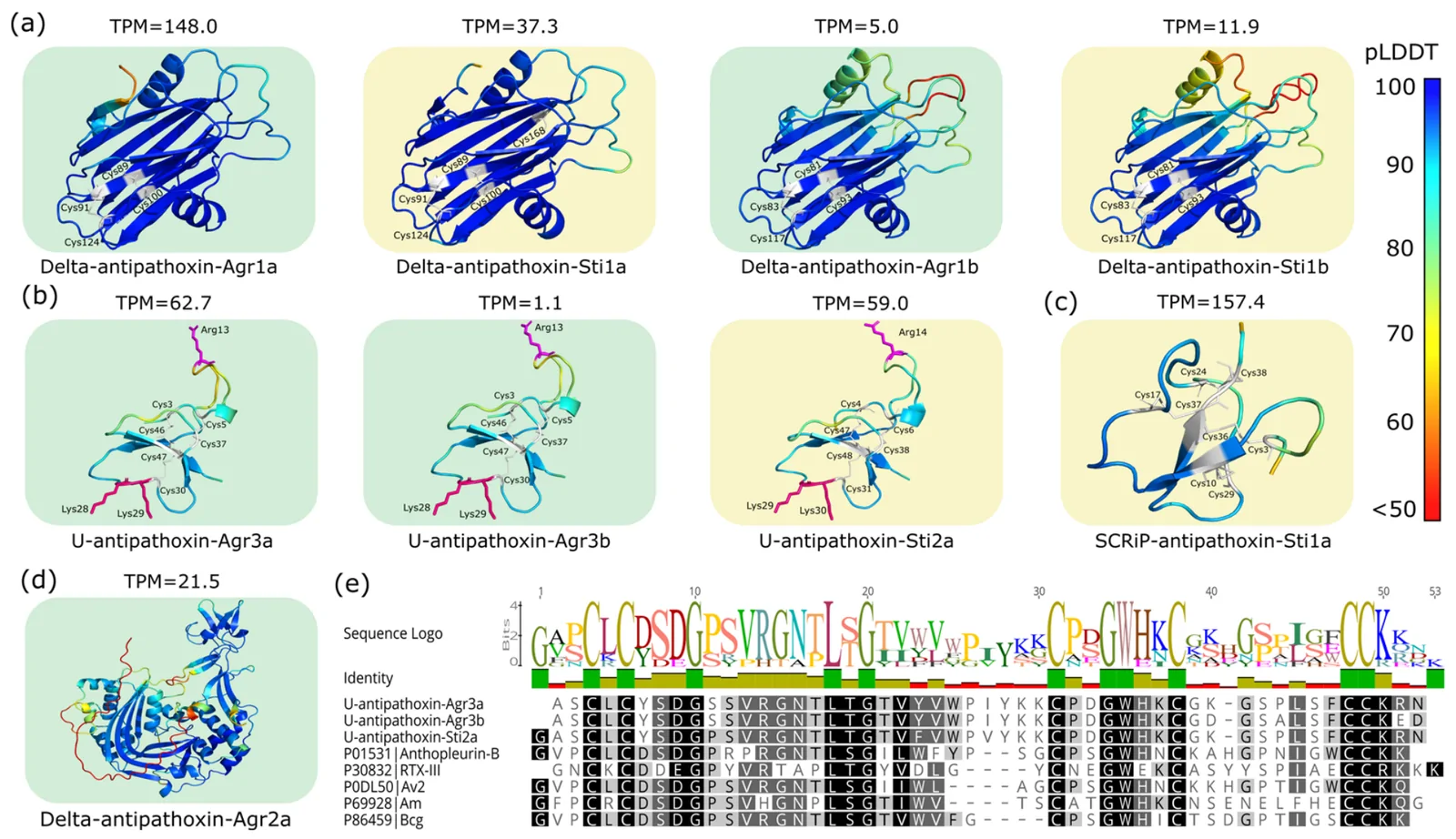
Putative toxins detected in the transcriptomes of *Antipathes griggi* (Agr) and *Stichopathes* sp. (Sti). The nomenclature was given according to Oliveira et al. 2012 [343]. (**a**) Actinoporin-like; (**b**) NaTx-like; (**c**) SCRiP-like; (**d**) MACPF-like; (**e**) sequence alignment of the three putative antipatharians neurotoxins with other NaTx from sea anemones. Models are colored according to pLDDT, with higher values indicating reliable predictions. Other colors indicate Lys residues (red), Arg residues (pink) and cysteine residues (white).

As no venom studies have been reported for Antipatharia, we analyzed the putative toxin repertoire of the transcriptomes of *Antipathes griggi* and *Stichopates* sp. (BUSCO: 71.2% and 79.9% completeness; Figure S1), identifying 97 and 142 putative toxins, respectively. In *A. griggi*, the most highly expressed toxins included a C-type isolectin (179.5 TPM), a thrombin-like enzyme (164.75 TPM) and an actinoporin (148.0 TPM). In *Stichopathes* sp., the most highly expressed toxins included an astacin-domain metalloproteinase (503.5 TPM), a thrombin-like enzyme (442.0 TPM) and a C-type isolectin (217.2 TPM) (Figure 12, Table S8). Putative pore-forming toxins identified in both species included actinoporin-like sequences containing two C-terminal disulfide bonds and a MACPF-like toxin in *A. griggi*. These additional disulfide bonds (CYS91-CYS124 and CYS89-CYS100 in Delta-antipathoxin-Agr1a and Sti1a; and CYS81-CYS93 and CYS83-CYS117 in Delta-antipathoxin-Agr1b and Sti1b) may represent adaptations to the deep-sea environment by increasing structural stability while preserving the flexible N-terminal region required for pore formation. We also identified three neurotoxins containing a PFAM Toxin_4 domain in both species, showing similar sequence identity to NaTx from sea anemones, but with slightly distinct structures (RMSD: 2.1–2.4 relative to Anthopleurin B (PDB:1AFP). These contain the common flexible Arg14 loop from Type I and II NaTx, and the presence of Lys residues (K28, K29), forming a positively charged patch, which requires experimental validation to infer their functional role. In addition, *Stichopates* sp. expressed an abundant SCRiP similar to those of stony corals (RMSD: 1.9 relative to Hact-SCRiP1 (PDB:5LAH)) (Figure 14, Table S8, File S2).

#### 5.1.6. Scleractinia

Stony corals are colonial anthozoans that secrete aragonite skeletons and capture prey using nematocyst-bearing tentacles or mucus adhesion. Most reef-building coral species also maintain an obligate symbiosis with photosynthetic dinoflagellates, from which they obtain the majority of their nutritional requirements [363]. Key toxins identified in stony corals include the neurotoxins SCRiPs [8] and Kunitz-like peptides [284,302]. Moreover, a Cys-containing actinoporin sharing ~60% sequence identity with Sticholysin II was isolated from *S. pistillata*, representing the major protein in hemolytic fractions [364]; *Goniopora* spp. produce neurotoxins, including an 88-aa, 10-Cys peptide that slowed Na_v_ currents [286,287,288], and a 19-kDa calcium channel blocker [285]. Aqueous extracts from multiple stony coral families exhibited hemolytic, antimicrobial and mouse toxicity [365], while nematocyst extracts from *Porites astreoides*, *Pseudodiploria strigosa* and *Siderastrea sidereal* displayed hemolytic, nociceptive and vasoconstrictive activities and showed PLA_2_ and serine protease activities, with mass spectrometry analysis revealing novel insecticidal peptides (3–6 kDa) (Figure 12) [366].

Stony corals employ distinct venom delivery strategies. In *Galaxea fascicularis*, catch tentacles are used for prey capture, and their transcriptomes express paralytic toxins and hemolysins, whereas sweeper tentacles have higher PLA_2_ activity associated with tissue damaging during competition [367]; solitary fungiid corals produce nematocyst-rich mucus that induces tissue necrosis in neighboring colonies [368]. Venom composition also varies with environmental conditions and nutritional strategies. Captive colonies of *S. pistillata* show lower hemolytic activity and nematocyst density than wild colonies [364]; in contrast, the azooxanthellate coral *Tubastraea coccinea* possesses a broader predicted toxin repertoire than the zooxanthellate coral *Acropora digitifera*, including cytolytic, neurotoxic, dyshomeostatic and protease inhibitor toxins [363,364,369,370]. Finally, venom from four scleractinian species exhibited PLA_2_ inhibitory activity [29], revealing a previously unreported functional property of cnidarian venoms.

#### 5.1.7. Octocorallia

Octocorallians are colonial anthozoans (soft corals, blue corals, sea pens and gorgonians) with skeletons composed of calcite or protein (gorgonin), aragonite in blue corals, or no skeleton at all [7]. Besides producing diverse secondary metabolites for chemical defense [371], they also possess proteinaceous venoms. Nematocyst fractions from *Nephthea* sp., *Dendronephthya* sp. and *Heteroxenia fuscescens* demonstrate hemolytic activity against human erythrocytes and are lethal to mice, with *H. fuscescens* displaying the highest toxicity (LD_50_: 0.7 mg/kg) [372]. More recently, a proteotranscriptomic study of the zooplanktivorous *Eunicella singularis* also identified toxins with novel scaffolds and distinct cytolytic proteins distributed both inside and outside the nematocysts [373] (Figure 12).

### 5.2. Medusozoa

#### 5.2.1. Scyphozoa

True jellyfish are often associated with blooms that can disrupt ecosystems, but remain essential components of marine food webs. Their stings cause symptoms from itching and pain to severe systemic toxicity [280,374]. Proteomic analyses show that scyphozoan venoms are dominated by metalloproteinases and PLA_2_s, together with proteases and pore-forming toxins such as JFTs (Figure 12). This pattern has been reported in numerous species, including *A. aurita* [375], *Aurelia coerulea* [376], *Chrysaora caliparea* [280], *C. fuscescens* [377], *C. lactea* [378], *Cyanea nozakii* [280,379], *Cyanea* sp. [380], *Lychnorhiza malayensis* [280], *P. noctiluca* [37], *P. camtschatica* [39], *Phyllorhiza punctata* [374], *Rhopilema esculentum* [381], *Sanderia malayensis* [381] and *Stomolophus meleagris* [376,382]. The presence of ShK domains in putative venom proteins from *P. noctiluca* [37] and *C. fuscescens* [377] suggests ion channel-modulating activity. Likewise, venom from *Catostylus* sp. contains a neurotoxin with high predicted affinity for hKv3.1 [277], whereas the NaTx CcNT identified in *C. capillata* may contribute to prey paralysis [282]. Venom composition also differs among jellyfish, with no protein toxins shared among the three nematocyst proteomes [280], and *P. punctata* JFTs are phylogenetically distinct from those of cubozoans [374].

In *Nemopilema nomurai*, metalloproteinases are the predominant venom proteins, consistent with the venom’s high metalloproteinase activity. They are likely responsible for the blistering and wheal formation observed after envenomation, and metalloproteinase inhibitors may be potential therapeutics [383]. Phospholipases, thrombin-like toxins, hemolysins, and neurotoxins also contribute to envenomation, with the burning pain likely resulting from neurotoxic activity and TRPV1 activation [33,383,384,385]. Similarly, *R. pulmo* exhibits proteolytic activity, likely mediated by the ZnMP Rhizoprotease [38], and also produces the hemolytic cytolysin Rhizolysin [303]. In contrast, PLA_2_s are the predominant venom proteins in *C. capillata*, consistent with the venom’s high PLA_2_ activity, followed by neurotoxins and proteases. The skin swelling and erythema caused by its stings suggest that PLA_2_ inhibitors may be potential therapeutics [33].

#### 5.2.2. Cubozoa

Box jellyfish possess a characteristic cubic bell with up to 15 tentacles, and their venoms are among the deadliest in the animal kingdom [386]. Contact with species such as *C. fleckeri* can be fatal, largely due to JFTs that induce erythrocyte lysis and severe cytotoxicity [35,213,215,217,219]. JFTs have also been identified in the proteomes of *Carybdea brevipedalia* [387], *Chiropsalmus quadrumanus* [378] and *C. indrasaksajiae* [386]. In contrast, JFTs were not detected in *Tamoya haplonema*, whose venom contains MACPFs, metalloproteinases and neurotoxins, illustrating the complexity of cubozoan venoms [378] (Figure 12). Tentacle extracts from several cubozoans also activate TRPV1, likely contributing to the sting-induced pain [383], while ZnMPs may promote extracellular matrix degradation [35,217]. In addition, the venom of *C. marsupialis* contains cytolysins [275,276] and neurotoxins [275] that may contribute to its high toxicity. Moreover, venom composition varies among cubozoan species and developmental stages. The hemolytic activity of *C. indrasaksajiae* venom increases in a concentration-dependent manner, resulting in complete erythrocyte lysis at 6.58 μg/mL [386]. In *Carukia barnesi* (Irukandji jellyfish), immature and mature individuals exhibit distinct venom protein profiles, possibly reflecting the dietary shift from invertebrate to vertebrate prey [388]. Likewise, *C. brevipedalia* venom causes lethality, edema and hemorrhage in zebrafish larvae, consistent with the presence of cytolysins, hemolysins, neurotoxins and hydrolases identified by proteomics [387]. Transcriptomic analysis of *A. alata* further revealed 450 venom-related transcripts highly expressed in the tentacles and gastric cirri, including JFTs and ZnMPs homologues [389], suggesting a dual role in venom and digestion.

#### 5.2.3. Hydrozoa

Hydrozoans are predatory cnidarians that occur as solitary or colonial forms with diverse life forms and possess one of the broadest distributions of toxin families across Cnidaria (Figure 12). *Hydra* species are widely used as laboratory models, leading to the discovery of the pore-forming toxins hydralysins [264,265] and the actinoporin-like toxins HALTs [239,240]. Moreover, the siphonophore *P. physalis* delivers one of the most venomous stings in the ocean, containing the hemolysin physalitoxin, which accounts for ~28% of the total nematocyst protein content. Physalitoxin lyses rat erythrocytes with a potency 5–6 orders of magnitude higher than cobra cardiotoxin or melittin [14]. The venom also contains several neurotoxins [299,300,301], which may contribute to envenomation. Its tentacle extracts also activate TRPV1, which may underlie the intense burning pain caused by stings [383]. In another siphonophore, *Velella velella*, proteomic and biochemical analysis identified several hydrolytic enzymes, including proteases, phospholipases, hyaluronidases, DNases, and chitinases [390].

Fire corals (*Millepora* sp.) can also cause severe burns, blisters and pain in humans [391]. Identified toxins include the cytolysin MCTx-1 from *M. dichotoma* [292], the hemolytic PLA_2_ Milleporin 1 from *M. platyphylla* [30], and the actinoporin-like toxin Alciporin from *M. alcicornis* [291]. Proteomic analysis of *M. alcicornis* and *M. complanata* nematocysts further identified diverse actinoporins and toxic enzymes [391,392]. In *M. complanata*, bleaching increased hemolytic activity but reduced PLA_2_ activity, suggesting compensatory regulation of venom composition after the loss of nutrients supplied by symbiotic algae [391]. In the hydromedusa *Olindias sambaquiensis*, venom composition was nearly identical across different nematocyst types despite differences in their penetrative ability, suggesting that multiple nematocyst types deliver the same venom [393]. Venom composition also remained largely stable between studies conducted in 2013 and 2019, with only pore-forming toxins detected exclusively in the latter [17,393]. In another hydromedusa, *Gonionemus vertens*, metalloproteinases and Kunitz-type protease inhibitors dominate the venom [394]. Finally, de novo sequencing of venom peptides from the colonial hydroid *Macrorhynchia philippina* identified 19 novel peptides (<1 kDa) with no similarity to known animal toxins and may contribute to pain and edema in mice [395].

#### 5.2.4. Staurozoa

Stalked jellyfish are sessile medusae attached to hard substrates by a stalk, and their venoms remain largely unexplored. However, proteomic analysis of the tentacles of *Calvadosia cruxmelitensis* [396] and the nematocysts of *Haliclystus antarcticus* [370] identified metalloproteinases, PLA_2_, and actinoporins, while *H. antarcticus* also contained a JFT (Figure 12).

### 5.3. Endocnidozoa

Myxozoan nematocysts are modified into polar capsules, which contain a tightly coiled, eversible polar tubule that anchors to the host, pulls the spore toward it, and releases capsule contents into the surrounding host tissue [397]. Proteomic analysis revealed that *Polypodium hydriforme* (the only known representative of Polypodiozoa) possesses a greater diversity of putative toxins than myxozoans [396] (Figure 12). In the malacosporean *Buddenbrockia plumatellae*, C-type lectins and serine peptidase inhibitors are expressed during presporogonic and early sporogonic stages, suggesting that toxin functions have been repurposed for parasite development and host interactions, including host muscle degradation, rather than prey capture or defense [396]. Likewise, venom-like compounds in the myxozoan *Ceratonova shasta* are expressed during early gill infection and later during intestinal spore formation, indicating that venom components have been co-opted to facilitate host invasion and parasite proliferation [398]. Phylogenetic analysis further shows that toxin genes from five myxozoan species form distinct clades separate from those of free-living cnidarians and have lost many toxin families. Nevertheless, most species retained Kunitz, M12B metalloproteinases and CRISP toxins, suggesting that these toxins continue to play important roles in endoparasitism [15].

## 6. Future Perspectives

Recent advances in omics technologies are transforming venom research and are expected to accelerate the discovery, annotation and experimental validation of novel venom-derived peptides, particularly from marine organisms and understudied taxa, through integrated multi-omics approaches (Figure 2). Structural prediction tools [311,312,313], signal peptide predictors such as SignalP [399], and machine learning approaches, including *ToxClassifier* [400], are improving the identification of candidate toxins and their putative molecular targets prior to experimental validation. In parallel, single-cell [240] and spatial proteomics (mass spectrometry imaging) [127,140,201] are beginning to reveal toxin expression at the cellular level and their spatial localization within tissues, respectively. Synthetic biology techniques and heterologous protein expression [310,323] will also improve the production of sufficient quantities of purified toxins for functional, pharmacological and preclinical studies. Finally, the continued expansion of public venom databases, such as ToxProt [401] and VenomZone [402], will strengthen comparative analysis across venomous lineages and improve the annotation of homologous toxin families. Together, these advances will unlock the full bioactive and evolutionary potential of cnidarian venoms.

## 7. Materials and Methods

### 7.1. Bibliographic Research and Data Collection

This review includes information from studies published up to 29 July 2026. To compile data on cnidarian toxins, studies associated with toxin entries in the ToxProt database were first retrieved (Table S2). A complementary literature search was then conducted in Google Scholar and PubMed using combinations of the keywords *Cnidaria*, *venom*, *toxin*, *transcriptome*, *proteome* and *venomics*. Duplicate records corresponding to ToxProt entries were removed, whereas additional studies reporting characterized toxins or omics analysis were retained (Table S3). Proteomics studies included proteomic, peptidomic, proteotranscriptomic, and proteogenomic analyses, although only toxins detected at the protein level were considered (Table S4). Transcriptomic studies comprised those reporting toxins identified exclusively at the RNA level (Table S5). Scientific names were verified and updated according to the World Register of Marine Species (WoRMS) database (Table S7) [403]. The year of publication was recorded for the first study reporting either toxin isolation (protein or nucleotide) or an omics-based venom analysis for each species. Studies including representatives from different cnidarian subphyla were treated separately (Table S7). For studies reporting venom sampling localities, geographic coordinates were recorded when available; otherwise, approximate coordinates were assigned based on the reported region. Duplicate records from the same species collected at the same locality were removed, whereas records from different species collected at the same locality were retained. Sampling locations were assigned to marine realms according to the Marine Ecoregions of the World (MEOW) classification [16] using QGIS v3.40.5 (https://qgis.org/ (accessed on 20 January 2026)) (Table S7, File S1).

### 7.2. Transcriptomic Analysis

Transcriptomes of three Corallimorpharia species from BioProject PRJNA313487 (*Corynactis australis* (SRR3198576, SRR3198579, SRR3198580)), *Rhodactis indosinensis* (SRR3201278-SRR3201281) and *Ricordea yuma* (SRR3201251-SRR3201253), and two Antipatharia species from BioProject PRJNA809900 (*Antipathes griggi* (SRR18121403)) and *Stichopathes* sp. (SRR18121404), were downloaded from the NCBI Sequence Read Archive (SRA). Adapter sequences and low-quality reads were removed using Trimmomatic v0.39 [404], followed by *de novo* transcriptome assembly with Trinity v2.15.2 [405]. Assembly completeness was assessed with BUSCO v5.5.0 [406] against the Metazoa lineage dataset (Figure S1). Coding regions were predicted using TransDecoder v5.5.0 (minimum ORF length of 40 amino acids; Haas, B.J., https://github.com/TransDecoder/TransDecoder (accessed on 12 August 2025)). ORF prediction was supported by DIAMOND v2.1.13 [407] searches against the UniRef90 database (downloaded on 12 August 2025; E-value ≤ 1 × 10^−5^) [408] and HMMER 3.3.2 [409] searches against the Pfam database (downloaded on 12 August 2025; E-value ≤ 1 × 10^−10^) [410]. For toxin annotation, predicted proteins were searched against a custom toxin database comprising ToxProt [401] (downloaded on 4 September 2025) and cnidarian venom proteins retrieved from the NCBI protein database using the query “Cnidaria” AND (“toxin” OR “venom”) (downloaded on 11 September 2025). Similarity searches were performed using DIAMOND v2.1.13 (E-value ≤ 1 × 10^−5^) [407]. Only proteins containing a predicted signal peptide (probability > 70%) identified by SignalP v6 [399] were retained as candidate toxins. Candidate toxins were further validated by reciprocal DIAMOND v.2.1.13 searches against ToxProt (E-value ≤ 1 × 10^−5^), followed by BLASTn v2.12.0 searches against the NCBI Cnidaria protein database (taxid:6073, downloaded on 22 September 2025) and domain annotation using HMMER v.3.3.2 against the Pfam database (E-value ≤ 1 × 10^−10^). Transcript abundance was estimated with Kallisto v0.51.1 [411] as transcripts per million (TPM). Transcripts with TPM < 1 were excluded from downstream analysis. Candidate toxins were classified into venom categories according to ToxProt annotations [401]. Protein structures of selected toxins were predicted using ColabFold/AlphaFold2 [311] and visualized with PyMOL v3.1.0 [412].

## 8. Conclusions

This review synthesizes the current knowledge of cnidarian venoms by integrating information from 342 curated toxins from the ToxProt database, 76 additional toxins reported in the literature, and 81 proteomic and 31 transcriptomic studies, covering 145 cnidarian species. The integration of available omics data reveals that metalloproteinases, phospholipases, lectins, Kunitz-like peptides, venom coagulation factors, and CRiSP/CAP toxins are the most widespread toxin families across Cnidaria, whereas neurotoxins exhibit a more restricted taxonomic distribution in sea anemones. Among the characterized toxins, 37 have experimentally resolved structures, and 78 exhibit promising biomedical or biotechnological potential. Despite these advances, toxin discovery remains strongly biased toward sea anemones, highlighting the vast unexplored venom diversity across the phylum. We also provide the first putative toxin profiles for Corallimorpharia (corallimorphs) and Antipatharia (black corals), revealing previously unrecognized pore-forming toxins, neurotoxins, and other venom components that warrant further proteomic and functional validation. Continued integration of transcriptomic, proteomic, structural, and functional data will be essential to improve toxin annotation, deepen our understanding of venom evolution, and accelerate the discovery of novel bioactive molecules. Overall, this review provides a comprehensive resource for future studies and a valuable reference framework for researchers investigating the diversity, evolution and biomedical potential of cnidarian venoms.

## Figures and Tables

**Table 2:** Representative toxins from the Type I and Type II NaTx from Cnidaria.

Name	Type	Species	UniProt	LD_50_ (μg/kg)	Targets	Ref.
Ae1	I	*Actinia equina*	Q9NJQ2	25 (Crabs, MLD)	-	[52]
AETX I	I	*Anemonia erythraea*	P69943	2.2 (Crabs)	-	[53]
ATX I	I	*Anemonia sulcata*	P01533	<2 (Crabs, LD_100_)	-	[54,55,56]
ATX II	I	*Anemonia sulcata*	P01528	<2 (Crabs, LD_100_)	DmNa_v_1, Na_v_1.1–Na_v_1.6	[55,56,57,58]
ATX V	I	*Anemonia sulcata*	P01529	-	-	[59]
Av2	I	*Anemonia viridis*	P0DL49	-	-	[47]
Am III	I	*Antheopsis maculata*	P69928	70 (Crabs)	-	[60]
AFT-I	I	*Anthopleura fuscoviridis*	P10453	98 (Mice); 100–150 (Crabs)	-	[61]
AFT-II	I	*Anthopleura fuscoviridis*	P10454	450 (Mice); 100–150 (Crabs)	Na_v_1.1–Na_v_1.6	[61,62]
Anthopleurin C	I	*Anthopleura elegantissima*	P01532	1 (Crabs)	-	[51,63]
APE 1-1	I	*Anthopleura elegantissima*	P0C1F0	10 (Crabs)	-	[51]
APE 1-2	I	*Anthopleura elegantissima*	P0C1F1	-	-	[51]
APE 2-2	I	*Anthopleura elegantissima*	P0C1F3	-	-	[51]
Hk2a	I	*Anthopleura* sp.	P0C5F4	1400 (Mice)	-	[50,64]
Hk7a	I	*Anthopleura* sp.	P0C5F5	-	-	[50]
Hk8a	I	*Anthopleura* sp.	P0C5F6	-	-	[50]
Hk16a	I	*Anthopleura* sp.	P0C5F7	-	-	[50]
Anthopleurin A	I	*Anthopleura xanthogrammica*	P01530	-	Na_v_1.5	[65,66,67]
Anthopleurin B	I	*Anthopleura xanthogrammica*	P01531	-	Na_v_1.5	[66,68,69]
Bc-III	I	*Bunodosoma caissarum*	Q7M425	600 (Mice)	Na_v_1.1–Na_v_1.6	[62,70]
Cangitoxin I	I	*Bunodosoma cangicum*	P82803	-	-	[71]
Cangitoxin II	I	*Bunodosoma cangicum*	P0C7P9	-	Na_v_1.1–Na_v_1.6	[72,73]
Cangitoxin III	I	*Bunodosoma cangicum*	P0C7Q0	-	Na_v_1.1	[73]
Toxin Bcg 30.24	I	*Bunodosoma cangicum*	P86459	-	-	[74]
δ-AITX-Bca1a	I	*Bunodosoma capense*	P0DTR3	-	-	[75]
NavTx-Bg1	I	*Bunodosoma goanense*	-	-	rNav1.4, hNa_v_1.5	[76]
Bg II	I	*Bunodosoma granuliferum*	P0C1F4	0.4 (Mice)	Na_v_1.2, Na_v_1.4, Na_v_1.5, para/tipE	[77,78]
Bg III	I	*Bunodosoma granuliferum*	P0C1F5	21 (Mice)	Na_v_1.2, Na_v_1.4, Nav1.5, para/tipE	[77,78]
NaTx-I	I	*Calliactis polypus*	P0DY38	-	-	[27]
CgNa	I	*Condylactis gigantea*	P0C280	1 (Crabs); 5 μg/g (Fly, LD_100_)	-	[79,80,81]
Cp I	I	*Condylactis gigantea*	P0CH42	7.3 (Crabs, MLD)	-	[80]
AnmTx Cj 1c-1	I	*Epiactis japonica*	P0DPE5	30 μg/g (Fly); 30 μg/g (Shrimp, LD_100_)	-	[79]
Hau IV	I	*Heteractis aurora*	A0A0N7KBN8	53 (Crabs)	-	[82]
Rc I	I	*Radianthus crispa*	P0C5G5	7.3 (Crabs, MLD)	-	[83]
Gigantoxin II	I	*Stichodactyla gigantea*	Q76CA3	70 (Crabs)	-	[84,85]
δ-TATX-Cad1a	II	*Cryptodendrum adhaesivum*	D2KX90	20 (Crabs)	-	[86]
δ-TATX-Hhe1a	II	*Heterodactyla hemprichii*	D2KX91	4.5–14 (Crabs)	-	[86]
Halcurin	II	*Isohalcurias carlgreni*	P0C5G6	5.8 (Crabs)	.	[48]
Nv1	II	*Nematostella vectensis*	A7SCE5	-	DmNa_v_1, rNa_v_1.4, hNa_v_1.5	[87,88]
Neurotoxin I	II	*Radianthus crispa*	P30831	-	-	[89]
Neurotoxin II	II	*Radianthus crispa*	P30783	-	-	[90]
Neurotoxin IV	II	*Radianthus crispa*	P30784	-	-	[91]
Neurotoxin V	II	*Radianthus crispa*	P30785	-	-	[91]
RTX-III	II	*Radianthus crispa*	P30832	-	Na_v_1.3, Na_v_1.6, BgNa_v_1, VdNa_v_1	[92,93]
RTX-IV	II	*Radianthus crispa*	P30832	-	Na_v_1.2, Na_v_1.6, BgNa_v_1, VdNa_v_1	[92,93]
RpII	II	*Radianthus magnifica*	P01534	15 (Crabs); 4.2 (Mice)	-	[94,95,96]
RpIII	II	*Radianthus magnifica*	P08380	10 (Crabs); 2.4 (Mice)	-	[95,97]
Gigantoxin III	II	*Stichodactyla gigantea*	Q76CA0	120 (Crabs)	-	[84,85]
Sh1	II	*Stichodactyla helianthus*	P19651	0.3 (Crabs)	-	[44,98,99]
SHTX-IV	II	*Stichodactyla haddoni*	B1B5I9	93 (Crabs)	-	[100]
δ-TLTX-Ta1a	II	*Thalassianthus aster*	D2KX92	24 (Crabs)	-	[86]

LD_50_—median lethal dose; LD_100_—lethal dose (100%); MLD—minimum lethal dose.

**Table 4:** Representative toxins from the Type I KTx from Cnidaria.

Name	Subtype	Species	UniProt	LD_50_ (μg/kg)	Targets	Ref.
AETX K	1a	*Anemonia erythraea*	Q0EAE5	-	K_v_1.1, K_v_1.2, K_v_1.3	[114,122]
Hmk	1a	*Radianthus magnifica*	O16846	-	K_v_1.1, K_v_1.2, K_v_1.3, K_v_1.5, K_v_7.2	[115,122,123]
Shk	1a	*Stichodactyla helianthus*	P29187	-	K_v_1.1–Kv1.7, K_v_Ca3.1, K_v_3.1, K_v_3.4	[113,116,124,125]
ShKT-Ts1	1a	*Telmatactis stephensoni*	P0DRH8	-	-	[126,127]
κ1.3-TLTX-Ta1a	1a	*Thalassianthus aster*	E2S066	-	K_v_1.3	[121,128]
Aek	1b	*Actinia equina*	P81897	-	-	[117]
Kaliseptine	1b	*Anemonia sulcata*	Q9TWG1	-	K_v_1.2	[118]
BcsTx1	1b	*Bunodosoma caissarum*	C0HJC2	-	rK_v_1.2, rK_v_1.1, rK_v_1.3, rK_v_1.6, insect Shaker IR	[129]
BcsTx2	1b	*Bunodosoma caissarum*	C0HJC3	-	rK_v_1.6, rK_v_1.1, rK_v_1.2, rK_v_1.3, insect Shaker IR	[129]
BgK	1b	*Bunodosoma granuliferum*	P29186	4.5 (Mice)	K_v_1.1, K_v_1.2, K_v_1.3, K_v_1.6, KCa3.1	[113,119,130,131,132,133]
Metridin	1b	*Metridium senile*	P11495	-	-	[134]
OspTx2a	1b	*Oulactis* sp.	A0A330KW27	-	K_v_1.2, K_v_1.6	[135]
OspTx2b	1b	*Oulactis* sp.	A0A330KUG5	-	-	[136]

LD_50_—median lethal dose.

**Table 6:** Representative toxins from the Type III KTx from Cnidaria.

Name	Species	UniProt	Targets	Ref.
BDS-1	*Anemonia sulcata*	P11494	K_v_3.4, K_v_3.1, K_v_3.2, hNa_v_1.7, Na_v_1.3	[170,171,179,180]
BDS-2	*Anemonia sulcata*	P59084	K_v_3.4, K_v_3.1, K_v_3.2	[170,179]
BDS-1	*Anemonia viridis*	P0DMX6	-	[120,155,181]
BDS-5	*Anemonia viridis*	P0DMX9	-	[181,182]
BDS-16	*Anemonia viridis*	P0DQN6	-	[181]
Am II	*Antheopsis maculata*	P69930	-	[60]
APETx1	*Anthopleura elegantissima*	P61541	K_v_11.1/hERG, K_v_11.3, Na_v_1.2–Na_v_1.6, Na_v_1.8, K_v_1.4	[169,172,176,183]
APETx2	*Anthopleura elegantissima*	P61542	ASIC3, K_v_11.1/hERG, Na_v_1.2, Na_v_1.6, Na_v_1.8, K_v_3.4	[173,174,176,184,185]
APETx3	*Anthopleura elegantissima*	B3EWF9	DmNa_v_1, BgNa_v_1, VdNa_v_1, Na_v_1.2, Nav1.3, Na_v_1.4, Na_v_1.6	[176]
APETx4	*Anthopleura elegantissima*	C0HL40	K_v_10.1/hEAG1, K_v_1.4, K_v_1.3, K_v_1.5, K_v_2.1, Na_v_1.4, Na_v_1.5, Na_v_1.6.	[175]
BcIV	*Bunodosoma caissarum*	P84919	(MLD: ~2000 μg/kg (Crabs))	[186]
Bcg 31.16	*Bunodosoma cangicum*	P86461	-	[74]
Bcg 25.52	*Bunodosoma cangicum*	P86462	-	[74]
Bcg 28.78	*Bunodosoma cangicum*	P86463	-	[74]
Bcg 29.21	*Bunodosoma cangicum*	P86464	-	[74]
Bg1a	*Bunodosoma granuliferum*	G0W2H7	-	[98]
Bg1b	*Bunodosoma granuliferum*	G0W2H8	-	[98]
Bg1c	*Bunodosoma granuliferum*	G0W2H9	-	[98]
Bg1d	*Bunodosoma granuliferum*	G0W2I0	-	[98]
Bg1e	*Bunodosoma granuliferum*	G0W2I1	-	[98]
Hcr 1b-2	*Radianthus crispa*	C0HL52	ASIC1a, ASIC3, K_v_1.3–1.6, K_v_2.1, K_v_4.2, K_v_7.1–7.4, K_v_11.1/hERG, QKT1, Na_v_1.1–1.8, BgNav, Ca_v_3.1, Ca_v_3.2, Ca_v_3.3	[187,188,189]
Hcr 1b-3	*Radianthus crispa*	C0HL53	ASIC1a, ASIC3	[188,189]
Hcr 1b-4	*Radianthus crispa*	C0HL54	ASIC1a, ASIC3	[188,189]
Hcr 1b-1	*Radianthus crispa*	P0DL87	hASIC3	[190]
Hmg 1b-5	*Radianthus magnifica*	C0HLS4	ASIC3, nAChRs	[177]
Hmg 1b-2	*Radianthus magnifica*	C0HM68	ASIC3, nAChRs	[177,191]
Hmg 1b-4	*Radianthus magnifica*	P0DRI4	ASIC1a, ASIC3, nAChRs	[177,191]
CogiTx1	*Condylactis gigantea*	C0HM42	-	[192]
PhcrTx2	*Phymanthus crucifer*	C0HK75	-	[193]
Crassicorin I	*Urticina crassicornis*	A0A1X9QHL1	-	[194]
Crassicorin II	*Urticina crassicornis*	P0DUG3	-	[194]

MLD—minimum lethal dose.

**Table 10:** Other toxins isolated from Cnidaria.

Name	Species	UniProt	LD_50_ (μg/kg)	Targets/Function	Ref.
Acrorhagin II	*Actinia equina*	Q3C256	80 (Crabs)	-	[266]
Acrorhagin I	*Actinia equina*	Q3C258	520 (Crabs)	Zinc and nickel	[266,267]
B3a-c29555_c4_g4	*Actinia equina*	-	-	Ca_v_3.1 (Molecular docking)	[270]
U-AITX-Ate1	*Actinia tenebrosa*	P0DQJ2	-	Cytotoxic	[271]
AETX II	*Anemonia erythraea*	P69944	0.53 (Crabs)	-	[53]
AETX III	*Anemonia erythraea*	P69945	0.28 (Crabs)	-	[53]
AsK132958	*Anemonia sulcata*	P0DPF1	-	ShK-like	[272]
Acatx1	*Anthopleura cascaia*	A0A6I8WFP9	-	-	-
Avt1	*Aulactinia veratra*	P0DRH7	-	-	[269]
BTTX I	*Bolocera tuediae*	-	500 (Crabs, LD_100_)	-	[273]
BTTX II	*Bolocera tuediae*	-	40 (Crabs, LD_100_)	-	[273]
GRX	*Bunodosoma cangicum*	P58305	400 (Mice)	Neurotoxic	[274]
SA9-Cpp1a	*Calliactis polypus*	P0DY44	-	-	[26]
Unknown 12C protein	*Calliactis polypus*	P0DY40	-	-	[27]
ShKC10-Cpp1a	*Calliactis polypus*	P0DY41	-	ShK-like	[26,27]
NaH-Cpp1a	*Calliactis polypus*	P0DY43	-	-	[26]
CmHl1	*Carybdea marsupialis*	-	-	1.4% hemolysis: 0.125 μg/mL (Human RBCs)	[275]
CmHl5	*Carybdea marsupialis*	-	-	2.4% hemolysis: 0.125 μg/mL (Human RBCs)	[275]
CmHl7	*Carybdea marsupialis*	-	-	10% hemolysis: 1.0 mg/mL (Human RBCs)	[275]
Hemolysin	*Carybdea marsupialis*	-	-	HC_50_: 27.91 ng/mL (Sheep RBCs)	[276]
CmNt	*Carybdea marsupialis*	-	15 μg/g (Crabs)	Neurotoxic	[275]
Toxin α-KTx 1.13	*Catostylus* sp.	-	-	hKv3.1 (Molecular docking)	[277]
ACE-inhibitory peptide	*Chiropsoides quadrigatus*	P0DQQ8	-	ACE	[278]
Chrysaoralin	*Chrysaora quinquecirrha*	-	-	Hemolytic lectin	[279,280]
Cystatin J	*Cyanea capillata*	Q331K1	-	Papain	[281]
CcNT	*Cyanea capillata*	-	-	Na_v_ channel blocker	[282]
EGCase	*Cyanea nozakii*	Q9GV16	-	Glycosphingolipids	[283]
CjTL8	*Epiactis japonica*	P0DPE6	10 μg/g (Shrimp, LD_100_)	Neurotoxic	[79]
CjTL7	*Epiactis japonica*	P0DPE7	-	Neurotoxic	[79]
GcKuz1	*Goniopora columna*	-	-	Kallikrein, FXIIa	[284]
Goniopora toxin	*Goniopora* sp.	-	-	Ca_v_ channels	[285]
GPT	*Goniopora* sp.	-	-	Na_v_ channels	[286,287,288]
Hact-1	*Heliofungia actiniformis*	P0DUS1	-	Lipopolysaccharides	[210,289]
Hact-2	*Heliofungia actiniformis*	C0HM69	-	-	[210]
Hact-3	*Heliofungia actiniformis*	C0HM25	-	-	[210]
Hact-4	*Heliofungia actiniformis*	C0HM26	-	-	[210]
Hau I	*Heteractis aurora*	A0A0P0UTJ1	14 (Crabs)	-	[82]
Ms11a-1	*Metridium senile*	P0DRC0	-	nAChR	[290]
Ms11a-2	*Metridium senile*	P0DRC1	-	nAChR	[290]
Ms11a-3	*Metridium senile*	P0DRC2	-	nAChR	[290]
Ms11a-4	*Metridium senile*	P0DRC3	-	nAChR	[290]
Alciporin	*Millepora alcicornis*	-	-	Hemolytic	[291]
MCTx-1	*Millepora dichotoma*	A8QZJ5	106 (Crayfish)	Cytotoxic	[292]
Cytolysin Oshem 1	*Olindias sambaquiensis*	P0DQW8	-	Hemolytic	[293]
Cytolysin Oshem 2	*Olindias sambaquiensis*	P0DQW9	-	Hemolytic	[293]
Insulin-like peptide IlO1_i1	*Oulactis* sp.	P0DY19	-	K_v_1.2, K_v_1.3, K_v_3.1, K_v_11.1/hERG, Na_v_1.4.	[294]
Anthozoan neurotoxin	*Palythoa caribaeorum*	-	10–30 μM (Zebrafish, LD_100_)	-	[295,296]
*P. variabilis* ShK/Aurelin	*Palythoa variabilis*	-	15–20 μM (Zebrafish)	-	[296]
Nephrotoxin PsTX-115	*Phyllodiscus semoni*	P84851	-	-	[297]
PhcrTx1	*Phymanthus crucifer*	C0HJB1	-	ASIC, K_v_ channels	[298]
PpV9.4	*Physalia physalis*	-	-	-	[299]
PpV19.3	*Physalia physalis*	-	-	-	[299]
P3	*Physalia physalis*	-	-	Neurotoxic	[300]
P1	*Physalia physalis*	-	-	Neurotoxic	[301]
Physalitoxin	*Physalia physalis*	-	200 (Mice)	HC_50_: 0.5 nM (Mice RBCs)	[14]
PlKuz1	*Porites lutea*	-	-	K_v_1.3, Kallikrein	[302]
RmI	*Radianthus crispa*	-	-	HC_50_: 25 HU/mg (Mice RBCs)	[255]
RmII	*Radianthus crispa*	-	-	HC_50_: 20 HU/mg (Mice RBCs)	[255]
HC-18	*Radianthus crispa*	P0DY24	-	-	[268]
HC-19	*Radianthus crispa*	P0DY25	-	-	[268]
Rhizolysin	*Rhizostoma pulmo*	-	-	Hemolytic	[303]
Gigantoxin-I	*Stichodactyla gigantea*	Q76CA1	-	Paralytic	[84,85]
SHTX-V	*Stichodactyla haddoni*	B1B5J0	-	-	[100]
SmP90	*Stomolophus meleagris*	P0DUW7	-	-	[304]
Uc-1	*Urticina crassicornis*	P0CG44	-	Hemolytic	[305]
Tealiatoxin	*Urticina felina*	-	-	Hemolytic	[306]
Up-1	*Urticina piscivora*	P0C1G1	-	Hemolytic	[307,308]
ZoaKuz1	*Zoanthus natalensis*	-	-	-	[309]

ACE—Angiotensin-converting enzyme; HC_50_—concentration causing 50% hemolysis; LD_50_—median lethal dose; LD_100_—lethal dose (100%); RBC—red blood cells.

## Data Availability

Protein sequences of toxins identified from publicly available transcriptomes of Corallimorpharia and Antipatharia are provided in Table S8.

## Supplementary Materials

The following supporting information can be downloaded at: https://www.mdpi.com/article/10.3390/md24090306/s1, Figure S1: Completeness scores of the 5 assembled transcriptomes obtained from NCBI, assessed with BUSCO using the Metazoa dataset; Table S1: Attribution information for the photographs used in Figure 1; Table S2: Cnidaria toxins isolated from the ToxProt database; Table S3: Cnidaria toxins isolated from the literature; Table S4: Cnidaria species targeted in venom proteomics and/or proteo-transcriptomic and/or proteo-genomic studies; Table S5: Cnidaria species targeted in venom transcriptomic studies; Table S6: Expression patterns and functional roles of characterized toxins in *Nematostella vectensis*; Table S7: Geographic locations of cnidarian venom samples, collection years, target species and toxin families present in proteomic and transcriptomic studies; Table S8: Toxin profiles from the transcriptomes of Corallimorpharia and Antipatharia; File S1: Geographic coordinates of marine cnidarian sampling localities assigned to Marine Ecoregions of the World (MEOW) realms, grouped by subphylum; File S2: Toxin structures predicted by the ColabFold/AlphaFold2 server.
