# Supplementary material for: Cnidarian Venoms in the Omics Era: Toxin Discovery and Bioactive Potential
Source: Mar Drugs. 2026 Sep 1;24(9):306. doi: 10.3390/md24090306 (PMC13608440; doi:10.3390/md24090306)
Supplement: Supplementary file 1 [file marinedrugs-24-00306-s001.zip › Supplementary_Information.pdf]

Supplementary Information

Cnidarian Venoms in the Omics Era: Toxin Discovery and Bioactive Potential

R. Alexandre Barroso [1,2]; Agostinho Antunes [1,2]

[1] CIIMAR/CIMAR, Interdisciplinary Centre of Marine and Environmental Research, University of Porto, Terminal de Cruzeiros do Porto de Leixões, Av. General Norton de Matos s/n, 4450-208 Porto, Portugal; [2] Department of Biology, Faculty of Sciences, University of Porto, Rua do Campo Alegre, 4169-007 Porto, Portugal

Figure S1. Completeness scores of the 5 assembled transcriptomes obtained from NCBI, assessed with BUSCO using the Metazoa dataset.

Table S1. Attribution information for the photographs used in Figure 1**.**

Table S2. Cnidaria toxins isolated from the ToxProt database.

Table S3. Cnidaria toxins isolated from the literature.

Table S4. Cnidaria species targeted in venom proteomics and/or proteo-transcriptomic and/or proteo-genomic studies.

Table S5. Cnidaria species targeted in venom transcriptomic studies.

Table S6. Expression patterns and functional roles of characterized toxins in *Nematostella vectensis*.

Table S7: Geographic locations of cnidarian venom samples, collection years, target species and toxin families present in proteomic and transcriptomic studies.

Table S8: Toxin profiles from the transcriptomes of Corallimorpharia and Antipatharia.

File S1: Geographic coordinates of marine cnidarian sampling localities assigned to Marine Ecoregions of the World (MEOW) realms, grouped by subphylum.

File S2: Toxin structures predicted by the ColabFold/AlphaFold2 server.

References

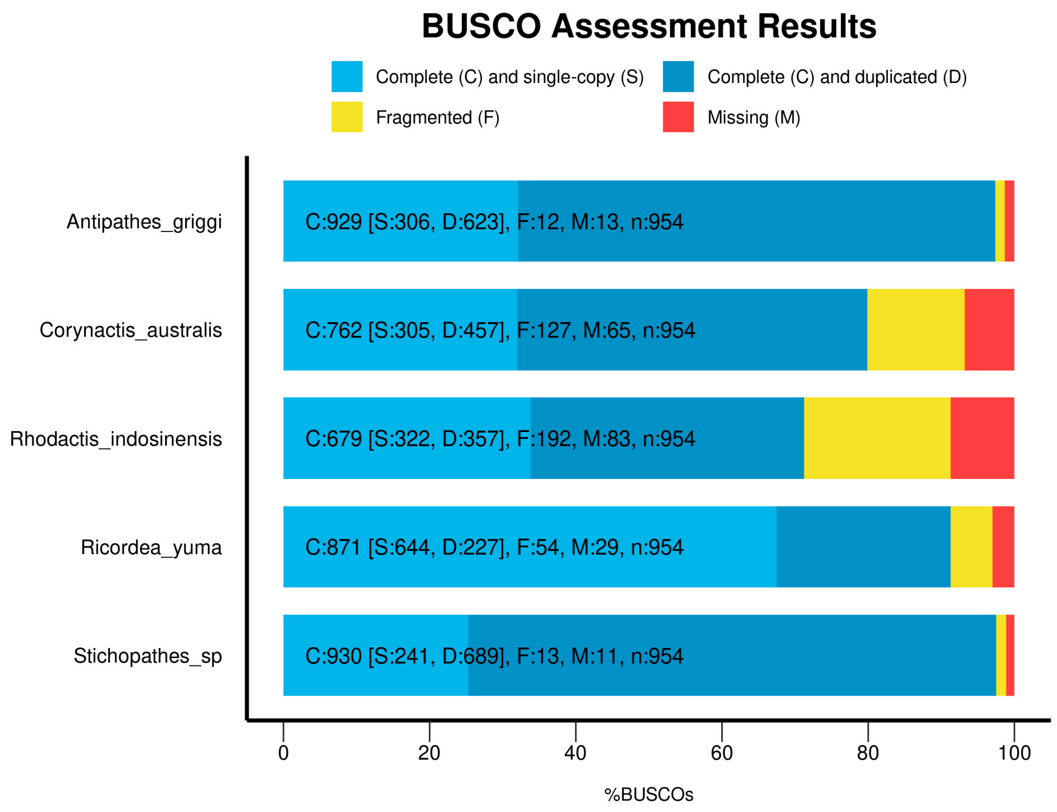

Figure S1. Completeness scores of the 5 assembled transcriptomes obtained from NCBI, assessed with BUSCO using the Metazoa dataset. Transcriptome completeness was evaluated using BUSCO against the Metazoa database (n = 954 BUSCOs). Stacked bars show the percentages of complete single-copy (S), complete duplicated (D), fragmented (F), and missing (M) BUSCOs for each assembly. The corresponding numbers of BUSCOs in each category are indicated within the bars. Transcriptomes of three Corallimorpharia species from BioProject PRJNA313487 (*Corynactis australis* (SRR3198576, SRR3198579, SRR3198580), *Rhodactis indosinensis* (SRR3201278-SRR3201281) and *Ricordea yuma* (SRR3201251-SRR3201253), and two Antipatharia species from BioProject PRJNA809900 (*Antipathes griggi* (SRR18121403) and *Stichopathes* sp. (SRR18121404), were downloaded from the NCBI Sequence Read Archive (SRA).

Table S1. Attribution information for the photographs used in Figure 1. These include the species depicted, photographer, source, Creative Commons license, and original iNaturalist photograph link. Panel a is a personal photograph by R. Alexandre Barroso; panels b–l were obtained from iNaturalist under the indicated Creative Commons licenses.

**Panel**	**Species**	**Photographer (iNaturalist username)**	**Source / License**	**Link**
a	*Actinia equina*	R. Alexandre Barroso	Personal photograph	n.a.
b	*Acropora palmata*	coralreefdreams	iNaturalist (Photo ID: 594711213), CC BY 4.0	inaturalist.org/photos/594711213
c	*Palythoa caribaeorum*	bertharris	iNaturalist (Photo ID: 595510132), CC BY-NC 4.0	inaturalist.org/photos/595510132
d	*Corynactis californica*	badgers	iNaturalist (Photo ID: 593886490), CC BY-NC 4.0	inaturalist.org/photos/593886490
e	*Pachycerianthus delwynae*	lawrencehylton	iNaturalist (Photo ID: 592221223), CC BY 4.0	inaturalist.org/photos/592221223
f	*Stichopathes luetkeni*	uma_dives	iNaturalist (Photo ID: 534379445), CC BY 4.0	inaturalist.org/photos/534379445
g	*Eunicella singularis*	frederic-andre	iNaturalist (Photo ID: 596184083), CC BY-NC 4.0	inaturalist.org/photos/596184083
h	*Ptilosarcus gurneyi*	milesuw	iNaturalist (Photo ID: 597357142), CC BY-NC 4.0	inaturalist.org/photos/597357142
i	*Carybdea marsupialis*	frederic-andre	iNaturalist (Photo ID: 551505759), CC BY-NC 4.0	inaturalist.org/photos/551505759
j	*Chrysaora fuscescens*	amaya-black	iNaturalist (Photo ID: 596980284), CC BY-NC 4.0	inaturalist.org/photos/596980284
k	*Physalia physalis*	chelseasea	iNaturalist (Photo ID: 526446309), CC BY 4.0	inaturalist.org/photos/526446309
l	*Calvadosia campanulata*	thomasmacro	iNaturalist (Photo ID: 693372735), CC BY-NC 4.0	inaturalist.org/photos/693372735

Table S2. Cnidaria toxins isolated from the ToxProt database. GenBank contain nucleotide sequences. The number of aa corresponds to the mature sequence (those complete, fragments or whose propeptide could not be identified are marked with *). n.a – not available.

UniProt	GenBank	Name	Other	Family	Species	Order	Targets	Nº aa	Location	Evidence	Year	3D structure	Biomedical Potencial	Ref
Q3C257	AB212067	Acrorhagin Ia	U-actitoxin-Aeq5b	Acrorhagin I	*Actinia equina*	Actiniaria	n.a.	52	Japan	Nucleotide	2005	n.a.	n.a.	[1]
Q3C258	AB212066	Acrorhagin I	U-actitoxin-Aeq5a	Acrorhagin I	*Actinia equina*	Actiniaria	n.a.	50	Japan	Protein	2005	6UX5	n.a.	[1]
Q3C255	AB212069	Acrorhagin IIa	U-actitoxin-Aeq6b	Acrorhagin II	*Actinia equina*	Actiniaria	n.a.	44	Japan	Nucleotide	2005	n.a.	n.a.	[1]
Q3C256	AB212068	Acrorhagin II	U-actitoxin-Aeq6a	Acrorhagin II	*Actinia equina*	Actiniaria	n.a.	44	Japan	Protein	2005	n.a.	n.a.	[1]
D2YZQ3	AB512460	Avt-II	DELTA-thalatoxin-Avl1b	Actinoporin	*Actineria villosa*	Actiniaria	n.a.	179	Japan	Nucleotide	2010	n.a.	n.a.	[2]
Q5R231	AB175824	Avt-I	DELTA-thalatoxin-Avl1a	Actinoporin	*Actineria villosa*	Actiniaria	Sphingomyelin	179	Japan	Protein	2005	n.a.	n.a.	[3]
P61914	U41661	Equinatoxin II	DELTA-actitoxin-Aeq1a	Actinoporin	*Actinia equina*	Actiniaria	Sphingomyelin	179	Northern Adriatic sea	Protein	1988	1IAZ	n.a.	[4]
Q93109	U51900	Equinatoxin V	DELTA-actitoxin-Aeq1b	Actinoporin	*Actinia equina*	Actiniaria	Sphingomyelin	179	n.a.	Nucleotide	1997	n.a.	n.a.	[5]
Q9Y1U9	AF057028	Equinatoxin IV	DELTA-actitoxin-Aeq1c	Actinoporin	*Actinia equina*	Actiniaria	Sphingomyelin	179	n.a.	Nucleotide	1999	n.a.	n.a.	[6]
P0C1H0	n.a.	Equinatoxin I'	n.a.	Actinoporin	*Actinia equina*	Actiniaria	n.a.	20*	Northern Adriatic sea	Protein	1988	n.a.	n.a.	[4]
P0C1H1	n.a.	Equinatoxin I''	n.a.	Actinoporin	*Actinia equina*	Actiniaria	n.a.	20*	Northern Adriatic sea	Protein	1988	n.a.	n.a.	[4]
P0C1H2	n.a.	Equinatoxin III	n.a.	Actinoporin	*Actinia equina*	Actiniaria	n.a.	20*	Northern Adriatic sea	Protein	1988	n.a.	n.a.	[4]
A0A515MEM9	MK936901	Fragaceatoxin E	DELTA-actitoxin-Afr1e	Actinoporin	*Actinia fragacea*	Actiniaria	n.a.	179	n.a.	Protein	2019	6K2G	n.a.	[7]
A0A515MEN7	MK936900	Fragaceatoxin B	DELTA-actitoxin-Afr1c	Actinoporin	*Actinia fragacea*	Actiniaria	n.a.	179	n.a.	Protein	2019	n.a.	n.a.	[7]
P0DUW8	n.a.	Fragaceatoxin A	DELTA-actitoxin-Afr1b	Actinoporin	*Actinia fragacea*	Actiniaria	n.a.	20	n.a.	Protein	2019	n.a.	n.a.	[7]

P0DUW9	n.a.	Fragaceatoxin D	DELTA-actitoxin-Afr1d	Actinoporin	*Actinia fragacea*	Actiniaria	n.a.	20	n.a.	Protein	2019	n.a.	n.a.	[7]
P30833	n.a.	Tenebrosin A	n.a.	Actinoporin	*Actinia tenebrosa*	Actiniaria	n.a.	20	Australia	Protein	1990	n.a.	n.a.	[8]
P30834	XM_031708028.1	Tenebrosin B	n.a.	Actinoporin	*Actinia tenebrosa*	Actiniaria	n.a.	179	Australia	Protein	1990	n.a.	n.a.	[8]
P61915	XM_031694881.1, XM_031694882.1	Tenebrosin C	DELTA-actitoxin-Ate1a	Actinoporin	*Actinia tenebrosa*	Actiniaria	n.a.	179	Australia	Protein	1990	n.a.	n.a.	[8]
C5NSL2	AB479475	Bandaporin	DELTA-actitoxin-Aas1a	Actinoporin	*Anthopleura asiatica*	Actiniaria	Sphingomyelin	179	Japan	Protein	2009	n.a.	n.a.	[9]
A0A345GPN1	MH204651	Nigrelysin	DELTA-actitoxin-Ani1a	Actinoporin	*Anthopleura nigrescens*	Actiniaria	Sphingomyelin	179	Costa Rica	Nucleotide	2019	n.a.	n.a.	[10]
A0A2Z5Z9H5	LC350099	Cjtox II	DELTA-actitoxin-Cja1b	Actinoporin	*Cribrinopsis japonica*	Actiniaria	Sphingomyelin	179	Japan	Nucleotide	2018	n.a.	n.a.	[11]
A0A2Z5Z9X0	LC350098	Cjtox I	DELTA-actitoxin-Cja1a	Actinoporin	*Cribrinopsis japonica*	Actiniaria	Sphingomyelin	178	Japan	Nucleotide	2018	n.a.	n.a.	[11]
P0DMX3	n.a.	EnT	n.a.	Actinoporin	*Entacmaea quadricolor*	Actiniaria	n.a.	40*	Jordan	Protein	2000	n.a.	n.a.	[12]
P0C1F8	n.a.	RTX-S II	DELTA-stichotoxin-Hcr4b	Actinoporin	*Radianthus crispa*	Actiniaria	Sphingomyelin	177	Seychelles	Protein	2004	n.a.	n.a.	[13]
P58691	AY855350	RTX-A	DELTA-stichotoxin-Hcr4a	Actinoporin	*Radianthus crispa*	Actiniaria	n.a.	175	Seychelles	Protein	2006	n.a.	Anticancer	[14]
P0DMX2	n.a.	HmT	DELTA-stichotoxin-Hmg2b	Actinoporin	*Radianthus magnifica*	Actiniaria	n.a.	117	Papua New Guinea	Protein	2000	n.a.	n.a.	[12]
P39088	n.a.	Cytolysin/DELTA-stichotoxin	n.a.	Actinoporin	*Radianthus magnifica*	Actiniaria	n.a.	54*	n.a.	Protein	1992	n.a.	n.a.	n.a.
P58689	n.a.	Magnificalysin I	n.a.	Actinoporin	*Radianthus magnifica*	Actiniaria	n.a.	20*	Singapore	Protein	1993	n.a.	n.a.	[15]
P58690	n.a.	Magnificalysin II	n.a.	Actinoporin	*Radianthus magnifica*	Actiniaria	n.a.	22*	Singapore	Protein	1993	n.a.	n.a.	[15]
Q9U6X1	AF170706	Magnificalysin III	DELTA-stichotoxin-Hmg2a	Actinoporin	*Radianthus magnifica*	Actiniaria	n.a.	177	Singapore	Protein	2000	n.a.	n.a.	[16]

Q5I2B1	AY861662	Or-G	DELTA-actitoxin-Oor1b	Actinoporin	*Oulactis orientalis*	Actiniaria	Sphingomyelin	173	Russia	Protein	2005	n.a.	n.a.	[17]
Q5I4B8	AY856481	Or-A	DELTA-actitoxin-Oor1a	Actinoporin	*Oulactis orientalis*	Actiniaria	Sphingomyelin	165	Russia	Protein	2005	n.a.	n.a.	[17]
P0DL55	AB063314	PsTX-20A	DELTA-alicitoxin-Pse1a	Actinoporin	*Phyllodiscus semoni*	Actiniaria	n.a.	179	Japan	Protein	2002	n.a.	n.a.	[18]
P0DL56	AB512461	Pst-I	DELTA-alicitoxin-Pse1b	Actinoporin	*Phyllodiscus semoni*	Actiniaria	n.a.	179	Japan	Nucleotide	2010	n.a.	n.a.	[2]
Q86FQ0	AY247033	Src-I	DELTA-sagatoxin-Srs1a	Actinoporin	*Cylista elegans*	Actiniaria	n.a.	178	China	Protein	2002	n.a.	n.a.	[19]
P07845	AJ005038	Sticholysin II	DELTA-stichotoxin-She4b	Actinoporin	*Stichodactyla helianthus*	Actiniaria	Sphingomyelin	175	n.a.	Protein	1983	1GWY	n.a.	[20]
P81662	AJ009931	Sticholysin I	DELTA-stichotoxin-She4a	Actinoporin	*Stichodactyla helianthus*	Actiniaria	Sphingomyelin	176	n.a.	Protein	2001	2KS4	n.a.	[21]
P0DMX4	n.a.	SmT-1/SmT-2	n.a.	Actinoporin	*Stichodactyla mertensii*	Actiniaria	n.a.	40	Papua New Guinea	Protein	2000	n.a.	n.a.	[12]
C9EIC7	GQ848199	Urticinatoxin	DELTA-actitoxin-Ucs1a	Actinoporin	*Urticina crassicornis*	Actiniaria	n.a.	179	n.a.	Nucleotide	2009	n.a.	n.a.	n.a.
B9W5G6	FM958450	Fragaceatoxin C	DELTA-actitoxin-Afr1a	Actinoporin	*Actinia fragacea*	Actiniaria	Sphingomyelin	179	Cantabrian Sea	Protein	2009	3LIM	Nanopore Technology	[22]
A0A8B7DWS6	XM_065800398.1	HALT-1	DELTA-hydritoxin-Hma1a	Actinoporin (HALT)	*Hydra vulgaris*	Hydrozoa	n.a.	186	n.a.	Protein	2014	7EKZ	n.a.	[23]
A0A8B6XDS4	XM_002153869.5	HALT-2	DELTA-hydritoxin-Hma1b	Actinoporin (HALT)	*Hydra vulgaris*	Hydrozoa	n.a.	203	n.a.	Nucleotide	2019	n.a.	n.a.	[24]
A0A8B6XS44	DN815714	HALT-3	DELTA-hydritoxin-Hma1c	Actinoporin (HALT)	*Hydra vulgaris*	Hydrozoa	n.a.	157	n.a.	Nucleotide	2019	n.a.	n.a.	[24]
A0A8B6XWY5	XM_068057649.1	HALT-5	DELTA-hydritoxin-Hma1e	Actinoporin (HALT)	*Hydra vulgaris*	Hydrozoa	n.a.	186	n.a.	Nucleotide	2019	n.a.	n.a.	[24]
A0A8B7DY61	XM_012707034.3	HALT-6	DELTA-hydritoxin-Hma1f	Actinoporin (HALT)	*Hydra vulgaris*	Hydrozoa	n.a.	186	n.a.	Nucleotide	2019	n.a.	n.a.	[24]

P0DQV6	XM_065828228.1	HALT-4	DELTA-hydritoxin-Hma1d	Actinoporin (HALT)	*Hydra vulgaris*	Hydrozoa	n.a.	302	n.a.	Nucleotide	2019	n.a.	n.a.	[24]
P0DQV7	CF778825	HALT-7	DELTA-hydritoxin-Hma1g	Actinoporin (HALT)	*Hydra vulgaris*	Hydrozoa	n.a.	187	n.a.	Nucleotide	2019	n.a.	n.a.	[24]
P86468	n.a.	Bcs III 15.09	n.a.	EGF domain peptide	*Bunodosoma caissarum*	Actiniaria	n.a.	45	n.a.	Protein	2010	n.a.	n.a.	n.a.
Q76CA1	AB110014	Gigantoxin I	OMEGA-stichotoxin-Sgt1a	EGF domain peptide	*Stichodactyla gigantea*	Actiniaria	EGF receptor	48	Japan	Protein	2003	n.a.	n.a.	[25]
B1B5J0	AB362571	SHTX 5	OMEGA-stichotoxin-Shd4a	EGF domain peptide	*Stichodactyla haddoni*	Actiniaria	n.a.	48	Japan	Nucleotide	2008	n.a.	n.a.	[26]
A0A0P0UTJ1	LC054037	Hau I	U-stichotoxin-Hau1a	Hau1a/HC18/HC19 family	*Heteractis aurora*	Actiniaria	n.a.	30	Japan	Protein	2024	n.a.	n.a.	[27]
P0DY24	n.a.	HC-18	U-stichotoxin-Hcr1a	Hau1a/HC18/HC19 family	*Radianthus crispa*	Actiniaria	n.a.	30	China	Nucleotide	2024	n.a.	n.a.	[28]
P0DY25	n.a.	HC-19	U-stichotoxin-Hcr1b	Hau1a/HC18/HC19 family	*Radianthus crispa*	Actiniaria	n.a.	32	China	Nucleotide	2024	n.a.	n.a.	[28]
Q52SK7	AY967764	Hydralysin	n.a.	Hydralysin	*Hydra vulgaris*	Hydrozoa	n.a.	244	n.a.	Nucleotide	2005	n.a.	n.a.	[29]
Q86LR2	AY178276	Hydralysin-1	n.a.	Hydralysin	*Hydra viridissima*	Hydrozoa	n.a.	244	n.a.	Protein	2003	n.a.	n.a.	[30]
Q564A5	AY655142	Hydralysin-2	n.a.	Hydralysin	*Hydra viridissima*	Hydrozoa	n.a.	244	n.a.	Protein	2005	n.a.	n.a.	[29]
Q52SK6	AY967765	Hydralysin-3	n.a.	Hydralysin	*Hydra viridissima*	Hydrozoa	n.a.	218	n.a.	Nucleotide	2005	n.a.	n.a.	[29]
P0DY19	LR700308	Insulin-like peptide IlO1_i1	n.a.	Insulin family	*Oulactis sp.*	Actiniaria	Kv1.2,Kv1.3, Kv3.1, Kv11.1/hERG, Nav1.4	115	Australia	Protein	2021	n.a.	n.a.	[31]
P0DUW6	n.a.	CcTX-1	n.a.	Jellyfish toxin family	*Cyanea capillata*	Scyphozoa	n.a.	243	Scotland	Protein	2011	n.a.	n.a.	[32]
A7L035	EF636902	CfTX-1	n.a.	Jellyfish toxin family Type I	*Chironex fleckeri*	Cubozoa	n.a.	456	Australia	Protein	2007	n.a.	n.a.	[33]
A7L036	EF636903	CfTX-2	n.a.	Jellyfish toxin family Type I	*Chironex fleckeri*	Cubozoa	n.a.	462	Australia	Protein	2007	n.a.	n.a.	[33]

P58762	AB045319	CqTX-A	n.a.	Jellyfish toxin family Type I	*Chiropsoides quadrigatus*	Cubozoa	n.a.	462	Japan	Protein	2002	n.a.	n.a.	[34]
Q9GNN8	AB036714	CaTX-A/CaTX-B	n.a.	Jellyfish toxin family Type II	*Alatina alata*	Cubozoa	n.a.	463	United States of America	Protein	2000	n.a.	n.a.	[35]
Q9GV72	AB015878	CrTX-A/CrTX-B	n.a.	Jellyfish toxin family Type II	*Carybdea rastonii*	Cubozoa	n.a.	450	Japan	Protein	2000	n.a.	n.a.	[36]
T1PQV6	JN695598	CfTX-B	n.a.	Jellyfish toxin family Type II	*Chironex fleckeri*	Cubozoa	n.a.	461	Australia	Protein	2014	n.a.	n.a.	[37]
T1PRE3	JN695597	CfTX-A	n.a.	Jellyfish toxin family Type II	*Chironex fleckeri*	Cubozoa	n.a.	454	Australia	Protein	2014	n.a.	n.a.	[37]
P0DY41	n.a.	ShKC10-Cpp1a	n.a.	KTx type I	*Calliactis polypus*	Actiniaria	n.a.	69	Australia	Protein	2023	n.a.	n.a.	[38]
Q76DT2	n.a.	AvTX-60A	DELTA-thalatoxin-Avl2a	MAC-PF	*Actineria villosa*	Actiniaria	n.a.	498	Japan	Protein	2004	n.a.	n.a.	[39]
P58911	AB063315	PsTX-60A	DELTA-alicitoxin-Pse2a	MAC-PF	*Phyllodiscus semoni*	Actiniaria	n.a.	501	Japan	Protein	2002	n.a.	n.a.	[40]
P58912	AB201429	PsTX-60B	DELTA-alicitoxin-Pse2b	MAC-PF	*Phyllodiscus semoni*	Actiniaria	n.a.	488	Japan	Protein	2004	n.a.	n.a.	[40]
A7RJ12	DS469513	NEP-3	n.a.	NEP3 family	*Nematostella vectensis*	Actiniaria	n.a.	355	na	Protein	2013	n.a.	n.a.	[41]
A7RJ13	DS469513	NEP-3-like	n.a.	NEP3 family	*Nematostella vectensis*	Actiniaria	n.a.	432	na	Nucleotide	2018	n.a.	n.a.	[42]
A7RNZ0	DS469524	NEP-4	n.a.	NEP3 family	*Nematostella vectensis*	Actiniaria	n.a.	370	na	Protein	2013	n.a.	n.a.	[41]
A7SXG9	DS469888	NEP-8	n.a.	NEP3 family	*Nematostella vectensis*	Actiniaria	n.a.	313	na	Protein	2013	n.a.	n.a.	[41]
E3P6S4	FJ418889	AdE-1	Delta-aiptatoxin-Adi1a	Other NaTx	*Exaiptasia diaphana*	Actiniaria	n.a.	44	Israel	Protein	2013	n.a.	Antiarrhythmic	[43]
P14531	S69403	Calitoxin I	Delta-hormotoxin-Cpt1b	Other NaTx	*Calliactis parasitica*	Actiniaria	n.a.	46	Italy	Protein	1989	n.a.	n.a.	[44]

P49127	S69399	Calitoxin II	Delta-hormotoxin-Cpt1a	Other NaTx	*Calliactis parasitica*	Actiniaria	n.a.	46	n.a.	Nucleotide	1994	n.a.	n.a.	[45]
P0DY39	n.a.	Calitoxin-Cpp1a	n.a.	Other NaTx	*Calliactis polypus*	Actiniaria	n.a.	48	Australia	Protein	2023	n.a.	n.a.	[38]
Q8WS88	AF347072	AcPLA2	n.a.	Phospholipase A2	*Calliactis palliata*	Actiniaria	n.a.	119	Norway	Nucleotide	2002	n.a.	n.a.	[46]
D2X8K2	GU046515	CgPLA2	n.a.	Phospholipase A2	*Condylactis gigantea*	Actiniaria	n.a.	119	Cuba	Protein	2010	n.a.	n.a.	[47]
P43318	n.a.	Toxic phospholipase A2	n.a.	Phospholipase A2	*Rhopilema nomadica*	Scyphozoa	n.a.	32*	n.a.	Protein	1995	n.a.	n.a.	[48]
A7LCJ2	EU003992	UcPLA2	n.a.	Phospholipase A2	*Urticina crassicornis*	Actiniaria	n.a.	104	Canada	Protein	2010	n.a.	n.a.	[49]
P0DY42	n.a.	PLA2-Cpp1a	n.a.	Phospholipase A2	*Calliactis polypus*	Actiniaria	n.a.	108	Australia	Protein	2023	n.a.	n.a.	[38]
P86780	n.a.	BcPLA21	n.a.	Phospholipase A2	*Bunodosoma caissarum*	Actiniaria	n.a.	39*	Brazil	Protein	2009	n.a.	Insulin Modulation	[50]
C0HK26	LT600337	Ueq 12-1	Tau-AnmTx Ueq 12-1	SCRiP	*Urticina eques*	Actiniaria	TRPA1	45	Norway	Protein	2017	5LAH	Analgesic Antimicrobial	[51]
P0DL61	FK725749	Avir_SCRiP	n.a.	SCRiP	*Anemonia viridis*	Actiniaria	n.a.	43	n.a.	Nucleotide	2015	n.a.	n.a.	[52]
C0H692	BK006536	Amil-SCRiP3	n.a.	SCRiP alpha subfamily	*Acropora millepora*	Scleractinia	n.a.	42	n.a.	Nucleotide	2009	n.a.	n.a.	[53]
C0H693	BK006537	Mcap-SCRiP1a	n.a.	SCRiP alpha subfamily	*Montipora capitata*	Scleractinia	n.a.	40	n.a.	Nucleotide	2009	n.a.	n.a.	[53]
C0H694	BK006538	Mcap-SCRiP1b	n.a.	SCRiP alpha subfamily	*Montipora capitata*	Scleractinia	n.a.	40	n.a.	Nucleotide	2009	n.a.	n.a.	[53]
B2ZG38	EU659816	Mfav-SCRiP8	n.a.	SCRiP beta subfamily	*Orbicella faveolata*	Scleractinia	n.a.	41	n.a.	Nucleotide	2009	n.a.	n.a.	[53]
C1KIY9	FJ842102	Mfav-SCRiP1	n.a.	SCRiP beta subfamily	*Orbicella faveolata*	Scleractinia	n.a.	42	n.a.	Nucleotide	2009	n.a.	n.a.	[53]
C1KIZ3	FJ842106	Mfav-SCRiP4	n.a.	SCRiP beta subfamily	*Orbicella faveolata*	Scleractinia	n.a.	50	n.a.	Nucleotide	2009	n.a.	n.a.	[53]
C1KIZ5	FJ842108	Mfav-SCRiP6	n.a.	SCRiP beta subfamily	*Orbicella faveolata*	Scleractinia	n.a.	50	n.a.	Nucleotide	2009	n.a.	n.a.	[53]
C0H691	BK006535	Amil-SCRiP2	n.a.	SCRiP delta subfamily	*Acropora millepora*	Scleractinia	n.a.	42	n.a.	Nucleotide	2009	n.a.	n.a.	[53]
P0DL60	FC835414	Msen-SCRiP	n.a.	SCRiP delta subfamily	*Metridium senile*	Actiniaria	n.a.	45	n.a.	Nucleotide	2015	n.a.	n.a.	[52]

A0A3P8MJV5	KY176766	Ate-SCRiP	n.a.	SCRiP delta subfamily	*Actinia tenebrosa*	Actiniaria	n.a.	60	n.a.	Nucleotide	2019	n.a.	n.a.	[54]
C0H690	BK006534	Amil-SCRiP1	n.a.	SCRiP gamma subfamily	*Acropora millepora*	Scleractinia	n.a.	42	n.a.	Nucleotide	2009	n.a.	n.a.	[53]
C1KIZ0	FJ842103	Mfav_SCRiP2	n.a.	SCRiP gamma subfamily	*Orbicella faveolata*	Scleractinia	n.a.	68*	n.a.	Nucleotide	2009	n.a.	n.a.	[53]
C1KIZ4	FJ842107	Mfav-SCRiP5	n.a.	SCRiP gamma subfamily	*Orbicella faveolata*	Scleractinia	n.a.	68*	n.a.	Nucleotide	2009	n.a.	n.a.	[53]
P0DMZ3	FK752567	Avtx-1	U-actitoxin-Avd8a	Sea anemone 8 toxin family	*Anemonia viridis*	Actiniaria	n.a.	43	France	Nucleotide	2011	n.a.	n.a.	[55]
P0DMZ4	FK728565	Avtx-2	U-actitoxin-Avd8b	Sea anemone 8 toxin family	*Anemonia viridis*	Actiniaria	n.a.	43	France	Nucleotide	2011	n.a.	n.a.	[55]
P0DMZ5	FK734835	Avtx-3	U-actitoxin-Avd8c	Sea anemone 8 toxin family	*Anemonia viridis*	Actiniaria	n.a.	43	France	Nucleotide	2011	n.a.	n.a.	[55]
P0DMZ6	FK736739	Avtx-4	U-actitoxin-Avd8d	Sea anemone 8 toxin family	*Anemonia viridis*	Actiniaria	n.a.	43	France	Nucleotide	2011	n.a.	n.a.	[55]
P0DMZ7	FK740713	Avtx-5	U-actitoxin-Avd8e	Sea anemone 8 toxin family	*Anemonia viridis*	Actiniaria	n.a.	41	France	Nucleotide	2011	n.a.	n.a.	[55]
A0A1R3S3A8	LT577947	Tau-AnmTX Ms 9a-1; AnmTx Ms 9a-2	n.a.	Sea anemone BBH family	*Metridium senile*	Actiniaria	rTRPA1	35, 27	Norway	Protein	2017	n.a.	Analgesic	[56]
R4ZCU1	HF562346	Pi-AnmTX Ugr 9a-1; AnmTX Ugr 9a-2; AnmTX Ugr 9a-3	Pi-actitoxin-Ugr1a; U-actitoxin-Ugr1b; U-actitoxin-Ugr1c	Sea anemone BBH family	*Urticina grebelnyi*	Actiniaria	hASIC3	29	Russia	Protein	2013	2LZO	Analgesic	[57]
P69929	AB180685	Am I	Delta-actitoxin-Amc1a	Sea anemone BBH family	*Antheopsis maculata*	Actiniaria	n.a.	27	Japan	Protein	2005	n.a.	n.a.	[58]
P86465	n.a.	Bcg 21.75	U-actitoxin-Bcg2a	Sea anemone BBH family	*Bunodosoma cangicum*	Actiniaria	n.a.	30	Brazil	Protein	2008	n.a.	n.a.	[59]

P86466	n.a.	Bcg 21.00	n.a.	Sea anemone BBH family	*Bunodosoma cangicum*	Actiniaria	n.a.	27	Brazil	Protein	2008	n.a.	n.a.	[59]
P86467	n.a.	Bcg 23.41	n.a.	Sea anemone BBH family	*Bunodosoma cangicum*	Actiniaria	n.a.	28	Brazil	Protein	2008	n.a.	n.a.	[59]
P0C7W7	n.a.	SHTX I; SHTX II	Kappa-stichotoxin-Shd1a/kappa-stichotoxin-Shd1b	Sea anemone BBH family	*Stichodactyla haddoni*	Actiniaria	Kv channels	28	Japan	Protein	2008	n.a.	n.a.	[26]
A0A0P0UTQ7	LC054039	Hau III	U-stichotoxin-Hau2b	Sea anemone BBH family	*Heteractis aurora*	Actiniaria	n.a.	28	Japan	Protein	2024	n.a.	n.a.	[27]
A0A0P0UTI6	LC054038	Hau II	U-stichotoxin-Hau2a	Sea anemone BBH family	*Heteractis aurora*	Actiniaria	n.a.	27	Japan	Protein	2024	n.a.	n.a.	[27]
C0HJB4	n.a.	n.a.	U-homostoxin-Hdu1a	Sea anemone BBH family	*Homostichanthus duerdeni*	Actiniaria	n.a.	29	Brazil	Protein	2013	n.a.	n.a.	[60]
P0DM22	n.a.	Ate1a	Kappa-actitoxin-Ate1a	Sea anemonne PHAB family	*Actinia tenebrosa*	Actiniaria	Kv1.1, Kv1.2, Kv1.3 Kv1.6, Shaker IR	17	n.a.	Protein	2018	6AZA	n.a.	[61]
P29187	n.a.	ShK	Kappa-stichotoxin-She3a	Type I KTx (1a subfamily)	*Stichodactyla helianthus*	Actiniaria	Kv1.1, Kv1.3, Kv1.4, Kv1.6, Kv1.2, Kv1.7, KCa3.1	35	Cuba	Protein	1995	1ROO	Antimicrobial; Immunomodulatory; Neuroprotective; Insulin Modulation	[62]
Q0EAE5	AB259113	AETX K	Kappa-actitoxin-Aer3a	Type I KTx (1a subfamily)	*Anemonia erythraea*	Actiniaria	Kv1.1, Kv1.2, Kv1.3	34	Japan	Protein	2006	7OD2	Epilepsy	[63]
O16846	U58107	HmK	Kappa-stichotoxin-Hmg1a	Type I KTx (1a subfamily)	*Radianthus magnifica*	Actiniaria	Kv1.1, Kv1.2, Kv1.5	35	n.a.	Protein	1997	n.a.	n.a.	[64]
P0DRH8	n.a.	ShKT-Ts1	Kappa-isophellitoxin-Tst1a	Type I KTx (1a subfamily)	*Telmatactis stephensoni*	Actiniaria	n.a.	36	Australia	Protein	2022	8SED	n.a.	[65]
E2S064	AB595207	κ1.3-TLTX-Ca1a	Kappa-thalatoxin-Cad2a	Type I KTx (1a subfamily)	*Cryptodendrum adhaesivum*	Actiniaria	n.a.	36	n.a.	Nucleotide	2010	n.a.	n.a.	[66]

E2S065	AB595208	κ1.3-TLTX-Hh1a	Kappa-thalatoxin-Hhe2a	Type I KTx (1a subfamily)	*Heterodactyla hemprichii*	Actiniaria	n.a.	36	n.a.	Nucleotide	2010	n.a.	n.a.	[66]
E2S061	AB595204	κ1.3-SHTX-Sg1a	Kappa-stichotoxin-Sgt4a	Type I KTx (1a subfamily)	*Stichodactyla gigantea*	Actiniaria	n.a.	36	n.a.	Nucleotide	2010	n.a.	n.a.	[66]
E2S062	AB595205	κ1.3-SHTX-Sha1a	Kappa-stichotoxin-Shd5a	Type I KTx (1a subfamily)	*Stichodactyla haddoni*	Actiniaria	n.a.	36	n.a.	Nucleotide	2010	n.a.	n.a.	[66]
E2S063	AB595206	κ1.3-SHTX-Sm1a	Kappa-stichotoxin-Smt1a	Type I KTx (1a subfamily)	*Stichodactyla mertensii*	Actiniaria	n.a.	36	n.a.	Nucleotide	2010	n.a.	n.a.	[66]
E2S066	AB595209	κ1.3-TLTX-Ta1a	Kappa-thalatoxin-Tas2a	Type I KTx (1a subfamily)	*Thalassianthus aster*	Actiniaria	n.a.	36	n.a.	Nucleotide	2010	n.a.	n.a.	[66]
P29186	n.a.	BgK	Kappa-actitoxin-Bgr1a	Type I KTx (1b subfamily)	*Bunodosoma granuliferum*	Actiniaria	Kv1.1, Kv1.2, Kv1.3, Kv1.6, KCa3.1	36	Cuba	Protein	1993	1BGK	Immunomodulatory	[67]
P81897	n.a.	Aek	Kappa-actitoxin-Aeq4a	Type I KTx (1b subfamily)	*Actinia equina*	Actiniaria	n.a.	36	Japan	Protein	1998	n.a.	n.a.	[68]
Q9TWG1	n.a.	Kaliseptine/AsKS	Kappa-actitoxin-Avd6a	Type I KTx (1b subfamily)	*Anemonia sulcata*	Actiniaria	Kv1.2	36	n.a.	Protein	1995	n.a.	n.a.	[69]
P0DN05	FK736704	Avtx-11	U-actitoxin-Avd11a	Type I KTx (1b subfamily)	*Anemonia viridis*	Actiniaria	n.a.	36	France	Nucleotide	2011	n.a.	n.a.	[55]
P0DN00	FK724096	Avtx-6	U-actitoxin-Avd9a	Type I KTx (1b subfamily)	*Anemonia viridis*	Actiniaria	n.a.	38	France	Nucleotide	2011	n.a.	n.a.	[55]
P0DN01	FK756695	Avtx-7	U-actitoxin-Avd9b	Type I KTx (1b subfamily)	*Anemonia viridis*	Actiniaria	n.a.	38	France	Nucleotide	2011	n.a.	n.a.	[55]
P0DN02	FK755121	Avtx-8	U-actitoxin-Avd9c	Type I KTx (1b subfamily)	*Anemonia viridis*	Actiniaria	n.a.	38	France	Nucleotide	2011	n.a.	n.a.	[55]
P0DN03	FK747792	Avtx-8	U-actitoxin-Avd9d	Type I KTx (1b subfamily)	*Anemonia viridis*	Actiniaria	n.a.	38	France	Nucleotide	2011	n.a.	n.a.	[55]
P0DN04	FK743341	Avtx-10	U-actitoxin-Avd10a	Type I KTx (1b subfamily)	*Anemonia viridis*	Actiniaria	n.a.	38	France	Nucleotide	2011	n.a.	n.a.	[55]

C0HJC2	n.a.	BcsTx1	Kappa-actitoxin-Bcs3a	Type I KTx (1b subfamily)	*Bunodosoma caissarum*	Actiniaria	Kv1.1, Kv1.2, Kv1.3, Kv1.6, Shaker	37	Brazil	Protein	2013	n.a.	n.a.	[70]
C0HJC3	n.a.	BcsTx2	Kappa-actitoxin-Bcs3b	Type I KTx (1b subfamily)	*Bunodosoma caissarum*	Actiniaria	Kv1.1, Kv1.2, Kv1.3, Kv1.6, Shaker	37	Brazil	Protein	2013	n.a.	n.a.	[70]
P11495	n.a.	Metridin	U-metritoxin-Msn1a	Type I KTx (1b subfamily)	*Metridium senile*	Actiniaria	n.a.	36	n.a.	Protein	1987	n.a.	n.a.	[71]
A0A330KUG5	LT985997	OspTx2b	U-actitoxin-Oulsp2	Type I KTx (1b subfamily)	*Oulactis sp.*	Actiniaria	n.a.	36	n.a.	Protein	2018	6BUC	n.a.	[72]
A0A330KW27	LT985996	OspTx2a	U-actitoxin-Oulsp1	Type I KTx (1b subfamily)	*Oulactis sp.*	Actiniaria	Kv1.2, Kv1.6	36	n.a.	Protein	2018	6CKD	n.a.	[73]
P01528	n.a.	ATX II	Delta-actitoxin-Avd1c	Type I NaTx	*Anemonia sulcata*	Actiniaria	Nav1.1-Nav1.6, Para/TipE	47	Italy	Protein	1975	n.a.	n.a.	[74]
P0C1F0	n.a.	APE 1-1	Delta-actitoxin-Ael1b	Type I NaTx	*Anthopleura elegantissima*	Actiniaria	n.a.	47	n.a.	Protein	2001	n.a.	Antiarrhythmic	[75]
P01532	n.a.	Anthopleurin-C/ APE 2-1	Delta-actitoxin-Ael1a	Type I NaTx	*Anthopleura elegantissima*	Actiniaria	n.a.	47	n.a.	Protein	1981	n.a.	Antiarrhythmic	[76]
P10453	n.a.	AFT-I	Delta-actitoxin-Afv1a	Type I NaTx	*Anthopleura fuscoviridis*	Actiniaria	n.a.	47	Japan	Protein	1987	n.a.	Antiarrhythmic	[77]
P10454	n.a.	AFT-II	Delta-actitoxin-Afv1b	Type I NaTx	*Anthopleura fuscoviridis*	Actiniaria	Nav1.1-Nav1.6	48	Japan	Protein	1987	n.a.	Antiarrhythmic	[77]
P0C5F5	n.a.	Hk7a	Delta-actitoxin-Aspp1b	Type I NaTx	*Anthopleura sp. (strain 'Zhanjiang')*	Actiniaria	n.a.	47	China	Nucleotide	2004	n.a.	Antiarrhythmic	[78]
P0C5F4	n.a.	Hk2a	Delta-actitoxin-Aspp1a	Type I NaTx	*Anthopleura sp. (strain 'Zhanjiang')*	Actiniaria	n.a.	47	China	Nucleotide	2003	n.a.	Antiarrhythmic	[79]
P0C5F6	n.a.	Hk8a	Delta-actitoxin-Aspp1c	Type I NaTx	*Anthopleura sp. (strain 'Zhanjiang')*	Actiniaria	n.a.	47	China	Nucleotide	2004	n.a.	Antiarrhythmic	[78]

P0C5F7	n.a.	Hk16a	Delta-actitoxin-Aspp1d	Type I NaTx	*Anthopleura sp. (strain 'Zhanjiang')*	Actiniaria	n.a.	47	China	Nucleotide	2004	n.a.	Antiarrhythmic	[78]
P01530	n.a.	Anthopleurin-A	Delta-actitoxin-Axm1a	Type I NaTx	*Anthopleura xanthogrammica*	Actiniaria	Nav1.5	49	n.a.	Protein	1977	1AHL	Antiarrhythmic	[80]
P01531	M90675	Anthopleurin-B	Delta-actitoxin-Axm1b	Type I NaTx	*Anthopleura xanthogrammica*	Actiniaria	Nav1.5	49	n.a.	Protein	1985	1APF	Antiarrhythmic	[81]
P0C1F5	n.a.	Bg III	Delta-actitoxin-Bgr2b	Type I NaTx	*Bunodosoma granuliferum*	Actiniaria	n.a.	48	Gulf of Mexico	Protein	1994	n.a.	Insecticides	[82]
P0C1F4	n.a.	Bg II	Delta-actitoxin-Bgr2a	Type I NaTx	*Bunodosoma granuliferum*	Actiniaria	para/TipE, Nav1.2, Nav1.4, Nav1.5	48	Gulf of Mexico	Protein	1994	n.a.	Insecticides	[82]
P0DL49	EU124447	Av2; Av2-1	Delta-actitoxin-Avd1c 1	Type I NaTx	*Anemonia viridis*	Actiniaria	n.a.	47	n.a.	Protein	2006	n.a.	n.a.	[83]
B1NWU2	EU124479	Ae2-1	Delta-actitoxin-Aeq2b 1	Type I NaTx	*Actinia equina*	Actiniaria	n.a.	54	Israel	Nucleotide	2008	n.a.	n.a.	[83]
B1NWU3	EU124480	Ae2-2	Delta-actitoxin-Aeq2b 2	Type I NaTx	*Actinia equina*	Actiniaria	n.a.	54	Israel	Nucleotide	2008	n.a.	n.a.	[83]
B1NWU4	EU124481	Ae2-3	Delta-actitoxin-Aeq2b 3	Type I NaTx	*Actinia equina*	Actiniaria	n.a.	54	Israel	Nucleotide	2008	n.a.	n.a.	[83]
B1NWU5	EU124482	Ae3-1	Delta-actitoxin-Aeq2c	Type I NaTx	*Actinia equina*	Actiniaria	n.a.	54	Israel	Nucleotide	2008	n.a.	n.a.	[83]
B1NWU6	EU124483	Ae4-1	Delta-actitoxin-Aeq2d	Type I NaTx	*Actinia equina*	Actiniaria	n.a.	54	Israel	Nucleotide	2008	n.a.	n.a.	[83]
Q9NJQ2	AF130344	Ae1	Delta-actitoxin-Aeq2a	Type I NaTx	*Actinia equina*	Actiniaria	n.a.	54	Japan	Protein	1996	n.a.	n.a.	[84]
P69943	n.a.	AETX I	Delta-actitoxin-Aer1a	Type I NaTx	*Anemonia erythraea*	Actiniaria	n.a.	47	Japan	Protein	1997	n.a.	n.a.	[85]
P01533	n.a.	ATX I	Delta-actitoxin-Avd1a	Type I NaTx	*Anemonia sulcata*	Actiniaria	n.a.	46	Italy	Protein	1975	1ATX	n.a.	[74]

P01529	n.a.	ATX V	Delta-actitoxin-Avd1d	Type I NaTx	*Anemonia sulcata*	Actiniaria	n.a.	46	n.a.	Protein	1982	n.a.	n.a.	[86]
B1NWT3	EU124470	Av6	U-actitoxin-Avd1f	Type I NaTx	*Anemonia viridis*	Actiniaria	n.a.	38	Israel	Nucleotide	2008	n.a.	n.a.	[83]
B1NWT4	EU124471	Av8	U-actitoxin-Avd7a	Type I NaTx	*Anemonia viridis*	Actiniaria	n.a.	41	Israel	Nucleotide	2008	n.a.	n.a.	[83]
B1NWT5	EU124472	Av9	U-actitoxin-Avd1g	Type I NaTx	*Anemonia viridis*	Actiniaria	n.a.	49	Israel	Nucleotide	2008	n.a.	n.a.	[83]
B1NWT7	EU124474	Toxin 2c2/Toxin 2c3	Delta-actitoxin-Avd1c 4	Type I NaTx	*Anemonia viridis*	Actiniaria	n.a.	47	Israel	Nucleotide	2008	n.a.	n.a.	[83]
P0DL50	EU124448	Av2-2	Delta-actitoxin-Avd1c 2	Type I NaTx	*Anemonia viridis*	Actiniaria	n.a.	47	n.a.	Protein	2006	n.a.	n.a.	[83]
P0DL51	EU124449	Av2-3	Delta-actitoxin-Avd1c 3	Type I NaTx	*Anemonia viridis*	Actiniaria	n.a.	47	n.a.	Protein	2006	n.a.	n.a.	[83]
P0DL52	EU124450	Av 2-4	Delta-actitoxin-Avd1e 1	Type I NaTx	*Anemonia viridis*	Actiniaria	n.a.	47	n.a.	Protein	2006	n.a.	n.a.	[83]
P0DL53	EU124451	Av 2-5	Delta-actitoxin-Avd1e 2	Type I NaTx	*Anemonia viridis*	Actiniaria	n.a.	47	n.a.	Protein	2006	n.a.	n.a.	[83]
P0DL54	EU124452	Av 2-6	Delta-actitoxin-Avd1e 3	Type I NaTx	*Anemonia viridis*	Actiniaria	n.a.	47	n.a.	Nucleotide	2006	n.a.	n.a.	[83]
P0DMZ1	FK754096	Neurotoxin 1-1	Delta-actitoxin-Avd1h	Type I NaTx	*Anemonia viridis*	Actiniaria	n.a.	46	France	Nucleotide	2011	n.a.	n.a.	[55]
P69928	AB180687	Am III	Delta-actitoxin-Amc3a	Type I NaTx	*Antheopsis maculata*	Actiniaria	n.a.	47	Japan	Protein	2005	n.a.	n.a.	[58]
P0C1F3	n.a.	APE 2-2	Delta-actitoxin-Ael1d	Type I NaTx	*Anthopleura elegantissima*	Actiniaria	n.a.	47	n.a.	Protein	2001	n.a.	n.a.	[75]
P0C1F1	n.a.	APE 1-2	Delta-actitoxin-Ael1c	Type I NaTx	*Anthopleura elegantissima*	Actiniaria	n.a.	47	n.a.	Protein	2001	n.a.	n.a.	[75]
P0C5F8	n.a.	Toxin PCR1	Delta-actitoxin-Axm1c	Type I NaTx	*Anthopleura xanthogram mica*	Actiniaria	n.a.	47	n.a.	Nucleotide	1998	n.a.	n.a.	[87]

P0C5F9	n.a.	Toxin PCR2	Delta-actitoxin-Axm1e	Type I NaTx	*Anthopleura xanthogrammica*	Actiniaria	n.a.	47	n.a.	Nucleotide	1998	n.a.	n.a.	[87]
P0C5G1	n.a.	Toxin PCR4	Delta-actitoxin-Axm1f	Type I NaTx	*Anthopleura xanthogrammica*	Actiniaria	n.a.	47	n.a.	Nucleotide	1998	n.a.	n.a.	[87]
P0C5G3	n.a.	Toxin PCR6	Delta-actitoxin-Axm1h	Type I NaTx	*Anthopleura xanthogrammica*	Actiniaria	n.a.	49	n.a.	Nucleotide	1998	n.a.	n.a.	[87]
P0C5G0	n.a.	Toxin PCR3	Delta-actitoxin-Axm1d	Type I NaTx	*Anthopleura xanthogrammica*	Actiniaria	n.a.	47	n.a.	Nucleotide	1998	n.a.	n.a.	[87]
Q7M425	n.a.	Bc-III	Delta-actitoxin-Bcs1a	Type I NaTx	*Bunodosoma caissarum*	Actiniaria	Nav1.1-Nav1.6	48	Brazil	Protein	1993	n.a.	n.a.	[88]
P0C7P9	n.a.	Cangitoxin II	Delta-actitoxin-Bcg1b	Type I NaTx	*Bunodosoma cangicum*	Actiniaria	Nav1.1-Nav1.6	48	Brazil	Protein	2008	n.a.	n.a.	[89]
P0C7Q0	n.a.	Cangitoxin III	Delta-actitoxin-Bcg1c	Type I NaTx	*Bunodosoma cangicum*	Actiniaria	Nav1.1	48	Brazil	Protein	2008	n.a.	n.a.	[89]
P86459	n.a.	Bcg 30.24	Delta-actitoxin-Bcg1d	Type I NaTx	*Bunodosoma cangicum*	Actiniaria	n.a.	47	Brazil	Protein	2008	n.a.	n.a.	[59]
P82803	n.a.	Cangitoxin I	Delta-actitoxin-Bcg1a	Type I NaTx	*Bunodosoma cangicum*	Actiniaria	n.a.	48	Brazil	Protein	2005	n.a.	n.a.	[90]
P0DTR3	n.a.	Del-ta-AITX-Bca1a	Delta-actitoxin-Bca1a	Type I NaTx	*Bunodosoma capense*	Actiniaria	n.a.	43	South Africa	Protein	2019	n.a.	n.a.	[91]
P0DY38	n.a.	NaTx-I	n.a.	Type I NaTx	*Calliactis polypus*	Actiniaria	n.a.	44	Australia	Protein	2023	n.a.	n.a.	[38]
P0C280	n.a.	Cgna	Delta-actitoxin-Cgg1a	Type I NaTx	*Condylactis gigantea*	Actiniaria	n.a.	47	Caribbean Sea	Protein	1995	2H9X	n.a.	[92]
P0CH42	n.a.	Cp I	Delta-actitoxin-Cgg1b	Type I NaTx	*Condylactis gigantea*	Actiniaria	n.a.	47	Caribbean Sea	Protein	1995	n.a.	n.a.	[92]
P0DPE5	n.a.	AnmTx Cj 1c-1	Toxin AnmTx Cj 1c-1	Type I NaTx	*Epiactis japonica*	Actiniaria	n.a.	54	Russia	Nucleotide	2017	n.a.	n.a.	[93]
A0A0N7KBN8	LC054040	Hau IV	U-stichotoxin-Hau3a	Type I NaTx	*Heteractis aurora*	Actiniaria	n.a.	49	Japan	Protein	2024	n.a.	n.a.	[27]

P0C5G5	n.a.	Rc-I	Delta-stichotoxin-Hcr3a	Type I NaTx	*Radianthus crispa*	Actiniaria	n.a.	47	n.a.	Protein	1996	n.a.	n.a.	[94]
Q76CA3	AB110012	Gigantoxin II	Delta-stichotoxin-Sgt2a	Type I NaTx	*Stichodactyla gigantea*	Actiniaria	n.a.	44	Japan	Protein	2003	n.a.	n.a.	[25]
B2G331	AM933240	APHC1	TauPI-stichotoxin-Hcr2b	Type II KTx	*Radianthus crispa*	Actiniaria	TRPV1, Trypsin, Chymotrypsin	56	Seychelles	Protein	2013	n.a.	Analgesic	[95]
C0HJF4	n.a.	APHC2	TauPI-stichotoxin-Hcr2c	Type II KTx	*Radianthus crispa*	Actiniaria	Trypsin, Chymotrypsin	56	n.a.	Protein	2009	n.a.	Analgesic	[96]
C0HJF3	n.a.	APHC3	TauPI-stichotoxin-Hcr2d	Type II KTx	*Radianthus crispa*	Actiniaria	TRPV1, Trypsin, Chymotrypsin	56	n.a.	Protein	2009	n.a.	Analgesic	[96]
P0DV03	n.a.	HCGS1.10	PI-stichotoxin-Hcr2m	Type II KTx	*Radianthus crispa*	Actiniaria	Trypsin	56	n.a.	Nucleotide	2012	n.a.	Analgesic; Neuroprotective; Antioxidant	[97]
A0A6B7FBD3	MH249934	HCIQ2c1	PI-stichotoxin-Hcr2i	Type II KTx	*Radianthus crispa*	Actiniaria	TRPA1, Trypsin, α-chymotrypsin, Kallikrein, Elastase, Cathepsin G	58	Vietnam	Protein	2020	9IJW	Analgesic; Neuroprotective; Antioxidant	[98]
P0DL86	KX900498	HCRG21	PI-stichotoxin-Hcr2h	Type II KTx	*Radianthus crispa*	Actiniaria	TRPV1, Trypsin, α-Chymotrypsin	56	n.a.	Protein	2016	n.a.	Analgesic; Neuroprotective; Antioxidant	[99]
P0DV02	n.a.	RmIn II	PI-stichotoxin-Hcr2l	Type II KTx	*Radianthus crispa*	Actiniaria	Trypsin, Chymotrypsin, Histamine	15	Seychelles	Protein	2007	n.a.	Anti-histaminic	[100]
P0DV07	n.a.	RmIn I	PI-stichotoxin-Hcr2k	Type II KTx	*Radianthus crispa*	Actiniaria	Trypsin, Chymotrypsin, Histamine	15	Seychelles	Protein	2007	n.a.	Anti-histaminic	[100]
P0DV04	n.a.	HCGS1.19	PI-stichotoxin-Hcr2n	Type II KTx	*Radianthus crispa*	Actiniaria	Trypsin, Histamine	56	n.a.	Nucleotide	2012	n.a.	Anti-histaminic; Analgesic; Antioxidant	[97]

P0DV06	n.a.	HCGS1.36	PI-stichotoxin-Hcr2p	Type II KTx	*Radianthus crispa*	Actiniaria	Trypsin, Histamine	56	n.a.	Nucleotide	2012	n.a.	Anti-histaminic; Analgesic; Neuroprotective; Antioxidant	[97]
P0DV05	n.a.	HCGS1.20	PI-stichotoxin-Hcr2o	Type II KTx	*Radianthus crispa*	Actiniaria	Trypsin, Histamine	56	n.a.	Nucleotide	2012	n.a.	Anti-histaminic; Analgesic; Neuroprotective; Antioxidant	[97]
C0HJU6	n.a.	HCRG1	PI-stichotoxin-Hcr2f	Type II KTx	*Radianthus crispa*	Actiniaria	Kv1.1, Kv1.2, Kv1.3, Kv1.6, Shaker, Trypsin, α-Chymotrypsin	56	Seychelles	Protein	2015	n.a.	Anti-inflammatory; Antioxidant	[101]
C0HJU7	n.a.	HCRG2	PI-stichotoxin-Hcr2g	Type II KTx	*Radianthus crispa*	Actiniaria	Kv1.1, Kv1.2, Kv1.3, Kv1.6, Shaker, Trypsin, α-Chymotrypsin	56	Seychelles	Protein	2015	n.a.	Anti-inflammatory; Antioxidant	[101]
P0DN15	FK727748	AsKC11	U-actitoxin-Avd3n	Type II KTx	*Anemonia viridis*	Actiniaria	GIRK1/2, Kv1.6	58	France	Protein	2011	n.a.	Modulators of Key Neuronal Channels	[55]
P0DMW6	n.a.	AEPI-I	PI-actitoxin-Aeq3a	Type II KTx	*Actinia equina*	Actiniaria	n.a.	59	Japan	Protein	1997	n.a.	n.a.	[102]
P0DMW7	n.a.	AEPI-II	PI-actitoxin-Aeq3b	Type II KTx	*Actinia equina*	Actiniaria	n.a.	59	Japan	Protein	1997	n.a.	n.a.	[102]
P0DMW8	n.a.	AEPI-III	n.a.	Type II KTx	*Actinia equina*	Actiniaria	n.a.	35	Japan	Protein	1997	n.a.	n.a.	[102]
P0DMW9	n.a.	AEPI-IV	n.a.	Type II KTx	*Actinia equina*	Actiniaria	n.a.	37	Japan	Protein	1997	n.a.	n.a.	[102]
A0A6P8HC43	XM_031694215	ATPI-I and ATPI-II	n.a.	Type II KTx	*Actinia tenebrosa*	Actiniaria	Trypsin, α-chymotrypti	58, 60	Australia	Protein	2019	n.a.	n.a.	[103]

							n, kallikreins							
Q9TWG0	n.a.	Kalicludine-1 / AsKC1	KappaPI-actitoxin-Avd3b	Type II KTx	*Anemonia sulcata*	Actiniaria	Kv1.2, trypsin	58	n.a.	Protein	1995	n.a.	n.a.	[69]
Q9TWF9	n.a.	Kalicludine-2 / AsKC2	KappaPI-actitoxin-Avd3c	Type II KTx	*Anemonia sulcata*	Actiniaria	Kv1.2, trypsin	58	n.a.	Protein	1995	n.a.	n.a.	[69]
Q9TWF8	n.a.	Kalicludine-3 / AsKC3	KappaPI-actitoxin-Avd3d	Type II KTx	*Anemonia sulcata*	Actiniaria	Kv1.2, trypsin	59	n.a.	Protein	1995	n.a.	n.a.	[69]
P0DN10	FK753133	AsKC6	U-actitoxin-Avd3i	Type II KTx	*Anemonia viridis*	Actiniaria	n.a.	45	France	Nucleotide	2011	n.a.	n.a.	[55]
P0DN14	FK752628	AsKC10	U-actitoxin-Avd3m	Type II KTx	*Anemonia viridis*	Actiniaria	n.a.	45	France	Nucleotide	2011	n.a.	n.a.	[55]
P0DN16	FK749190	AsKC12	U-actitoxin-Avd3o	Type II KTx	*Anemonia viridis*	Actiniaria	n.a.	58	France	Nucleotide	2011	n.a.	n.a.	[55]
P0DN17	FK753478	AsKC13	U-actitoxin-Avd3p	Type II KTx	*Anemonia viridis*	Actiniaria	n.a.	45	France	Nucleotide	2011	n.a.	n.a.	[55]
P0DN18	FK758844	AsKC14	U-actitoxin-Avd3q	Type II KTx	*Anemonia viridis*	Actiniaria	n.a.	45	France	Nucleotide	2011	n.a.	n.a.	[55]
P0DN19	FK741437	AsKC15	U-actitoxin-Avd3r	Type II KTx	*Anemonia viridis*	Actiniaria	n.a.	45	France	Nucleotide	2011	n.a.	n.a.	[55]
P0DN20	FK744640	AsKC16	U-actitoxin-Avd3s	Type II KTx	*Anemonia viridis*	Actiniaria	n.a.	45	France	Nucleotide	2011	n.a.	n.a.	[55]
P0DN06	FK756625	AsKC1a	U-actitoxin-Avd3f	Type II KTx	*Anemonia viridis*	Actiniaria	n.a.	45	France	Nucleotide	2011	n.a.	n.a.	[55]
P0DN07	FK756159	AsKC4	U-actitoxin-Avd3g	Type II KTx	*Anemonia viridis*	Actiniaria	n.a.	45	France	Nucleotide	2011	n.a.	n.a.	[55]

P0DN09	FK731360	AsKC5	U-actitoxin-Avd3h	Type II KTx	*Anemonia viridis*	Actiniaria	n.a.	45	France	Nucleotide	2011	n.a.	n.a.	[55]
P0DN11	FK740712	AsKC7	U-actitoxin-Avd3j	Type II KTx	*Anemonia viridis*	Actiniaria	n.a.	45	France	Nucleotide	2011	n.a.	n.a.	[55]
P0DN12	FK722334	AsKC8	U-actitoxin-Avd3k	Type II KTx	*Anemonia viridis*	Actiniaria	n.a.	45	France	Nucleotide	2011	n.a.	n.a.	[55]
P0DN13	FK731883	AsKC9	U-actitoxin-Avd3l	Type II KTx	*Anemonia viridis*	Actiniaria	n.a.	45	France	Nucleotide	2011	n.a.	n.a.	[55]
P86862	n.a.	APEKTx1	KappaPI-actitoxin-Ael3a	Type II KTx	*Anthopleura elegantissima*	Actiniaria	Kv1.1	65	n.a.	Protein	2011	n.a.	n.a.	[104]
P0DQQ9	n.a.	PcKuz1	PI-sphenopitoxin-Pc1a	Type II KTx	*Palythoa caribaeorum*	Zoantharia	Trypsin, elastase	51	Brazil	Nucleotide	2018	n.a.	n.a.	[105]
B1B5I8	AB362569	SHTX III	KappaPI-stichotoxin-Shd2a	Type II KTx	*Stichodactyla haddoni*	Actiniaria	n.a.	54	Japan	Protein	2008	n.a.	n.a.	[26]
A0A3G2FQK2	MG788754	IQ-peptide / HMIQ3c1	PI-stichotoxin-Hmg3d	Type II KTx	*Radianthus magnifica*	Actiniaria	Trypsin, α-Chymotrypsin, kallikrein, neutrophil elastase	58	Seychelles	Protein	2018	n.a.	Neuroprotective	[106]
P0DQR1	n.a.	PcKuz3	PI-sphenopitoxin-Pc1c	Type II KTx	*Palythoa caribaeorum*	Zoantharia	Trypsin, elastase	51	Brazil	Nucleotide	2018	n.a.	Neuroprotective	[105]
A0A6B7FEJ3	MH249938	HCIQ1c9	PI-stichotoxin-Hcr2k	Type II KTx	*Radianthus crispa*	Actiniaria	Trypsin	58	n.a.	Protein	2022	n.a.	Neuroprotective	[107]
A0A6B7FA07	MH249939	HCIQ4c7	PI-stichotoxin-Hcr2j	Type II KTx	*Radianthus crispa*	Actiniaria	Trypsin, Elastase, P2RX7	58	Vietnam	Protein	2020	n.a.	Neuroprotective; Antioxidant	[98]
P19651	n.a.	Sh1	Delta-stichotoxin-She1a	Type II NaTx	*Stichodactyla helianthus*	Actiniaria	n.a.	48	United States of America	Protein	1989	1SH1	n.a.	[108]

D2KX90	AB512761	Delta-TATX-Cad1a	Delta-thalatoxin-Cad1a	Type II NaTx	*Cryptodendrum adhaesivum*	Actiniaria	n.a.	49	n.a.	Protein	2010	n.a.	n.a.	[109]
P0C5G6	n.a.	Halcurin	Delta-halcutoxin-Hcg1a	Type II NaTx	*Isohalcurias carlgreni*	Actiniaria	n.a.	47	Japan	Protein	1997	n.a.	n.a.	[110]
P01534	n.a.	RpII	Delta-stichotoxin-Hmg4a	Type II NaTx	*Radianthus magnifica*	Actiniaria	n.a.	48	French Polynesia	Protein	1985	n.a.	n.a.	[111]
P08380	n.a.	RpIII	Delta-stichotoxin-Hmg4b	Type II NaTx	*Radianthus magnifica*	Actiniaria	n.a.	48	French Polynesia	Protein	1985	n.a.	n.a.	[111]
D2KX91	AB512762	Delta-TATX-Hhe1a	Delta-thalatoxin-Hhe1a	Type II NaTx	*Heterodactyla hemprichii*	Actiniaria	n.a.	49	n.a.	Protein	2010	n.a.	n.a.	[109]
B1NWR6	EU422969	Nv1-3	Delta-edwarditoxin-Nvc1c	Type II NaTx	*Nematostella vectensis*	Actiniaria	DmNav1, rNav1.4, hNav1.5	47	n.a.	Protein	2008	n.a.	n.a.	[83]
P0CH90	EU124462	Nv1-4	n.a.	Type II NaTx	*Nematostella vectensis*	Actiniaria	DmNav1, rNav1.4, hNav1.5	47	n.a.	Protein	2008	n.a.	n.a.	[83]
B1NWS4	EU124461	Nv1-1	Delta-edwarditoxin-Nvc1b	Type II NaTx	*Nematostella vectensis*	Actiniaria	DmNav1, rNav1.4, hNav1.5	47	n.a.	Protein	2008	n.a.	n.a.	[83]
B1NWS1	EU124458	Nv1-2	n.a.	Type II NaTx	*Nematostella vectensis*	Actiniaria	DmNav1, rNav1.4, hNav1.5	47	n.a.	Protein	2008	n.a.	n.a.	[83]
B1NWS8	EU124460	Nv1-6	n.a.	Type II NaTx	*Nematostella vectensis*	Actiniaria	DmNav1, rNav1.4, hNav1.5	47	n.a.	Protein	2008	n.a.	n.a.	[83]
A7SCE5	DS469622	Nv1-7	n.a.	Type II NaTx	*Nematostella vectensis*	Actiniaria	DmNav1, rNav1.4, hNav1.5	47	n.a.	Protein	2008	n.a.	n.a.	[83]
P0CH46	DS469622	Nv1-5	n.a.	Type II NaTx	*Nematostella vectensis*	Actiniaria	DmNav1, rNav1.4, hNav1.5	47	n.a.	Protein	2008	n.a.	n.a.	[83]
B1NWR7	EU124454	Nv1-8	n.a.	Type II NaTx	*Nematostella vectensis*	Actiniaria	DmNav1, rNav1.4, hNav1.5	47	n.a.	Protein	2008	n.a.	n.a.	[83]
P0CH45	DS472756	Nv1-9	Delta-edwarditoxin-Nvc1a	Type II NaTx	*Nematostella vectensis*	Actiniaria	DmNav1, rNav1.4, hNav1.5	47	n.a.	Protein	2008	n.a.	n.a.	[83]

P30832	n.a.	Neurotoxin III (RTX-III and RTX-VI)	Delta-stichotoxin-Hcr1a; Delta-stichotoxin-Hcr1f	Type II NaTx	*Radianthus crispa*	Actiniaria	Nav1.2 (RTX-VI), Nav1.3 (RTX-III), Nav1.6, BgNav1, VdNav1	48	n.a.	Protein	1985	n.a.	n.a.	[112]
P30783	n.a.	Neurotoxin II / RTX-II	Delta-stichotoxin-Hcr1b	Type II NaTx	*Radianthus crispa*	Actiniaria	n.a.	48	n.a.	Protein	1988	n.a.	n.a.	[113]
P30784	n.a.	Neurotoxin IV / RTX-IV	Delta-stichotoxin-Hcr1c	Type II NaTx	*Radianthus crispa*	Actiniaria	n.a.	48	n.a.	Protein	1988	n.a.	n.a.	[114]
P30785	n.a.	Neurotoxin V / RTX-V	Delta-stichotoxin-Hcr1d	Type II NaTx	*Radianthus crispa*	Actiniaria	n.a.	47	n.a.	Protein	1988	n.a.	n.a.	[114]
P30831	n.a.	Neurotoxin I / RTX-I	Delta-stichotoxin-Hcr1e	Type II NaTx	*Radianthus crispa*	Actiniaria	n.a.	48	n.a.	Protein	1989	n.a.	n.a.	[115]
Q76CA0	AB110015	Gigantoxin III	Delta-stichotoxin-Sgt3a	Type II NaTx	*Stichodactyla gigantea*	Actiniaria	n.a.	48	Japan	Protein	2003	n.a.	n.a.	[25]
B1B5I9	AB362570	SHTX IV	Delta-stichotoxin-Shd3a	Type II NaTx	*Stichodactyla haddoni*	Actiniaria	n.a.	48	Japan	Protein	2008	n.a.	n.a.	[26]
D2KX92	AB512763	Delta-TATX-Tas1a	Delta-thalatoxin-Tas1a	Type II NaTx	*Thalassianthus aster*	Actiniaria	n.a.	49	n.a.	Protein	2010	n.a.	n.a.	[109]
P61542	n.a.	APETx2	Pi-actitoxin-Ael2b	Type III KTx	*Anthopleura elegantissima*	Actiniaria	rASIC3, ASIC2b+3, ASIC1b+3, ASIC1a+3, ERG1/Kv11.1, Kv3.4, Nav1.2, Nav1.6, Nav1.8	42	n.a.	Protein	2004	1WXN	Analgesic	[116]
C0HL52	n.a.	Hcr 1b-2	Pi-stichotoxin-Hcr5b	Type III KTx	*Radianthus crispa*	Actiniaria	rASIC1a, rASIC13, Kv1.3-1.6, Kv2.1, Kv4.2, Kv7.1-7.4, hERG,	41	Vietnam	Protein	2018	n.a.	Analgesic	[117]

							QKT1, Nav1.1-1.8, BgNav, Cav3.1-3.3							
P0DMX9	FK720902	BDS-5	Kappa-actitoxin-Avd4e	Type III KTx	*Anemonia viridis*	Actiniaria	n.a.	43	France	Protein	2011	n.a.	Anticancer	[55]
C0HL40	n.a.	APETx4	Kappa-actitoxin-Ael2e	Type III KTx	*Anthopleura elegantissima*	Actiniaria	hEag1/Kv10.1, Kv1.3-1.5, Kv2.1, Nav1.4-1.6	42	n.a.	Protein	2017	n.a.	Anticancer	[118]
P11494	n.a.	BDS-1	Delta/kappa-actitoxin-Avd4a	Type III KTx	*Anemonia sulcata*	Actiniaria	Kv3.4, Kv3.1, Kv3.2, Nav1.7, Nav1.3	43	n.a.	Protein	1998	1BDS	Antihypertensive	[119]
P59084	n.a.	BDS-2	DeltaKappa-actitoxin-Avd4b	Type III KTx	*Anemonia sulcata*	Actiniaria	Kv3.4, Kv3.1	43	n.a.	Protein	1998	n.a.	Antihypertensive	[119]
C0HM68	n.a.	Hmg 1b-2	Pi/alpha-stichotoxin-Hmg5b	Type III KTx	*Radianthus magnifica*	Actiniaria	hASIC3, muscle-type T. californica $\alpha1\beta1\gamma\delta$ nAChR, human $\alpha7$ nAChR	37	Vietnam	Protein	2022	n.a.	Anti-inflammatory; Analgesic;	[120]
P0DRI4	n.a.	Hmg 1b-4	Pi-stichotoxin-Hmg5c	Type III KTx	*Radianthus magnifica*	Actiniaria	rASIC1a, r/hASIC3	41	Vietnam	Protein	2022	n.a.	Anti-inflammatory; Analgesic; Anxiolytic	[120]
P61541	n.a.	APETx1	Kappa-actitoxin-Ael2a	Type III KTx	*Anthopleura elegantissima*	Actiniaria	ERG1/Kv11.1, Nav1.1-1.6, Nav1.8, Kv1.4	42	n.a.	Protein	2003	1WQK	Antimicrobial	[121]
C0HM42	n.a.	CogiTx1	U-actitoxin-Cgg3	Type III KTx	*Condylactis gigantea*	Actiniaria	SUBTA, rStmPr1, Porcine Pancreatic Elastase, Proteinase K	48	Cuba	Protein	2023	n.a.	Antimicrobial	[122]

A0A1X9QHL1	KY229683	Crassicorin-I	na	Type III KTx	*Urticina crassicornis*	Actiniaria	n.a.	40	South Korea	Protein	2017	n.a.	Antimicrobial	[123]
B3EWF9	n.a.	APETx3	Delta-actitoxin-Ael2d	Type III KTx	*Anthopleura elegantissima*	Actiniaria	Nav1.2-1.4, Nav1.6, DmNav1, BgNav1, VdNav1	42	n.a.	Protein	2012	n.a.	Insecticides	[124]
P0DL87	n.a.	Hcr 1b-1	Pi-stichotoxin-Hcr5a	Type III KTx	*Radianthus crispa*	Actiniaria	hASIC3	41	Seychelles	Protein	2012	n.a.	Modulators of Key Neuronal Channels	[125]
C0HL53	n.a.	Hcr 1b-3	Pi-stichotoxin-Hcr5c	Type III KTx	*Radianthus crispa*	Actiniaria	rASIC1a, rASIC3	41	Vietnam	Protein	2018	n.a.	Modulators of Key Neuronal Channels	[117]
C0HL54	n.a.	Hcr 1b-4	Pi-stichotoxin-Hcr5d	Type III KTx	*Radianthus crispa*	Actiniaria	rASIC1a, rASIC3	41	Vietnam	Protein	2018	n.a.	Modulators of Key Neuronal Channels	[117]
C0HLS4	n.a.	Hmg 1b-5	Pi-stichotoxin-Hmg5a	Type III KTx	*Radianthus magnifica*	Actiniaria	rASIC3, rASIC1a, muscle-type T. californica α1β1γδ nAChR	41	Vietnam	Protein	2022	n.a.	Modulators of Key Neuronal Channels	[120]
P0DMX6	FK728690	BDS-1	Kappa-actitoxin-Avd4a	Type III KTx	*Anemonia viridis*	Actiniaria	n.a.	43	France	Protein	2011	n.a.	n.a.	[55]
P0DMY4	FK725608	BDS-10	Kappa-actitoxin-Avd4j	Type III KTx	*Anemonia viridis*	Actiniaria	n.a.	49	France	Nucleotide	2011	n.a.	n.a.	[55]
P0DMY5	FK740326	BDS-11	Kappa-actitoxin-Avd4i	Type III KTx	*Anemonia viridis*	Actiniaria	n.a.	47	France	Nucleotide	2011	n.a.	n.a.	[55]
P0DMY6	FK736010	BDS-12	Kappa-actitoxin-Avd4l	Type III KTx	*Anemonia viridis*	Actiniaria	n.a.	43	France	Nucleotide	2011	n.a.	n.a.	[55]
P0DMY7	FK752236	BDS-13	Kappa-actitoxin-Avd4m	Type III KTx	*Anemonia viridis*	Actiniaria	n.a.	43	France	Nucleotide	2011	n.a.	n.a.	[55]

P0DMY8	FK745823	BDS-14	Kappa-actitoxin-Avd4n	Type III KTx	*Anemonia viridis*	Actiniaria	n.a.	43	France	Nucleotide	2011	n.a.	n.a.	[55]
P0DQN7	FK725211	BDS-15	Kappa-actitoxin-Avd4o	Type III KTx	*Anemonia viridis*	Actiniaria	n.a.	43	n.a.	Nucleotide	2013	n.a.	n.a.	[126]
P0DQN6	n.a.	BDS-16	Kappa-actitoxin-Avd4p	Type III KTx	*Anemonia viridis*	Actiniaria	n.a.	43	France	Protein	2018	n.a.	n.a.	[127]
P0DMX7	FK744472	BDS-3	Kappa-actitoxin-Avd4c	Type III KTx	*Anemonia viridis*	Actiniaria	n.a.	43	France	Nucleotide	2011	n.a.	n.a.	[55]
P0DMX8	FK722457	BDS-4	Kappa-actitoxin-Avd4d	Type III KTx	*Anemonia viridis*	Actiniaria	n.a.	43	France	Nucleotide	2011	n.a.	n.a.	[55]
P0DMY0	FK754940	BDS-6	Kappa-actitoxin-Avd4f	Type III KTx	*Anemonia viridis*	Actiniaria	n.a.	47	France	Nucleotide	2011	n.a.	n.a.	[55]
P0DMY1	FK736435	BDS-7	Kappa-actitoxin-Avd4g	Type III KTx	*Anemonia viridis*	Actiniaria	n.a.	43	France	Nucleotide	2011	n.a.	n.a.	[55]
P0DMY2	FK723172	BDS-8	Kappa-actitoxin-Avd4h	Type III KTx	*Anemonia viridis*	Actiniaria	n.a.	44	France	Nucleotide	2011	n.a.	n.a.	[55]
P69930	AB180686	Am-II	Delta-actitoxin-Amc2a	Type III KTx	*Antheopsis maculata*	Actiniaria	n.a.	43	Japan	Protein	2005	n.a.	n.a.	[58]
P0DMX5	FG392547	n.a.	U-AITX-Ael2c / U-AITX-Ael1a	Type III KTx	*Anthopleura elegantissima*	Actiniaria	n.a.	42	United States of America	Nucleotide	2008	n.a.	n.a.	[128]
P86470	n.a.	Bc V	n.a.	Type III KTx	*Bunodosoma caissarum*	Actiniaria	n.a.	33	n.a.	Protein	2010	n.a.	n.a.	n.a.
P84919	n.a.	Bc IV	U-actitoxin-Bcs2a	Type III KTx	*Bunodosoma caissarum*	Actiniaria	n.a.	41	Brazil	Protein	2006	n.a.	n.a.	[129]
P86463	n.a.	Bcg 25.52	n.a.	Type III KTx	*Bunodosoma cangicum*	Actiniaria	n.a.	33	Brazil	Protein	2008	n.a.	n.a.	[59]

P86462	n.a.	Bcg 28.78	n.a.	Type III KTx	*Bunodosoma cangicum*	Actiniaria	n.a.	37	Brazil	Protein	2008	n.a.	n.a.	[59]
P86464	n.a.	Bcg 29.21	n.a.	Type III KTx	*Bunodosoma cangicum*	Actiniaria	n.a.	36	Brazil	Protein	2008	n.a.	n.a.	[59]
P86461	n.a.	Bcg 31.16	n.a.	Type III KTx	*Bunodosoma cangicum*	Actiniaria	n.a.	38	Brazil	Protein	2008	n.a.	n.a.	[59]
G0W2H7	HE577144	Bg1a	U-AITX-Bg1a	Type III KTx	*Bunodosoma granuliferum*	Actiniaria	n.a.	41	Cuba	Protein	2012	n.a.	n.a.	[130]
G0W2H8	HE577145	Bg1b	U-actitoxin-Bgr3a	Type III KTx	*Bunodosoma granuliferum*	Actiniaria	n.a.	43	Cuba	Protein	2012	n.a.	n.a.	[130]
G0W2H9	HE577146	Bg1c	U-actitoxin-Bgr3b	Type III KTx	*Bunodosoma granuliferum*	Actiniaria	n.a.	43	Cuba	Nucleotide	2012	n.a.	n.a.	[130]
G0W2I0	HE577147	Bg1d	U-actitoxin-Bgr3c	Type III KTx	*Bunodosoma granuliferum*	Actiniaria	n.a.	43	Cuba	Protein	2012	n.a.	n.a.	[130]
G0W2I1	HE577148	Bg1e	U-actitoxin-Bgr3d	Type III KTx	*Bunodosoma granuliferum*	Actiniaria	n.a.	39	Cuba	Nucleotide	2012	n.a.	n.a.	[130]
C0HK75	n.a.	PhcrTx2	Toxin PhcrTx2	Type III KTx	*Phymanthus crucifer*	Actiniaria	n.a.	46	Cuba	Protein	2018	n.a.	n.a.	[131]
P0DUG3	n.a.	Crassicorin-II	na	Type III KTx	*Urticina crassicornis*	Actiniaria	n.a.	34	South Korea	Protein	2017	n.a.	n.a.	[123]
P01535	n.a.	ATX III / Av3	Delta-actitoxin-Avd2a	Type III NaTx	*Anemonia sulcata*	Actiniaria	DmNav1	27	Italy	Protein	1975	1ANS	Insecticides	[74]
C3TS06	EU919729	Av7	Delta-actitoxin-Avd2b 4	Type III NaTx	*Anemonia viridis*	Actiniaria	n.a.	28	Israel	Nucleotide	2009	n.a.	Insecticides	[132]
C3TS07	EU919730	Av10	Delta-actitoxin-Avd2c	Type III NaTx	*Anemonia viridis*	Actiniaria	n.a.	28	Israel	Nucleotide	2009	n.a.	Insecticides	[132]

C3TS04	EU919727	Av7	Delta-actitoxin-Avd2b 2	Type III NaTx	*Anemonia viridis*	Actiniaria	n.a.	28	Israel	Nucleotide	2009	n.a.	n.a.	[132]
C3TS08	EU919731	Av7	Delta-actitoxin-Avd2b 1	Type III NaTx	*Anemonia viridis*	Actiniaria	n.a.	28	Israel	Nucleotide	2009	n.a.	n.a.	[132]
C3TS10	EU919733	Av7	Delta-actitoxin-Avd2b 3	Type III NaTx	*Anemonia viridis*	Actiniaria	n.a.	28	Israel	Nucleotide	2009	n.a.	n.a.	[132]
P0DMZ2	n.a.	Da I / Da II	Delta-actitoxin-Dar1b	Type III NaTx	*Dofleinia armata*	Actiniaria	n.a.	31	n.a.	Protein	2003	n.a.	n.a.	[133]
P09949	n.a.	Er I	Delta-actitoxin-Eqd1a	Type III NaTx	*Entacmaea quadricolor*	Actiniaria	n.a.	32	n.a.	Protein	2003	n.a.	n.a.	[133]
C0HJC4	n.a.	BcsTx3	Kappa-actitoxin-Bcs4a	Type V KTx	*Bunodosoma caissarum*	Actiniaria	rKv1.1, Shaker IR, rKv1.2 , hKv1.3, rKv1.6	50	Brazil	Protein	2013	n.a.	n.a.	[134]
P0DMD7	FC839755	MsePTx1	U-metritoxin-Msn2a	Type V KTx	*Metridium senile*	Actiniaria	n.a.	50	n.a.	Nucleotide	2013	n.a.	n.a.	n.a.
A7RMN1	DS469520	NvePTx1	n.a.	Type V KTx	*Nematostella vectensis*	Actiniaria	n.a.	50	n.a.	Protein	2007	n.a.	n.a.	[135]
C0HJD9	n.a.	AbeTx1	n.a.	Type VI KTx	*Actinia bermudensis*	Actiniaria	Kv1.1, Kv1.2, Kv1.6, Kv1.3, Shaker IR	17	Brazil	Protein	2018	n.a.	n.a.	[136]
P0C1G1	n.a.	Up-I	n.a.	Unknown	*Urticina piscivora*	Actiniaria	n.a.	46	Canada	Protein	1995	n.a.	Antiarrhythmic; Anticancer	[137]
P0DQJ2	n.a.	U-AITX-Ate1	U-actitoxin-Ate1	Unknown	*Actinia tenebrosa*	Actiniaria	Lipid membranes	28	Australia	Protein	2019	6OQP	Anticancer	[138]
C0HJB1	n.a.	PhcrTx1	Pi-phymatoxin-Pcf1a	Unknown	*Phymanthus crucifer*	Actiniaria	ASIC	32	Cuba	Protein	2014	n.a.	Modulators of Key Neuronal Channels	[139]

P69944	n.a.	AETX II	U-actitoxin-Aer2a	Unknown	*Anemonia erythraea*	Actiniaria	n.a.	59	Japan	Protein	1997	n.a.	n.a.	[85]
P69945	n.a.	AETX III	U-actitoxin-Aer2b	Unknown	*Anemonia erythraea*	Actiniaria	n.a.	59	Japan	Protein	1997	n.a.	n.a.	[85]
A0A6I8WFP9	n.a.	Acatoxin 1/ Acatx1	n.a.	Unknown	*Anthopleura cascaia*	Actiniaria	n.a.	32	n.a.	Protein	2019	6NK9	n.a.	n.a.
P58305	n.a.	Granulitoxin/GRX	n.a.	Unknown	*Bunodosoma cangicum*	Actiniaria	n.a.	21	Brazil	Protein	1998	n.a.	n.a.	[140]
P86469	n.a.	Toxin Bcg III 15.67	n.a.	Unknown	*Bunodosoma cangicum*	Actiniaria	n.a.	29	n.a.	Protein	2010	n.a.	n.a.	n.a.
P0DPE6	n.a.	CjTL8	n.a.	Unknown	*Epiactis japonica*	Actiniaria	n.a.	37	Russia	Nucleotide	2017	n.a.	n.a.	[93]
P0DPE7	n.a.	CjTL7	n.a.	Unknown	*Epiactis japonica*	Actiniaria	n.a.	29	Russia	Nucleotide	2017	n.a.	n.a.	[93]
P0DQR6	n.a.	Neurotoxin ShK-like1	n.a.	Unknown	*Nematostella vectensis*	Actiniaria	n.a.	44	n.a.	Protein	2020	n.a.	n.a.	[141]
P84851	n.a.	PsTX-115	n.a.	Unknown	*Phyllodiscus semoni*	Actiniaria	n.a.	23	Japan	Protein	2007	n.a.	n.a.	[142]
P0CG44	n.a.	Uc-I	n.a.	Unknown	*Urticina crassicornis*	Actiniaria	n.a.	29	United States of America	Protein	2009	n.a.	n.a.	[143]
P0DY40	n.a.	Unknown 12C protein	n.a.	Unknown	*Calliactis polypus*	Actiniaria	n.a.	107	Australia	Protein	2023	n.a.	n.a.	[38]
P0DY43	n.a.	Putative neurotoxin NaH-Cpp1a (Unknown C6 protein)	n.a.	Unknown	*Calliactis polypus*	Actiniaria	n.a.	50	Australia	Protein	2024	n.a.	n.a.	[144]

P0DY44	n.a.	Structural toxin peptide SA9-Cpp1a	n.a.	Unknown	*Calliactis polypus*	Actiniaria	n.a.	95	Australia	Protein	2024	n.a.	n.a.	[144]
P0DRC2	ON605615	Ms11a-3	Alpha-anmTX-Ms11a-3	Unknown	*Metridium senile*	Actiniaria	T. california α1β1γδ nAChR, human α7 nAChR , nAChR sub-types rα9α10, mα1β1δε, rα3β4, rα7, hα7	42	Norway	Protein	2022	6XYI	n.a.	[145]
P0DRC0	ON605613	Ms11a-1	Alpha-anmTX-Ms11a-1	Unknown	*Metridium senile*	Actiniaria	T. california α1β1γδ nAChR, human α7 nAChR	40	Norway	Protein	2022	n.a.	n.a.	[145]
P0DRC1	ON605614	Ms11a-2	Alpha-anmTX-Ms11a-2	Unknown	*Metridium senile*	Actiniaria	T. california α1β1γδ nAChR, human α7 nAChR	40	Norway	Protein	2022	6XYH	n.a.	[145]
P0DRC3	ON605616	Ms11a-4	Alpha-anmTX-Ms11a-4	Unknown	*Metridium senile*	Actiniaria	T. california α1β1γδ nAChR, human α7 nAChR	32	Norway	Protein	2022	n.a.	n.a.	[145]
A0A5J6K6K4	MK608361	Nv4	n.a.	Unknown	*Nematostella vectensis*	Actiniaria	n.a.	61	n.a.	Protein	2019	n.a.	n.a.	[146]
A0A5J6KCM0	MK608362	Nv5	n.a.	Unknown	*Nematostella vectensis*	Actiniaria	n.a.	63	n.a.	Protein	2019	n.a.	n.a.	[146]
P0DQR2	n.a.	Nv6	n.a.	Unknown	*Nematostella vectensis*	Actiniaria	n.a.	65	n.a.	Nucleotide	2019	n.a.	n.a.	[146]
P0DQR3	n.a.	Nv7	n.a.	Unknown	*Nematostella vectensis*	Actiniaria	n.a.	68	n.a.	Protein	2019	n.a.	n.a.	[146]

P0DQR4	n.a.	Nv8	n.a.	Unknown	*Nematostella vectensis*	Actiniaria	n.a.	65	n.a.	Nucleotide	2019	n.a.	n.a.	[146]
P0DRH7	n.a.	Avt1	U-actitoxin-Avt1	Unknown	*Aulactinia veratra*	Actiniaria	n.a.	28	Australia	Protein	2005	9CRB	Stability Properties	[147]
K7Z9Q9	JQ829079	NEP-6	n.a.	Zinc metalloprote ase	*Nematostella vectensis*	Actiniaria	n.a.	287	n.a.	Protein	2013	n.a.	n.a.	[41]

Table S3. Cnidaria toxins isolated from the literature. The number of aa corresponds to the mature sequence (those complete, fragments or whose propeptide could not be identified are marked with *). n.a – not available.

UniProt	GenBank	Name	Other	Family	Species	Order	Targets	Nº aa	Location	Evidence	Year	3D structure	Biomedical Potencial	Ref
n.a.	n.a.	RTX-S	n.a.	Actinoporin	*Radianthus crispa*	Actiniaria	n.a.	n.a.	Seychelles	Protein	2002	n.a.	n.a.	[14]
n.a.	n.a.	RTX-G	n.a.	Actinoporin	*Radianthus crispa*	Actiniaria	n.a.	n.a.	Seychelles	Protein	2002	n.a.	n.a.	[14]
n.a.	n.a.	Caissarolysi n I/ Bcs-I	n.a.	Actinoporin /Hemolysin	*Bunodosoma caissarum*	Actiniaria	Sphingomye lin	n.a.	Brazil	Protein	2006	n.a.	n.a.	[148]
n.a.	n.a.	Sticholysin 2	Delta-Pocilopotoxi n-Spi1	Actinoporin /Hemolysin	*Stylophora pistillata*	Scleractinia	n.a.	194*	Israel	Protein	2018	n.a.	n.a.	[149]
n.a.	UWV31474/ ON158507	Alciporin	n.a.	Actinoporin -like	*Millepora alcicornis*	Hydrozoa	Sphingomye lin	371*	Spain	Protein	2022	n.a.	n.a.	[150]
n.a.	CAA06314.1 / AJ005052.1	PMP1	n.a.	Astacin-like Metalloprot einase	*Podocoryna carnea*	Hydrozoa	n.a.	195	n.a.	Nucleotide	1998	n.a.	n.a.	[151]
P0DQQ8	n.a.	ACE-inhibitory peptide	n.a.	Bradykinin-potentiating peptide family	*Chiropsoides quadrigatus*	Cubozoa	Angiotensin -converting enzyme (ACE)	222	Philippines	Protein	2016	n.a.	Antihyperte nsive	[152]
n.a.	n.a.	Goniopora toxin	n.a.	Ca^{2+} channel toxin	*Goniopora sp.*	Scleractinia	Ca^{2+} channels	n.a.	Jordan	Protein	1986	n.a.	n.a.	[153]
Q331K1	AY167572	Cystatin J	n.a.	Cystatin family	*Cyanea capillata*	Scyphozoa	Papain	10	n.a.	Nucleotide	2003	n.a.	n.a.	[154]
A8QZJ5	AB299385	MCTx-1	n.a.	Dermatopon tin family	*Millepora dichotoma*	Hydrozoa	Oligosaccha rides and	503	Japan	Protein	2008	n.a.	n.a.	[155]

							glycosphingolipids							
P0DMY9	FK735889	Gigantoxin-4	U-actitoxin-Avd12a	EGF domain peptide	*Anemonia viridis*	Actiniaria	n.a.	48	France	Nucleotide	2011	n.a.	n.a.	[55]
P0DMZ0	FK749754	Gigantoxin-5	U-actitoxin-Avd12b	EGF domain peptide	*Anemonia viridis*	Actiniaria	n.a.	48	France	Nucleotide	2011	n.a.	n.a.	[55]
Q9GV16	AB047321	EGCase	n.a.	Glycosyl hydrolase 5 (cellulase A) family	*Cyanea nozakii*	Scyphozoa	n.a.	113	Japan	Protein	2000	n.a.	n.a.	[156]
n.a.	n.a.	Physalitoxin	n.a.	Hemolysin	*Physalia physalis*	Hydrozoa	n.a.	n.a.	United States of America	Protein	1981	n.a.	n.a.	[157]
n.a.	n.a.	NvLysin1b	n.a.	Hydralysin-like	*Nematostella vectensis*	Actiniaria	n.a.	n.a.	n.a.	Nucleotide	2012	n.a.	n.a.	[158]
n.a.	n.a.	PlKuz1	n.a.	Kunitz-type	*Porites lutea*	Scleractinia	Kv1.3, Plasma kallikrein (PKa)	53	China	Protein	2025	n.a.	Anti-inflammatory; Neuroprotective	[159]
n.a.	n.a.	GcKuz1	n.a.	Kunitz-type	*Goniopora columna*	Scleractinia	Plasma kallikrein (PKa), FXIIa	53	Australia	Protein	2025	n.a.	Anti-thrombotic	[160]
n.a.	n.a.	BTX I	n.a.	n.a.	*Bolocera tuediae*	Actiniaria	n.a.	47	Baltic Sea	Protein	1980	n.a.	n.a.	[161]
n.a.	n.a.	A2-PLTX-Pcb1a	n.a.	Phospholipase A2	*Palythoa caribaeorum*	Zoantharia	n.a.	149	Mexico	Protein	2019	n.a.	n.a.	[162]
n.a.	n.a.	Milleporin 1	n.a.	Phospholipase A2	*Millepora platyphylla*	Hydrozoa	Arachidonyl phosphatidylcholine	n.a.	Egypt	Protein	2004	n.a.	n.a.	[163]
n.a.	n.a.	ZoaKuz1	n.a.	Potassium channel toxin	*Zoanthus natalensis*	Zoantharia	n.a.	53	n.a.	Protein	2019	n.a.	Neuroprotective	[164]
C0HM70	n.a.	Hact-SCRiP1	n.a.	SCRiP	*Heliofungia actiniformis*	Scleractinia	n.a.	41	Australia	Protein	2022	7LX4	n.a.	[165]
n.a.	n.a.	SA8	n.a.	Sea anemone 8 toxin family	*Telmatactis stephensoni*	Actiniaria	hKv1.2	49	Australia	Protein	2022	n.a.	n.a.	[65]
P0DMZ8	FK719982	AV-1	U-actitoxin-Avd13a/b	Sea anemone BBH family	*Anemonia viridis*	Actiniaria	n.a.	29	France	Nucleotide	2011	n.a.	n.a.	[55]

P0DMZ9	FK736087	AV-2	U-actitoxin-Avd13b	Sea anemone BBH family	*Anemonia viridis*	Actiniaria	n.a.	29	France	Nucleotide	2011	n.a.	n.a.	[55]
n.a.	n.a.	BTTX II	n.a.	n.a.	*Bolocera tuediae*	Actiniaria	n.a.	47	Baltic Sea	Protein	1980	n.a.	n.a.	[161]
n.a.	n.a.	GPT	n.a.	Sodium channel toxin	*Goniopora sp.*	Scleractinia	Sodium channels	88	Japan	Protein	1987	n.a.	n.a.	[166]
P0DPF1	n.a.	Ask132958	n.a.	Type I KTx (1a subfamily)	*Anemonia sulcata*	Actiniaria	n.a.	29	n.a.	Protein	2018	5WCV	Stability Properties	[167]
P31713	n.a.	ShPI I	PI-stichotoxin-She2a	Type II KTx	*Stichodactyla helianthus*	Actiniaria	Kv1.1, Kv1.2, Kv1.6, Trypsin, Plasmin, Chymotrypsin, Pepsin	55	Cuba	Protein	1993	1SHP	Antimicrobial; Stability Properties	[168]
P0DQR0	n.a.	PcKuz2	PI-sphenopitoxin-Pc1b	Type II KTx	*Palythoa caribaeorum*	Zoantharia	Trypsin, elastase	51	Brazil	Nucleotide	2018	n.a.	n.a.	[105]
P0DMJ2	n.a.	AEAPI	PI-actitoxin-Aeq3c	Type II KTx	*Actinia equina*	Actiniaria	Trypsin, plasmin, α-chymotryptin	57	Japan	Protein	2008	n.a.	n.a.	[169]
P10280	n.a.	SA5 II	KappaPI-actitoxin-Avd3a	Type II KTx	*Anemonia viridis*	Actiniaria	n.a.	62	n.a.	Protein	1981	n.a.	n.a.	[170]
P0DN08	FK749713	SA5 III	KappaPI-actitoxin-Avd3e	Type II KTx	*Anemonia viridis*	Actiniaria	n.a.	59	France	Nucleotide	2011	n.a.	n.a.	[55]
P81547	n.a.	AXPI I/AXAPI	PI-actitoxin-Axm2a	Type II KTx	*Anthopleura aff. xanthogrammica*	Actiniaria	Trypsin, plasmin	58	Japan	Protein	1997	n.a.	n.a.	[171]
P81548	n.a.	AXPI II	PI-actitoxin-Axm2b	Type II KTx	*Anthopleura aff. xanthogrammica*	Actiniaria	Trypsin, α-chymotrypsin	61	Japan	Protein	1997	n.a.	n.a.	[171]
P0DMX0	n.a.	AXPI III	PI-actitoxin-Axm2b	Type II KTx	*Anthopleura aff. xanthogrammica*	Actiniaria	n.a.	58	Japan	Protein	1998	n.a.	n.a.	[172]

P0DMJ3	n.a.	AFAPI I	PI-actitoxin-Afv2a	Type II KTx	*Anthopleura fuscoviridis*	Actiniaria	Trypsin, plasmin	56	Japan	Protein	2008	n.a.	n.a.	[169]
P0DMJ4	n.a.	AFAPI III	PI-actitoxin-Afv2b	Type II KTx	*Anthopleura fuscoviridis*	Actiniaria	Trypsin, plasmin	56	Japan	Protein	2008	n.a.	n.a.	[169]
C0HK72	n.a.	HMRG1	PI-stichotoxin-Hmg3a	Type II KTx	*Radianthus magnifica*	Actiniaria	n.a.	56	Vietnam	Protein	2018	n.a.	n.a.	[173]
C0HK73	n.a.	HMGS1	PI-stichotoxin-Hmg3b	Type II KTx	*Radianthus magnifica*	Actiniaria	n.a.	56	Vietnam	Protein	2018	n.a.	n.a.	[173]
C0HK74	n.a.	HMGS2	PI-stichotoxin-Hmg3c	Type II KTx	*Radianthus magnifica*	Actiniaria	n.a.	56	Vietnam	Protein	2018	n.a.	n.a.	[173]
P16344	n.a.	Jn-IV	PI-stichotoxin-Hcr2a	Type II KTx	*Radianthus crispa*	Actiniaria	Trypsin	56	n.a.	Protein	1985	n.a.	n.a.	[174]
P81129	n.a.	SHPI II	PI-stichotoxin-She2b	Type II KTx	*Stichodactyla helianthus*	Actiniaria	n.a.	55	n.a.	Protein	1998	n.a.	n.a.	[175]
P0DMJ5	n.a.	InhVJ	PI-stichotoxin-Hcr2e	Type II KTx	*Radianthus crispa*	Actiniaria	Trypsin	56	n.a.	Protein	2006	n.a.	Neuroprotective; Antioxidant	[176]
n.a.	n.a.	PaTX	n.a.	Type III NaTx	*Exocoelactis actinostoloides*	Actiniaria	n.a.	31	Japan	Protein	1980	n.a.	n.a.	[177]
n.a.	n.a.	RmI	n.a.	Unknown	*Radianthus crispa*	Actiniaria	Histamine	n.a.	Seychelles	Protein	2002	n.a.	Anti-histaminic	[14]
n.a.	n.a.	RmII	n.a.	Unknown	*Radianthus crispa*	Actiniaria	Histamine	n.a.	Seychelles	Protein	2002	n.a.	Anti-histaminic	[14]
P0DUW7	n.a.	Antioxidant protein SmP90	n.a.	Unknown	*Stomolophus meleagris*	Scyphozoa	n.a.	15	China	Protein	2012	n.a.	Antioxidant	[178]
C0HM69	n.a.	Hact-2	n.a.	Unknown	*Heliofungia actiniformis*	Scleractinia	n.a.	21	Australia	Protein	2022	7LRW	Cell proliferation	[165]
P0DUS1	n.a.	Hact-1	n.a.	Unknown	*Heliofungia actiniformis*	Scleractinia	n.a.	12	Australia	Protein	2020	n.a.	Immunomodulatory	[179]
C0HM25	n.a.	Hact-3	n.a.	Unknown	*Heliofungia actiniformis*	Scleractinia	n.a.	35	Australia	Protein	2022	7LT7	n.a.	[165]
C0HM26	n.a.	Hact-4	n.a.	Unknown	*Heliofungia actiniformis*	Scleractinia	n.a.	44	Australia	Protein	2022	7MJ3	n.a.	[165]
P0DQW8	n.a.	Cytolysin Oshem 1	n.a.	Unknown	*Olindias sambaquiensis*	Hydrozoa	n.a.	29	Brazil	Protein	2014	n.a.	n.a.	[180]

P0DQW9	n.a.	Cytolysin Oshem 2	n.a.	Unknown	*Olindias sambaquiensis*	Hydrozoa	n.a.	31	Brazil	Protein	2014	n.a.	n.a.	[180]
n.a.	n.a.	Anthozoan neurotoxin	n.a.	Unknown	*Palythoa caribaeorum*	Zoantharia	Sodium Channels	32	Mexico	Protein	2014	n.a.	n.a.	[181]
n.a.	n.a.	Toxin α-KTx 1.13	n.a.	Potassium channel toxin	*Catostylus sp.*	Scyphozoa	Potassium channels	n.a.	Sri Lanka	Protein	2024	n.a.	n.a.	[182]
n.a.	YEV25364.1	NavTx-Bg1	n.a.	Sodium channel toxin	*Bunodosoma goanense*	Actiniaria	rNav1.4, hKv1.5	48	India	Protein	2026	n.a.	n.a.	[183]
n.a.	n.a.	PpV9.4	n.a.	Neurotoxin	*Physalia physalis*	Hydrozoa	n.a.	n.a.	Cuba	Protein	2012	n.a.	Insulin Modulation	[184]
n.a.	n.a.	PpV19.3	n.a.	Neurotoxin	*Physalia physalis*	Hydrozoa	n.a.	n.a.	Cuba	Protein	2012	n.a.	Insulin Modulation	[184]
n.a.	n.a.	P3	n.a.	Neurotoxin	*Physalia physalis*	Hydrozoa	n.a.	n.a.	n.a.	Protein	1989	n.a.	n.a.	[185]
n.a.	n.a.	P1	n.a.	Neurotoxin	*Physalia physalis*	Hydrozoa	Nicotinic cholinergic receptors	n.a.	n.a.	Protein	1990	n.a.	n.a.	[186]
n.a.	n.a.	CcNT	n.a.	Sodium channel toxin	*Cyanea capillata*	Scyphozoa	Sodium channels	n.a.	Scotland	Protein	2012	n.a.	n.a.	[187]
n.a.	n.a.	Hemolysin	n.a.	Cytolysin	*Carybdea marsupialis*	Cubozoa	n.a.	n.a.	Italy	Protein	1995	n.a.	n.a.	[188]
n.a.	n.a.	CmNt	n.a.	Neurotoxin	*Carybdea marsupialis*	Cubozoa	n.a.	n.a.	Mexico	Protein	2006	n.a.	n.a.	[189]
n.a.	n.a.	CmHl1	n.a.	Cytolysin	*Carybdea marsupialis*	Cubozoa	n.a.	n.a.	Mexico	Protein	2006	n.a.	n.a.	[189]
n.a.	n.a.	CmHl5	n.a.	Cytolysin	*Carybdea marsupialis*	Cubozoa	n.a.	n.a.	Mexico	Protein	2006	n.a.	n.a.	[189]
n.a.	n.a.	CmHl7	n.a.	Cytolysin	*Carybdea marsupialis*	Cubozoa	n.a.	n.a.	Mexico	Protein	2006	n.a.	n.a.	[189]
n.a.	n.a.	Rhizoprotease	n.a.	Zinc metalloprotease	*Rhizostoma pulmo*	Scyphozoa	n.a.	n.a.	India	Protein	2017	n.a.	n.a.	[190]
n.a.	n.a.	Rhizolysin	n.a.	Cytolysin	*Rhizostoma pulmo*	Scyphozoa	n.a.	n.a.	Italy	Protein	1988	n.a.	n.a.	[191]
n.a.	n.a.	*P. variabilis* ShK/Aurelin	n.a.	Neurotoxin	*Palythoa variabilis*	Zoantharia	n.a.	41	Brazil	Protein	2016	n.a.	n.a.	[192]
n.a.	n.a.	*Anthozoan neurotoxin-like*	n.a.	Neurotoxin	*Palythoa variabilis*	Zoantharia	n.a.	22	Brazil	Protein	2016	n.a.	n.a.	[192]

n.a.	AOO35153.1	*Chrysaoralin*	n.a.	Pore-Forming Toxin (Lectin)	*Chrysaora quinquicherra*	Scyphozoa	n.a.	454	United States	Nucleotide	2017	n.a.	n.a.	[193]
n.a.	n.a.	A1A-c14965_c0_g1	n.a.	Unkown	*Anthopleura midori*	Actiniaria	n.a.	80	China	Nucleotide	2025	n.a.	n.a.	[194]
n.a.	n.a.	B3a-c29555_c4_g4	n.a.	Neurotoxin	*Actinia equina*	Actiniaria	Cav3.1	9	China	Protein	2025	n.a.	n.a.	[194]
H9CNF5	P0DMY9	Gigantoxin IV	U-AITX-Avd12a	Actinoporin	*Stichodactyla gigantea*	Actiniaria	Sphingomyelin	77*	China	Protein	2011	n.a.	n.a.	[195]
n.a.	n.a.	Tealiatoxin	n.a.	Hemolysin	*Urticina felina*	Actiniaria	n.a.	n.a.	n.a.	Protein	1986	n.a.	Anti-histaminic	[196]

Table S4. Cnidaria species targeted in venom proteomics, peptidomics and/or proteotranscriptomic and/or proteogenomic studies. Key toxins are highlighted in green.

Species	Order	Toxins	Tissues	Location	Year	Ref
Actinia equina	Actiniaria	A.superbus venom factor, Acyl-CoA ligase, Acyl-CoA synthetase, Aflatoxin B1, Alpha-elapitoxin-Al2a, Alpha-latrotoxin, Aminotransferase, AM-toxin biosynthesis protein 12-2, AM-toxin synthetase AMT1, Ankyrin repeat domain-containing protein SAT10, Astacin-like metalloprotease, Beta-1,4-mannosyltransferase bre-3, Bifunctional cytochrome P450/NADPH--P450 reductase ALT2, Celestoxin, Cephalotoxin-like protein, Cobra venom factor, Complement C3, Complement C4, Conotoxin, Cysteine-rich venom protein, Cytochrome P450 monooxygenase, Cytoinsectotoxin, Cytolytic toxin-alpha, Delta-actitoxin-Aeq2a, delta-actitoxin-Aeq2a-like, Delta-actitoxin-Aeq2b 2, Delta-actitoxin-Aeq2b 3, DELTA-alicitoxin-Pse2a, DELTA-alicitoxin-Pse2b, delta-latroinsectotoxin-Lt1a-like isoform X1, DELTA-miturgitoxin-Cp2a, DELTA-thalatoxin-Avl2a, Deoxypodophyllotoxin synthase, Dermonecrotic toxin LiSicTox, Dermonecrotic toxin LspiSicTox, Dermonecrotic toxin SdSicTox, Dual function macrocyclase-peptidase POPB, Dual O-methyltransferase/FAD-dependent monooxygenase CTB3, Fatty acid synthase, GDP-mannose 4,6 dehydratase 1, Glycerotoxin (Fragment), HC-toxin synthetase, Highly reducing polyketide synthase, Hyaluronidase, Hydroxylase/desaturase CTB9, Kappa-4-bungarotoxin, Kappa-actitoxin-Ate Potassium channel toxin type 3 BDS, Kappa-actitoxin-Avd4i, Kappa-actitoxin-Bcs4a, KappaPI-actitoxin-Ael3a, Kappa-scoloptoxin(07)-Ssm2c, Ketoreductase CTB6, Killer toxin subunits alpha/beta, Kunitz-type kappaPI-theraphotoxin-Hs1e, Kunitz-type serine protease inhibitor long epsilon-dendrotoxin Arg55, Lipolysis-activating peptide 1-alpha chain, Mucoricin, M-zodatoxin-Lt4b, M-zodatoxin-Lt6a/c, NAD-dependent epimerase/dehydratase ALT6, NADH-dependent flavin oxidoreductase nadA, Neoverrucotoxin subunit alpha, neoverrucotoxin subunit beta-like, Neurotoxin beta-KTx 52.1, Neurotoxin Cex6 (Fragment), Non-reducing polyketide synthase, Nonribosomal peptide synthetase, Norsolorinic acid synthase, Omega-conotoxin-like SVIA mutant 2, O-methylsterigmatocystin oxidoreductase, O-methyltransferase, Ophiophagus venom factor, Phospholipase A2 A2-actitoxin-Ucs2a, PI-actitoxin-Aeq3a, PI-actitoxin-Aeq3a-like, PI-actitoxin-Aeq3c, PI-actitoxin-Aeq3c, Potassium channel toxin alpha-KTx 6.2, Potassium channel toxin BmTXK-beta, Prenyltransferase vrtC, Probable esterase TOX9, Protein KTI12, Putative 15-O-acetyltransferase SAT12, Putative neurotoxin-I (Fragment), ras-related C3 botulinum toxin	Tentacles (red and green morphotypes)	Portugal	2024	[197]

		substrate, Reducing polyketide synthase, Satratoxin biosynthesis SC3 cluster transcription factor SAT20, Scoloptoxin SSD14, SE-cephalotoxin, Serine protease VLSP-1, Short-chain dehydrogenase/reductase, snake venom 5'-nucleotidase-like, Snake venom metalloprotease inhibitor 02D01, Snake venom metalloproteinase, Snake venom serine protease, Stonustoxin subunit alpha, Stonustoxin subunit beta, Synergistic-type venom protein S2C4, Threonine dehydratase-like protein AKTS1-1, Thrombin-like enzyme cerastocytin, Toxin a, Toxin b (Fragment), Toxin Bro, Toxin c, Toxin candidate (26), Toxin ICK-42, Toxin PsTX-60B, Toxin tox21A, Toxin TxP-I, Transcription activator AKTR-1, Transcription factor otaR1, Transcriptionnal regulator VdtR, Turripeptide OL172, TX-like toxin (Fragment), Type III potassium channel toxin protein, Type III potassium channel toxin protein (Fragment), U10-theraphotoxin-Cg1a 2, U16-lycotoxin-Ls1a, U32-theraphotoxin-Cg1a, U-actitoxin-Avd3n-like, U-actitoxin-Avd3s (Fragment), U-actitoxin-Avd8a-like, U-actitoxin-Avd8e-like, U-actitoxin-Avd9c, U-actitoxin-Bgr3d, U-scoloptoxin, Venom allergen, Venom carboxylesterase-6, Venom factor, Venom metalloproteinase antarease-like, Venom phosphodiesterase, Venom plasminogen activator Haly-PA, Venom prothrombin activator nigrarin-D, Venom prothrombin activator omicarin-C non-catalytic subunit, Venom prothrombin activator oscutarin-C non-catalytic subunit, Venom prothrombin activator pseutarin-C non-catalytic subunit, Verrucotoxin subunit beta, Vitellogenin (Fragment), Zinc finger CCCH-type antiviral protein 1, Zinc metalloproteinase/disintegrin, Zinc metalloproteinase-disintegrin-like				
Actinia fragacea	Actiniaria	Actinoporin, Cobra venom factor-like, Cytolysin, DELTA-actitoxin-Afr1b, DELTA-actitoxin-Afr1c, DELTA-alicitoxin-Pse2b-like, DELTA-thalatoxin-Avl1a-like, DELTA-actitoxin-Aeq1b, Kappa-actitoxin-Avd4e-like, Kappa-actitoxin-Avd4m, KappaPI-actitoxin-Avd3a, KappaPI-actitoxin-Avd3e-like, Natterin-4-like, Nematocyst expressed protein 6-like, Neuropeptide ShK-like2, Phospholipase A2, Phospholipase A2 A2-actitoxin-Cgg2a-like, PI-actitoxin-Aeq3a-like, Delta-actitoxin-Aeq2b 2, Sodium channel toxin protein, Type III potassium channel toxin protein, U-actitoxin-Avd3g, U-actitoxin-Avd3r, U-actitoxin-Avd8d-like, Venom phosphodiesterase-like, Venom prothrombin activator oscutarin-C non-catalytic subunit-like isoform X1, Verrucotoxin subunit beta, A disintegrin and metalloproteinase with thrombospondin motifs 16-like, A disintegrin and metalloproteinase with thrombospondin motifs 18-like, A disintegrin and metalloproteinase with thrombospondin motifs 6-like, A disintegrin and metalloproteinase with thrombospondin motifs adt-1-like isoform X1, A disintegrin and metalloproteinase with thrombospondin motifs adt-2-like, Acidic endochitinase-like, Acidic mammalian chitinase-like, ADAM10 endopeptidase, Cytosolic 5'-nucleotidase 1A-like isoform X1, Digestive cysteine proteinase 2-like, Golgi-associated plant pathogenesis-related protein 1-like, Group XV phospholipase A2, Group XV phospholipase A2-like, Matrix metalloproteinase-9-like, Metalloendopeptidase, Peroxiredoxin-2-like isoform X1, Peroxiredoxin-5, Peroxiredoxin-6-like, Peroxiredoxin-like 2A, Phosphoinositide phospholipase C, Phospholipase A-2-activating protein-like, Serine protease K12H4.7 OS=Actinia tenebrosa OX=6105 GN=LOC116303022 PE=3 SV=1, Thioredoxin-dependent peroxiredoxin, Zinc metalloproteinase nas-13-like, Zinc metalloproteinase nas-15-like, Zinc metalloproteinase nas-15-like isoform X1, Zinc metalloproteinase nas-4-like, Zinc metalloproteinase-disintegrin-like berythractivase isoform X1	Nematocysts, Acrorhagi, Column, Mesenteric Filaments, Mucus, Tentacles	Portugal	2025	[198]
Anemonia viridis	Actiniaria	neurotoxin - type 1 K (full=potassium channel toxin x1), neurotoxin - type 1 Na (toxin 2-1 precursor), neurotoxin - type 2 K (kalicludine-2), neurotoxin - type 2 K (kalicludine-1), neurotoxin - type 2 K (kunitz-type protease inhibitor 2), neurotoxin - type 2 K (serine proteinase), neurotoxin - type 3 K (BDS-1), neurotoxin - type 3 K, putative - MAC-PF, putative - ShKT	Nematocysts	Israel	2015	[199]
Anthopleura dowii	Actiniaria	Delta-actitoxin-Avd1e 1, Delta-actitoxin-Ael1b, Delta-actitoxin-Axm1f, Delta-actitoxin-Ael1c, Delta-actitoxin-Axm1a, KappaPI-actitoxin-Ael3a, Leukocyte elastase inhibitor, Equistatin, Aerolysin-like, Predicted protein, CUB and peptidase domain-containing protein 1, Metalloendopeptidase, Carboxypeptidase A4, Chitinase-C enzyme, Chitinase-A enzyme, Aminopeptidase, Calpain-1 catalytic subunit-like protein	Mucus, Tentacles	Mexico	2019	[200]
Bunodactis verrucosa	Actiniaria	SE-cephalotoxin, Basic phospholipase A2 vurtoxin, Alpha-latroinsectotoxin-Lt1a, Zinc metalloproteinase/disintegrin, Neprilysin-1	Whole Body	Portugal	2018	[201]

Bunodosoma caissarum	Actiniaria	U-actitoxin-Bcs3d , U-actitoxin-Bcs3a, U-actitoxin-Bcs2c, K-actitoxin-Bcs4m, U-actitoxin-Bcs2a, Bcs Tx3, K-actitoxin-Bcs2a, KΠ-actitoxin-Bcs3e, Π-actitoxin-Bcs2e, U-actitoxin-Bcs3n, Π-actitoxin-Bcs3a, U-actitoxin-Bcs12a, Turripeptide Pal 9.2-like, Π-actitoxin-Bcs5a, Basic phospholipase A2 pseudexin A chain-like, K-actitoxin-Bcs1a, K-actitoxin-Bcs3a, U-actitoxin-Bcs8a, Δ-actitoxin-Bcs2a, Zinc metalloproteinase nas-13-like, Zinc metalloproteinase nas-4-like, Zinc metalloproteinase nas-15-like, Cystatin	Mucus, Tentacles	Brazil	2023	[202]
Bunodosoma cangicum	Actiniaria	81 peptide components, including Bcg 21.00 (Sea anemone BBH family), Bcg 21.75 (Sea anemone BBH family), Bcg 23.41 (Sea anemone BBH family), Bcg 25.52 (BcIV- and APETx-like), Bcg 25.52 (BcIV- and APETx-like), Bcg 25.96 / Bc III (Type I NaTx), Bcg 28.78 (BcIV- and APETx-like), Bcg 29.21 (BcIV- and APETx-like), Bcg 30.24 (Type I NaTx), Bcg 31.16 (BcIV- and APETx-like)	Milked Venom	Brazil	2008	[59]
Bunodosoma granuliferum	Actiniaria	156 peptide components, including Bg 16.07a / BgK (Type I KTx (subfamily 1b), Bg 26.91a / Bg II (Type I NaTx), Bg 26.91b / Bg III (Type I NaTx), Bg 30.66b / Bg1a (Type III KTx), Bg 32.67 / Bg1b (Type III KTx), Bg 34.22 / Bg1d (Type III KTx), and others paralyzing and lethal to crabs (2000 μg/kg)	Mucus	Cuba	2012	[130]
Calliactis polypus	Actiniaria	Knottin-like, KTx type I (ShK-Cpp3a), KTx type I (ShK-Cpp2a), KTx type I (ShK-Cpp1a), KTx type I (ShKC10-Cpp1a), NaTx (calitoxin-Cpp1a), PLA2-Cpp1a, PLA2-like, Sea Anemone structural class 9 (SA9-Cpp1a), Unknown C6, Unknown C6, Unknown C6, Unknown C6, Unknown C6 (NaH-Cpp1a), Unknown C8	Milked venom	Australia	2024	[144]
Calliactis polypus	Actiniaria	Lectin Fincolin, Phospholipase A2 (PLA2-Cpp1a), Disintegrin and metalloprotease, Peptidase M12A, Multicopper oxidase, Type I (ShK) Potassium channel toxin (ShKC10-Cpp1a), Type III Potassium channel toxin, Calitoxin (caliatoxin-Cpp1a), Sea anemone sodium channel toxin, Sea anemone sodium channel toxin Type I, Sea anemone structural class type 8, Unknown (12C)	Acontia	Australia	2023	[38]
Epiactis japonica	Actiniaria	AnmTX Cj 1a-1 (NaTX Type II), AnmTX Cj 1b-1 (KTX type III), AnmTX Cj 1c-1 (NaTX Type I), AnmTX Cj 3a-1 (Kunitz-type), AnmTX Cj 3a-2 (Kunitz-type), AnmTX Cj 6a-1, AnmTX Cj 8a-1, AnmTX Cj 8a-2, CjTL 1, CjTL 2, CjTL 3, CjTL 4, CjTL 5, CjTL 6, CjTL 7, CjTL 8, CjTL 9	Milked venom	Russia	2017	[93]
Entacmaea quadricolor	Actiniaria	Actinoporin (Type II cytolysins), BßH-like (Type IV Kv channel), Coagulation factor V-like, CREC, CRISP, DELTA-actitoxin-Ucs1a, DELTA-alicitoxin-Pse2a-like, DELTA-alicitoxin-Pse2b-like, DPP IV/FAP, EGF-like, Ficolin lectin family, Immunoglobulin-like, NEP 3 Family, Peptidase M12A, Peptidase S1, Phospholipase A2, ShK-like (Type I Kv channel), True venom lectin family, U12, U15, U16, uncharacterised toxins, Venom Kunitz-type family, Z3, Z7	Milked venom	n.a.	2024	[203]
Radianthus magnifica	Actiniaria	unigene000172_i1 (metalloproteinase), unigene000903_i1 (SHK), unigene001065_i1 (Glycosyl hydrolase), unigene002839_i1 (F5_F8_type_C), unigene003824_i6 (CAP), unigene003941_i2 (Carbon-nitrogen hydrolase), unigene004770_i1 (Peptidase family M12A), unigene006504_i2 (EGF), unigene007157_i2 (Proprotein convertase), unigene007246_i1 (ICK), unigene007561_i1 (Peptidase_M2), unigene008831_i1 (Thrombospondin), unigene009208_i1 (Proprotein convertase), unigene010382_i4 (Laminin), unigene010558_i2 (Cadherin), unigene011578_i7 (Peptidase family M12A), unigene014195_i1 (Gly_rich), unigene014939_i1 (CAP), unigene015828_i1 (Collagen), unigene019494_i1 (Asp), unigene020284_i1 (Fibrinogen), unigene021507_i3 (Glycosyl hydrolase), unigene022826_i1 (Defensin), unigene024628_i1 (DnaJ domain), unigene026007_i2 (Peptidase family M12A), unigene026432_i1 (Cathepsin propeptide inhibitor), unigene029734_i1 (Defensin), unigene031062_i1 (Peptidase_M2), unigene031663_i1 (Collagen), unigene032282_i1 (Thioredoxin), unigene035332_i1 (Defensin), unigene036749_i1 (SHK), unigene036854_i1 (Thioredoxin), unigene036954_i4 (Collagen), unigene039394_i8 (Peptidase_M2), unigene039955_i3 (Copper type II ascorbate-dependent monooxygenase), unigene041192_i2 (CAP), unigene046010_i1 (EGF), unigene046066_i1 (SHK), unigene047449_i1 (SHK), unigene049641_i1 (Laminin), unigene051039_i1 (Defensin), unigene051740_i1 (SHK), unigene052379_i17 (SHK), unigene052380_i1 (SHK), unigene053434_i1 (UDPGT), unigene053529_i1 (Cupin_8), unigene054598_i2 (Heme-binding protein), unigene056047_i3 (Disintegrin), unigene059953_i1 (Cathepsin propeptide inhibitor), unigene061072_i2 (Proprotein convertase), unigene064856_i1 (Peptidase_C1), unigene066312_i1 (VWA), unigene067741_i1 (Cathepsin propeptide inhibitor), unigene067940_i12 (SHK), unigene068507_i11 (Defensin), unigene068864_i16 (Thrombospondin), unigene070716_i4 (Fibronectin), unigene071105_i1 (Defensin), unigene072292_i1 (Disintegrin), unigene072318_i1	Tentacles, Column	China	2024	[204]

		(Kunitz), unigene072362_i1 (Collagen), unigene073914_i1 (Thioredoxin), unigene075130_i3 (CUB domain), unigene076082_i1 (Fibronectin), unigene077800_i1 (Asp), unigene078763_i1 (Immunoglobulin), unigene080720_i1 (Proprotein convertase), unigene080816_i3 (Defensin), unigene081210_i1 (SHK), unigene082025_i1 (Laminin), unigene083146_i6 (Peptidase_C13), unigene083730_i1 (Thioredoxin), unigene084965_i2 (MAM domain), unigene085054_i2 (Glycosyl hydrolase), unigene085398_i7 (SHK), unigene085880_i3 (SHK), unigene086439_i1 (Trypsin), unigene087148_i1 (SHK), unigene087432_i1 (Kazal), unigene087469_i1 (Fibronectin), unigene089522_i9 (Neurotensin receptor), unigene091141_i1 (SHK), unigene091350_i1 (Peptidase_S28), unigene092392_i1 (Fibronectin), unigene092846_i1 (Fibronectin), unigene093259_i9 (HMg III), unigene096015_i1 (EGF), unigene096907_i1 (CUB domain), unigene097141_i1 (Defensin), unigene099576_i1 (Cathepsin propeptide inhibitor), unigene101323_i1 (metalloproteinase), unigene101323_i2 (metalloproteinase), unigene102076_i1 (SHK), unigene104936_i1 (EGF), unigene110700_i1 (SHK), unigene112583_i2 (domain-containing protein), unigene113731_i1 (SHK), unigene113782_i1 (Glycosyl hydrolase), unigene113819_i1 (Peptidase family M12A), unigene113898_i1 (Immunoglobulin), unigene115719_i1 (Kazal), unigene116358_i1 (SHK), unigene118461_i4 (Peptidase family M12A), unigene118739_i1 (Trypsin), unigene119711_i1 (Glycosyl hydrolase), unigene122106_i1 (CAP), unigene124634_i1 (Kazal), unigene126675_i1 (DnaJ domain), unigene128009_i2 (Peptidase family M12A), unigene130393_i3 (Gly_rich), unigene132705_i1 (EGF), unigene132719_i1 (Defensin), unigene132816_i1 (Kunitz), unigene132816_i5 (Kunitz), unigene137144_i2 (Glycosyl hydrolase), unigene138905_i1 (FG-GAP repeat profile), unigene142870_i4 (SHK), unigene143278_i3 (Defensin), unigene145221_i1 (WSC), unigene146403_i1 (SHK), unigene146642_i1 (Thioredoxin), unigene149215_i5 (DnaJ domain), unigene153225_i1 (Transferrin), unigene155692_i1 (Carbon-nitrogen hydrolase), unigene157927_i2 (VWA), unigene159316_i3 (Thioredoxin), unigene162331_i1 (SHK), unigene164626_i1 (ICK), unigene165246_i1 (Kazal)				
Nematostela vectensis	Actiniaria	NEP-1 (2 Endoglucanase C-terminal domain(COG4305); Chitin binding Peritrophin-A domain (PF01607)), NEP-2 (RNA polymerase sigma-B factor (TIGR02941)), NEP-3, NEP-4, NEP-5 (Galactose binding lectin domain (PF02140)), NEP-6 (Astacin (PF01400)), NEP-7, NEP-8, NEP-9 (Collagen triple helix repeat (PF01391). Galactose binding lectin domain (PF02140)), NEP-10, NEP-11 (Cyclophilin type peptidyl-prolyl cis–trans isomerase (PF00160)), NEP-12, NEP-13 (Inosine-uridine preferring nucleoside hydrolase (PF01156)), NEP-14 (Astacin (PF01400)), NEP-15 (PLAT/LH2 (PF01477)), NEP-16 (CAP, Cysteine-rich secretory protein family (PF00188)), NEP-17 (CAP, Cysteine-rich secretory protein family (PF00188)), NEP-18 (Inosine-uridine preferring nucleoside hydrolase (PF01156)), NEP-19 (2 EGF-like domains (PF00008)), NEP-20 (CAP, Cysteine-rich secretory protein family (PF00188))	Nematocysts	n.a.	2013	[41]
Homostichanthus duerdeni	Actiniaria	134 peptide components, including Analgesic polypeptide HC1 (APHC1), Nigrescease-1, Potassium channel toxin SHTX-1/SHTX-2, Putative beta-tubulin (fragment), rRNA processing protein RRP7, U-SHTX-Sdd/U-HMTX-Hdu1a	Mucus	Brazil	2013	[60]
Stichodactyla haddoni	Actiniaria	ASIC, Cytolysin type2, Cytolysin type3, EGF-like sea anemone, ICK - Unknow, Kv type1, Kv type2, Kv type3, Kv type4, Kv Type5, Nav type1, Nav type2, Prothrombin activator, TRPV1, TRPV2, Venom allergen 5	Nematocysts	Australia	2017	[205]
Stichodactyla helianthus	Actiniaria	113 peptide components, including Sh 17.55/ ShPI-2, Sh 27.26/ ShI and others paralyzing and lethal to crabs (2000 μg/kg)	Mucus	Cuba	2012	[130]
Telmatactis stephensoni	Actiniaria	Peptidase M12A (3), Sea anemone 8 toxin (2), Sea anemone structural class 9a (1), Sea anemone type 1 potassium channel toxin /ShKT-Ts1, type 1a subfamily (1), Venom Kunitz-type family, sea anemone type 2 potassium channel toxin (2)	Milked venom	Australia	2022	[65]
Phymanthus crucifer	Actiniaria	504 peptide components within the 1-10 kDa range	Mucus	Cuba	2012	[206]
Radianthus magnifica	Actiniaria	134 peptide components, including RpII, HMGS2, HMGS3c1, HMGS3c4, HMGS1, HMGS3c6, HMGS3c9, HMRG1	Mucus	Vietnam	2018	[173]
Carybdea brevipedalia	Cubozoa	Potassium channel toxin TtrKIK, Toxin CrTX-A, Toxin CSTX-18, Dermonecrotic toxin LruSicTox-alphaIC1d, Eumenine mastoparanER, Non-disulfide-bridged peptide 5.6, Kappa-thalatoxin-Cad2a, Cysteine-rich venom protein 2	Nematocysts	South Korea	2025	[207]

Chironex fleckeri	Cubozoa	Toxin B precursor (CfTX-B), Toxin A precursor (CfTX-A), Toxin CfTX-1 precursor, Toxin CfTX-2 precursor, Predicted protein Nematostella vectensis (Metalloprotease), Metalloendopeptidase, Metalloproteases, Predicted protein	Nematocysts	Australia	2015	[208]
Chironex fleckeri	Cubozoa	Astacin-like metalloprotease toxin, Blarina toxin, Cysteine-rich venom protein latiseimn, Cysteine-rich venom protein psededechetoxin-like, Peroxiredoxin, Peroxiredoxin-4, Toxin AaTX-1-like, Toxin CfTX-1, Toxin CfTX-1-like, Toxin CfTX-2, Toxin CfTX-2-like, Toxin CfTX-A, Toxin CfTX-A-like, Toxin CfTX-B, Toxin CqTX-A-like, Turripeptide, Venom dipeptidyl peptidase 4, Venom factor (Crotalus adamanteus), Venom protease (Megabombus pennsylvanicus), Zinc metalloproteinase nas-15, Zinc metalloproteinase nas-4	Nematocysts	Australia	2015	[209]
Chironex indrasaksajiae	Cubozoa	Allene oxide synthase-lipoxygenase protein-like, Alpha-xenorhabdolysin family binary toxin subunit B, Ankyrin repeat and SAM domain-containing protein 1A, Brain-derived neurotrophic factor, Ca2+-binding protein, RTX toxin-related, Calcium binding hemolysin protein, Calmodulin, Chain B, Proteasome subunit alpha type-2, Colicin-like pore-forming protein, Cytotoxin (Fragment), DNA protection during starvation protein, Delta-latroinsectotoxin-Lt1a, Disintegrin and metalloproteinase domain-containing protein, Enoyl-CoA hydratase, Fibronectin-binding outer membrane protein CadF, Flp pilus assembly complex ATPase component TadA, Hemolysin A, Hemolysin-type calcium-binding protein repeat protein, Heterotepalin-4, Maltoporin, Metalloendopeptidase, Mitogen-activated protein kinase, Outer membrane porin A, Outer membrane porin A-F, Outer membrane porin A-like, Outer membrane porin C, Outer membrane porin K36, Outer membrane porin N, Peptidase C80, Peptidase C81, Peptidase M12B domaincontaining protein, Peroxiredoxin-4, Phosphoporin PhoE, Phosphopyruvate hydratase, Polycystic kidney disease protein 1-like 2, Pore-forming outer membrane protein, Porin, rotein CyaE, Putative RTX-toxin (Fragment), Putative ankyrin, Putative toxin VapC, Rab GDP dissociation inhibitor, Serine/threonine-protein phosphatase PP1-beta, Thrombospondin type 3 repeat-containing protein, TonB-dependent receptor, Toxin CdiA; Haemagg_act domain-containing protein, Toxin CdiA; Putative hemagglutinin, Toxin CfTX-1, Toxin CfTX-2, Toxin CfTX-A, Toxin CfTX-B, Toxin CqTX-A, Toxin VapC, Transient receptor potential channel A1 isoform a, TX-like toxin, Type II secretion outermembrane pore forming protein (PulD), Voltage-dependent anionselective channel protein 3, V-type proton ATPase 16 kDa proteolipid subunit, Zinc metalloproteinase nas-15	Cnidae	Thailand	2021	[210]
Chiropsalmus quadrumanus	Cubozoa	Alpha-latroinsectotoxin-Lt1a, Conotoxin Bu2, Echotoxin-2, Hainantoxin-XVIII-5, Neurotoxin LmNaTx1, Toxin CfTX-2	Nematocysts	Brazil	2017	[211]
Tamoya haplonema	Cubozoa	Alpha-latroinsectotoxin-Lt1a, Conotoxin Lt5.9, DELTA-alicitoxin-Pse2b, Disintegrin acostatin-alpha, Echotoxin-2, Equinatoxin-3, Im-conomarphin, Maximins 3/H2, Phospholipase A2 3, Phospholipase D LlSicTox-alphaIII2, Potassium channel toxin alpha-KTx Tx773, Potassium channel toxin TdiKIK, Snake venom metalloproteinase aculysin-1, U5-ctenitoxin-Co1a, Venom allergen 5, Venom carboxylesterase-6, Venom nerve growth factor 1	Nematocysts	Brazil	2017	[211]
Ectopleura crocea	Hydrozoa	A. superbus venom factor 1, Alpha-latrocrustotoxin-Lt1a, Alpha-latroinsectotoxin-Lt1a, Alpha-latrotoxin-Lh1a, Alpha-latrotoxin-Lhe1a, Bradykinin-potentiating and C-type natriuretic peptides, Cobra venom factor, C-type natriuretic peptide, DELTA-alicitoxin-Pse2b, Natriuretic peptide, Natriuretic peptide BM026, Phospholipase D LhSicTox-alphaIIIli, Phospholipase D LiSicTox-betaIIB1bx, Protein C activator, Serine protease harobin, Snake venom serine protease 2, Stonustoxin subunit alpha, Venom allergen 5, Venom metalloproteinase antarease TserMP_A, Venom prothrombin activator pseutarin-C catalytic subunit, Zinc metalloproteinase-disintegrin-like EoVMP2, Zinc metalloproteinase-disintegrin-like VLAIP-B	Nematocysts	Brazil	2019	[212]
Hydra magnipapillata	Hydrozoa	putative – ShKT (50 kda hatching enzyme-like), lectin, lectin, disintegrin (disintegrin and metalloproteinase domain-containing protein 24- partial), putative - C type lectin and ShKT (galactose-specific lectin nattectin-like), hemolysin – actinoporin (hemolytic toxin), putative – MACPF (mac perforin domain containing protein), C type lectin (macrophage mannose receptor 1-like), putative – MACPF (membrane attack complex perforin domain-containing protein), putative – ShKT (meprin a subunit beta-like), putative – ShKT (nematoblast-specific protein nb082), PLA2 (phospholipase a2), putative – ShKT (PREDICTED: uncharacterized protein LOC100200257, partial), putative – ShKT (protein), putative –	Nematocysts	n.a.	2015	[199]

		MACPF (sporozoite protein with mac perforin domain), hemolysin – CaTX (toxin -a-like), putative – ShKT (zinc metalloproteinase nas-15-like)				
Millepora alcicornis	Hydrozoa	DELTA-actitoxin-Aeq1b, Toxin CrTX-A, Zinc metalloproteinase	Nematocysts	Mexico	2023	[213]
Millepora complanata	Hydrozoa	Acidic calcium-independent phospholipase A2-like protein, Acidic PLA2 PA4, DELTA-actitoxin-Oor1b, Echotoxin-2, PREDICTED: Astacin-like metalloprotease toxin 5	Nematocysts	Mexico	2019	[214]
Olindias sambaquiensis	Hydrozoa	Alpha-conotoxin TxID, Calglandulin, Cathelicidin-related peptide Na_CRAMP, Conoporin-Cn1, Conotoxin Cal6.1e, Conotoxin De13.1, Conotoxin Gla-TxX, Cysteine-rich venom protein, DELTA-actitoxin-Aas1a (Bandaporin), Golgi-associated plant pathogenesis-related protein 1, Hyaluronidase 2, Kunitz-type serine protease inhibitor bitisilin-3, Lysosomal aspartic protease, Omega-conotoxin-like 3, Phosphodiesterase, Phospholipase A2, Phospholipase A2 PLA2-I, Phospholipase D LcsSicTox-betaIC1, Phospholipase D LlSicTox-alphaIII3i, Phospholipase D LlSicTox-alphaIV1i, Phospholipase D LvSicTox-alphaIC1bii, Potassium channel toxin alpha-Ktx, Potassium channel toxin alpha-KTx 2.14, Putative lysosomal acid lipase/cholesteryl ester hydrolase, Putative potassium channel blocker TXKS1, Snake venom metalloproteinase ACLF, Snake venom serine protease 1, Three-finger toxin 3, U7-lycotoxin-Ls1e, U-actitoxin-Avd11a, Venom serine carboxypeptidase	Nematocysts	Brazil	2019	[215]
Olindias sambaquiensis	Hydrozoa	Acanmyotoxin-3, Acrorhagin-1, Aculysin-1, a-Latrocrustotoxin-Lt1a, Bothrojaractivase, Butantoxin-like peptide, Cytotoxic linear peptide, k -Theraphotoxin-Cj1b, k-4-Bungarotoxin, Metalloprotease, Metalloproteinase, Natriuretic peptide Oh-NP, Neublin-like protein, Phospholipase A2, Phospholipase A2 isozyme 1, Phospholipase A2 isozyme Ts-A1, Prepro-halystatin, Serine protease HS112, Serine proteinase 8, Sphingomyelin phosphodiesterase D, Toxin AvTX-60A, Toxin Gkn9.1, Toxin PsTX-60A, Turritoxin UID-02, U14-Theraphotoxin-Cj1b, Venom allergen 5, Venom allergen 5.01, Venom dipeptidylpeptidase IV, Venom serine protease 34	Nematocysts	Brazil	2013	[216]
Velella velella	Hydrozoa	25 kDa ookinete surface antigen, A disintegrin and metalloproteinase, Alpha-elapitoxin-Dpp2a, Alpha-mannosidase-like, Aminopeptidase 1-like, Aminopeptidase N, Angiotensin-converting enzyme, Antigen 5 like allergen Cul-n-1, Beta-hexosaminidase, C5a peptidase, CAAX prenyl protease 1, Carboxypeptidase B-like, Carboxypeptidase D-like, Cation-independent mannose-6-phosphate receptor, Chitinase, Chitooligosaccharidolytic beta-N-acetylglucosaminidase, Chitotriosidase-1, Coagulation factor FVIII-Fc-XTEN, Coagulation factor V, Coagulation factor VIII, Complement C1q and TNF-related protein 9B-like, Cry1K-like protein chimera, D-galactoside-specific lectin-like, Diphosphonucleotide phosphatase-1, Ectonucleoside triphosphate diphosphohydrolase-2, Endonuclease/exonuclease/phosphatase family protein, FBP32 f-lectin, Furin-like protease 1, Gelatinase, Group XIIA secretory phospholipase A2-like, Hemagglutinin/amebocyte aggregation factor-like, Hemolectin, putative, Kappa-stichotoxin-She3a, Kunitz-type protease inhibitor-1, Lectin-2, Long neurotoxin OH-55, L-rhamnose-binding lectin CSL3-like, Ly-6/neurotoxin-like protein-1, Macrophage mannose receptor 1, Maltose-binding periplasmic protein, Merozoite surface protein-1, N-acetylated-alpha-linked acidic dipeptidase 2-like, N-acyl-phosphatidylethanolamine-hydrolyzing phospholipase D-like, Neprilysin-4 isoform X2, Neutrophil cytosol factor-2-like, Non-lysosomal glucosylceramidase-like, Pappalysin1, Pesticidal crystal protein Cry11Ba, Phospholipase D1-like, PI-Phospholipase-C X domain-containing protein 3-like, Properdin, Pro-X carboxypeptidase, Puromycin-sensitive aminopeptidase-like, ShKT peptide ShKT-Ts1, SUEL-type lectin, TatD hydrolase, Trypsin-1 isoform X3-like, Urokinase plasminogen activator, XaxB pore forming toxin, Zinc metalloproteinase nas-13-like	Nematocysts	Italy	2024	[217]
Buddenbrockia plumatellae	Myxozoa	Venom allergen, Snaclec 7, U-actitoxin-Avd3n	Whole Body	Switzerland	2021	[218]
Ceratonova shasta	Myxozoa	ATP synthase alpha/beta family, nucleotide-binding domain, Actin, C-terminus of histone H2A, Collagen triple helix repeat (20 copies), Concanavalin A-like lectin/glucanases superfamily, Cyclophilin type peptidyl-prolyl cis-trans isomerase/CLD, Cystatin domain, Cysteine-rich secretory protein family/Peptidase inhibitor-16, Elongation factor Tu GTP binding domain, F5/8 type C domain/Lactadherin, FKBP-type peptidyl-prolyl cis-trans isomerase, Galactose	Whole Body	n.a.	2021	[219]

		binding lectin domain, Gamma-glutamyltranspeptidase, Histidine phosphatase superfamily (branch 2), Hsp70 protein, Lectin C-type domain, PLAT/LH2 domain/Lactadherin, Peptidase family M1 domain, Peptidase family M28, ShK domain-like, Thioredoxin, Zinc carboxypeptidase				
Sphaerospora elegans and Myxobilatus gasterostei	Myxozoa	Blarina toxin	Whole Body	United Kingdom	2021	[218]
Eunicella singularis	Octocorallia	PI-GRTX-Esi10a (2 x Kazal), PI-GRTX-Esi10b (2 x Kazal), PI-GRTX-Esi11 (Cystatin), PI-GRTX-Esi12 (4 x Antistasin, Kazal), PI-GRTX-Esi5 (2 x kunitz), PI-GRTX-Esi6 (kunitz), PI-GRTX-Esi7a (Kazal), PI-GRTX-Esi7b (Kazal), PI-GRTX-Esi7c (Kazal), PI-GRTX-Esi8 (2 x EGF-like, 2 x Kazal), PI-GRTX-Esi9 (Kazal), U-GRTX-Esi13 (Macin), U-GRTX-Esi14 (Colipase), U-GRTX-Esi15a (Colipase), U-GRTX-Esi15b (Colipase), U-GRTX-Esi15c (Colipase), U-GRTX-Esi16 (Colipase), U-GRTX-Esi17 (Colipase), U-GRTX-Esi18 (Novel), U-GRTX-Esi19 (Novel), U-GRTX-Esi1a (octo-SCRiP), U-GRTX-Esi20 (Alpha-hairpin), U-GRTX-Esi21 (Alpha-hairpin), U-GRTX-Esi22 (Novel), U-GRTX-Esi23 (Novel), U-GRTX-Esi24 (Novel), U-GRTX-Esi25 (ShK + helical), U-GRTX-Esi26 (Novel), U-GRTX-Esi27 (BDS-like), U-GRTX-Esi28 (Novel), U-GRTX-Esi2a (octo-SCRiP), U-GRTX-Esi2b (octo-SCRiP), U-GRTX-Esi3 (2 x octo-SCRiP), U-GRTX-Esi32a (Saposin), U-GRTX-Esi32b (Saposin), U-GRTX-Esi32c (Saposin), U-GRTX-Esi32d (Saposin), U-GRTX-Esi32e (Saposin), U-GRTX-Esi32f (Saposin), U-GRTX-Esi4 (3 x ShK), Δ-GRTX-Esi29 (Aerolysin), Δ-GRTX-Esi30 (Actinoporin), Δ-GRTX-Esi31 (Actinoporin)	Nematocysts and Whole Body	Italy	2025	[220]
Nephthea sp	Octocorallia	Gel chromatography-separated fractions	Nematocysts	Egypt	2002	[221]
Dendronephthya sp	Octocorallia	Gel chromatography-separated fractions	Nematocysts	Egypt	2002	[221]
Heteroxenia fuscescens	Octocorallia	Gel chromatography-separated fractions	Nematocysts	Egypt	2002	[221]
Polypodium hydriforme	Polypodiozoa	Aspartyl protease, C-type lectin, Enzyme inhibitor, Glutaminyl-peptide cyclotransferase, Glycoside hydrolase, Hyaluronidase, Kunitz-type serine protease inhibitor stephenin-1, L-amino-acid oxidase, Lipase, Metalloprotease, Metalloprotease (Astacin-like toxin), Metalloprotease (Nematocyte expressed protein 6), Phospholipase A2 (Conodipine-M), Phospholipase-B, Plancitoxin-1, Prothrombin activator (Notecarin), Serine-type endopeptidase (Blarina toxin), Snaclec 7, Snake venom 5'-nucleotidase, Venom allergen	Whole Body	United States of America	2021	[218]
Acropora digitifera	Scleractinia	Snaclec 7, Serine carboxypeptidase, Phospholipase A2 activation (Reticulocalbin-2), Translationally-controlled tumor protein homolog, Equinatoxin-5 (Actinoporin), Calglandulin, Serine type endopeptidase, Δ-ALTX-Pse, Phospholipase B, Metalloprotease (Astacin-like toxin)	Nematocysts	Australia	2015	[222]
Heliofungia actiniformis	Scleractinia	Hact-1	Nematocysts	Australia	2020	[179]
Heliofungia actiniformis	Scleractinia	Hact-1, Hact-2, Hact-3, Hact-4, Hact-SCRiP1	Nematocysts	Australia	2022	[165]
Stylophora pistilata	Scleractinia	annexin A6-like, beta-catenin, caspase 3-like protein, gelsolin, golgi-associated plant pathogenesis-related protein 1-like, Hypothetical Protein (Myosin 10 like), hypothetical protein (peroxiredoxin-1), hypothetical protein (Polysaccharide deacetylase-like protein), Hypothetical protein (serine hydroxymethyltransferase, mitochondrial), iron/zinc purple acid phosphatase-like protein, nuclear receptor coactivator 5-like, predicted protein (catenin alpha-2-like isoform X4), predicted protein (Glucosamine-6-phosphate isomerase 1), predicted protein (glutamate dehydrogenase), protein disulfide-isomerase A6, Sticholysin 2, transmembrane protease serine 6-like, uracil-DNA glycosylase	Coral Fragment	Israel	2018	[149]
Tubastraea coccinea	Scleractinia	Alpha-conotoxin, Alpha-latrotoxin-Lh1a, Basic phospholipase A2 pseudexin A chain, Calglandulin, Coagulation factor X isoform 2, Conotoxin TsMLKM-02, Hyaluronidase A, L-amino-acid oxidase, Metalloproteinase, Peroxiredoxin-4, Phospholipase D SpaSicTox-betaIF1, Stonustoxin subunit-a, Three finger toxin, Venom allergen 5, Waprin-Phi1, X-theraphotoxin-Hs1a, Zinc metalloproteinase-disintegrinlike ACLD	Nematocysts	Brazil	2019	[212]

Tubastraea coccinea	Scleractinia	Basic phospholipase A2, Calglandulin, Disintegrin, Endothelin-converting enzyme 1, Flavoxobin, Gigantoxin-4, K+ channel toxin α-KTx 4.2, Phosphodiesterase, Phospholipase D, Snaclec 7, Stonustoxin subunit-α, Waprin-Phi1, α-Latrotoxin-Lhe1a, Ω-theraphotoxin-Hs1a	Nematocysts	Brazil	2020	[223]
Pseudodiploria strigosa	Scleractinia	Peptides within the 3-6 kDa range in low-molecular-weight fractions	Nematocysts	Mexico	2016	[224]
Porites astreoides	Scleractinia	Peptides within the 3-6 kDa range in low-molecular-weight fractions	Nematocysts	Mexico	2016	[224]
Siderastrea siderea	Scleractinia	Peptides within the 3-6 kDa range in low-molecular-weight fractions	Nematocysts	Mexico	2016	[224]
Aurelia aurita	Scyphozoa	Toxin - Av120, cytolysin – CaTX (toxin -a-like), cytolysin – CaTX (toxin partial), PLA2 (group 3 secretory phospholipase a2), putative – MACPF (vitelline membrane outer layer protein 1 homolog), putative – MACPF (membrane attack complex perforin domain-containing protein), putative - ShKT (hypothetical protein EMIHUDRAFT_106738), putative - ShKT (hypothetical protein CAPTEDRAFT_201069), putative – ShKT (apical endosomal glycol), putative – ShKT (zinc metalloproteinase nas-15-like), putative - ShKT (pr-1 like protein), putative - ShKT (astacin protein), putative - ShKT (protein isoform b)	Nematocysts	Gulf of Eilat	2015	[199]
Chrysaora caliparea	Scyphozoa	Calmodulin, Chrysaoralin, Snake venom metalloproteinase BnP1, Snake venom metalloproteinase BnP2, Snake venom metalloproteinase leucurolysin-A, TX-1 toxin, Zinc metalloproteinase	Nematocysts	India	2021	[225]
Chrysaora fuscescens	Scyphozoa	Aminopeptidase N-like, aminopeptidase-like Cathepsin D, Angiotensin-converting enzyme, Angiotensin-converting enzyme-like isoform, Aspartyl, Aspartyl protease, beta-hexosaminidase subunit alpha-like isoform X1, Carboxypeptidase D-like, Cell wall protein PRY3-like, Endoplasmic reticulum aminopeptidase 1-like, Endothelin-converting enzyme 1-like, Endothelin-converting enzyme 2-like, Golgi-associated plant pathogenesis-related protein 1, PC3-like endoprotease variant B isoform X1, PRY2-like protein, Toxin TX2, Tripeptidyl-peptidase 1-like, Uncharacterized protein LOC105843890	Nematocysts	United States of America	2016	[226]
Chrysaora lactea	Scyphozoa	Cathelicidin-related peptide Na_CRAMP, CfTX-2, Conotoxin Bu2, Cysteine-rich venom protein LIO1, L-amino-acid oxidase, M-zodatoxin-Lt4a, Snake venom serine protease KN2, Toxin CfTX-2, U16-lycotoxin-Ls1a, Venom peptide Ocy2	Nematocysts	Brazil	2017	[211]
Cyanea capillata	Scyphozoa	A disintegrin and metalloproteinase with thrombospondin motifs 6-like, A.superbus venom factor 1, Acidic phospholipase A2, Acidic phospholipase A2 homolog textilotoxin C chain, Alpha-latroinsectotoxin-Lt1a, Alpha-toxin BmalphaTx47, Cystatin, Basic phospholipase A2 homolog, Bitiscystatin, Cathelicidin-related peptide Pt_CRAMP1, Cholecystoxin, Chymotrypsinogen B-like, Conotoxin Vc6.16, C-type lectin lectoxin-Lio1, Cystatin-like, Cysteine-rich venom protein, Cytotoxin 11, Delta-latroinsectotoxin-Lt1a, DELTA-miturgitoxin-Cp1a, Dinoponeratoxin, Hainantoxin-XV-2, Kunitz-type U1-uranetoxin-Av1a, Long neurotoxin 1, Mitogen-activated protein kinase 1-like, Neurotoxin alpha-kTX 12.6, Omega-hexatoxinHm01c, Ophiophagus venom factor, Oxyuranus microlepidotus, Phospholipase A1, Phospholipase A2, Phospholipase D, Phospholipase D LspISicTox-betaIE 3i, Potassium channel toxin alpha-kTX J123, Scolopendra 20528.11 Da toxin, Serine protease 1, Serine protease K12H4.7, Thrombin-like enzyme, U-actitoxin-Avd9b, Venom allergen, Venom plasminogen activator TSV-PA, Venom serine carboxypeptidase, Zinc metalloproteinase disintegrin-like, Zinc metalloproteinase nas-13-like, Zinc metalloproteinase nas-15	Nematocysts	China	2018	[227]
Cyanea nozakii	Scyphozoa	A disintegrin and metalloproteinase with thrombospondin motifs 7, A disintegrin and metalloproteinase with thrombospondin motifs 7 isoform X2, A disintegrin and metalloproteinase with thrombospondin motifs 9-like, A.superbus venom factor 2, Acidic phospholipase A2 PA4, Alpha-latrocrustotoxin-Lt1a (Fragment), Phospholipase A2, Phospholipase A2 isozyme PA4, partial, Phospholipase A2 isozymes PA3A/PA3B/PA5, PLA2G12, Plancitoxin-1, Protease inhibitor PIVL, Serine protease inhibitor, Toxin (ATPase), Toxin TX2, partial, Venom dipeptidyl peptidase 4, Zinc metalloproteinase-disintegrin agkistin	Tentacles	China	2016	[228]
Cyanea nozakii	Scyphozoa	Calmodulin, Peroxiredoxin 4, Pp3, RACK-like protein, Serine protease inhibitor	Nematocysts	India	2021	[225]

Cyanea sp.	Scyphozoa	85/88 kDa calcium-independent phospholipase A2, A.superbus venom factor 1, ADMTM 14, ADMTM-6, Chymotrypsin-like elastase family member 2A, Cystatin-1, Galactose-specific lectin nattectin, Glutaminyl-peptide cyclotransferase-like protein, Kunitz-type serine protease inhibitor mulgin-2, L-amino-acid oxidase, Matrix metalloproteinase-25, Matrix metalloproteinase-7, Ophiophagus venom factor, Peroxiredoxin-4, Phospholipase A2, Phospholipase B1, Phospholipase D1, Putative protein-glutamate O-methyltransferase, Serine protease 23, Serine protease 30, Snake venom metalloproteinase-disintegrin-like mocarhagin, Translationally-controlled tumor protein homolog, Turripeptide Lol9.1, Venom allergen 3, Venom dipeptidyl peptidase 4, Venom factor, Venom prothrombin activator omicarin-C noncatalytic subunit, Venom prothrombin activator pseutarin-C noncatalytic subunit, Zinc metalloproteinase nas-14	Whole Body	Antarctica	2019	[229]
Lychnorhiza malayensis	Scyphozoa	332-1 secreted propeptide, Kininogen-1, Pp11 (Neuropeptide), Pp3 (Neuropeptide), Zinc metalloproteinase	Nematocysts	India	2021	[225]
Nemopilema nomurai	Scyphozoa	85/88 kDa calcium-independent phospholipase A2, A disintegrin and metalloproteinase with thrombospondin motifs 10, A disintegrin and metalloproteinase with thrombospondin motifs 10 fragment, A disintegrin and metalloproteinase with thrombospondin motifs 23, A disintegrin and metallo-proteinase with thrombospondin motifs 4, Allergen Mag (fragment), ATP-dependent Clp protease ATP-binding subunit ClpE, ATP-dependent zinc metalloprotease FtsH, ATP-dependent zinc metalloprotease YME1 homolog, Calcium-independent phospholipase A2-gamma, Cell death abnormality protein 1, Cell death abnormality protein 12, Disintegrin and metalloproteinase domain-containing protein 17, Heat-stable enterotoxin recepter, Hemolysin transporter, protein ShlB, Leukotoxin, Major pollen allergen Lol p 5a, Major serine/threonine-protein phosphatase PP2A_2 catalytic subunit, Phospholipase A-2-activating protein, Phospholipase D gamma 2, Phospholipase D LiSicTox-betaIDI, Pro-apoptotic serine protease nma111, Putative Kunitz-type serine protease inhibitor, RTX-I toxin determinant A from serotypes 1/9, RTX-III toxin determinant A from serotype 2, Serine protease HtrA-like, Shiga-like toxin 1 subunit A, Snake venom serine protease nikobin, Three-finger toxin MALT0044C, Tricorn protease-interacting factor F2, Turripeptide Gsp9.1, Venom prothrombin activator trocarin-D, Zinc metalloproteinase nas-8, Zinc metalloproteinase-disintegrin-like / Snake venom metalloproteinase	Nematocysts	South Korea	2019	[230]
Nemopilema nomurai	Scyphozoa	basic phospholipase A2 homologue, cytoinsectotoxin-4, dermonecrotic toxin StSicTox-betaIF1 (Fragment), disintegrin leucogastin-B, eumenine mastoparan-OD, kappa-stichotoxin-Shd1a/ kappa-stichotoxin-Shd1b, M-zodatoxin-Lt8j, reticulocalbin-2, teretoxin Tan6.8, thrombin-like enzyme calobin-2 (Fragment), U-scoloptoxin (16)-Sm3a, U-scoloptoxin (25)-Er1a, vespid chemotactic peptide M	Nematocysts	China	2020	[231]
Nemopilema nomurai	Scyphozoa	A disintegrin and metalloproteinase with thrombospondin motifs 3, A disintegrin and metalloproteinase with thrombospondin motifs 6, A disintegrin and metalloproteinase with thrombospondin motifs 9, Acidic phospholipase A2, Alpha-latrotoxin-Lh1a, Aminopeptidase 2, Antimicrobial peptide Xac-1, Broad-complex core protein isoforms 1/2/3/4/5, carboxypeptidase D-like, clip domain serine proteinase 2, Conotoxin like, CRISP/Allergen/PR-1, cystatin-B-like, Cysteine-rich venom protein LIO1, Galactose-specific lectin nattectin, Hyaluronidase 2, Hyaluronidase conohyal-P1, Insecticidal toxin LaIT1, Kunitz-type serine protease inhibitor dendrotoxin DaE1, L-rhamnose-binding lectin CSL2, Lysenin, MAC/Perforin domain containing protein, matrix metalloproteinase-16 isoform X3, metallophosphoesterase domain-containing protein 1, Microtubule-associated serine/threonine-protein kinase 2, M-zodatoxin-Lt8l, Neurotoxin Nk-3FTx, Phosphatidylinositide phosphatase SAC2, Phospholipase A2, Phospholipase D LrSicTox-alphaIA1i, PI-actitoxin-Aeq3b, Ponericin-G2, Potassium channel toxin alpha-KTx, Potassium voltage-gated channel protein Shaker, putative phospholipase B-like 2 isoform X1, Ryncolin-4, Serine protease inhibitor, serine/threonine-protein kinase dyrk2 isoform X1, Serine/threonine-protein phosphatase 5, Snaclec 2, snake venom metalloproteinase, Snake venom serine protease 3, Toxin CqTX-A, Toxin CrTX-A, Translationally-controlled tumor protein, Venom allergen 5, Venom protein 27.1, Zinc metalloproteinase nas-13, Zinc metalloproteinase nas-15, Zinc metalloproteinase nas-28, zinc metalloproteinase nas-8-like, zinc metalloproteinase-disintegrin bilitoxin-1, Zinc metalloproteinase-disintegrin BlatH1, zinc metalloproteinase-disintegrin jerdonitin, zinc metalloproteinase-disintegrin VMP-II, zinc metalloproteinase-disintegrin-like, zinc metalloproteinase-disintegrin-like atragin	Nematocysts	China	2018	[227]

Pelagia noctiluca	Scyphozoa	Zink Metalloproteinase, Peroxiredoxin	Whole Body	Portugal	2017	[232]
Phacellophora camtschatica	Scyphozoa	5'-nucleotidase domain-containing protein 1-like, 5'-nucleotidase-like, acetylcholinesterase, acidic PLA2 PA4, ADAM 10-like, ADAM 17-like protease, ADAMTS 19, ADAMTS 6, ATP-dependent zinc metalloprotease FTSH 4, mitochondrial, Basic PLA2 beta-bungarotoxin A-AL2 chain, coagulation factor X-like isoform X1, C-type lectin domain family 4 member M, Cystatin J, Cystatin-1, cystatin-A5-like, cystatin-B-like, epididymal secretory glutathione peroxidase-like, glutathione peroxidase 1-like isoform X2, glutathione peroxidase-like, gluthatione peroxidase, mannan-binding lectin serine protease 1 isoform X2, MMP 25-like, multiple coagulation factor deficiency protein 2-like isoform X2, N-acyl-phosphatidylethanolamine-hydrolyzing phospholipase D-like, patatin-like phospholipase domain-containing protein 7, peroxiredoxin-6, phospholipase A2 PA3A/PA3B/PA5, phospholipase B1, membrane-associated-like, phospholipase DDHD1-like, phospholipase DDHD2, PLA2, plancitoxin-1-like, putative phospholipase B-like 2, putative serine protease K12H4.7, putative zinc metalloproteinase, Ryncolin-4	Tentacle	n.a.	2022	[233]
Phyllorhiza punctata	Scyphozoa	ADAM 17-like protease, aflatoxin B1 aldehyde reductase member 2-like, agrin-like, BPTI/Kunitz inhibitor domain-containing protein, conodipine-P3-like, LOW-QUALITY PROTEIN: venom factor-like, plancitoxin-1-like toxin candidate, ras-related C3 botulinum toxin substrate 1, snake venom 5′-nucleotidase-like, toxin CfTX-A-like, toxin TX1, TPA_exp: toxin a, venom factor-like, zinc metalloproteinase-disintegrin-like MTP8	Gonads, Mantle, Oral Arms	n.a.	2025	[234]
Rhopilema esculentum	Scyphozoa	Acidic phospholipase A2 PA4 (PLA2) (EC 3.1.1.4) (phosphatidylcholine 2-acylhydrolase) (cleaved into acidic phospholipase A2 PA2), Astacin-like metalloprotease toxin 2 (EC 3.4.24.-) (Loxosceles astacin-like protease 2) (LALP2), Blarina toxin (BLTX) (EC 3.4.21.-), Calglandulin, Coagulation factor V (cleaved into coagulation factor V heavy chain and coagulation factor V light chain), C-type lectin lectoxin-Lio2 (CTL), Cystatin-1 (cystatin JZTX-75), Cysteine-rich venom protein pseudechetoxin-like (CRVP), Cysteine-rich venom protein pseudecin (CRVP Pdc), Delta-latroinsectotoxin-Lt1a (delta-LIT-Lt1a) (delta-latroinsectotoxin) (delta-LIT), Galactose-specific lectin nattectin (CTL), Glutaminyl-peptide cyclotransferase (EC 2.3.2.5) (glutaminyl cyclase) (QC) (glutaminyl-tRNA cyclotransferase), Kunitz-type serine protease inhibitor Hg1 (delta-KTx 1.1), Nematocyst-expressed protein 6 (NEP-6) (EC 3.4.24.-) (astacin-like metalloprotease toxin), Neprilysin-1 (EC 3.4.24.-), Phospholipase A2 isozymes PA3A/PA3B/PA5 (PLA2) (EC 3.1.1.4) (phosphatidylcholine 2-acylhydrolase), Plancitoxin-1 (EC 3.1.22.1) (plancitoxin I) (plan-I) (cleaved into plancitoxin-1 subunit alpha and plancitoxin-1 subunit beta), Reticulocalbin-2 (taipoxin-associated calcium-binding protein 49 homolog), Ryncolin-2, Ryncolin-4, Snaclec botrocetin subunit beta (platelet coagglutinin), Stonustoxin subunit beta (SNTX subunit beta) (DELTA-synanceitoxin-Sh1b) (DELTA-SYTX-Sh1b) (trachynilysin subunit beta) (TLY subunit beta), Translationally controlled tumor protein homolog (GTx-TCTP1), U-actitoxin-Avd3j (U-AITX-Avd3j) (AsKC7), Venom dipeptidyl peptidase 4 (allergen C) (venom dipeptidyl peptidase IV) (EC 3.4.14.5) (allergen Api m 5), Venom protease (EC 3.4.21.-) (allergen Bom p 4), Venom prothrombin activator pseutarin-C non-catalytic subunit (PCNS) (vPA) (venom coagulation factor Va-like protein) (cleaved into pseutarin-C non-catalytic subunit heavy chain and pseutarin-C non-catalytic subunit light chain), Venom prothrombin activator vestarin-D1 (vPA) (EC 3.4.21.6) (venom coagulation factor Xa-like protease) (cleaved into vestarin-D1 light chain and vestarin-D1 heavy chain), Venom serine carboxypeptidase (EC 3.4.16.5) (allergen Api m 9), Zinc metalloproteinase-disintegrin-like 3a (EC 3.4.24.-) (snake venom metalloproteinase) (SVMP), Zinc metalloproteinase-disintegrin-like MTP8 (EC 3.4.24.-) (snake venom metalloproteinase) (SVMP), Zinc metalloproteinase-disintegrin-like TSV-DM (EC 3.4.24.-) (snake venom metalloproteinase) (SVMP)	Nematocysts	n.a.	2020	[235]
Sanderia malayensis	Scyphozoa	Acetylcholinesterase (BfAChE) (EC 3.1.1.7), Acidic phospholipase A2 PA4 (PLA2) (EC 3.1.1.4) (phosphatidylcholine 2-acylhydrolase) (cleaved into acidic phospholipase A2 PA2), Alpha-latrotoxin-Lh1a (Alpha-LTX-Lh1a) (alpha-latrotoxin) (fragment), Astacin-like metalloprotease toxin 1 (EC 3.4.24.-) (Loxosceles astacin-like protease 1) (LALP) (LALP1), Astacin-like metalloprotease toxin 5 (EC 3.4.24.-) (Loxosceles astacin-like protease 5) (LALP5) (Fragment), Blarina toxin (BLTX) (EC 3.4.21.-), Coagulation factor V (cleaved into coagulation factor V heavy chain and coagulation factor V light chain), Coagulation factor X (EC 3.4.21.6) (cleaved into factor X light chain, factor X heavy chain, and activated factor Xa	Nematocysts	n.a.	2020	[235]

		heavy chain), C-type lectin (CTL), Cystatin-1, Cysteine-rich venom protein (CRVP) (cysteine-rich secretory protein) (CRISP), Galactose-specific lectin nattectin (CTL), Glutaminyl-peptide cyclotransferase (EC 2.3.2.5) (glutaminyl cyclase) (QC) (glutaminyl-tRNA cyclotransferase), Hyaluronidase (EC 3.2.1.35) (hyaluronoglucosaminidase) (venom-spreading factor), Kunitz-type serine protease inhibitor bitisilin-3 (two-Kunitz protease inhibitor) (fragment), Kunitz-type U19-barytoxin-Tl1a (U19-BATX-Tl1a) (Kunitz-type serine protease inhibitor Kunitz-1), l-amino-acid oxidase (Bm-LAAO) (LAO) (EC 1.4.3.2), Nematocyst-expressed protein 6 (NEP-6) (EC 3.4.24.-) (astacin-like metalloprotease toxin), Neoverrucotoxin subunit beta (NeoVTX subunit beta), Neprilysin-1 (EC 3.4.24.-), Phospholipase A(2) (EC 3.1.1.4) (phosphatidylcholine 2-acylhydrolase), Phospholipase A2 isozymes PA3A/PA3B/PA5 (PLA2) (EC 3.1.1.4) (phosphatidylcholine 2-acylhydrolase), Plancitoxin-1 (EC 3.1.22.1) (plancitoxin I) (Plan-I) (cleaved into plancitoxin-1 subunit alpha and plancitoxin-1 subunit beta), Reticulocalbin-2 (taipoxin-associated calcium-binding protein 49 homolog), Ryncolin-2, Ryncolin-4, Snaclec bothrojaracin subunit beta (BJC subunit beta), Translationally controlled tumor protein homolog (GTx-TCTP1), Venom acid phosphatase Acph-1 (EC 3.1.3.2) (allergen Api m 3), Venom allergen 5 (antigen 5) (Ag5) (cysteine-rich venom protein) (CRVP) (allergen Pol d 5), Venom dipeptidyl peptidase 4 (allergen C) (venom dipeptidyl peptidase IV) (EC 3.4.14.5) (allergen Api m 5), Venom prothrombin activator omicarin-C non-catalytic subunit (vPA) (venom coagulation factor Va-like protein) (cleaved into omicarin-C non-catalytic subunit heavy chain and omicarin-C non-catalytic subunit light chain), Venom prothrombin activator oscutarin-C non-catalytic subunit (vPA) (venom coagulation factor Va-like protein) (cleaved into oscutarin-C non-catalytic subunit heavy chain and oscutarin-C non-catalytic subunit light chain), Venom prothrombin activator pseutarin-C non-catalytic subunit (PCNS) (vPA) (venom coagulation factor Va-like protein) (cleaved into pseutarin-C non-catalytic subunit heavy chain and pseutarin-C non-catalytic subunit light chain), Venom prothrombin activator vestarin-D2 (vPA) (EC 3.4.21.6) (venom coagulation factor Xa-like protease) (cleaved into vestarin-D2 light chain and vestarin-D2 heavy chain), Zinc metalloproteinase/disintegrin (cleaved into snake venom metalloproteinase Mt-b (SVMP) (EC 3.4.24.-) and disintegrin), Zinc metalloproteinase-disintegrin-like HR1b (EC 3.4.24.-) (snake venom metalloproteinase) (SVMP) (trimerelysin I) (trimerelysin-1) (cleaved into disintegrin-like 1b), Zinc metalloproteinase-disintegrin-like NaMP (EC 3.4.24.-) (snake venom metalloproteinase) (SVMP)				
Stomolophus meleagris	Scyphozoa	A2-gamma, Alpha-latrocrustotoxin-Lt1a, Astacin-like metalloprotease toxin, Botulinum neurotoxin, Botulinum neurotoxin A5, Botulinum neurotoxin sub-type A5, Botulinum neurotoxin type A5, BPTI/Kunitz family of serine protease inhibitors, Calcium-independent phospholipase A2-gamma, Conodipine-M alpha chain, C-type lectin, C-type lectin or carbohydrate-recognition domain (CRD), Cytotoxin 2, Cytotoxin 5, Cytotoxin 6, Cytotoxin 7, Ectonucleotide pyrophosphatase/ phosphodiesterase family member 2, ExoU and VipD-like proteins, Group XV phospholipase A2, HlyIII, Kazal type serine protease inhibitors, Kazal type serine protease inhibitors and follistatin-like domains, Kunitz/Bovine pancreatic trypsin inhibitor domain, Kunitz/Bovine pancreatic trypsin inhibitor domain containing protein, Kunitz-type protease inhibitor, Lon protease, Neoverrucotoxin subunit beta, PAF_acetylesterase_like, Phospholipase A2, Phospholipase A2 isozyme PA4, Phospholipase A2 isozyme Vb-2, Phospholipase A2 isozymes, Phospholipase A2. group VI, Phospholipase B1, PLA2_bee_venom_like, Plancitoxin-1, Prokaryotic phospholipase A2, Putative zinc metalloproteinase YIL108W, Ryncolin-2, Ryncolin-3, Serpin, ShK, ShK domain-like, ShKT, Staphopain C, The kazal-type serine protease inhibitor domain, U9-ctenitoxin-Pr1a, Veficolin-1, Venom protease, Venom protein 302, Zinc metallo protein, Zinc metalloproteinase nas-13, Zinc metalloproteinase nas-15, Zinc metalloproteinase nas-39, Zinc metalloproteinase nas-4, Zinc metalloproteinase nas-6, Zinc metalloproteinase nas-7, Zinc-dependent metalloprotease	Milked venom	China	2014	[236]
Calvadosia cruxmelitensis	Staurozoa	Snaclec 7, Venom allergen, Plancitoxin-1, Glycoside hydrolase, Hyaluronidase, Lipase, Metalloprotease (Nematocyte expressed protein 6), Metalloprotease, Metalloprotease (Astacin-like toxin), Metalloprotease (SVMP), Zinc metalloproteinase-disintegrin-like berythractivase, Pore-forming toxin, C-type lectin, Venom protein 302, Phosphodiesterase, Basic phospholipase A2 textilotoxin A chain, Phospholipase-B, Serine protease, Serine-type	Tentacles	United Kingdom	2021	[218]

		endopeptidase (Blarina toxin), Enzyme inhibitor, Coagulation factor X, Turripeptide Gsg9.2, U-actitoxin-Avd3q, A.superbus venom factor 2				
Haliclystus antarcticus	Staurozoa	A. superbus venom factor 1, Alpha-latroinsectotoxin-Lt1a (Fragment), Basic phospholipase A2 nigroxin B, Beta-fibrinogenase stejnefibrase-2, Coagulation factor X-activating enzyme heavy chain, Cobra venom factor, Conotoxin Lt9a, Cysteine-rich venom protein natrin-1, DELTA-actitoxin-Ucs1a, Delta-latroinsectotoxin-Lt1a, Hyaluronidase, Inactive hyaluronidase B, Kunitz-type serine protease inhibitor U1-aranetoxin-Av1a, Metalloproteinase, Metalloproteinase 5, M-zodatoxin-Lt4b, Phospholipase A1, Phospholipase D LbSicTox-betaIA1a, Phospholipase D LiSicTox-alphaI-1 (Fragment), Phospholipase D LruSicTox-alphaIC1a (Fragment), Phospholipase D StSicTox-betaIC1 (Fragment), Phospholipase D StSicTox-betaIF1 (Fragment), Serine protease sp-Eoc49, Snaclec mamushigin subunit alpha, Snake venom metalloproteinase (Type III) 6, Snake venom serine protease KN5, Snake venom serine proteinase 14, SVMP-CohPH-2, Toxin CfTX-2, Turripeptide OL172, Venom allergen 5, Venom metalloproteinase 2, Venom plasminogen activator GPV-PA, Venom plasminogen activator TSV-PA, Zinc metalloproteinase homolog-disintegrin albolatin, Zinc metalloproteinase-disintegrin-like atrolysin-A	Nematocysts	Antarctica	2019	[212]
Zoanthus natalensis	Zoantharia	CL11004.Contig1 (Neprilysin-1), CL20605.Contig2 (Venom prothrombin activator), CL20909.Contig1 (Turripeptide), CL431.Contig1 (C-type lectin lectoxin), CL4489.Contig2 (Neprilysin-1), CL59.Contig2 (Venom prothrombin activator), CL6358.Contig1 (Venom prothrombin activator), CL6453.Contig1 (Venom prothrombin activator), CL6618.Contig3 (Venom phosphodiesterase), CL7419.Contig1 (Venom dipeptidyl peptidase), CL7419.Contig2 (Venom dipeptidyl peptidase), Unigene11574 (Venom prothrombin activator), Unigene12406 (Neprilysin-1), Unigene131487 (Venom phosphodiesterase), Unigene14196 (Perivitellin-2), Unigene143484 (Venom prothrombin activator), Unigene152864 (Kunitz toxin), Unigene160034 (U24-ctenitoxin (Protease inhibitor)), Unigene161096 (U24-ctenitoxin (Protease inhibitor)), Unigene1677 (Neprilysin-1), Unigene21022 (Neprilysin-1), Unigene25734 (Vascular endothelial growth factor), Unigene26067 (Vascular endothelial growth factor), Unigene34275 (Venom prothrombin activator), Unigene42296 (Alpha-latrocrustotoxin-Lt1a), Unigene510 (Venom prothrombin activator), Unigene59550 / ZoaKuz1 (Kunitz toxin), Unigene73653 (Venom prothrombin activator), Unigene77303 (Turripeptide), Unigene90060 (Venom prothrombin activator)	Polyps	n.a.	2019	[164]
Catostylus sp.	Scyphozoa	Toxin alpha-KTx1.13 and 12.5, Toxin BmKaTx10, Turripeptide VIII-01, Basic phospholipase A2 sistruxin B, Phospholipase A2, Phospholipase A1, Snake venom serine protease pictobin, Thrombin-like enzyme elegaxobin1, Venom factor, Toxin CrTX-A, Small cysteine-rich protein 1 2, Putative antimicrobial peptide 7848	Nematocysts	Sri Lanka	2024	[182]
Aurelia coerulea	Scyphozoa	Snaclec, Ctenitoxin, Veficolin, Neprilysin, Scoloptoxin, 5′-nucleotidase, Techylectin, Conodipine, Turripeptide, Reticulocalbin, Lactotransferrin, Venom factor, Thrombin, Phospholipase, Blarina toxin, Coagulation factor, Nematocyst expressed protein, Latroinsectotoxin, Latrotoxin, Serine protease inhibitor, Metalloprotease, Latrocrustotoxin, Ryncolin, Venom prothrombin activator, Calglandulin	Tentacles (medusa and polyp)	n.a.	2022	[237]
Stomolophus meleagris	Scyphozoa	Snaclec, Ctenitoxin, Veficolin, Neprilysin, Scoloptoxin, 5′-nucleotidase, Techylectin, Conodipine, Turripeptide, Reticulocalbin, Lactotransferrin, Venom factor, Thrombin, Phospholipase, Blarina toxin, Coagulation factor, Nematocyst expressed protein, Latroinsectotoxin, Latrotoxin, Serine protease inhibitor, Metalloprotease, Latrocrustotoxin, Ryncolin, Venom prothrombin activator, Calglandulin	Tentacles	China	2022	[237]
Macrorhynchia philippina	Hydrozoa	19 newly-sequenced peptides (LPLYKNLH, VPPFLFEPV, PMHPLFAYP, LGSMCSNAVLGP, NPGQPLYKGGLH, HSCDCM, DPVDLDDGPPL, DRPPLYKGGLH, EEEEEEPDGN, FDCYD, SSQFAFEEVEY, MNDLPKPA, HAPLVVEGLVAVMKN, MFAN, APPMVKYW, PVTLKKKC, CHAAPLCLTHLGVLVL, PAGEPTWF, PETGALCVGAVSHC)	Hydrocladiums, Hydrothecas	Brazil	2022	[238]
Liponema brevicorne	Actiniaria	70 peptides (1458–10,395 Da) and 50 peptides (1527 to 10,846 Da) in aqueous extracts and ethanol fractions, respectively	Tentacles	Russia	2021	[239]
Corallimorphus cf. pilatus	Corallimorpharia	83 peptides (1298–9812 Da) and 43 peptides (2984 to 9808 Da) in aqueous extracts and ethanol fractions, respectively	Tentacles	Russia	2021	[239]

Urticina aff. coriacea	Actiniaria	Fraction U1.3 - Peptides with molecular masses of 4179.9, 4391.6, 4536.5, 5651.6, and 6784.4 Da; Fraction U1.4 - peptides of 1748.3, 1988.4, 2206.5, 2505.5, 2737.0, and 4817.1 Da; Fraction U1.5 - peptides of 1988.0, 2232.1, 2421.4, 3474.9, and 5182.4 Da	Aqueous extract	Russia	2018	[240]
Rhizostoma pulmo	Scyphozoa	Rhizoprotease; Peptides from 905 to 3700 KDa	Tentacles	India	2017	[190]
Anthopleura midori	Actiniaria	A1A-c14965_c0_g1	Tentacles	China	2025	[194]
Actinia equina	Actiniaria	B3a-c29555_c4_g4	Tentacles	China	2025	[194]

Table S5. Cnidaria species targeted in venom transcriptomic studies. Key toxins are highlighted in green.

Species	Order	Toxins	Tissues	Location	Year	Ref
Ceriantheomorphe brasiliensis	Ceriantharia	Cysteine-rich venom protein, ShK-domain, Three-finger toxin, Turripeptide, Blarina Toxin, C-type lectin lectoxin, Coagualtion factor X, Coagulation factor V, Coagulation factor X-activating enzyme heavy chain, Galactose-specific lectin, Ryncolin, Snaclec, Snake venom 5′nucleotidase, Thrombin-like enzyme, Thyrostimulin, Veficolin-1, Venom peptide isomerase heavy chain, Venom prothrombin activator (F5/F8 type C), Venom prothrombin activator (Trypsin), Zinc metalloproteinase-disintegrin, Jellyfish Toxin, Stonutoxin/Neoverrucotoxin, Acetylcholinesterase, L-amino-acid oxidase, Phospholipase-A2/Conodpine, Phospholipase-B, Phospholipase-D, Putative endothelial lipase, Putative lysosomal acid lipase/cholesteryl ester hydrolase, Venom phosphodiesterase, Kunitz-type serine protease inhibitor, U24-ctenitoxin-Pn1a, CRISP/Allergen/PR-1, Venom allergen, Venom phosphatase, Venom protease, Venom serine protease, Techylectin-like, Astacin-like metalloprotease toxin, Glutaminyl-peptide cyclotransferase, Hyaluronidase, Nematocyst expressed protein, Neprilysin, Reticulocalbin, Venom protein 302	Tentacles	Brazil	2020	[241]
Pachycerianthus cf. maua	Ceriantharia	Cysteine-rich venom protein, ShK-domain, Three-finger toxin, Turripeptide, U-actitoxin-Avd9a, Beta-fibrinogenase mucrofibrase-3, Blarina Toxin, C-type lectin lectoxin, Coagulation factor V, Galactose-specific lectin, Ryncolin, Snaclec, Snake venom 5′nucleotidase, Snake venom serine proteinase, Veficolin-1, Venom prothrombin activator (F5/F8 type C), Venom prothrombin activator (Trypsin), Zinc metalloproteinase-disintegrin, DELTA-thalatoxin-Avl2a, Jellyfish Toxin, Stonutoxin/Neoverrucotoxin, Waprin, Acetylcholinesterase, L-amino-acid oxidase, Peroxiredoxin, Phospholipase-A2/Conodpine, Putative endothelial lipase, Putative lysosomal acid lipase/cholesteryl ester hydrolase, Kunitz-type serine protease inhibitor, U-actitoxin-Avd3m, U24-ctenitoxin-Pn1a, Venom allergen, Venom protease, Venom serine protease, Techylectin-like, Astacin-like metalloprotease toxin, Cystatin, Glutaminyl-peptide cyclotransferase, Nematocyst expressed protein, Reticulocalbin, Venom protein 302	Tentacles	n.a.	2020	[241]
Isarachnanthus nocturnus	Ceriantharia	Cysteine-rich venom protein, ShK-domain, Three-finger toxin, C-type lectin lectoxin, Coagualtion factor X, Coagulation factor V, Ryncolin, Snaclec, Snake venom VEGF, Veficolin-1, Venom prothrombin activator (F5/F8 type C), Venom prothrombin activator (Trypsin), Zinc metalloproteinase-disintegrin, Acetylcholinesterase, Gilatoxin, L-amino-acid oxidase, Phospholipase-A2/Conodpine, Phospholipase-B, Putative endothelial lipase, Putative lysosomal acid lipase/cholesteryl ester hydrolase, Kunitz-type serine protease inhibitor, Venom allergen, Venom phosphatase, Venom serine protease, Astacin-like metalloprotease toxin, Glutaminyl-peptide cyclotransferase, Nematocyst expressed protein, Neprilysin, Reticulocalbin, Venom protein 302	Tentacles	Brazil	2020	[241]
Pachycerianthus borealis	Ceriantharia	332-1 propeptide toxin, Cysteine-rich venom protein, ShK-domain, Turripeptide, U33-theraphotoxin-Cg1b, Blarina Toxin, C-type lectin lectoxin, Coagualtion factor X, Coagulation factor V, Coagulation factor X-activating enzyme heavy chain, Galactose-specific lectin, Ryncolin, Snaclec, Snake venom 5′nucleotidase, Snake venom VEGF, Thrombin-like enzyme, Veficolin-1, Venom peptide isomerase heavy chain, Venom prothrombin activator (F5/F8 type C), Venom prothrombin activator (Trypsin), Zinc	Tentacles	n.a.	2020	[241]

		metalloproteinase-disintegrin, DELTA-thalatoxin-Avl2a, Jellyfish Toxin, Millepora cytotoxin, Stonutoxin/Neoverrucotoxin, Waprin, Acetylcholinesterase, L-amino-acid oxidase, Peroxiredoxin, Phospholipase-A2/Conodpine, Phospholipase-B, Phospholipase-D, Putative endothelial lipase, Putative lysosomal acid lipase/cholesteryl ester hydrolase, Trehalase, Venom phosphodiesterase, Kunitz-type serine protease inhibitor, U24-ctenitoxin-Pn1a, CRISP/Allergen/PR-1, Venom allergen, Venom phosphatase, Venom protease, Venom serine carboxypeptidase, Venom serine protease, Astacin-like metalloprotease toxin, Glutaminyl-peptide cyclotransferase, Nematocyst expressed protein, Neprilysin, Reticulocalbin, Venom protein 302				
Arachnanthus errans	Ceriantharia	AB hydrolase superfamily, Acetylcholinesterase family, Arthropod phospholipase D family, AVIT (prokinetin) family, Conopeptide P-like superfamily, CREC family, CRISP, Cystatin family, Ficolin lectin family, Flavin monoamine oxidase family, Glutaminyl-peptide cyclotransferase family, Glycoside hydrolase family, Glycopeptide/Perforin (magpf) family, Membrane attack complex/Phosphodiesterase family, Multicopper oxidase family, Nucleotide pyrophosphatase/Phosphohodiesterase family, Peptidase family, Peptidase M12A, Peptidase M13 family, Peptidase S1 family, Phospholipase A2 family, Phospholipase B-like family, Scoloptoxin-01 family, Snaclec family, Snake waprin family, SNTX/VTX toxin family, Techylectin-like family, Transferrin, True venom lectin family, Venom C3 homolog family, Venom Kunitz-type family, Venom metalloproteinase (M12B) family, Venom protein 302	Larva, Polyp	Brazil	2025	[242]
Palythoa variabilis	Zoantharia	ShK/Aurelin, Anthozoan neurotoxin-like, Three finger toxin, Turripeptide, Snake venom VEGF, C-type lectin (Snaclec), Kunitz-type, Thyroglobulin type-1, Actinoporin, Helofensin, MACPF, Mixed function enzymes, Phospholipase A2, Venom protein 302	Whole Body	Brazil	2016	[192]
Thelohanellus kitauei	Myxozoa	K-II/Kunitz, CRiSP, Peptidase-S1, True-venom-lectin, M12B, Snaclec	Whole Body	China	2022	[243]
Myxobolus xiantaoensis	Myxozoa	K-II/Kunitz, CRiSP, Peptidase-S1, Phospholipase B-like, True-venom-lectin, M12B, Snaclec, TCTP, AB-hydrolase/Lipase, PLA2, Glycosyl hydrolase 56, PDGF/VEGF, Venom-complement-C3	Whole Body	China	2022	[243]
Myxobolus ampullicapsulatus	Myxozoa	K-II/Kunitz, CRiSP, Phospholipase B-like, M12B, Snaclec, TCTP, AB-hydrolase/Lipase,	Whole Body	China	2022	[243]
Myxobolus turpisrotundus	Myxozoa	K-II/Kunitz, CRiSP, True-venom-lectin, M12B, Snaclec, TCTP, AB-hydrolase/Lipase, PLA2, META/ShK, Natriuretic-peptide	Whole Body	China	2022	[243]
Myxobolus honghuensis	Myxozoa	K-II/Kunitz, CRiSP, Peptidase S1, True-venom-lectin, M12B, Snaclec, TCTP, AB-hydrolase/Lipase, PDGF/VEGF	Whole Body	China	2022	[243]
Goniopora columna	Scleractinia	Hemostatic and hemorrhagic toxins (Phosphodiest, EGF, Lectin_C, Fibrinogen_C, F5_F8_type_C), Mixed function enzymes (Thyroglobulin_1, Trypsin, Acetylcholinesterase, Metalloprotease, Phospholip_B, Phospholip_A2), protease inhibitors (Kunitz – including GcKuz1, Kazal), Neurotoxins (CAP, Ank) and cytotoxins (MACPF)	Polyp	Australia	2025	[160]
Radianthus crispa	Actiniaria	AB hydrolase, acrorhagin, BBH, β-Defensins, CAP, C-type lectin, Collagen, CUB domain, EGF domain peptide family, Factor 5/8 C-domain, G-protein coupled receptor, Immunoglobulin, ICK, Kunitz-type, kazal-like type, Other proteins, Peptidase S1, ShKT domain, supwaprin-a-like, Type-B carboxylesterase, Uncharacterized proteins, Unknow (including HC-18 and HC-19)	Tentacles, Column, Mesenterial filaments	China	2024	[28]
Anthopleura elegantissima	Actiniaria	Phospholipase A2, Cytolysins Type II and IV, KTx Type I-V, NaTx Type I-III. Acrorhagins Type I and II	Acrorhagi (aggressive and non-aggressive polyps)	United States of America	2015	[244]
Alatina alata	Cubozoa	450 transcripts related to "venom implicated genes", including CRISPs, zinc metalloproteinase/astacin, venom protein 302, venom prothrombin activator pseutarin-c non-catalytic subunit, alpha-2-macroglobulin family Nterminal region, CaTX/CrTX, serine protease coagulation factor vii, chymotrypsin-like elastase family, serine protease inhibitor, zinc metalloproteinase nas-4/venom factor,	Gastric cirri, Ovaries, Tentacle (with pedalium base), Rhopalium (Adult female) and Larval planulae	Netherlands	2016	[245]
Anemonia sulcata	Actiniaria	Acrorhagins, AETX-like, Metalloproteases, Class 9a (KTx), Type II Cytolysins, MACPF, NaTx, EGF-like, Type I KTxs, PI/Type II KTxs, Type III KTxs, Type V KTxs, PLA2, Neurotoxin-1, Calglandulin, Venom peptide isomerase heavy chain, Venom prothrombin activator vestarin-D2, Toxin Bcg III 23.41, Analgesic polypeptide HC3, Turripeptide Lol9.1, Translationally	Tentacles, Mesenterial Filaments, Column	Ireland	2016	[246]

		controlled tumor protein, Nematocyte expressed protein 6, Antihypertensive protein BDS-1, Kunitz-type proteinase inhibitor 5 II, SE-cephalotoxin				
Radianthus crispa	Actiniaria	ASIC toxins, Acrorhagins, Metalloproteases, Type II Cytolysins, MACPF, NaTx, EGF-like, Type I KTxs, PI/Type II KTxs, Type III KTxs, Type V KTxs, PLA2, Calglandulin, Venom allergen 5, Venom prothrombin activator vestarin-D2, Augerpeptide hhe53, Translationally controlled tumor protein, Peptide toxin Am-2, Peptide toxin Am-3, SE-cephalotoxin, Vespryn, Basic phospholipase A2 acanthin-1, Snake venom 5′-nucleotidase, Kunitz-type serine protease inhibitor, Turripeptide lci9.1, Cytolysin Src-1, Venom protease, Toxin pi-PMTX-Pcr1a, Nematocyte expressed protein 6, Peroxiredoxin-4, Venom prothrombin activator porpharin-D	Tentacles, Mesenterial Filaments, Column	n.a.	2016	[246]
Megalactis griffithsi	Actiniaria	Acrorhagins, Metalloproteases, Type II Cytolysins, NaTx, Type I KTxs, PI/Type II KTxs, Type III KTxs, PLA2, Augerpeptide hhe53, Translationally controlled tumor protein, SE-cephalotoxin, Neprilysin-1, L-amino-acid oxidase, Snake venom 5′-nucleotidase, Neurotoxin Ae I, Calglandulin, Reticulocalbin-2, Peptide toxin Am-2, Kunitz-type serine protease inhibitor, Phospholipase A2, Kunitz-type serine protease inhibitor bitisilin-2	Tentacles, Mesenterial Filaments, Column	n.a.	2016	[246]
Gonionemus vertens	Hydrozoa	Neprilysin-1_1, Zinc_metalloproteinase-disintegrin-like_VAP2B_272, Planctotixin-1_1, Glutaminyl-peptide_cyclotransferase_1, Putative_protein-glutamate_O-methyltransferase_1, Alpha-latroinsectotoxin-Lt1a_2, Alpha-latroinsectotoxin-Lt1a_1, Snake_venom_metalloproteinase_acutolysin-C_1, Delta-latroinsectotoxin-Lt1a_1, Phospholipase_A1_19, Astacin-like_metalloprotease_toxin_1_1, Disintegrin-like_leberagin-C_8, Venom_prothrombin_activator_pseutarin-C_catalytic_subunit_12, Techylectin-like_protein_1, Zinc_metalloproteinase-disintegrin-like_alternagin_2, Astacin-like_metalloprotease_toxin_3_2, Zinc_metalloproteinase_recombinant_fibrinogenase_II_1, Astacin-like_metalloprotease_toxin_5_1, Snake_venom_metalloproteinase_fibrolase_19, Venom_peptide_isomerase_heavy_chain_2, Acetylcholinesterase-1_1, Kunitz-type_serine_protease_inhibitor_bitisilin-3_1, Hyaluronidase_1, Snake_venom_metalloproteinase_Ac1_1, Factor_V_activator_RVV-V_gamma_142, Thrombin-like_enzyme_ancrod_11, Astacin-like_metalloprotease_toxin_4_1, Galactose-specific_lectin_natterin_1, Kunitz-type_serine_protease_inhibitor_U1-aranetoxin-Av1a_1, Disintegrin_bitistatin_6, Basic_phospholipase_A2_notexin_18, Phospholipase_A2_large_subunit_1, Acidic_phospholipase_A2_1_322, Venom_allergen_5, Kunitz-type_serine_protease_inhibitor_2_4, C-type_lectin_mannose-binding_isoform_30, Protease_inhibitor_1_6, Kunitz-type_serine_protease_inhibitor_Hg1_1, Peroxiredoxin-4_1, Kunitz-type_serine_protease_inhibitor_BmKTT-2_1, Disintegrin_saxatilin_24, Kunitz-type_serine_protease_inhibitor_conotoxin_Cal9, Hyaluronidase_conohyal-ad1_1, Zinc_metalloproteinase-disintegrin-like_mikarin_1, Kunitz-type_serine_protease_inhibitor_PIVL_13, Kappa-PHI-teraphotoxin-H5-Ia_80, Kunitz-type_serine_protease_inhibitor_BmKTT-3_1, Venom_allergen_5_1, Phospholipase_A2_imperatoxin-I_4, Kunitz-type_serine_protease_inhibitor_As-fr-19_1, Thrombin-like_enzyme_TLBm_1, Basic_phospholipase_A2_2_60, Venom_allergen_3_30, Zinc_metalloproteinase-disintegrin-like_ammodytagin_1, Basic_phospholipase_A2_acanthin-1_3, Snaclec_echicetin_subunit_beta_6, Snaclec_echicetin_subunit_alpha_1, Condopine-M_alpha_chain_1, Hyaluronidase_conohyal-Cn1_1, Sphingomyelin_phosphodiesterase_D_1, Snaclec_coagulation_factor_X-activating_enzyme_light_chain_2_135, Snaclec_coagulation_factor_IX(a)factor_X-binding_protein_subunit_A_1Gliodyus_halys, Snaclec_rhodocetin_subunit_delta_1, Kunitz-type_serine_protease_inhibitor_homolog_dendrotoxin_K_154, Kunitz-type_serine_protease_inhibitor_homolog_beta-bungarotoxin_B5-B_chain_1, Disintegrin_acostatin-alpha_26, Kunitz-type_serine_protease_inhibitor_homolog_delta-dendrotoxin_2, Phospholipase_A2_notodacylipin_6, Turripeptide_lci9, Turripeptide_Pal9, Kunitz-type_serine_protease_inhibitor_homolog_alpha-dendrotoxin_3, Snaclec_bothrocretin_subunit_beta_1, L-amino-acid_oxidase_31, Cysteine-rich_venom_protein_Mr30_2, Toxin_CrTX-A_1, Snaclec_rhodocetin_subunit_alpha_2, Phospholipase_A2_Scol@Pla_1, Disintegrin_EMF10B_17, Neutral_phospholipase_A2_3_21, Disintegrin_lebein-1-beta_8, Kunitz-type_proteinase_inhibitor_AEPI-IV_2, C-type_lectin_galatrox_1, SE-cephalotoxin_1, U24-ctenitoxin-Pn1a_1, Kunitz-type_conkunitzin-S1_2, Cystatin-1_1, Kunitz-type_serine_protease_inhibitor_bicolin_1, Thrombin-like_enzyme_catroxobin-1_1, C-type_lectin_6, Serine_proteinase-like_BMK-CBP_1, Snake_venom_metalloprotease_inhibitor_02A10_1, Venom_protein_164_1, Kunitz-type_serine_protease_inhibitor_Kunitz-1_1, Nematocyte_expressed_protein_6_1, Phospholipase_A2_1,	Whole Body	United States of America	2020	[247]

		Phospholipase_A2_homolog_crotoxin_acid_subunit_CA_21, Snaclec_lebein_subunit_alpha_9, Venom_allergen_5_15, Zinc_metalloproteinase@disintegrin_107, Zinc_metalloproteinase@disintegrin_4, Zinc_metalloproteinase@disintegrin_5				
Cryptodendrum adhaesivum	Actiniaria	Astacin-like metalloprotease toxin M12A, Nematocyst expressed protein 6, Venom protein 302, Actinoporin family, MAC/PF, Cysteine-rich venom protein, Sea anemone structural class 9a, Sea anemone type 1 potassium channel toxin subfamily, Sea anemone type 3 potassium channel toxin subfamily (BDS), sodium channel toxin Type II, Conopeptide P-like superfamily, Venom Kunitz-type family, A. superbus venom factor 1, snake venom serine protease Dav-PA, Venom serine carboxypeptidase, Venom allergen 5, Venom serine protease, Venom serine protease Bi-VSP, Blarina toxin, Coagulation factor X-activating enzyme, Ryncolin, Snaclec family, True venom lectin family, Veficolin-1, Venom factor (VF), Venom prothrombin activator, Zinc metalloproteinase/disintegrin-like (M12B), Galactose-specific lectin nattectin, Venom protease, Venom peptide isomerase heavy chain, EGF domain peptide family, Sea anemone 8 toxin family	Nematocysts, Column	Australia	2022	[248]
Stichodactyla haddoni	Actiniaria	Astacin-like metalloprotease toxin M12A, Nematocyst expressed protein 6, Cystatin-2, Venom protein 59.1, MAC/PF, Sea anemone short toxin (type III) family (Delta-actitoxin-Avd2b1), Sea anemone structural class 9a, Sea anemone type 1 potassium channel toxin subfamily, Sea anemone type 3 potassium channel toxin subfamily (BDS), sodium channel toxin Type I, sodium channel toxin Type II, Conopeptide P-like superfamily, Omega-theraphotoxin-Pm1a, Neurotoxin 10, Psalmotoxin-1 (PcTx1), Scoloptoxin, Phospholipase A2, Venom Kunitz-type family, Venom serine carboxypeptidase, Venom allergen 5, Venom serine protease, Venom serine protease Bi-VSP, Blarina toxin, Coagulation factor X-activating enzyme, Cobra venom factor, Ryncolin, Snaclec family, True venom lectin family, Veficolin-1, Venom prothrombin activator, Zinc metalloproteinase/disintegrin-like (M12B), Galactose-specific lectin nattectin, Venom protease, Venom peptide isomerase heavy chain, EGF domain peptide family,	Tentacles	Australia	2022	[248]
Heterodactyla hemprichii	Actiniaria	Astacin-like metalloprotease toxin M12A, Nematocyst expressed protein 6, Actinoporin family, MAC/PF, Cnidaria small cysteine-rich protein (SCRiP) family, Neurotoxin ShK-like1, Sea anemone short toxin (type III) family (Delta-actitoxin-Avd2b1), Sea anemone type 3 potassium channel toxin subfamily (BDS), sodium channel toxin Type II, Conopeptide P-like superfamily, Venom Kunitz-type family, A. superbus venom factor 1, Venom allergen 5, Venom serine protease, Venom serine protease Bi-VSP, Blarina toxin, Coagulation factor V, Cobra venom factor, Ryncolin, Snaclec family, Veficolin-1, Venom factor (VF), Venom prothrombin activator, Zinc metalloproteinase/disintegrin-like (M12B), Venom protease, Venom peptide isomerase heavy chain, EGF domain peptide family,	Nematocysts, Exocoelic tentacles, Endocoelic tentacles	Australia	2022	[248]
Heteractis doreensis	Actiniaria	Astacin-like metalloprotease toxin M12A, Nematocyst expressed protein 6, Cystatin-2, Actinoporin family, MAC/PF, Cysteine-rich venom protein, Sea anemone type 1 potassium channel toxin subfamily, Sea anemone type 3 potassium channel toxin subfamily (BDS), sodium channel toxin Type I, Conopeptide P-like superfamily, Venom Kunitz-type family, Snake venom serine protease salmobin, Venom serine carboxypeptidase, Venom allergen 5, Venom serine protease, Venom serine protease Bi-VSP, Ryncolin, Snaclec family, True venom lectin family, Veficolin-1, Venom prothrombin activator, Zinc metalloproteinase/disintegrin-like (M12B), Galactose-specific lectin nattectin, Venom protease, Venom peptide isomerase heavy chain, EGF domain peptide family, Sea anemone 8 toxin family	Tentacle, Column, Mesenterial Filaments,	n.a.	2022	[248]
Radianthus crispa	Actiniaria	Astacin-like metalloprotease toxin M12A, Nematocyst expressed protein 6, Cystatin-2, Actinoporin family, MAC/PF, Cysteine-rich venom protein, Sea anemone type 1 potassium channel toxin subfamily, Sea anemone type 3 potassium channel toxin subfamily (BDS), sodium channel toxin Type I, Conopeptide P-like superfamily, Scoloptoxin, Phospholipase A2, Venom Kunitz-type family, A. superbus venom factor 1, Snake venom serine protease salmobin, Venom serine carboxypeptidase, Venom allergen 5, Venom serine protease, Venom serine protease Bi-VSP, Blarina toxin, Coagulation factor V, Coagulation factor X-activating enzyme, Ryncolin, Snaclec family, True venom lectin family, Veficolin-1, Venom phosphodiesterase, Venom prothrombin activator, Zinc metalloproteinase/disintegrin-like (M12B), Galactose-specific lectin nattectin, Natterin-4, Venom protease, Venom peptide isomerase heavy chain, EGF domain peptide family,	Inner and Outer Tentacles	Australia	2022	[248]
Entacmaea quadricolor	Actiniaria	Astacin-like metalloprotease toxin M12A, Nematocyst expressed protein 6, Cystatin-2, Actinoporin family, MAC/PF, Cnidaria small cysteine-rich protein (SCRiP) family, Cysteine-rich venom protein, Sea anemone structural class 9a, Sea anemone type 1 potassium channel toxin subfamily, Sea anemone type 3 potassium channel toxin subfamily (BDS), Conopeptide P-like superfamily, Phospholipase A2, Venom Kunitz-type family, Venom serine carboxypeptidase, Techylectin-like protein, Venom	n.a.	n.a.	2022	[248]

		allergen 5, Venom serine protease Bi-VSP, Blarina toxin, Coagulation factor V, Coagulation factor X-activating enzyme, Cobra venom factor, Ryncolin, Snaclec family, True venom lectin family, Veficolin-1, Venom factor (VF), Venom prothrombin activator, Zinc metalloproteinase/disintegrin-like (M12B), Galactose-specific lectin nattectin, Natterin-4, Venom protease, Venom peptide isomerase heavy chain, EGF domain peptide family, Sea anemone 8 toxin family				
Porites lutea	Scleractinia	Cysteine-rich venom protein, Kunitz-type serine protease inhibitor (including PlKuz1), U-actitoxin-Avd3h, Turripeptide, Scoloptoxin, Ctenitoxin, Ophiophagus venom factor, Venom allergen, Venom carboxylesterase, DELTA-actitoxin, Pervitellin-2, Stonustoxin, Veswaprin, Con-Ins, Venom prothrombin activator, Snaclec, Venom prothrombin activator trocarin-D, Snake venom metalloproteinase hemorrhagic factor, Zinc metalloproteinase	Polyps	Kenya	2025	[159]
Palythoa caribaeorum	Zoantharia	Kunitz/Bovine pancreatic trypsin inhibitor domain (including PcKuz1, PcKuz2, PcKuz3), Cysteine-rich secretory protein family, ShK domain-like, Kazal-type serine protease inhibitor domain, Disintegrin, Coagulation Factor Xa inhibitory site, WAP-type (Whey Acidic Protein) 'four-disulfide core', Cysteine-rich secretory protein family, Fibrinogen beta and gamma chains, C-terminal globular domain, Phospholipase A2, Phospholipase A3	Whole Body	Brazil	2018	[105]
Galaxea fascicularis	Scleractinia	Phospholipase A2, Phospholipase A2 Cdr-13, Phospholipase B, Phospholipase D, basic phospholipase A2, cytosolic phospholipase A2, 85/88 kDa calcium-independent phospholipase A2, DELTA-alicitoxin-Pse2b, DELTA-actitoxin-Aeq1b, Toxin CrTX-A, Kunitz-type kappaPI-theraphotoxin, Zinc metalloproteinase-disintegrin-like halysase, Astacin-like metalloprotease toxin	Hunting tentacles, Sweeper tentacles	Israel	2020	[249]
Bunodosoma goanense	Actiniaria	Actinoporin family, Sea anemone subfamily, Cnidaria small cysteine-rich protein (SCRiP) family, EGF domain peptide family, Jellyfish toxin family, Type II subfamily, NEP3 family, Phospholipase A2 family, Sea anemone 8 toxin family, Sea anemone short toxin (type III) family, Sea anemone sodium channel inhibitory toxin family, Sea anemone structural class 9a family, Sea anemone type 1 potassium channel toxin family, Type 1a subfamily, Sea anemone type 1 potassium channel toxin family, Type 1b subfamily, Sea anemone type 3 (BDS) potassium channel toxin family, Sea anemone type 5 potassium channel toxin family, Sea anemone type 6 potassium channel toxin family, Venom Kunitz-type family, Sea anemone type 2 potassium channel toxin subfamily	Whole Body	India	2026	[183]
Chrysaora quinquecirrha	Scyphozoa	Chrysaoralin	Nematocysts	United States	2017	[193]
Stylophora pistillata	Scleractinia	SCRiP, PLA2, Actinoporin, MACPF, Kunitz-like, Hemagglutinin/amebocyte aggregation factor	Coral fragment	Israel	2021	[250]

Table S6. Expression patterns and functional roles of characterized toxins in *Nematostella vectensis*. Expression data are based on protein and/or mRNA levels across life stages.

Toxin	Expression Pattern	Main Cell	Tissue Location	Function	Key Notes	UniProt	Reference
NEP3	Low in unfertilized eggs; high in planula (peak), primary polyp and adult	Nematocysts (sub-populations in the same tissue)	Late planula – Ectoderm, with high concentration of expressing cells in the oral pole	Defense from fish predators	Zebrafish (*D. rerio*) larvae exhibited neurotoxic signals, followed by death (5h)	A7RJ12	[42]

			Primary polyp - Body wall and physa ectoderm; upper and lower pharynx		Killifish (*F. heteroclitus*) avoid consumption of *N. vectensis* planulae.		
NEP3-like	Planula and primary polyp	Nematocysts		Unknown	n.a.	A7RJ13	
NEP4	Planula and primary polyp	Nematocysts		Unknown	n.a.	A7RNZ0	
NEP6	Planula and primary polyp	Nematocysts and gland cells	Early planula – Ectoderm (developing nematocysts)<br>Primary polyp – Pharynx (gland cells), no expression in tentacle tips	Possible defense in early stages and prey killing/digestion in later stages	Metalloprotease with Astacin Domain	K7Z9Q9	[41]
NEP8	Low in planula and primary polyp	Nematocysts	Late planula – Lower pharynx (handful of nematocysts)<br>Primary polyp - Absent from tentacles and outer body wall	Possible prey killing/digestion	n.a.	A7SXG9	[42]
NEP16	Planula and primary polyp	Nematocysts	Planula – Body wall ectoderm<br>Primary polyp – Tentacle ectoderm	Unknown	CAP domain (involved in toxicity)<br>Co-expressed with the nematocyst-specific minicollagen NvNCol-3	AFY07205.1	[41]
Nv1	Low in unfertilized eggs and planula (intron retention); high in primary polyp (proper alternative splicing); very high in adults	Ectodermal gland cells	Adult (female) - Mesenteries (gametes-producing tissue), tentacles, pharynx and physa	Predation of arthropod prey (paralysis/death)	Encoded by 8 different genes<br>Purely adult toxin due to intron retention<br>Active against arthropod sodium channels<br>Grass shrimps (*Palaemonetes* sp), natural predators of *N. vectensis*, were repelled by the tentacles<br>Induces impaired swimming, paralysis, and death of the crustacean *A. nauplii* within several hours	B1NWR6, P0CH90, B1NWS4, B1NWS1, B1NWS8, A7SCE5, P0CH46, B1NWR7	[42,83,146,251,252]

Nv4	All life stages, with high expression in adult females	Ectodermal gland cells (different types)	Adult (female) - Highly transcribed in mesenteries (gametes-producing tissue) and slightly transcribed in tentacles, pharynx and physa	Defense from fish predators and prey paralysis/death	Active against fish via an unknown receptor<br>Contractile paralysis and twitching of the tail within 30 minutes, followed by death within 40 mins in zebra fish larvae (*D. rerio*)	A0A5J6K6K4	[42,146]
Nv5	High in unfertilized eggs and female adults; low in planula and primary polyp; small increase in male adults	Ectodermal gland cells			Active against fish via an unknown receptor<br>Contractile paralysis and twitching of the tail within 20 minutes, followed by death within 30 mins in zebra fish larvae (*D. rerio*)	A0A5J6KCM0	[42,146]
Nv7	All life stages. In adults, only on females	Ectodermal gland cells (different types)	Unknown	Unknown	Expression pattern like NvePtx1 and co-expressed with other toxins in the planula gland cells, indicating that it might be a venom component	P0DQR3	[146]
NvLysin1b	Planula and primary polyp	Large gland cells	Pharynx	Unknown	Aerolysin-homologue (pore forming toxin). May have a similar role to hydralysins.	n.a.	[158]
NvePTx1	Highly expressed in unfertilized eggs, planula, primary polyp and female adults; absent in male adults (only low mRNA expression)	Different types of ectodermal gland cells (one large and elongated and another small and round)	Unfertilized eggs and early post-fertilization stages – Uniform expression<br>Gastrulae - Absent from the oral and aboral poles<br>Planula - ectoderm in packed gland cells absent from the two body poles, and upon metamorphosis, the expression diminishes<br>Adult females – Highly expressed in mesenteries (gametes-producing tissue)	Probable defense from fish predators (presence in egg and ectodermal gland cells of the planula)	Sea anemone type 5 potassium channel toxin family<br>Killifish (*F. heteroclitus*) avoid consumption of *N. vectensis* eggs and planulae loaded with toxins, possibly mainly by the action of NvePTx1 and members of the NEP3 family<br>Induces a rapid increase in swimming speed on zebrafish (*D. rerio*) larvae, as well as death 2h-18h later	A7RMN1	[42,134,146]

Neurotoxin Shk-like1	Low in adults (male and female); High in planula and primary polyp	Nematocysts	Primary polyp – Body column nematocysts and absent from tentacles	Probable defense from fish predators in early stages	Induces contraction paralysis followed by death (2h) on zebrafish larvae and body contractions on *N. vectensis* polyps.	P0DQR6	[146]

References

1. Honma, T.; Minagawa, S.; Nagai, H.; Ishida, M.; Nagashima, Y.; Shiomi, K. Novel peptide toxins from acrorhagi, aggressive organs of the sea anemone Actinia equina. *Toxicon* **2005**, *46*, 768-774, doi:10.1016/j.toxicon.2005.08.003.
2. Uechi, G.; Toma, H.; Arakawa, T.; Sato, Y. Molecular characterization on the genome structure of hemolysin toxin isoforms isolated from sea anemone Actineria villosa and Phyllodiscus semoni. *Toxicon* **2010**, *56*, 1470-1476, doi:10.1016/j.toxicon.2010.09.002.
3. Uechi, G.; Toma, H.; Arakawa, T.; Sato, Y. Biochemical and physiological analyses of a hemolytic toxin isolated from a sea anemone Actineria villosa. *Toxicon* **2005**, *45*, 761-766, doi:10.1016/j.toxicon.2005.01.015.
4. Macek, P.; Lebez, D. Isolation and characterization of three lethal and hemolytic toxins from the sea anemone Actinia equina L. *Toxicon* **1988**, *26*, 441-451, doi:10.1016/0041-0101(88)90183-3.
5. Pungercar, J.; Anderluh, G.; Macek, P.; Franc, G.; Strukelj, B. Sequence analysis of the cDNA encoding the precursor of equinatoxin V, a newly discovered hemolysin from the sea anemone Actinia equina. *Biochim Biophys Acta* **1997**, *1341*, 105-107, doi:10.1016/s0167-4838(97)00083-6.
6. Anderluh, G.; Krizaj, I.; Strukelj, B.; Gubensek, F.; Macek, P.; Pungercar, J. Equinatoxins, pore-forming proteins from the sea anemone Actinia equina, belong to a multigene family. *Toxicon* **1999**, *37*, 1391-1401, doi:10.1016/s0041-0101(99)00082-3.
7. Morante, K.; Bellomio, A.; Viguera, A.R.; González-Mañas, J.M.; Tsumoto, K.; Caaveiro, J.M. The isolation of new pore-forming toxins from the sea anemone Actinia fragacea provides insights into the mechanisms of actinoporin evolution. *Toxins* **2019**, *11*, 401, doi:10.3390/toxins11070401.
8. Norton, R.S.; Bobek, G.; Ivanov, J.O.; Thomson, M.; Fiala-Beer, E.; Moritz, R.L.; Simpson, R.J. Purification and characterisation of proteins with cardiac stimulatory and haemolytic activity from the anemone Actinia tenebrosa. *Toxicon* **1990**, *28*, 29-41, doi:10.1016/0041-0101(90)90004-q.
9. Kohno, Y.; Satoh, H.; Iguchi, A.; Nagai, H. Characterization of a new hemolytic protein toxin from the sea anemone Anthopleura asiatica. *Fisheries Science* **2009**, *75*, 1049-1054, doi:10.1007/s12562-009-0112-2.

10. Alvarado-Mesen, J.; Solano-Campos, F.; Canet, L.; Pedrera, L.; Hervis, Y.P.; Soto, C.; Borbon, H.; Lanio, M.E.; Lomonte, B.; Valle, A.; et al. Cloning, purification and characterization of nigrelysin, a novel actinoporin from the sea anemone Anthopleura nigrescens. *Biochimie* **2019**, *156*, 206-223, doi:10.1016/j.biochi.2018.07.013.
11. Tsutsui, K.; Sato, T. Identification of the two new, functional actinoporins, CJTOX I and CJTOX II, from the deep-sea anemone Cribrinopsis japonica. *Toxicon* **2018**, *148*, 40-49, doi:10.1016/j.toxicon.2018.04.008.
12. Samejima, Y.; Yanagisawa, M.; Aoki-Tomomatsu, Y.; Iwasaki, E.; Ando, J.; Mebs, D. Amino acid sequence studies on cytolytic toxins from sea anemone Heteractis magnifica, Entacmaea quadricolor and Stichodactyla mertensii (Anthozoa). *Toxicon* **2000**, *38*, 259-264, doi:10.1016/s0041-0101(99)00154-3.
13. Klyshko, E.V.; Issaeva, M.P.; Monastyrnaya, M.M.; Il'yna, A.P.; Guzev, K.V.; Vakorina, T.I.; Dmitrenok, P.S.; Zykova, T.A.; Kozlovskaya, E.P. Isolation, properties and partial amino acid sequence of a new actinoporin from the sea anemone Radianthus macrodactylus. *Toxicon* **2004**, *44*, 315-324, doi:10.1016/j.toxicon.2004.06.006.
14. Monastyrnaya, M.M.; Zykova, T.A.; Apalikova, O.V.; Shwets, T.V.; Kozlovskaya, E.P. Biologically active polypeptides from the tropical sea anemone Radianthus macrodactylus. *Toxicon* **2002**, *40*, 1197-1217, doi:10.1016/s0041-0101(02)00139-3.
15. Khoo, K.S.; Kam, W.K.; Khoo, H.E.; Gopalakrishnakone, P.; Chung, M.C. Purification and partial characterization of two cytolysins from a tropical sea anemone, Heteractis magnifica. *Toxicon* **1993**, *31*, 1567-1579, doi:10.1016/0041-0101(93)90341-f.
16. Wang, Y.; Chua, K.L.; Khoo, H.E. A new cytolysin from the sea anemone, Heteractis magnifica: isolation, cDNA cloning and functional expression. *Biochim Biophys Acta* **2000**, *1478*, 9-18, doi:10.1016/s0167-4838(99)00289-7.
17. Il'ina, A.P.; Monastyrsnaia, M.M.; Sokotun, I.N.; Egorov Ts, A.; Nazarenko Iu, A.; Likhatskaia, G.N.; Kozlovskaia, E.P. Actinoporins from the Sea of Japan anemone Oulactis orientalis: isolation and partial characterization. *Bioorg Khim* **2005**, *31*, 39-48, doi:10.1007/s11171-005-0004-x.
18. Nagai, H.; Oshiro, N.; Takuwa-Kuroda, K.; Iwanaga, S.; Nozaki, M.; Nakajima, T. A new polypeptide toxin from the nematocyst venom of an Okinawan sea anemone Phyllodiscus semoni (Japanese name "unbachi-isoginchaku"). *Biosci Biotechnol Biochem* **2002**, *66*, 2621-2625, doi:10.1271/bbb.66.2621.
19. Jiang, X.Y.; Yang, W.L.; Chen, H.P.; Tu, H.B.; Wu, W.Y.; Wei, J.W.; Wang, J.; Liu, W.H.; Xu, A.L. Cloning and characterization of an acidic cytolysin cDNA from sea anemone Sagartia rosea. *Toxicon* **2002**, *40*, 1563-1569, doi:10.1016/s0041-0101(02)00173-3.
20. Blumenthal, K.M.; Kem, W.R. Primary structure of Stoichactis helianthus cytolysin III. *J Biol Chem* **1983**, *258*, 5574-5581, doi:10.1016/S0021-9258(20)81929-2.
21. Huerta, V.; Morera, V.; Guanche, Y.; Chinea, G.; González, L.; Betancourt, L.; Martınez, D.; Alvarez, C.; Lanio, M.; Besada, V. Primary structure of two cytolysin isoforms from Stichodactyla helianthus differing in their hemolytic activity. *Toxicon* **2001**, *39*, 1253-1256, doi:10.1016/s0041-0101(00)00247-6.
22. Bellomio, A.; Morante, K.; Barlic, A.; Gutierrez-Aguirre, I.; Viguera, A.R.; Gonzalez-Manas, J.M. Purification, cloning and characterization of fragaceatoxin C, a novel actinoporin from the sea anemone Actinia fragacea. *Toxicon* **2009**, *54*, 869-880, doi:10.1016/j.toxicon.2009.06.022.

23. Glasser, E.; Rachamim, T.; Aharonovich, D.; Sher, D. Hydra actinoporin-like toxin-1, an unusual hemolysin from the nematocyst venom of Hydra magnipapillata which belongs to an extended gene family. *Toxicon* **2014**, *91*, 103-113, doi:10.1016/j.toxicon.2014.04.004.
24. Yap, W.Y.; Tan, K.J.S.X.; Hwang, J.S. Expansion of Hydra actinoporin-like toxin (HALT) gene family: Expression divergence and functional convergence evolved through gene duplication. *Toxicon* **2019**, *170*, 10-20, doi:10.1016/j.toxicon.2019.09.007.
25. Honma, T.; Nagai, H.; Nagashima, Y.; Shiomi, K. Molecular cloning of an epidermal growth factor-like toxin and two sodium channel toxins from the sea anemone Stichodactyla gigantea. *Biochim Biophys Acta* **2003**, *1652*, 103-106, doi:10.1016/j.bbapap.2003.08.007.
26. Honma, T.; Kawahata, S.; Ishida, M.; Nagai, H.; Nagashima, Y.; Shiomi, K. Novel peptide toxins from the sea anemone Stichodactyla haddoni. *Peptides* **2008**, *29*, 536-544, doi:10.1016/j.peptides.2007.12.010.
27. Homma, T.; Ishida, M.; Nagashima, Y.; Shiomi, K. Isolation and cDNA cloning of four peptide toxins from the sea anemone Heteractis aurora. *J Venom Anim Toxins Incl Trop Dis* **2024**, *30*, e20240019, doi:10.1590/1678-9199-JVATITD-2024-0019.
28. Guo, Q.; Fu, J.; Yuan, L.; Liao, Y.; Li, M.; Li, X.; Yi, B.; Zhang, J.; Gao, B. Diversity analysis of sea anemone peptide toxins in different tissues of Heteractis crispa based on transcriptomics. *Sci Rep* **2024**, *14*, 7684, doi:10.1038/s41598-024-58402-2.
29. Sher, D.; Fishman, Y.; Zhang, M.; Lebendiker, M.; Gaathon, A.; Mancheno, J.M.; Zlotkin, E. Hydralysins, a new category of beta-pore-forming toxins in cnidaria. *J Biol Chem* **2005**, *280*, 22847-22855, doi:10.1074/jbc.M503242200.
30. Zhang, M.; Fishman, Y.; Sher, D.; Zlotkin, E. Hydralysin, a novel animal group-selective paralytic and cytolytic protein from a noncnidocystic origin in hydra. *Biochemistry* **2003**, *42*, 8939-8944, doi:10.1021/bi0343929.
31. Mitchell, M.L.; Hossain, M.A.; Lin, F.; Pinheiro-Junior, E.L.; Peigneur, S.; Wai, D.C.C.; Delaine, C.; Blyth, A.J.; Forbes, B.E.; Tytgat, J.; et al. Identification, Synthesis, Conformation and Activity of an Insulin-like Peptide from a Sea Anemone. *Biomolecules* **2021**, *11*, 1785, doi:10.3390/biom11121785.
32. Lassen, S.; Helmholz, H.; Ruhnau, C.; Prange, A. A novel proteinaceous cytotoxin from the northern Scyphozoa Cyanea capillata (L.) with structural homology to cubozoan haemolysins. *Toxicon* **2011**, *57*, 721-729, doi:10.1016/j.toxicon.2011.02.004.
33. Brinkman, D.; Burnell, J. Identification, cloning and sequencing of two major venom proteins from the box jellyfish, Chironex fleckeri. *Toxicon* **2007**, *50*, 850-860, doi:10.1016/j.toxicon.2007.06.016.
34. Nagai, H.; Takuwa-Kuroda, K.; Nakao, M.; Oshiro, N.; Iwanaga, S.; Nakajima, T. A novel protein toxin from the deadly box jellyfish (Sea Wasp, Habu-kurage) Chiropsalmus quadrigatus. *Biosci Biotechnol Biochem* **2002**, *66*, 97-102, doi:10.1271/bbb.66.97.
35. Nagai, H.; Takuwa, K.; Nakao, M.; Sakamoto, B.; Crow, G.L.; Nakajima, T. Isolation and characterization of a novel protein toxin from the Hawaiian box jellyfish (sea wasp) Carybdea alata. *Biochem Biophys Res Commun* **2000**, *275*, 589-594, doi:10.1006/bbrc.2000.3352.
36. Nagai, H.; Takuwa, K.; Nakao, M.; Ito, E.; Miyake, M.; Noda, M.; Nakajima, T. Novel proteinaceous toxins from the box jellyfish (sea wasp) Carybdea rastoni. *Biochem Biophys Res Commun* **2000**, *275*, 582-588, doi:10.1006/bbrc.2000.3353.

37. Brinkman, D.L.; Konstantakopoulos, N.; McInerney, B.V.; Mulvenna, J.; Seymour, J.E.; Isbister, G.K.; Hodgson, W.C.J.J.o.b.c. Chironex fleckeri (box jellyfish) venom proteins: expansion of a cnidarian toxin family that elicits variable cytolytic and cardiovascular effects. **2014**, *289*, 4798-4812.
38. Smith, H.L.; Prentis, P.J.; Bryan, S.E.; Norton, R.S.; Broszczak, D.A. Acontia, a Specialised Defensive Structure, Has Low Venom Complexity in Calliactis polypus. *Toxins (Basel)* **2023**, *15*, 218, doi:10.3390/toxins15030218.
39. Oshiro, N.; Kobayashi, C.; Iwanaga, S.; Nozaki, M.; Namikoshi, M.; Spring, J.; Nagai, H. A new membrane-attack complex/perforin (MACPF) domain lethal toxin from the nematocyst venom of the Okinawan sea anemone Actineria villosa. *Toxicon* **2004**, *43*, 225-228, doi:10.1016/j.toxicon.2003.11.017.
40. Nagai, H.; Oshiro, N.; Takuwa-Kuroda, K.; Iwanaga, S.; Nozaki, M.; Nakajima, T. Novel proteinaceous toxins from the nematocyst venom of the Okinawan sea anemone Phyllodiscus semoni Kwietniewski. *Biochem Biophys Res Commun* **2002**, *294*, 760-763, doi:10.1016/S0006-291X(02)00547-8.
41. Moran, Y.; Praher, D.; Schlesinger, A.; Ayalon, A.; Tal, Y.; Technau, U. Analysis of soluble protein contents from the nematocysts of a model sea anemone sheds light on venom evolution. *Mar Biotechnol (NY)* **2013**, *15*, 329-339, doi:10.1007/s10126-012-9491-y.
42. Columbus-Shenkar, Y.Y.; Sachkova, M.Y.; Macrander, J.; Fridrich, A.; Modepalli, V.; Reitzel, A.M.; Sunagar, K.; Moran, Y. Dynamics of venom composition across a complex life cycle. *Elife* **2018**, *7*, e35014, doi:10.7554/eLife.35014.
43. Nesher, N.; Shapira, E.; Sher, D.; Moran, Y.; Tsveyer, L.; Turchetti-Maia, A.L.; Horowitz, M.; Hochner, B.; Zlotkin, E. AdE-1, a new inotropic Na+ channel toxin from Aiptasia diaphana, is similar to, yet distinct from, known anemone Na+ channel toxins. *Biochemical Journal* **2013**, *451*, 81-90.
44. Cariello, L.; de Santis, A.; Fiore, F.; Piccoli, R.; Spagnuolo, A.; Zanetti, L.; Parente, A. Calitoxin, a neurotoxic peptide from the sea anemone Calliactis parasitica: amino acid sequence and electrophysiological properties. *Biochemistry* **1989**, *28*, 2484-2489, doi:10.1021/bi00432a020.
45. Spagnuolo, A.; Zanetti, L.; Cariello, L.; Piccoli, R. Isolation and characterization of two genes encoding calitoxins, neurotoxic peptides from Calliactis parasitica (Cnidaria). *Gene* **1994**, *138*, 187-191, doi:10.1016/0378-1119(94)90805-2.
46. Talvinen, K.A.; Nevalainen, T.J. Cloning of a novel phospholipase A2 from the cnidarian Adamsia carciniopados. *Comp Biochem Physiol B Biochem Mol Biol* **2002**, *132*, 571-578, doi:10.1016/s1096-4959(02)00073-8.
47. Romero, L.; Marcussi, S.; Marchi-Salvador, D.P.; Silva, F.P., Jr.; Fuly, A.L.; Stabeli, R.G.; da Silva, S.L.; Gonzalez, J.; Monte, A.D.; Soares, A.M. Enzymatic and structural characterization of a basic phospholipase A(2) from the sea anemone Condylactis gigantea. *Biochimie* **2010**, *92*, 1063-1071, doi:10.1016/j.biochi.2010.05.007.
48. Lotan, A.; Fishman, L.; Loya, Y.; Zlotkin, E. Delivery of a nematocyst toxin. *Nature* **1995**, *375*, 456, doi:10.1038/375456a0.

49. Razpotnik, A.; Krizaj, I.; Sribar, J.; Kordis, D.; Macek, P.; Frangez, R.; Kem, W.R.; Turk, T. A new phospholipase A2 isolated from the sea anemone Urticina crassicornis - its primary structure and phylogenetic classification. *FEBS J* **2010**, *277*, 2641-2653, doi:10.1111/j.1742-464X.2010.07674.x.
50. Martins, R.D.; Alves, R.S.; Martins, A.M.; Barbosa, P.S.; Evangelista, J.S.; Evangelista, J.J.; Ximenes, R.M.; Toyama, M.H.; Toyama, D.O.; Souza, A.J.; et al. Purification and characterization of the biological effects of phospholipase A(2) from sea anemone Bunodosoma caissarum. *Toxicon* **2009**, *54*, 413-420, doi:10.1016/j.toxicon.2009.05.005.
51. Logashina, Y.A.; Solstad, R.G.; Mineev, K.S.; Korolkova, Y.V.; Mosharova, I.V.; Dyachenko, I.A.; Palikov, V.A.; Palikova, Y.A.; Murashev, A.N.; Arseniev, A.S.J.T. New disulfide-stabilized fold provides sea anemone peptide to exhibit both antimicrobial and TRPA1 potentiating properties. **2017**, *9*, 154, doi:10.3390/toxins9050154.
52. Jouiaei, M.; Sunagar, K.; Federman Gross, A.; Scheib, H.; Alewood, P.F.; Moran, Y.; Fry, B.G. Evolution of an ancient venom: recognition of a novel family of cnidarian toxins and the common evolutionary origin of sodium and potassium neurotoxins in sea anemone. *Mol Biol Evol* **2015**, *32*, 1598-1610, doi:10.1093/molbev/msv050.
53. Sunagawa, S.; DeSalvo, M.K.; Voolstra, C.R.; Reyes-Bermudez, A.; Medina, M. Identification and gene expression analysis of a taxonomically restricted cysteine-rich protein family in reef-building corals. *PLoS One* **2009**, *4*, e4865, doi:10.1371/journal.pone.0004865.
54. Surm, J.M.; Smith, H.L.; Madio, B.; Undheim, E.A.B.; King, G.F.; Hamilton, B.R.; van der Burg, C.A.; Pavasovic, A.; Prentis, P.J. A process of convergent amplification and tissue-specific expression dominates the evolution of toxin and toxin-like genes in sea anemones. *Mol Ecol* **2019**, *28*, 2272-2289, doi:10.1111/mec.15084.
55. Kozlov, S.; Grishin, E. The mining of toxin-like polypeptides from EST database by single residue distribution analysis. *BMC Genomics* **2011**, *12*, 88, doi:10.1186/1471-2164-12-88.
56. Logashina, Y.A.; Mosharova, I.V.; Korolkova, Y.V.; Shelukhina, I.V.; Dyachenko, I.A.; Palikov, V.A.; Palikova, Y.A.; Murashev, A.N.; Kozlov, S.A.; Stensvag, K.; et al. Peptide from Sea Anemone Metridium senile Affects Transient Receptor Potential Ankyrin-repeat 1 (TRPA1) Function and Produces Analgesic Effect. *J Biol Chem* **2017**, *292*, 2992-3004, doi:10.1074/jbc.M116.757369.
57. Osmakov, D.I.; Kozlov, S.A.; Andreev, Y.A.; Koshelev, S.G.; Sanamyan, N.P.; Sanamyan, K.E.; Dyachenko, I.A.; Bondarenko, D.A.; Murashev, A.N.; Mineev, K.S.; et al. Sea anemone peptide with uncommon beta-hairpin structure inhibits acid-sensing ion channel 3 (ASIC3) and reveals analgesic activity. *J Biol Chem* **2013**, *288*, 23116-23127, doi:10.1074/jbc.M113.485516.
58. Honma, T.; Hasegawa, Y.; Ishida, M.; Nagai, H.; Nagashima, Y.; Shiomi, K. Isolation and molecular cloning of novel peptide toxins from the sea anemone Antheopsis maculata. *Toxicon* **2005**, *45*, 33-41, doi:10.1016/j.toxicon.2004.09.013.
59. Zaharenko, A.J.; Ferreira, W.A., Jr.; Oliveira, J.S.; Richardson, M.; Pimenta, D.C.; Konno, K.; Portaro, F.C.; de Freitas, J.C. Proteomics of the neurotoxic fraction from the sea anemone Bunodosoma cangicum venom: Novel peptides belonging to new classes of toxins. *Comp Biochem Physiol Part D Genomics Proteomics* **2008**, *3*, 219-225, doi:10.1016/j.cbd.2008.04.002.

60. Cassoli, J.S.; Verano-Braga, T.; Oliveira, J.S.; Montandon, G.G.; Cologna, C.T.; Peigneur, S.; de Castro Pimenta, A.M.; Kjeldsen, F.; Roepstorff, P.; Tytgat, J. The proteomic profile of Stichodactyla duerdeni secretion reveals the presence of a novel O-linked glycopeptide. *Journal of proteomics* **2013**, *87*, 89-102.
61. Madio, B.; Peigneur, S.; Chin, Y.K.Y.; Hamilton, B.R.; Henriques, S.T.; Smith, J.J.; Cristofori-Armstrong, B.; Dekan, Z.; Boughton, B.A.; Alewood, P.F.; et al. PHAB toxins: a unique family of predatory sea anemone toxins evolving via intra-gene concerted evolution defines a new peptide fold. *Cell Mol Life Sci* **2018**, *75*, 4511-4524, doi:10.1007/s00018-018-2897-6.
62. Castaneda, O.; Sotolongo, V.; Amor, A.M.; Stocklin, R.; Anderson, A.J.; Harvey, A.L.; Engstrom, A.; Wernstedt, C.; Karlsson, E. Characterization of a potassium channel toxin from the Caribbean Sea anemone Stichodactyla helianthus. *Toxicon* **1995**, *33*, 603-613, doi:10.1016/0041-0101(95)00013-c.
63. Hasegawa, Y.; Honma, T.; Nagai, H.; Ishida, M.; Nagashima, Y.; Shiomi, K. Isolation and cDNA cloning of a potassium channel peptide toxin from the sea anemone Anemonia erythraea. *Toxicon* **2006**, *48*, 536-542, doi:10.1016/j.toxicon.2006.07.002.
64. Gendeh, G.S.; Young, L.C.; de Medeiros, C.L.; Jeyaseelan, K.; Harvey, A.L.; Chung, M.C. A new potassium channel toxin from the sea anemone Heteractis magnifica: isolation, cDNA cloning, and functional expression. *Biochemistry* **1997**, *36*, 11461-11471, doi:10.1021/bi970253d.
65. Ashwood, L.M.; Undheim, E.A.B.; Madio, B.; Hamilton, B.R.; Daly, M.; Hurwood, D.A.; King, G.F.; Prentis, P.J. Venoms for all occasions: The functional toxin profiles of different anatomical regions in sea anemones are related to their ecological function. *Mol Ecol* **2022**, *31*, 866-883, doi:10.1111/mec.16286.
66. Yamaguchi, Y.; Hasegawa, Y.; Honma, T.; Nagashima, Y.; Shiomi, K. Screening and cDNA cloning of Kv1 potassium channel toxins in sea anemones. *Mar Drugs* **2010**, *8*, 2893-2905, doi:10.3390/md8122893.
67. Aneiros, A.; Garcia, I.; Martinez, J.R.; Harvey, A.L.; Anderson, A.J.; Marshall, D.L.; Engstrom, A.; Hellman, U.; Karlsson, E. A potassium channel toxin from the secretion of the sea anemone Bunodosoma granulifera. Isolation, amino acid sequence and biological activity. *Biochim Biophys Acta* **1993**, *1157*, 86-92, doi:10.1016/0304-4165(93)90082-j.
68. Minagawa, S.; Ishida, M.; Nagashima, Y.; Shiomi, K. Primary structure of a potassium channel toxin from the sea anemone Actinia equina. *FEBS Lett* **1998**, *427*, 149-151, doi:10.1016/s0014-5793(98)00403-7.
69. Schweitz, H.; Bruhn, T.; Guillemare, E.; Moinier, D.; Lancelin, J.-M.; Béress, L.; Lazdunski, M.J.J.o.B.C. Kalicludines and kaliseptine. Two different classes of sea anemone toxins for voltage sensitive K+ channels. **1995**, *270*, 25121-25126, doi:10.1074/jbc.270.42.25121.
70. Orts, D.J.; Peigneur, S.; Madio, B.; Cassoli, J.S.; Montandon, G.G.; Pimenta, A.M.; Bicudo, J.E.; Freitas, J.C.; Zaharenko, A.J.; Tytgat, J. Biochemical and electrophysiological characterization of two sea anemone type 1 potassium toxins from a geographically distant population of Bunodosoma caissarum. *Mar Drugs* **2013**, *11*, 655-679, doi:10.3390/md11030655.
71. Krebs, H.; Habermehl, G. Isolation and structural determination of a hemolytic active peptide from the sea anemone Metridium senile. *Naturwissenschaften* **1987**, *74*, 395-396.

72. Sunanda, P.; Krishnarjuna, B.; Peigneur, S.; Mitchell, M.L.; Estrada, R.; Villegas-Moreno, J.; Pennington, M.W.; Tytgat, J.; Norton, R.S. Identification, chemical synthesis, structure, and function of a new KV1 channel blocking peptide from Oulactis sp. *Peptide Science* **2018**, *110*, e24073, doi:10.1002/pep2.24073.
73. Krishnarjuna, B.; Villegas-Moreno, J.; Mitchell, M.L.; Csoti, A.; Peigneur, S.; Amero, C.; Pennington, M.W.; Tytgat, J.; Panyi, G.; Norton, R.S. Synthesis, folding, structure and activity of a predicted peptide from the sea anemone Oulactis sp. with an ShKT fold. *Toxicon* **2018**, *150*, 50-59, doi:10.1016/j.toxicon.2018.05.006.
74. Beress, L.; Beress, R.; Wunderer, G. Isolation and characterisation of three polypeptides with neurotoxic activity from Anemonia sulcata. *FEBS Lett* **1975**, *50*, 311-314, doi:10.1016/0014-5793(75)80517-5.
75. Bruhn, T.; Schaller, C.; Schulze, C.; Sanchez-Rodriguez, J.; Dannmeier, C.; Ravens, U.; Heubach, J.F.; Eckhardt, K.; Schmidtmayer, J.; Schmidt, H.; et al. Isolation and characterisation of five neurotoxic and cardiotoxic polypeptides from the sea anemone Anthopleura elegantissima. *Toxicon* **2001**, *39*, 693-702, doi:10.1016/s0041-0101(00)00199-9.
76. Norton, T. Cardiotonic polypeptides from Anthopleura xanthogrammica (Brandt) and A. elegantissima (Brandt). In Proceedings of the Federation Proceedings, 1981; pp. 21-25.
77. Sunahara, S.; Muramoto, K.; Tenma, K.; Kamiya, H. Amino acid sequence of two sea anemone toxins from Anthopleura fuscoviridis. *Toxicon* **1987**, *25*, 211-219, doi:10.1016/0041-0101(87)90243-1.
78. Wang, L.; Ou, J.; Peng, L.; Zhong, X.; Du, J.; Liu, Y.; Huang, Y.; Liu, W.; Zhang, Y.; Dong, M.; et al. Functional expression and characterization of four novel neurotoxins from sea anemone Anthopleura sp. *Biochem Biophys Res Commun* **2004**, *313*, 163-170, doi:10.1016/j.bbrc.2003.11.102.
79. Liu, W.H.; Wang, L.; Wang, Y.L.; Peng, L.S.; Wu, W.Y.; Peng, W.L.; Jiang, X.Y.; Tu, H.B.; Chen, H.P.; Ou-Yang, P.; et al. Cloning and characterization of a novel neurotoxin from the sea anemone Anthopleura sp. *Toxicon* **2003**, *41*, 793-801, doi:10.1016/s0041-0101(03)00033-3.
80. Tanaka, M.; Hainu, M.; Yasunobu, K.T.; Norton, T.R. Amino acid sequence of the Anthopleura xanthogrammica heart stimulant, anthopleurin A. *Biochemistry* **1977**, *16*, 204-208, doi:10.1021/bi00621a007.
81. Reimer, N.S.; Yasunobu, C.L.; Yasunobu, K.T.; Norton, T.R. Amino acid sequence of the Anthopleura xanthogrammica heart stimulant, anthopleurin-B. *J Biol Chem* **1985**, *260*, 8690-8693, doi:10.1016/S0021-9258(17)39403-6
82. Loret, E.P.; del Valle, R.M.; Mansuelle, P.; Sampieri, F.; Rochat, H. Positively charged amino acid residues located similarly in sea anemone and scorpion toxins. *J Biol Chem* **1994**, *269*, 16785-16788, doi:10.1016/S0021-9258(19)89460-7.
83. Moran, Y.; Weinberger, H.; Sullivan, J.C.; Reitzel, A.M.; Finnerty, J.R.; Gurevitz, M. Concerted Evolution of Sea Anemone Neurotoxin Genes Is Revealed through Analysis of the Nematostella vectensis Genome. *Molecular Biology and Evolution* **2008**, *25*, 737-747, doi:10.1093/molbev/msn021 %J Molecular Biology and Evolution.

84. Lin, X.Y.; Ishida, M.; Nagashima, Y.; Shiomi, K. A polypeptide toxin in the sea anemone Actinia equina homologous with other sea anemone sodium channel toxins: isolation and amino acid sequence. *Toxicon* **1996**, *34*, 57-65, doi:10.1016/0041-0101(95)00121-2.
85. Shiomi, K.; Qian, W.H.; Lin, X.Y.; Shimakura, K.; Nagashima, Y.; Ishida, M. Novel polypeptide toxins with crab lethality from the sea anemone Anemonia erythraea. *Biochim Biophys Acta* **1997**, *1335*, 191-198, doi:10.1016/s0304-4165(96)00137-7.
86. Scheffler, J.J.; Tsugita, A.; Linden, G.; Schweitz, H.; Lazdunski, M. The amino acid sequence of toxin V from Anemonia sulcata. *Biochem Biophys Res Commun* **1982**, *107*, 272-278, doi:10.1016/0006-291x(82)91700-4.
87. Kelso, G.J.; Blumenthal, K.M. Identification and characterization of novel sodium channel toxins from the sea anemone Anthopleura xanthogrammica. *Toxicon* **1998**, *36*, 41-51, doi:10.1016/s0041-0101(97)00064-0.
88. Malpezzi, E.L.; de Freitas, J.; Muramoto, K.; Kamiya, H. Characterization of peptides in sea anemone venom collected by a novel procedure. *Toxicon* **1993**, *31*, 853-864.
89. Zaharenko, A.J.; Ferreira, W.A., Jr.; de Oliveira, J.S.; Konno, K.; Richardson, M.; Schiavon, E.; Wanke, E.; de Freitas, J.C. Revisiting cangitoxin, a sea anemone peptide: purification and characterization of cangitoxins II and III from the venom of Bunodosoma cangicum. *Toxicon* **2008**, *51*, 1303-1307, doi:10.1016/j.toxicon.2008.01.011.
90. Cunha, R.B.; Santana, A.N.; Amaral, P.C.; Carvalho, M.D.; Carvalho, D.M.; Cavalheiro, E.A.; Maigret, B.; Ricart, C.A.; Cardi, B.A.; Sousa, M.V.; et al. Primary structure, behavioral and electroencephalographic effects of an epileptogenic peptide from the sea anemone Bunodosoma cangicum. *Toxicon* **2005**, *45*, 207-217, doi:10.1016/j.toxicon.2004.10.011.
91. van Losenoord, W.; Krause, J.; Parker-Nance, S.; Krause, R.; Stoychev, S.; Frost, C.L. Purification and biochemical characterisation of a putative sodium channel agonist secreted from the South African Knobbly sea anemone Bunodosoma capense. *Toxicon* **2019**, *168*, 147-157, doi:10.1016/j.toxicon.2019.06.222.
92. Shiomi, K.; Lin, X.-Y.; Nagashima, Y.; Ishida, M. Isolation and amino acid sequences of polypeptide toxins in the Caribbean sea anemone Condylactis passiflora. *Fisheries science* **1995**, *61*, 1016-1021, doi:doi.org/10.2331/fishsci.61.1016.
93. Babenko, V.V.; Mikov, A.N.; Manuvera, V.A.; Anikanov, N.A.; Kovalchuk, S.I.; Andreev, Y.A.; Logashina, Y.A.; Kornilov, D.A.; Manolov, A.I.; Sanamyan, N.P.; et al. Identification of unusual peptides with new Cys frameworks in the venom of the cold-water sea anemone Cnidopus japonicus. *Sci Rep* **2017**, *7*, 14534, doi:10.1038/s41598-017-14961-1.
94. Shiomi, K.; Lin, X.-Y.; Nagashima, Y.; Ishida, M. Isolation and amino acid sequence of a polypeptide toxin from the sea anemone Radianthus crispus. *Fisheries science* **1996**, *62*, 629-633, doi:doi.org/10.2331/fishsci.62.629.
95. Andreev, Y.A.; Kozlov, S.A.; Koshelev, S.G.; Ivanova, E.A.; Monastyrnaya, M.M.; Kozlovskaya, E.P.; Grishin, E.V.J.J.o.B.C. Analgesic compound from sea anemone Heteractis crispa is the first polypeptide inhibitor of vanilloid receptor 1 (TRPV1). **2008**, *283*, 23914-23921, doi:10.1074/jbc.M800776200.
96. Kozlov, S.A.; Andreev Ia, A.; Murashev, A.N.; Skobtsov, D.I.; D'Iachenko I, A.; Grishin, E.V. New polypeptide components from the Heteractis crispa sea anemone with analgesic activity. *Bioorg Khim* **2009**, *35*, 789-798, doi:10.1134/s1068162009060065.

97. Isaeva, M.P.; Chausova, V.E.; Zelepuga, E.A.; Guzev, K.V.; Tabakmakher, V.M.; Monastyrnaya, M.M.; Kozlovskaya, E.P. A new multigene superfamily of Kunitz-type protease inhibitors from sea anemone Heteractis crispa. *Peptides* **2012**, *34*, 88-97, doi:10.1016/j.peptides.2011.09.022.
98. Kvetkina, A.; Leychenko, E.; Chausova, V.; Zelepuga, E.; Chernysheva, N.; Guzev, K.; Pislyagin, E.; Yurchenko, E.; Menchinskaya, E.; Aminin, D.; et al. A new multigene HCIQ subfamily from the sea anemone Heteractis crispa encodes Kunitz-peptides exhibiting neuroprotective activity against 6-hydroxydopamine. *Sci Rep* **2020**, *10*, 4205, doi:10.1038/s41598-020-61034-x.
99. Monastyrnaya, M.; Peigneur, S.; Zelepuga, E.; Sintsova, O.; Gladkikh, I.; Leychenko, E.; Isaeva, M.; Tytgat, J.; Kozlovskaya, E.J.M.d. Kunitz-type peptide HCRG21 from the sea anemone Heteractis crispa is a full antagonist of the TRPV1 receptor. **2016**, *14*, 229, doi:10.3390/md14120229.
100. Sokotun, I.N.; Il'ina, A.P.; Monastyrnaya, M.M.; Leychenko, E.V.; Es'kov, A.A.; Anastuk, S.D.; Kozlovskaya, E.P. Proteinase inhibitors from the tropical sea anemone Radianthus macrodactylus: isolation and characteristic. *Biochemistry (Mosc)* **2007**, *72*, 301-306, doi:10.1134/s0006297907030078.
101. Gladkikh, I.; Monastyrnaya, M.; Zelepuga, E.; Sintsova, O.; Tabakmakher, V.; Gnedenko, O.; Ivanov, A.; Hua, K.F.; Kozlovskaya, E. New Kunitz-Type HCRG Polypeptides from the Sea Anemone Heteractis crispa. *Mar Drugs* **2015**, *13*, 6038-6063, doi:10.3390/md13106038.
102. Ishida, M.; Minagawa, S.; Miyauchi, K.; Shimakura, K.; Nagashima, Y.; Shiomi, K. Amino acid sequences of Kunitz-type protease inhibitors from the sea anemone Actinia equina. *Fisheries science* **1997**, *63*, 794-798, doi:10.1016/j.cbpb.2008.03.010.
103. Chen, X.; Leahy, D.; Van Haeften, J.; Hartfield, P.; Prentis, P.J.; van der Burg, C.A.; Surm, J.M.; Pavasovic, A.; Madio, B.; Hamilton, B.R.; et al. A Versatile and Robust Serine Protease Inhibitor Scaffold from Actinia tenebrosa. *Mar Drugs* **2019**, *17*, 701, doi:10.3390/md17120701.
104. Peigneur, S.; Billen, B.; Derua, R.; Waelkens, E.; Debaveye, S.; Beress, L.; Tytgat, J. A bifunctional sea anemone peptide with Kunitz type protease and potassium channel inhibiting properties. *Biochem Pharmacol* **2011**, *82*, 81-90, doi:10.1016/j.bcp.2011.03.023.
105. Liao, Q.; Li, S.; Siu, S.W.I.; Yang, B.; Huang, C.; Chan, J.Y.-W.; Morlighem, J.-E.R.; Wong, C.T.T.; Radis-Baptista, G.; Lee, S.M.-Y. Novel Kunitz-like peptides discovered in the zoanthid Palythoa caribaeorum through transcriptome sequencing. *Journal of proteome research* **2018**, *17*, 891-902.
106. Kvetkina, A.; Leychenko, E.; Yurchenko, E.; Pislyagin, E.; Peigneur, S.; Tytgat, Y.; Isaeva, M.; Aminin, D.; Kozlovskaya, E. A new Iq-peptide of the Kunitz type from the Heteractis magnifica sea anemone exhibits neuroprotective activity in a model of Alzheimer’s disease. *Russian Journal of Bioorganic Chemistry* **2018**, *44*, 416-423, doi:doi.org/10.1134/S106816201804012X.
107. Kvetkina, A.; Pislyagin, E.; Menchinskaya, E.; Yurchenko, E.; Kalina, R.; Kozlovskiy, S.; Kaluzhskiy, L.; Menshov, A.; Kim, N.; Peigneur, S.; et al. Kunitz-Type Peptides from Sea Anemones Protect Neuronal Cells against Parkinson's Disease Inductors via Inhibition of ROS Production and ATP-Induced P2X7 Receptor Activation. *Int J Mol Sci* **2022**, *23*, 5115, doi:10.3390/ijms23095115.
108. Kem, W.R.; Parten, B.; Pennington, M.W.; Price, D.A.; Dunn, B.M. Isolation, characterization, and amino acid sequence of a polypeptide neurotoxin occurring in the sea anemone Stichodactyla helianthus. *Biochemistry* **1989**, *28*, 3483-3489, doi:10.1021/bi00434a050.

109. Maeda, M.; Honma, T.; Shiomi, K. Isolation and cDNA cloning of type 2 sodium channel peptide toxins from three species of sea anemones (Cryptodendrum adhaesivum, Heterodactyla hemprichii and Thalassianthus aster) belonging to the family Thalassianthidae. *Comp Biochem Physiol B Biochem Mol Biol* **2010**, *157*, 389-393, doi:10.1016/j.cbpb.2010.08.008.
110. Ishida, M.; Yokoyama, A.; Shimakura, K.; Nagashima, Y.; Shiomi, K. Halcurin, a polypeptide toxin from the sea anemone Halcurias sp., with a structural resemblance to type 1 and 2 toxins. *Toxicon* **1997**, *35*, 537-544, doi:10.1016/s0041-0101(96)00143-2.
111. Schweitz, H.; Bidard, J.N.; Frelin, C.; Pauron, D.; Vijverberg, H.P.; Mahasneh, D.M.; Lazdunski, M.; Vilbois, F.; Tsugita, A. Purification, sequence, and pharmacological properties of sea anemone toxins from Radianthus paumotensis. A new class of sea anemone toxins acting on the sodium channel. *Biochemistry* **1985**, *24*, 3554-3561, doi:10.1021/bi00335a025.
112. Zykova, T. Amino-acid sequence of neurotoxin III from the sea anemone Radianthus macrodactylus. *Bioorg Khim* **1985**, *11*, 302-310.
113. Zykova, T.A.; Kozlovskaia, E.P.; Eliakov, G.B. Amino acid sequence of neurotoxin II from the sea anemone Radianthus macrodactylus. *Bioorg Khim* **1988**, *14*, 878-882.
114. Zykova, T.A.; Kozlovskaia, E.P.; Eliakov, G.B. Amino acid sequence of neurotoxins IV and V from the sea anemone Radianthus macrodactylus. *Bioorg Khim* **1988**, *14*, 1489-1494.
115. Zykova, T.A.; Kozlovskaia, E.P. Amino acid sequence of a neurotoxin from the anemone Radianthus macrodactylus. *Bioorg Khim* **1989**, *15*, 1301-1306.
116. Diochot, S.; Baron, A.; Rash, L.D.; Deval, E.; Escoubas, P.; Scarzello, S.; Salinas, M.; Lazdunski, M. A new sea anemone peptide, APETx2, inhibits ASIC3, a major acid-sensitive channel in sensory neurons. *EMBO J* **2004**, *23*, 1516-1525, doi:10.1038/sj.emboj.7600177.
117. Kalina, R.; Gladkikh, I.; Dmitrenok, P.; Chernikov, O.; Koshelev, S.; Kvetkina, A.; Kozlov, S.; Kozlovskaya, E.; Monastyrnaya, M. New APETx-like peptides from sea anemone Heteractis crispa modulate ASIC1a channels. *Peptides* **2018**, *104*, 41-49, doi:10.1016/j.peptides.2018.04.013.
118. Moreels, L.; Peigneur, S.; Galan, D.T.; De Pauw, E.; Beress, L.; Waelkens, E.; Pardo, L.A.; Quinton, L.; Tytgat, J. APETx4, a Novel Sea Anemone Toxin and a Modulator of the Cancer-Relevant Potassium Channel K(V)10.1. *Mar Drugs* **2017**, *15*, 287, doi:10.3390/md15090287.
119. Diochot, S.; Schweitz, H.; Beress, L.; Lazdunski, M. Sea anemone peptides with a specific blocking activity against the fast inactivating potassium channel Kv3.4. *J Biol Chem* **1998**, *273*, 6744-6749, doi:10.1074/jbc.273.12.6744.
120. Kalina, R.S.; Kasheverov, I.E.; Koshelev, S.G.; Sintsova, O.V.; Peigneur, S.; Pinheiro-Junior, E.L.; Popov, R.S.; Chausova, V.E.; Monastyrnaya, M.M.; Dmitrenok, P.S.; et al. Nicotinic Acetylcholine Receptors Are Novel Targets of APETx-like Toxins from the Sea Anemone Heteractis magnifica. *Toxins (Basel)* **2022**, *14*, 697, doi:10.3390/toxins14100697.
121. Diochot, S.; Loret, E.; Bruhn, T.; Beress, L.; Lazdunski, M. APETx1, a new toxin from the sea anemone Anthopleura elegantissima, blocks voltage-gated human ether-a-go-go-related gene potassium channels. *Mol Pharmacol* **2003**, *64*, 59-69, doi:10.1124/mol.64.1.59.
122. Rojas, L.; Cabrera-Munoz, A.; Espinosa, L.A.; Montane, S.; Alvarez-Lajonchere, L.; Mojarena, J.D.; Moya, G.; Lorenzo, J.; Gonzalez, L.J.; Betzel, C.; et al. CogiTx1: A novel subtilisin A inhibitor isolated from the sea anemone Condylactis gigantea belonging to the defensin 4 protein family. *Biochimie* **2023**, *213*, 41-53, doi:10.1016/j.biochi.2023.04.015.

123. Kim, C.H.; Lee, Y.J.; Go, H.J.; Oh, H.Y.; Lee, T.K.; Park, J.B.; Park, N.G. Defensin-neurotoxin dyad in a basally branching metazoan sea anemone. *FEBS J* **2017**, *284*, 3320-3338, doi:10.1111/febs.14194.
124. Peigneur, S.; Béress, L.; Möller, C.; Marí, F.; Forssmann, W.G.; Tytgat, J. A natural point mutation changes both target selectivity and mechanism of action of sea anemone toxins. *The FASEB Journal* **2012**, *26*, 5141-5151, doi:10.1096/fj.12-218479.
125. Kozlov, S.A.; Osmakov, D.I.; Andreev Ia, A.; Koshelev, S.G.; Gladkikh, I.N.; Monastyrnaia, M.M.; Kozlovskaia, E.P.; Grishin, E.V. Polypeptide toxin from sea anemone inhibiting proton-sensitive channel ASIC3. *Bioorg Khim* **2012**, *38*, 653-659, doi:10.1134/s1068162012060064.
126. Nicosia, A.; Maggio, T.; Mazzola, S.; Cuttitta, A. Evidence of accelerated evolution and ectodermal-specific expression of presumptive BDS toxin cDNAs from Anemonia viridis. *Mar Drugs* **2013**, *11*, 4213-4231, doi:10.3390/md11114213.
127. Loret, E.P.; Luis, J.; Nuccio, C.; Villard, C.; Mansuelle, P.; Lebrun, R.; Villard, P.H. A Low Molecular Weight Protein from the Sea Anemone Anemonia viridis with an Anti-Angiogenic Activity. *Mar Drugs* **2018**, *16*, 134, doi:10.3390/md16040134.
128. Richier, S.; Rodriguez-Lanetty, M.; Schnitzler, C.E.; Weis, V.M. Response of the symbiotic cnidarian Anthopleura elegantissima transcriptome to temperature and UV increase. *Comp Biochem Physiol Part D Genomics Proteomics* **2008**, *3*, 283-289, doi:10.1016/j.cbd.2008.08.001.
129. Oliveira, J.S.; Zaharenko, A.J.; Ferreira Jr, W.A.; Konno, K.; Shida, C.S.; Richardson, M.; Lúcio, A.D.; Beirão, P.S.L.; de Freitas, J.C. BcIV, a new paralyzing peptide obtained from the venom of the sea anemone Bunodosoma caissarum. A comparison with the Na+ channel toxin BcIII. *Biochimica et Biophysica Acta (BBA)-Proteins and Proteomics* **2006**, *1764*, 1592-1600.
130. Rodriguez, A.A.; Cassoli, J.S.; Sa, F.; Dong, Z.Q.; de Freitas, J.C.; Pimenta, A.M.; de Lima, M.E.; Konno, K.; Lee, S.M.; Garateix, A.; et al. Peptide fingerprinting of the neurotoxic fractions isolated from the secretions of sea anemones Stichodactyla helianthus and Bunodosoma granulifera. New members of the APETx-like family identified by a 454 pyrosequencing approach. *Peptides* **2012**, *34*, 26-38, doi:10.1016/j.peptides.2011.10.011.
131. Rodriguez, A.A.; Garateix, A.; Salceda, E.; Peigneur, S.; Zaharenko, A.J.; Pons, T.; Santos, Y.; Arreguin, R.; Standker, L.; Forssmann, W.G.; et al. PhcrTx2, a New Crab-Paralyzing Peptide Toxin from the Sea Anemone Phymanthus crucifer. *Toxins (Basel)* **2018**, *10*, 72, doi:10.3390/toxins10020072.
132. Moran, Y.; Weinberger, H.; Lazarus, N.; Gur, M.; Kahn, R.; Gordon, D.; Gurevitz, M. Fusion and retrotransposition events in the evolution of the sea anemone Anemonia viridis neurotoxin genes. *J Mol Evol* **2009**, *69*, 115-124, doi:10.1007/s00239-009-9258-x.
133. Honma, T.; Iso, T.; Ishida, M.; Nagashima, Y.; Shiomi, K. Occurrence of type 3 sodium channel peptide toxins in two species of sea anemones (Dofleinia armata and Entacmaea ramsayi). *Toxicon* **2003**, *41*, 637-639, doi:10.1016/s0041-0101(02)00368-9.
134. Orts, D.J.; Moran, Y.; Cologna, C.T.; Peigneur, S.; Madio, B.; Praher, D.; Quinton, L.; De Pauw, E.; Bicudo, J.E.; Tytgat, J.J.T.F.j. Bcs T x3 is a founder of a novel sea anemone toxin family of potassium channel blocker. **2013**, *280*, 4839-4852, doi:10.1111/febs.12456.

135. Putnam, N.H.; Srivastava, M.; Hellsten, U.; Dirks, B.; Chapman, J.; Salamov, A.; Terry, A.; Shapiro, H.; Lindquist, E.; Kapitonov, V.V.; et al. Sea anemone genome reveals ancestral eumetazoan gene repertoire and genomic organization. *Science* **2007**, *317*, 86-94, doi:10.1126/science.1139158.
136. B. Orts, D.J.; Peigneur, S.; Silva-Gonçalves, L.C.; Arcisio-Miranda, M.; PW Bicudo, J.E.; Tytgat, J. AbeTx1 is a novel sea anemone toxin with a dual mechanism of action on shaker-type K+ channels activation. *Marine Drugs* **2018**, *16*, 360, doi:10.3390/md16100360.
137. Cline, E.I.; Wiebe, L.I.; Young, J.D.; Samuel, J. Toxic effects of the novel protein UpI from the sea anemone Urticina piscivora. *Pharmacol Res* **1995**, *32*, 309-314, doi:10.1016/s1043-6618(05)80020-9.
138. Elnahriry, K.A.; Wai, D.C.C.; Krishnarjuna, B.; Badawy, N.N.; Chittoor, B.; MacRaild, C.A.; Williams-Noonan, B.J.; Surm, J.M.; Chalmers, D.K.; Zhang, A.H.; et al. Structural and functional characterisation of a novel peptide from the Australian sea anemone Actinia tenebrosa. *Toxicon* **2019**, *168*, 104-112, doi:10.1016/j.toxicon.2019.07.002.
139. Rodríguez, A.A.; Salceda, E.; Garateix, A.G.; Zaharenko, A.J.; Peigneur, S.; López, O.; Pons, T.; Richardson, M.; Díaz, M.; Hernández, Y. A novel sea anemone peptide that inhibits acid-sensing ion channels. *Peptides* **2014**, *53*, 3-12, doi:10.1016/j.peptides.2013.06.003.
140. Santana, A.; Leite, A.; França, M.; França, L.; Vale, O.; Cunha, R.; Ricart, C.; Sousa, M.; Carvalho, K. Partial sequence and toxic effects of granulitoxin, a neurotoxic peptide from the sea anemone Bunodosoma granulifera. *Brazilian Journal of Medical and Biological Research* **1998**, *31*, 1335-1338.
141. Sachkova, M.Y.; Landau, M.; Surm, J.M.; Macrander, J.; Singer, S.A.; Reitzel, A.M.; Moran, Y. Toxin-like neuropeptides in the sea anemone Nematostella unravel recruitment from the nervous system to venom. *Proc Natl Acad Sci U S A* **2020**, *117*, 27481-27492, doi:10.1073/pnas.2011120117.
142. Mizuno, M.; Nozaki, M.; Morine, N.; Suzuki, N.; Nishikawa, K.; Morgan, B.P.; Matsuo, S. A protein toxin from the sea anemone Phyllodiscus semoni targets the kidney and causes a severe renal injury with predominant glomerular endothelial damage. *Am J Pathol* **2007**, *171*, 402-414, doi:10.2353/ajpath.2007.060984.
143. Razpotnik, A.; Križaj, I.; Kem, W.R.; Maček, P.; Turk, T. A new cytolytic protein from the sea anemone Urticina crassicornis that binds to cholesterol-and sphingomyelin-rich membranes. *Toxicon* **2009**, *53*, 762-769, doi:10.1016/j.toxicon.2009.02.007.
144. Smith, H.L.; Broszczak, D.A.; Bryan, S.E.; Norton, R.S.; Prentis, P.J. Molecular Insights into the Low Complexity Secreted Venom of Calliactis polypus. *Genome Biol Evol* **2024**, *16*, evae154, doi:10.1093/gbe/evae154.
145. Kasheverov, I.E.; Logashina, Y.A.; Kornilov, F.D.; Lushpa, V.A.; Maleeva, E.E.; Korolkova, Y.V.; Yu, J.; Zhu, X.; Zhangsun, D.; Luo, S.; et al. Peptides from the Sea Anemone Metridium senile with Modified Inhibitor Cystine Knot (ICK) Fold Inhibit Nicotinic Acetylcholine Receptors. *Toxins (Basel)* **2022**, *15*, 28, doi:10.3390/toxins15010028.
146. Sachkova, M.Y.; Singer, S.A.; Macrander, J.; Reitzel, A.M.; Peigneur, S.; Tytgat, J.; Moran, Y. The Birth and Death of Toxins with Distinct Functions: A Case Study in the Sea Anemone Nematostella. *Mol Biol Evol* **2019**, *36*, 2001-2012, doi:10.1093/molbev/msz132.

147. Albar, R.A.; Smith, H.L.; Sanches, K.; Wai, D.C.C.; Naseem, M.U.; Szanto, T.G.; Panyi, G.; Prentis, P.J.; Norton, R.S. Structure and functional studies of Avt1, a novel peptide from the sea anemone Aulactinia veratra. *Biochim Biophys Acta Proteins Proteom* **2025**, *1873*, 141050, doi:10.1016/j.bbapap.2024.141050.
148. de Oliveira, J.S.; Zaharenko, A.J.; de Freitas, J.C.; Konno, K.; de Andrade, S.A.; Portaro, F.C.; Richardson, M.; Sant'anna, O.A.; Tambourgi, D.V. Caissarolysin I (Bcs I), a new hemolytic toxin from the Brazilian sea anemone Bunodosoma caissarum: purification and biological characterization. *Biochim Biophys Acta* **2006**, *1760*, 453-461, doi:10.1016/j.bbagen.2005.12.018.
149. Ben-Ari, H.; Paz, M.; Sher, D. The chemical armament of reef-building corals: inter- and intra-specific variation and the identification of an unusual actinoporin in Stylophora pistilata. *Sci Rep* **2018**, *8*, 251, doi:10.1038/s41598-017-18355-1.
150. Nocchi, N.; Gonzalez-Orive, A.; Hernandez-Creus, A.; Lorenzo-Morales, J.; Rodríguez, A.; Morchon, R.; Díaz-Marrero, A.R.; Fernandez, J.J. Alciporin, a pore-forming protein as complementary defense mechanism in Millepora alcicornis. *Frontiers in Marine Science* **2022**, *9*, 914084, doi:10.3389/fmars.2022.914084.
151. Pan, T.; Groger, H.; Schmid, V.; Spring, J. A toxin homology domain in an astacin-like metalloproteinase of the jellyfish Podocoryne carnea with a dual role in digestion and development. *Dev Genes Evol* **1998**, *208*, 259-266, doi:10.1007/s004270050180.
152. So, P.B.; Rubio, P.; Lirio, S.; Macabeo, A.P.; Huang, H.Y.; Corpuz, M.J.; Villaflores, O.B. In vitro angiotensin I converting enzyme inhibition by a peptide isolated from Chiropsalmus quadrigatus Haeckel (box jellyfish) venom hydrolysate. *Toxicon* **2016**, *119*, 77-83, doi:10.1016/j.toxicon.2016.04.050.
153. Qar, J.; Schweitz, H.; Schmid, A.; Lazdunski, M. A polypeptide toxin from the coral Goniopora. Purification and action on Ca2+ channels. *FEBS Lett* **1986**, *202*, 331-336, doi:10.1016/0014-5793(86)80712-8.
154. Yang, Y.; Cun, S.; Peng, L.; Xie, X.; Wei, J.; Yang, W.; Xu, A. cDNA cloning, identification and characterization of a novel cystatin from the tentacle of Cyanea capillata. *Biochimie* **2003**, *85*, 1033-1039, doi:10.1016/s0300-9084(03)00132-9.
155. Iguchi, A.; Iwanaga, S.; Nagai, H. Isolation and characterization of a novel protein toxin from fire coral. *Biochem Biophys Res Commun* **2008**, *365*, 107-112, doi:10.1016/j.bbrc.2007.10.153.
156. Horibata, Y.; Okino, N.; Ichinose, S.; Omori, A.; Ito, M. Purification, characterization, and cDNA cloning of a novel acidic endoglycoceramidase from the jellyfish, Cyanea nozakii. *J Biol Chem* **2000**, *275*, 31297-31304, doi:10.1074/jbc.M003575200.
157. Tamkun, M.M.; Hessinger, D.A. Isolation and partial characterization of a hemolytic and toxic protein from the nematocyst venom of the Portuguese Man-of-War, Physalia physalis. *Biochim Biophys Acta* **1981**, *667*, 87-98, doi:10.1016/0005-2795(81)90069-6.
158. Moran, Y.; Fredman, D.; Szczesny, P.; Grynberg, M.; Technau, U. Recurrent horizontal transfer of bacterial toxin genes to eukaryotes. *Mol Biol Evol* **2012**, *29*, 2223-2230, doi:10.1093/molbev/mss089.
159. Chen, Q.; Deng, C.-S.; Wang, W.-T.; Chen, Y.; Huang, X.-S.; Wang, A.-L.; Xu, N.; He, Y.-L.; Cao, K.-X.; Fan, G.-Y. A dual functional transcriptome-derived Kunitz-like polypeptide from stony coral Porites lutea displays anti-inflammatory and anti-Parkinson's disease effects in vitro and in vivo. *Water Biology and Security* **2025**, 100516, doi:10.1016/j.watbs.2025.100516.

160. Chen, Q.; Deng, C.; Huang, X.; Cao, K.; Chen, H.; Chen, Y.; Mehwish, K.; Xu, Q.; Lu, Q.; Wang, Z.; et al. A novel polypeptide from stony coral Goniopora columna inhibits thrombosis by interfering with the contact-kinin pathway. *Arch Toxicol* **2025**, *99*, 3839-3855, doi:10.1007/s00204-025-04090-4.
161. Béress, L.; Zwick, J. Purification of two crab-paralysing polypeptides from the sea anemone Bolocera tuediae. *Marine Chemistry* **1980**, *8*, 333-338, doi:10.1016/0304-4203(80)90022-5.
162. Cuevas-Cruz, M.; Lazcano-Perez, F.; Hernandez-Guzman, U.; Diaz de la Vega-Castaneda, K.H.; Roman-Gonzalez, S.A.; Valdez-Cruz, N.A.; Velasco-Bejarano, B.; Colin-Gonzalez, A.L.; Santamaria, A.; Gomez-Manzo, S.; et al. A Novel Phospholipase A2 Isolated from Palythoa caribaeorum Possesses Neurotoxic Activity. *Toxins (Basel)* **2019**, *11*, 89, doi:10.3390/toxins11020089.
163. Radwan, F.F.; Aboul-Dahab, H.M. Milleporin-1, a new phospholipase A2 active protein from the fire coral Millepora platyphylla nematocysts. *Comp Biochem Physiol C Toxicol Pharmacol* **2004**, *139*, 267-272, doi:10.1016/j.cca.2004.12.002.
164. Liao, Q.; Gong, G.; Poon, T.C.W.; Ang, I.L.; Lei, K.M.K.; Siu, S.W.I.; Wong, C.T.T.; Radis-Baptista, G.; Lee, S.M. Combined transcriptomic and proteomic analysis reveals a diversity of venom-related and toxin-like peptides expressed in the mat anemone Zoanthus natalensis (Cnidaria, Hexacorallia). *Arch Toxicol* **2019**, *93*, 1745-1767, doi:10.1007/s00204-019-02456-z.
165. Schmidt, C.A.; Cooke, I.; Wilson, D.T.; Miller, D.J.; Peigneur, S.; Tytgat, J.; Field, M.; Takjoo, R.; Smout, M.J.; Loukas, A.J.J.o.N.P. Newly Discovered Peptides from the Coral Heliofungia actiniformis Show Structural and Functional Diversity. **2022**, *85*, 1789-1798.
166. Ashida, K.; Toda, H.; Fujiwara, M.; Sakiyama, F. Amino acid sequence of Goniopora toxin. *Japanese Journal of Pharmacology* **1987**, *43*, 187, doi:10.1016/S0021-5198(19)58365-2.
167. Krishnarjuna, B.; MacRaild, C.A.; Sunanda, P.; Morales, R.A.V.; Peigneur, S.; Macrander, J.; Yu, H.H.; Daly, M.; Raghothama, S.; Dhawan, V.; et al. Structure, folding and stability of a minimal homologue from Anemonia sulcata of the sea anemone potassium channel blocker ShK. *Peptides* **2018**, *99*, 169-178, doi:10.1016/j.peptides.2017.10.001.
168. Delfin, J.; Martinez, I.; Antuch, W.; Morera, V.; Gonzalez, Y.; Rodriguez, R.; Marquez, M.; Saroyan, A.; Larionova, N.; Diaz, J.; et al. Purification, characterization and immobilization of proteinase inhibitors from Stichodactyla helianthus. *Toxicon* **1996**, *34*, 1367-1376, doi:10.1016/s0041-0101(96)00114-6.
169. Minagawa, S.; Sugiyama, M.; Ishida, M.; Nagashima, Y.; Shiomi, K. Kunitz-type protease inhibitors from acrorhagi of three species of sea anemones. *Comp Biochem Physiol B Biochem Mol Biol* **2008**, *150*, 240-245, doi:10.1016/j.cbpb.2008.03.010.
170. Wunderer, G.; Machleidt, W.; Fritz, H. The broad-specificity proteinase inhibitor 5 II from the sea anemone Anemonia sulcata. In *Methods in Enzymology*; Elsevier: 1981; Volume 80, pp. 816-820.
171. Minagawa, S.; Ishida, M.; Shimakura, K.; Nagashima, Y.; Shiomi, K. Isolation and amino acid sequences of two Kunitz-type protease inhibitors from the sea anemone Anthopleura aff. xanthogrammica. *Comp Biochem Physiol B Biochem Mol Biol* **1997**, *118*, 381-386, doi:10.1016/s0305-0491(97)00174-0.

172. Minagawa, S.; Ishida, M.; Shimakura, K.; Nagashima, Y.; Shiomi, K. Amino acid sequence and biological activities of another kunitz-type protease inhibitor isolated from the sea anemone Anthopleura aff. xanthogrammica. *Fisheries science* **1998**, *64*, 155-159, doi:10.2331/fishsci.64.155.
173. Sintsova, O.; Gladkikh, I.; Chausova, V.; Monastyrnaya, M.; Anastyuk, S.; Chernikov, O.; Yurchenko, E.; Aminin, D.; Isaeva, M.; Leychenko, E.; et al. Peptide fingerprinting of the sea anemone Heteractis magnifica mucus revealed neurotoxins, Kunitz-type proteinase inhibitors and a new beta-defensin alpha-amylase inhibitor. *J Proteomics* **2018**, *173*, 12-21, doi:10.1016/j.jprot.2017.11.019.
174. Zykova T.A., V.L.M., Markova L.F., Kozlovskaya E.P., Elyakov G.B. Amino-acid sequence of trypsin inhibitor IV from the sea anemone Radianthus macrodactylus. *Bioorg him* **1985**, *11*, 293-301.
175. Diaz, J.; Morera, V.; Delfin, J.; Huerta, V.; Lima, G.; Rodríguez de la Vega, M.; Garcia, B.; Padron, G.; Assfalg-Machleidt, I.; Machleidt, W. Purification and partial characterization of a novel proteinase inhibitor from the sea anemone Stichodactyla helianthus. *Toxicon* **1998**, *36*, 1275-1276.
176. Sokotun, I.N.; Gnedenko, O.V.; Leichenko, E.V.; Monastyrnaia, M.M.; Kozlovskaia, E.P.; Mol'nar, A.A.; Ivanov, A.C. Interaction investigation of trypsin inhibitor from sea anemone Radianthus macrodactylus with proteases. *Biomed Khim* **2006**, *52*, 595-600.
177. Fujita, S.; Warashina, A. Parasicyonis toxin: effect on crayfish giant axon. *Comp Biochem Physiol C Comp Pharmacol* **1980**, *67C*, 71-74, doi:10.1016/0306-4492(80)90060-x.
178. Li, R.; Yu, H.; Xing, R.; Liu, S.; Qing, Y.; Li, K.; Li, B.; Meng, X.; Cui, J.; Li, P. Isolation, identification and characterization of a novel antioxidant protein from the nematocyst of the jellyfish Stomolophus meleagris. *Int J Biol Macromol* **2012**, *51*, 274-278, doi:10.1016/j.ijbiomac.2012.05.015.
179. Schmidt, C.A.; Wilson, D.T.; Cooke, I.; Potriquet, J.; Tungatt, K.; Muruganandah, V.; Boote, C.; Kuek, F.; Miles, J.J.; Kupz, A.; et al. Identification and Characterization of a Peptide from the Stony Coral Heliofungia actiniformis. *J Nat Prod* **2020**, *83*, 3454-3463, doi:10.1021/acs.jnatprod.0c00981.
180. Junior, V.H.; Zara, F.; Marangoni, S.; Toyama Dde, O.; de Souza, A.J.; de Oliveira, S.C.; Toyama, M.H. Identification of two novel cytolysins from the hydrozoan Olindias sambaquiensis (Cnidaria). *J Venom Anim Toxins Incl Trop Dis* **2014**, *20*, 10, doi:10.1186/1678-9199-20-10.
181. Lazcano-Perez, F.; Vivas, O.; Roman-Gonzalez, S.A.; Rodriguez-Bustamante, E.; Castro, H.; Arenas, I.; Garcia, D.E.; Sanchez-Puig, N.; Arreguin-Espinosa, R. A purified Palythoa venom fraction delays sodium current inactivation in sympathetic neurons. *Toxicon* **2014**, *82*, 112-116, doi:10.1016/j.toxicon.2014.02.013.
182. Edirisinghe, E.A.H.W.; Athukorala, B.N.; Perera, M.; Abeywardana, B.A.S.D.; Sigera, P.S.T.; Eranga, P.; Theekshana, K.D.; Boudjelal, M.; Ali, R.; Peiris, D.C. Jellyfish Venom Peptides Targeting Human Potassium Channels Identified through Ligand Screening: Morphometric and Molecular Identification of the Species and Antibiotic Potential. *Marine Drugs* **2024**, *22*, 333.
183. Menezes, C.; Thakur, N.; Carleer, K.; Peigneur, S.; Tytgat, J. Identification and Functional Characterization of a Nav-Targeting Peptide NavTx-Bg1 from Sea Anemone Bunodosoma goanense (Vennam & den Hartog, 1993) Transcriptome. *Toxicon* **2026**, 109016.

184. Diaz-Garcia, C.; Fuentes-Silva, D.; Sanchez-Soto, C.; Dominguez-Perez, D.; Garcia-Delgado, N.; Varela, C.; Mendoza-Hernandez, G.; Rodriguez-Romero, A.; Castaneda, O.; Hiriart, M. Toxins from Physalia physalis (Cnidaria) raise the intracellular Ca2+ of beta-cells and promote insulin secretion. *Current medicinal chemistry* **2012**, *19*, 5414-5423.
185. Mas, R.; Mene, R.; Garateix, A.; Garcia, M.; Cha, M. Effects of a high molecular weight toxin from Physalia physalis on glutamate responses. *Neuroscience* **1989**, *33*, 269-273.
186. Menéndez, R.; Mas, R.; Garateix, A.; Garcia, M.; Chavez, M. Effects of a high molecular weight polypeptidic toxin from Physalia physalis (Portuguese man-of-war) on cholinergic responses. *Comparative Biochemistry and Physiology Part C: Comparative Pharmacology* **1990**, *95*, 63-69.
187. Lassen, S.; Wiebring, A.; Helmholz, H.; Ruhnau, C.; Prange, A. Isolation of a Nav channel blocking polypeptide from Cyanea capillata medusae–a neurotoxin contained in fishing tentacle isorhizas. *Toxicon* **2012**, *59*, 610-616.
188. Rottini, G.; Gusmani, L.; Parovel, E.; Avian, M.; Patriarca, P. Purification and properties of a cytolytic toxin in venom of the jellyfish Carybdea marsupialis. *Toxicon* **1995**, *33*, 315-326.
189. Sánchez-Rodríguez, J.; Torrens, E.; Segura-Puertas, L. Partial purification and characterization of a novel neurotoxin and three cytolysins from box jellyfish (Carybdea marsupialis) nematocyst venom. *Archives of Toxicology* **2006**, *80*, 163-168.
190. Rastogi, A.; Sarkar, A.; Chakrabarty, D. Partial purification and identification of a metalloproteinase with anticoagulant activity from Rhizostoma pulmo (Barrel Jellyfish). *Toxicon* **2017**, *132*, 29-39.
191. Cariello, L.; Romano, G.; Spagnuolo, A.; Zanetti, L. Isolation and partial characterization of Rhizolysin, a high molecular weight protein with hemolytic activity, from the jellyfish Rhizostoma pulmo. *Toxicon* **1988**, *26*, 1057-1065.
192. Huang, C.; Morlighem, J.R.; Zhou, H.; Lima, E.P.; Gomes, P.B.; Cai, J.; Lou, I.; Perez, C.D.; Lee, S.M.; Radis-Baptista, G. The Transcriptome of the Zoanthid Protopalythoa variabilis (Cnidaria, Anthozoa) Predicts a Basal Repertoire of Toxin-like and Venom-Auxiliary Polypeptides. *Genome Biol Evol* **2016**, *8*, 3045-3064, doi:10.1093/gbe/evw204.
193. Khanal, A. Molecular Cloning and Characterization of a Hemolytic Lectin from Venom of the Stinging Sea Nettle, Chrysaora quinquecirrha. Montclair State University, 2017.
194. Zhang, H.; Pan, X.; Weigang, C. Transcriptomics-driven exploration of genetic variation and peptide discovery in the sea anemones Anthopleura midori and Actinia equina. *Sci Rep* **2025**, *15*, 12061, doi:10.1038/s41598-025-96976-7.
195. Hu, B.; Guo, W.; Wang, L.-h.; Wang, J.-g.; Liu, X.-y.; Jiao, B.-h. Purification and characterization of gigantoxin-4, a new actinoporin from the sea anemone Stichodactyla gigantea. *International journal of biological sciences* **2011**, *7*, 729.
196. Elliott, R.; Konya, R.; Vickneshwara, K. The isolation of a toxin from the dahlia sea anemone, Tealia felina L. *Toxicon* **1986**, *24*, 117-122.
197. Alcaide, M.; Moutinho Cabral, I.; Carvalho, L.; Mendes, V.M.; Alves de Matos, A.P.; Manadas, B.; Saúde, L.; D'Ambrosio, M.; Costa, P.M. A comparative analysis of the venom system between two morphotypes of the sea anemone actinia equina. *Animals* **2024**, *14*, 981, doi:10.3390/ani14060981.

198. Barroso, R.A.; Rodrigues, T.; Campos, A.; Almeida, D.; Guardiola, F.A.; Turkina, M.V.; Antunes, A. Proteomic Diversity of the Sea Anemone Actinia fragacea: Comparative Analysis of Nematocyst Venom, Mucus, and Tissue-Specific Profiles. *Mar Drugs* **2025**, *23*, 79, doi:10.3390/md23020079.
199. Rachamim, T.; Morgenstern, D.; Aharonovich, D.; Brekhman, V.; Lotan, T.; Sher, D. The dynamically evolving nematocyst content of an anthozoan, a scyphozoan, and a hydrozoan. *Mol Biol Evol* **2015**, *32*, 740-753, doi:10.1093/molbev/msu335.
200. Ramirez-Carreto, S.; Vera-Estrella, R.; Portillo-Bobadilla, T.; Licea-Navarro, A.; Bernaldez-Sarabia, J.; Rudino-Pinera, E.; Verleyen, J.J.; Rodriguez, E.; Rodriguez-Almazan, C. Transcriptomic and Proteomic Analysis of the Tentacles and Mucus of Anthopleura dowii Verrill, 1869. *Mar Drugs* **2019**, *17*, 436, doi:10.3390/md17080436.
201. Dominguez-Perez, D.; Campos, A.; Alexei Rodriguez, A.; Turkina, M.V.; Ribeiro, T.; Osorio, H.; Vasconcelos, V.; Antunes, A. Proteomic Analyses of the Unexplored Sea Anemone Bunodactis verrucosa. *Mar Drugs* **2018**, *16*, doi:10.3390/md16020042.
202. Mazzi Esquinca, M.E.; Correa, C.N.; Marques de Barros, G.; Montenegro, H.; Mantovani de Castro, L. Multiomic Approach for Bioprospection: Investigation of Toxins and Peptides of Brazilian Sea Anemone Bunodosoma caissarum. *Mar Drugs* **2023**, *21*, 197, doi:10.3390/md21030197.
203. Hoepner, C.M.; Stewart, Z.K.; Qiao, R.; Fobert, E.K.; Prentis, P.J.; Colella, A.; Chataway, T.; Burke da Silva, K.; Abbott, C.A. Proteotransciptomics of the most popular host sea anemone Entacmaea quadricolor reveals not all toxin genes expressed by tentacles are recruited into its venom arsenal. *Toxins* **2024**, *16*, 85, doi:10.3390/toxins16020085.
204. Li, M.; Mao, K.; Huang, M.; Liao, Y.; Fu, J.; Pan, K.; Shi, Q.; Gao, B. Venomics Reveals the Venom Complexity of Sea Anemone Heteractis magnifica. *Mar Drugs* **2024**, *22*, 71, doi:10.3390/md22020071.
205. Madio, B.; Undheim, E.A.B.; King, G.F. Revisiting venom of the sea anemone Stichodactyla haddoni: Omics techniques reveal the complete toxin arsenal of a well-studied sea anemone genus. *J Proteomics* **2017**, *166*, 83-92, doi:10.1016/j.jprot.2017.07.007.
206. Rodriguez, A.A.; Standker, L.; Zaharenko, A.J.; Garateix, A.G.; Forssmann, W.G.; Beress, L.; Valdes, O.; Hernandez, Y.; Laguna, A. Combining multidimensional liquid chromatography and MALDI-TOF-MS for the fingerprint analysis of secreted peptides from the unexplored sea anemone species Phymanthus crucifer. *J Chromatogr B Analyt Technol Biomed Life Sci* **2012**, *903*, 30-39, doi:10.1016/j.jchromb.2012.06.034.
207. Hwang, D.H.; Prakash, R.L.M.; Asirvatham, R.D.; Kang, C.; Kim, E. Identification and characterization of toxic components of Carybdea brevipedalia venom: implications for understanding box jellyfish (Cnidaria: Cubozoa) envenomation. *Arch Toxicol* **2025**, *99*, 4263-4279, doi:10.1007/s00204-025-04117-w.
208. Jouiaei, M.; Casewell, N.R.; Yanagihara, A.A.; Nouwens, A.; Cribb, B.W.; Whitehead, D.; Jackson, T.N.; Ali, S.A.; Wagstaff, S.C.; Koludarov, I.; et al. Firing the sting: chemically induced discharge of cnidae reveals novel proteins and peptides from box jellyfish (Chironex fleckeri) venom. *Toxins (Basel)* **2015**, *7*, 936-950, doi:10.3390/toxins7030936.
209. Brinkman, D.L.; Jia, X.; Potriquet, J.; Kumar, D.; Dash, D.; Kvaskoff, D.; Mulvenna, J. Transcriptome and venom proteome of the box jellyfish Chironex fleckeri. *BMC Genomics* **2015**, *16*, 407, doi:10.1186/s12864-015-1568-3.

210. Yongstar, C. Venom characterization and toxicology of box jellyfish chironex indrasaksajiae. *Chulalongkorn University Theses and Dissertations (Chula ETD).* **2021**, *10407.*, doi:10.58837/chula.The.2021.1203.
211. Jaimes-Becerra, A.; Chung, R.; Morandini, A.C.; Weston, A.J.; Padilla, G.; Gacesa, R.; Ward, M.; Long, P.F.; Marques, A.C. Comparative proteomics reveals recruitment patterns of some protein families in the venoms of Cnidaria. *Toxicon* **2017**, *137*, 19-26, doi:10.1016/j.toxicon.2017.07.012.
212. Jaimes-Becerra, A.; Gacesa, R.; Doonan, L.B.; Hartigan, A.; Marques, A.C.; Okamura, B.; Long, P.F. "Beyond Primary Sequence"-Proteomic Data Reveal Complex Toxins in Cnidarian Venoms. *Integr Comp Biol* **2019**, *59*, 777-785, doi:10.1093/icb/icz106.
213. Hernandez-Elizarraga, V.H.; Vega-Tamayo, J.E.; Olguin-Lopez, N.; Ibarra-Alvarado, C.; Rojas-Molina, A. Transcriptomic and proteomic analyses reveal the first occurrence of diverse toxin groups in Millepora alcicornis. *J Proteomics* **2023**, *288*, 104984, doi:10.1016/j.jprot.2023.104984.
214. Hernández-Elizárraga, V.H.; Olguín-López, N.; Hernández-Matehuala, R.; Ocharán-Mercado, A.; Cruz-Hernández, A.; Guevara-González, R.G.; Caballero-Pérez, J.; Ibarra-Alvarado, C.; Sánchez-Rodríguez, J.; Rojas-Molina, A. Comparative analysis of the soluble proteome and the cytolytic activity of unbleached and bleached Millepora complanata (“Fire Coral”) from the Mexican Caribbean. *Marine Drugs* **2019**, *17*, 393.
215. Doonan, L.B.; Lynham, S.; Quinlan, C.; Ibiji, S.C.; Winter, C.E.; Padilla, G.; Jaimes-Becerra, A.; Morandini, A.C.; Marques, A.C.; Long, P.F. Venom composition does not vary greatly between different nematocyst types isolated from the primary tentacles of Olindias sambaquiensis (Cnidaria: Hydrozoa). *The Biological Bulletin* **2019**, *237*, 26-35, doi:10.1086/705113.
216. Weston, A.J.; Chung, R.; Dunlap, W.C.; Morandini, A.C.; Marques, A.C.; Moura-da-Silva, A.M.; Ward, M.; Padilla, G.; da Silva, L.F.; Andreakis, N.; et al. Proteomic characterisation of toxins isolated from nematocysts of the South Atlantic jellyfish Olindias sambaquiensis. *Toxicon* **2013**, *71*, 11-17, doi:10.1016/j.toxicon.2013.05.002.
217. Tassara, E.; Miksik, I.; Pompach, P.; Mariottini, G.L.; Xiao, L.; Giovine, M.; Pozzolini, M. Proteomic Analysis and Biochemical Characterization of the Nematocyst Extract of the Hydrozoan Velella velella. *Mar Drugs* **2024**, *22*, 468, doi:10.3390/md22100468.
218. Hartigan, A.; Jaimes-Becerra, A.; Okamura, B.; Doonan, L.B.; Ward, M.; Marques, A.C.; Long, P.F. Recruitment of toxin-like proteins with ancestral venom function supports endoparasitic lifestyles of Myxozoa. *PeerJ* **2021**, *9*, e11208, doi:10.7717/peerj.11208.
219. Americus, B.; Hams, N.; Klompen, A.M.L.; Alama-Bermejo, G.; Lotan, T.; Bartholomew, J.L.; Atkinson, S.D. The cnidarian parasite Ceratonova shasta utilizes inherited and recruited venom-like compounds during infection. *PeerJ* **2021**, *9*, e12606, doi:10.7717/peerj.12606.
220. Modica, M.V.; Leone, S.; Gerdol, M.; Greco, S.; Aurelle, D.; Oliverio, M.; Fassio, G.; El Koulali, K.; Barrachina, C.; Dutertre, S. The proteotranscriptomic characterization of venom in the white seafan Eunicella singularis elucidates the evolution of Octocorallia arsenal. *Open Biol* **2025**, *15*, 250015, doi:10.1098/rsob.250015.
221. Radwan, F.F.; Aboul-Dahab, H.M.; Burnett, J.W. Some toxicological characteristics of three venomous soft corals from the Red Sea. *Comp Biochem Physiol C Toxicol Pharmacol* **2002**, *132*, 25-35, doi:10.1016/s1532-0456(02)00045-5.

222. Gacesa, R.; Chung, R.; Dunn, S.R.; Weston, A.J.; Jaimes-Becerra, A.; Marques, A.C.; Morandini, A.C.; Hranueli, D.; Starcevic, A.; Ward, M.; et al. Gene duplications are extensive and contribute significantly to the toxic proteome of nematocysts isolated from Acropora digitifera (Cnidaria: Anthozoa: Scleractinia). *BMC Genomics* **2015**, *16*, 774, doi:10.1186/s12864-015-1976-4.
223. Kitahara, M.V.; Jaimes-Becerra, A.; Gamero-Mora, E.; Padilla, G.; Doonan, L.B.; Ward, M.; Marques, A.C.; Morandini, A.C.; Long, P.F. Reciprocal transplantation of the heterotrophic coral Tubastraea coccinea (Scleractinia: Dendrophylliidae) between distinct habitats did not alter its venom toxin composition. *Ecology and Evolution* **2020**, *10*, 1794-1803, doi:10.1002/ece3.5959.
224. Garcia-Arredondo, A.; Rojas-Molina, A.; Ibarra-Alvarado, C.; Lazcano-Perez, F.; Arreguin-Espinosa, R.; Sanchez-Rodriguez, J. Composition and biological activities of the aqueous extracts of three scleractinian corals from the Mexican Caribbean: Pseudodiploria strigosa, Porites astreoides and Siderastrea siderea. *J Venom Anim Toxins Incl Trop Dis* **2016**, *22*, 32, doi:10.1186/s40409-016-0087-2.
225. Riyas, A.; Kumar, A.; Chandran, M.; Jaleel, A.; Biju Kumar, A. The venom proteome of three common scyphozoan jellyfishes (Chrysaora caliparea, Cyanea nozakii and Lychnorhiza malayensis) (Cnidaria: Scyphozoa) from the coastal waters of India. *Toxicon* **2021**, *195*, 93-103, doi:10.1016/j.toxicon.2021.03.005.
226. Ponce, D.; Brinkman, D.L.; Potriquet, J.; Mulvenna, J. Tentacle Transcriptome and Venom Proteome of the Pacific Sea Nettle, Chrysaora fuscescens (Cnidaria: Scyphozoa). *Toxins (Basel)* **2016**, *8*, 102, doi:10.3390/toxins8040102.
227. Wang, C.; Wang, B.; Wang, B.; Wang, Q.; Liu, G.; Wang, T.; He, Q.; Zhang, L. Unique Diversity of Sting-Related Toxins Based on Transcriptomic and Proteomic Analysis of the Jellyfish Cyanea capillata and Nemopilema nomurai (Cnidaria: Scyphozoa). *J Proteome Res* **2019**, *18*, 436-448, doi:10.1021/acs.jproteome.8b00735.
228. Li, R.; Yu, H.; Yue, Y.; Liu, S.; Xing, R.; Chen, X.; Li, P. Combined proteomics and transcriptomics identifies sting-related toxins of jellyfish Cyanea nozakii. *J Proteomics* **2016**, *148*, 57-64, doi:10.1016/j.jprot.2016.07.023.
229. Liang, H.; Jiang, G.; Wang, T.; Zhang, J.; Liu, W.; Xu, Z.; Zhang, J.; Xiao, L. An integrated transcriptomic and proteomic analysis reveals toxin arsenal of a novel Antarctic jellyfish Cyanea sp. *J Proteomics* **2019**, *208*, 103483, doi:10.1016/j.jprot.2019.103483.
230. Choudhary, I.; Hwang, D.H.; Lee, H.; Yoon, W.D.; Chae, J.; Han, C.H.; Yum, S.; Kang, C.; Kim, E. Proteomic Analysis of Novel Components of Nemopilema nomurai Jellyfish Venom: Deciphering the Mode of Action. *Toxins (Basel)* **2019**, *11*, 153, doi:10.3390/toxins11030153.
231. Li, R.; Yu, H.; Li, T.; Li, P. Comprehensive Proteome Reveals the Key Lethal Toxins in the Venom of Jellyfish Nemopilema nomurai. *J Proteome Res* **2020**, *19*, 2491-2500, doi:10.1021/acs.jproteome.0c00277.
232. Frazao, B.; Campos, A.; Osorio, H.; Thomas, B.; Leandro, S.; Teixeira, A.; Vasconcelos, V.; Antunes, A. Analysis of Pelagia noctiluca proteome Reveals a Red Fluorescent Protein, a Zinc Metalloproteinase and a Peroxiredoxin. *Protein J* **2017**, *36*, 77-97, doi:10.1007/s10930-017-9695-0.
233. Yang, F.; Ye, R.; Ma, C.; Wang, Y.; Wang, Y.; Chen, J.; Yang, J.; Hofer, J.; Zhu, Y.; Xiao, L.; et al. Toxicity evaluation, toxin screening and its intervention of the jellyfish Phacellophora camtschatica based on a combined transcriptome-proteome analysis. *Ecotoxicol Environ Saf* **2022**, *233*, 113315, doi:10.1016/j.ecoenv.2022.113315.

234. Rodrigues, T.; Barroso, R.A.; Campos, A.; Almeida, D.; Guardiola, F.A.; Turkina, M.V.; Antunes, A. Unveiling the Bioactive Potential of the Invasive Jellyfish Phyllorhiza punctata Through Integrative Transcriptomic and Proteomic Analyses. *Biomolecules* **2025**, *15*, 1121, doi:10.3390/biom15081121.
235. Leung, T.C.N.; Qu, Z.; Nong, W.; Hui, J.H.L.; Ngai, S.M. Proteomic Analysis of the Venom of Jellyfishes Rhopilema esculentum and Sanderia malayensis. *Mar Drugs* **2020**, *18*, 655, doi:10.3390/md18120655.
236. Li, R.; Yu, H.; Xue, W.; Yue, Y.; Liu, S.; Xing, R.; Li, P. Jellyfish venomics and venom gland transcriptomics analysis of Stomolophus meleagris to reveal the toxins associated with sting. *J Proteomics* **2014**, *106*, 17-29, doi:10.1016/j.jprot.2014.04.011.
237. Li, X.; Ma, X.; Chen, X.; Wang, T.; Liu, Q.; Wang, Y.; Li, Z.; Höfer, J.; Li, F.; Xiao, L. The medusa of Aurelia coerulea is similar to its polyp in molecular composition and different from the medusa of Stomolophus meleagris in toxicity. *Toxicon* **2022**, *210*, 89-99.
238. Banagouro, K.C.Q.; Viana, J.; de Lima, L.P.; Coelho, G.R.; Rocha, T.; Girardello, R.; Russi, K.L.; Kitahara, M.V.; Sciani, J.M. Biochemical and Toxinological Characterization of Venom from Macrorhynchia philippina (Cnidaria, Hydrozoa). *Biomed Res Int* **2022**, *2022*, 8170252, doi:10.1155/2022/8170252.
239. Kvetkina, A.; Kostina, E.; Gladkikh, I.; Chausova, V.; Yurchenko, E.; Bakunina, I.; Pivkin, M.; Anastyuk, S.; Popov, R.; Monastyrnaya, M.; et al. Deep-Sea Anemones Are Prospective Source of New Antimicrobial and Cytotoxic Compounds. *Mar Drugs* **2021**, *19*, 654, doi:10.3390/md19120654.
240. Gladkikh, I.; Kvetkina, A.; Kostina, E.; Kalina, R.; Grebnev, B.; Koshelev, S.; Kozlov, S.; Monastyrnaya, M.; Kozlovskaya, E. Peptide modulators of ASIC channels of the sea anemone Urticina aff. coriacea (Cuvier, 1798) from the Sea of Okhotsk. *Russian Journal of Marine Biology* **2018**, *44*, 458-464.
241. Klompen, A.M.L.; Macrander, J.; Reitzel, A.M.; Stampar, S.N. Transcriptomic Analysis of Four Cerianthid (Cnidaria, Ceriantharia) Venoms. *Mar Drugs* **2020**, *18*, 413, doi:10.3390/md18080413.
242. Lopes, C.S.; Iwama, R.E.; Cortez, T.; Andrade, S.C.; Klompen, A.M.; Audino, J.A.; Macrander, J.; Reitzel, A.M.; Nagata, R.M.; Lanna, E. Evolutionary insights into toxins diversity in Ceriantharia (Cnidaria; Anthozoa). *Toxicon: X* **2025**, 100227, doi:10.1016/j.toxcx.2025.100227.
243. Xiao, B.; Guo, Q.; Zhai, Y.; Gu, Z. Transcriptomic Insights into the Diversity and Evolution of Myxozoa (Cnidaria, Endocnidozoa) Toxin-like Proteins. *Mar Drugs* **2022**, *20*, 291, doi:10.3390/md20050291.
244. Macrander, J.; Brugler, M.R.; Daly, M. A RNA-seq approach to identify putative toxins from acrorhagi in aggressive and non-aggressive Anthopleura elegantissima polyps. *BMC Genomics* **2015**, *16*, 221, doi:10.1186/s12864-015-1417-4.
245. Lewis Ames, C.; Ryan, J.F.; Bely, A.E.; Cartwright, P.; Collins, A.G. A new transcriptome and transcriptome profiling of adult and larval tissue in the box jellyfish Alatina alata: an emerging model for studying venom, vision and sex. *BMC genomics* **2016**, *17*, 650, doi:10.1186/s12864-016-2944-3.
246. Macrander, J.; Broe, M.; Daly, M. Tissue-Specific Venom Composition and Differential Gene Expression in Sea Anemones. *Genome Biol Evol* **2016**, *8*, 2358-2375, doi:10.1093/gbe/evw155.

247. Bliese, A.D. Transcriptomic Identification of Venom Candidates in the Clinging Jellyfish Gonionemus vertens. *Theses, Dissertations and Culminating Projects. 629.* **2020**.
248. Delgado, A.; Benedict, C.; Macrander, J.; Daly, M. Never, Ever Make an Enemy... Out of an Anemone: Transcriptomic Comparison of Clownfish Hosting Sea Anemone Venoms. *Mar Drugs* **2022**, *20*, 730, doi:10.3390/md20120730.
249. Yosef, O.; Popovits, Y.; Malik, A.; Ofek-Lalzer, M.; Mass, T.; Sher, D. A tentacle for every occasion: comparing the hunting tentacles and sweeper tentacles, used for territorial competition, in the coral Galaxea fascicularis. *BMC Genomics* **2020**, *21*, 548, doi:10.1186/s12864-020-06952-w.
250. Drake, J.L.; Malik, A.; Popovits, Y.; Yosef, O.; Shemesh, E.; Stolarski, J.; Tchernov, D.; Sher, D.; Mass, T.J.F.i.M.S. Physiological and transcriptomic variability indicative of differences in key functions within a single coral colony. **2021**, 768.
251. Moran, Y.; Genikhovich, G.; Gordon, D.; Wienkoop, S.; Zenkert, C.; Ozbek, S.; Technau, U.; Gurevitz, M. Neurotoxin localization to ectodermal gland cells uncovers an alternative mechanism of venom delivery in sea anemones. *Proc Biol Sci* **2012**, *279*, 1351-1358, doi:10.1098/rspb.2011.1731.
252. Moran, Y.; Weinberger, H.; Reitzel, A.M.; Sullivan, J.C.; Kahn, R.; Gordon, D.; Finnerty, J.R.; Gurevitz, M. Intron retention as a posttranscriptional regulatory mechanism of neurotoxin expression at early life stages of the starlet anemone Nematostella vectensis. *Journal of Molecular biology* **2008**, *380*, 437-443, doi:10.1016/j.jmb.2008.05.011.
